# Supplementary material for: Dynamic SAS-6 phosphorylation aids centrosome duplication and elimination in C. elegans oogenesis
Source: EMBO Rep. 2025 May 23;26(13):3411–44. doi: 10.1038/s44319-025-00485-7 (PMC12238530; doi:10.1038/s44319-025-00485-7)
Supplement: Supplementary file 1 — Table EV1 [file 44319_2025_485_MOESM1_ESM.docx]

**Table EV1. List of *C. elegans* strains used in this study.**

| **Strain name & Genotype** | **Source** | **Identifier** |
| --- | --- | --- |
| *C. elegans:* N2 wild type (Bristol) | Caenorhabditis Genetics Center | N2 |
| *C. elegans: ieSi64[gld-1p::tir1::mRuby::gld-1 3’UTR + Cbr-unc-119(+)] II; unc-119(ed3) III* | Caenorhabditis Genetics Center | CA1352 |
| *C. elegans:* *sas-7(or1940[gfp::sas-7]) III; Itls37[pie-1p::mCherry::his-58 + unc-119(+)] IV* | Caenorhabditis Genetics Center | EU3000 |
| *C. elegans: Psas-6::sas-6::gfp; Ppie-1::mCherry::sas-4* | Li et al., 2017 | GOU899 |
| *C. elegans: unc-119(ed3) III; ddls36[pie-1p::gfp::sas-5 + unc-119(+)]*; *sas-6(syb1706[sas-6::degron::flag::ha::flag::ha]) IV; ieSi64[gld-1p::tir1::mRuby::gld-1 3’UTR + Cbr-unc-119(+)] II; unc-119(ed3) III* | This Paper | MGC595 |
| *C. elegans: sas-7(or1940[gfp::sas-7]) III; Itls37[pie-1p::mCherry::his-58 + unc-119(+)]IV*; *sas-6(syb1706[sas-6::degron::flag::ha::flag::ha]) IV; ieSi64[gld-1p::tir1::mRuby::gld-1 3’UTR + Cbr-unc-119(+)] II; unc-119(ed3) III* | This Paper | MGC618 |
| *C. elegans: unc-119(ed3) III; ddls36[pie-1p::gfp::sas-5 + unc-119(+)]*; *sas-6(syb3988[sas-6(5A)::degron::flag::ha::flag::ha])IV;ieSi64[gld1p::tir1::mRuby::gld-1 3’UTR + Cbr-unc-119(+)] II; unc-119(ed3) III* | This Paper | MGC619 |
| *C. elegans: sas-7(or1940[gfp::sas-7]) III; Itls37[pie-1p::mCherry::his-58 + unc-119(+)]IV*; *sas-6(syb3988[sas-6(5A)::degron::flag::ha::flag::ha]) IV; ieSi64[gld-1p::tir1::mRuby::gld-1 3’UTR + Cbr-unc-119(+)] II; unc-119(ed3) III* | This Paper | MGC620 |
| *C. elegans: unc-119(ed3) III; ddls36[pie-1p::gfp::sas-5 + unc-119(+)]*; *sas-6(syb5687[sas-6(5D)::degron::4xflag])IV/nT1[myo-2p::GFP+pes-10p::GFP+F22B7.9p::GFP qIs51] (IV;V); ieSi64[gld-1p::tir1::mRuby::gld-1 3’UTR + Cbr-unc-119(+)] II; unc-119(ed3) III* | This Paper | MGC621 |
| *C. elegans: sas-7(or1940[gfp::sas-7]) III; Itls37[pie-1p::mCherry::his-58 + unc-119(+)]IV*; *sas-6(syb5687[sas-6(5D)::degron::4xflag]) IV / nT1[myo-2p::GFP+pes-10p::GFP+F22B7.9p::GFP qIs51] (IV;V); ieSi64[gld-1p::tir1::mRuby::gld-1 3’UTR + Cbr-unc-119(+)] II; unc-119(ed3) III* | This Paper | MGC622 |
| *C. elegans: sas-6(syb1706[sas-6::degron::flag::ha::flag::ha]) IV* | This Paper | PHX1706 |
| *C. elegans:* *sas-6(syb3988[sas-6(5A)::degron::flag::ha::flag::ha]) IV* | This Paper | PHX3988 |
| *C. elegans: sas-6(syb5687[sas-6(5D)::degron::4xflag]) IV /nT1[myo-2p::GFP+pes-10p::GFP+F22B7.9p::GFP qIs51] (IV;V)* | This Paper | PHX5687 |
| *C. elegans: unc-119(ed3) III; ddEx10[pie-1p::gfp::sas-4 + unc-119(+)]* | Caenorhabditis Genetics Center | TH26 |
| *C. elegans: unc-119(ed3) III; ddls36[pie-1p::gfp::sas-5 + unc-119(+)]* | Caenorhabditis Genetics Center | TH61 |
| *C. elegans: cdk-1(q923[3xMyc::cdk-1])* | Caenorhabditis Genetics Center | JK5751 |
| *C. elegans: neSi12 [cdk-1::GFP + Cbr-unc-119(+)] II; unc-119(ed3) III* | Caenorhabditis Genetics Center | WM242 |
| *C. elegans: cdk-1(ne2257)* | Caenorhabditis Genetics Center | WM99 |
