# Supplementary material for: Dynamic SAS-6 phosphorylation aids centrosome duplication and elimination in C. elegans oogenesis
Source: EMBO Rep. 2025 May 23;26(13):3411–44. doi: 10.1038/s44319-025-00485-7 (PMC12238530; doi:10.1038/s44319-025-00485-7)
Supplement: Supplementary file 2 — Appendix [file 44319_2025_485_MOESM2_ESM.pdf]

EMBOR-2024-60173V3

Appendix

Dynamic SAS-6 phosphorylation aids centrosome duplication and elimination in *C. elegans* oogenesis

Feifei Qi, Shanshan Yin, Xiangrui Yang, Ning Ju, Bohan Liu, Xing Zhang, Zixuan Zhu, Li Ji, Fuxin Zhang, Li Zhao, Ruoxi Wang, Min Liu, Liangran Zhang, Huijie Zhao, Jun Zhou, and Jinmin Gao

Table of Content

Appendix Figure S1……………………………………2
Appendix Figure S2……………………………………3
Appendix Figure S3……………………………………4
Appendix Figure S4……………………………………5
Appendix Figure S5……………………………………6

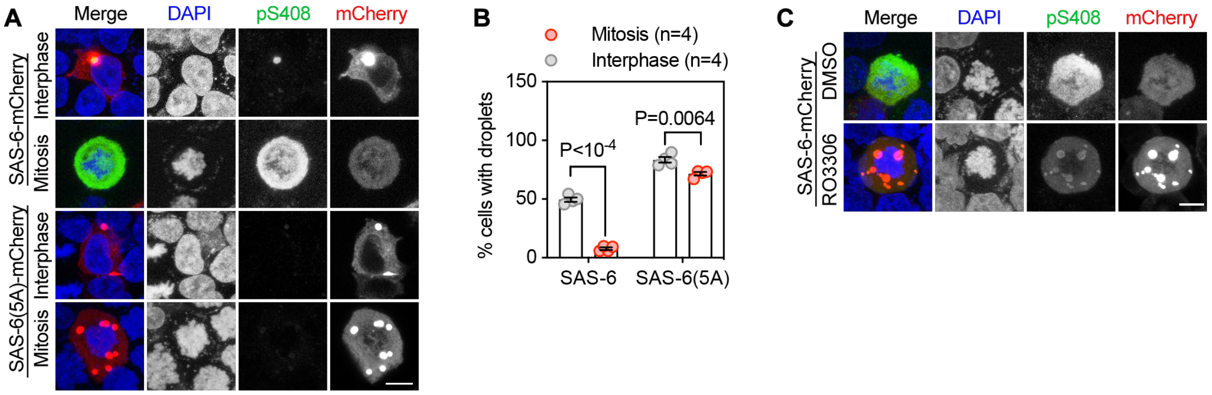

Appendix Figure S1. SAS-6 phosphorylation and droplet formation in HEK293T cells.
(A) Immunofluorescence images droplet formation by exogenously expressed wild-type SAS-6 and SAS-6(5A) during interphase and mitosis in HEK293T cells. Phosphorylation of SAS-6 at S408 was detected using an anti-pS408 antibody. Scale bar,10 µm.
(B) Quantification of transfected HEK293T cells exhibiting droplet formation by wild-type or mutant SAS-6 proteins, as shown in (A).
(C) Immunofluorescence images of SAS-6-transfected HEK293T cells stained with anti-pS408 antibody following treatment with or without the CDK1 inhibitor RO3306. All eight mitotic cells expressing mCherry-tagged SAS-6, across three biological replicates, consistently exhibited the phenotype shown in the representative images. Scale bar,10 µm.

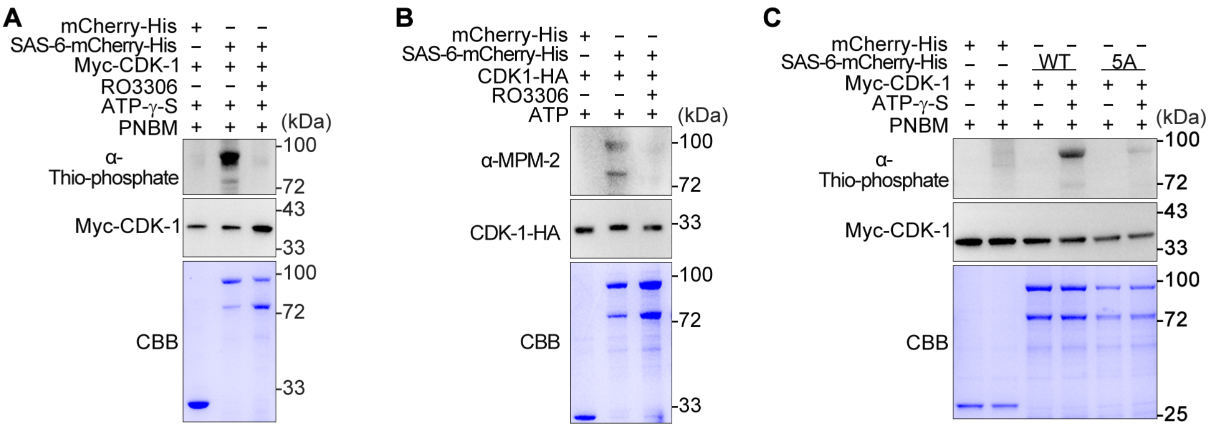

Appendix Figure S2. CDK1 inhibitor RO3306 and SAS-6(5A) mutation block SAS-6 phosphorylation.

(A) *In vitro* kinase assay using *C. elegans* CDK-1 and purified SAS-6-mCherry-His, with or without the CDK1 inhibitor RO3306. Protein phosphorylation was assessed by Western blot (WB) using an anti-thiophosphate ester antibody.

(B) *In vitro* kinase assay using human CDK1 and purified SAS-6-mCherry-His, with or without the CDK1 inhibitor RO3306. Protein phosphorylation was assessed by WB using an anti-MPM-2 antibody.

(C) *In vitro* kinase assay using *C. elegans* CDK-1 and purified SAS-6-mCherry-His or SAS-6(5A)-mCherry-His. Protein phosphorylation was assessed by WB using an anti-thiophosphate ester antibody.

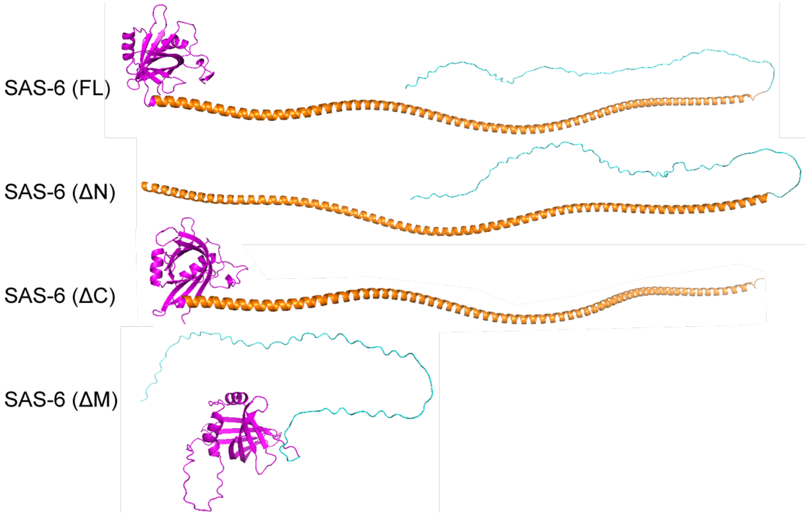

Appendix Figure S3. Structure prediction of full-length (FL) and truncated SAS-6 mutants using AlphaFold3. The N-terminal domain is presented in magenta, the central coiled-coil region in orange, and the C-terminal region in cyan.

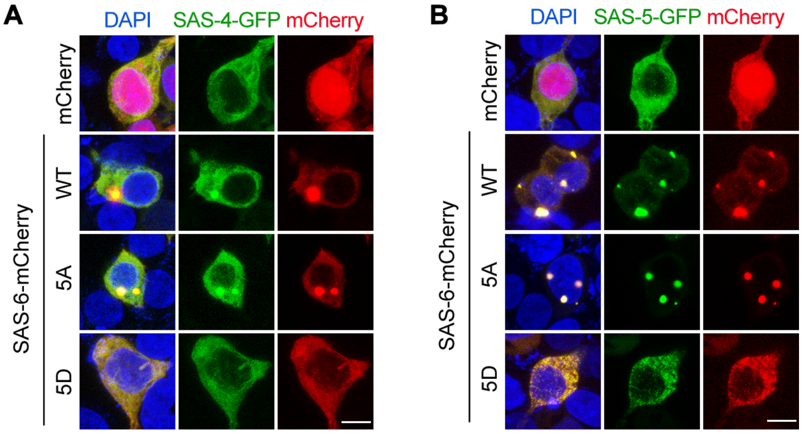

Appendix Figure S4. Co-recruitment analysis of SAS-6 with SAS-4 and SAS-5 in HEK293T cells.

(A) Co-expression of SAS-4-GFP (green) and the indicated mCherry-tagged proteins (red) in HEK293T cells. Nuclear DNA was stained with DAPI (blue). The phenotype was consistently observed in all 15 transfected cells across three biological replicates. Scale bar, 10 µm. **(B)** Co-expression of SAS-5-GFP (green) and the indicated mCherry-tagged proteins (red) in HEK293T cells. Nuclear DNA was stained with DAPI (blue). The phenotype was consistently observed in all 15 transfected cells across three biological replicates. Scale bar, 10 µm.

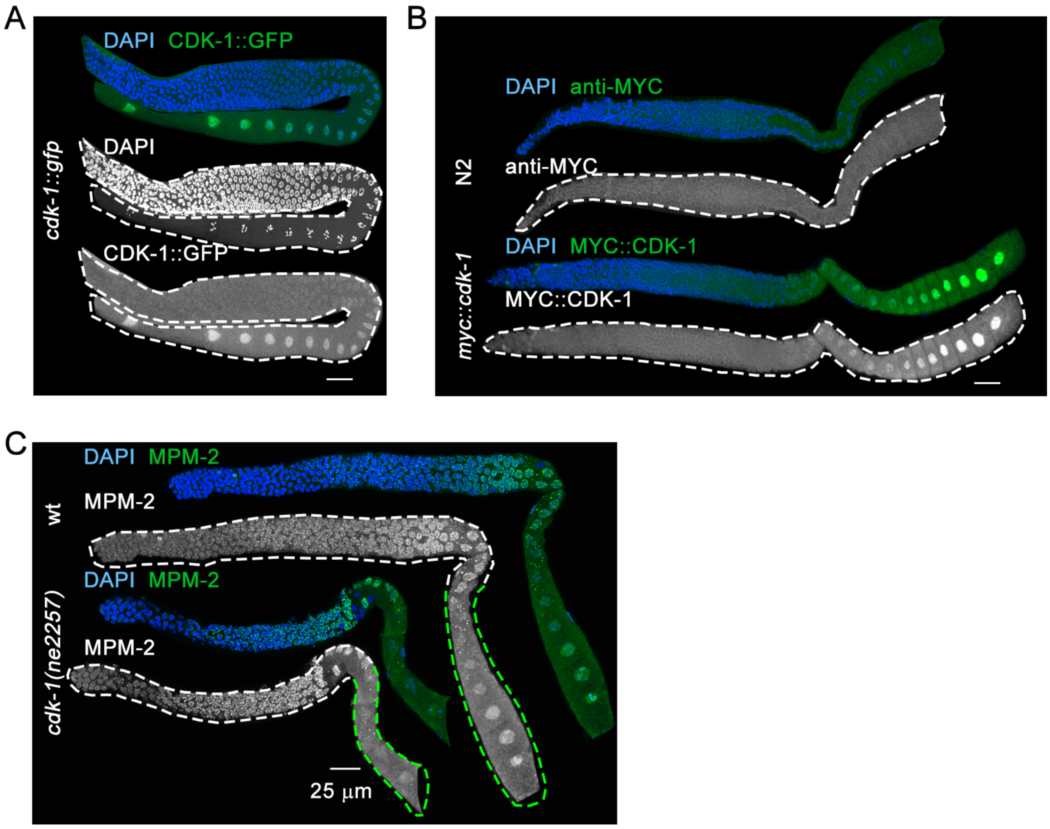

Appendix Figure S5. CDK-1 expression pattern and MPM-2 staining in *C. elegans* germline.

(A) Immunofluorescence images showing the expression pattern of CDK-1::GFP (green) in the gonad. A consistent expression pattern was observed in all six gonads from three biological replicates. Scale bar, 25 μm.

(B) Immunofluorescence images showing the expression pattern of MYC::CDK-1 (green) in the gonad. A consistent expression pattern was observed in all six gonads from three biological replicates. Scale bar, 25 μm.

(C) MPM-2 (green) immunostaining of gonads from worms with the indicated genotypes. All six gonads examined per genotype showed a consistent phenotype. Scale bar, 25 μm.
