# Supplementary figures and images for: Dynamic SAS-6 phosphorylation aids centrosome duplication and elimination in C. elegans oogenesis (part 1 of 3)
Source: EMBO Rep. 2025 May 23;26(13):3411–44. doi: 10.1038/s44319-025-00485-7 (PMC12238530; doi:10.1038/s44319-025-00485-7)

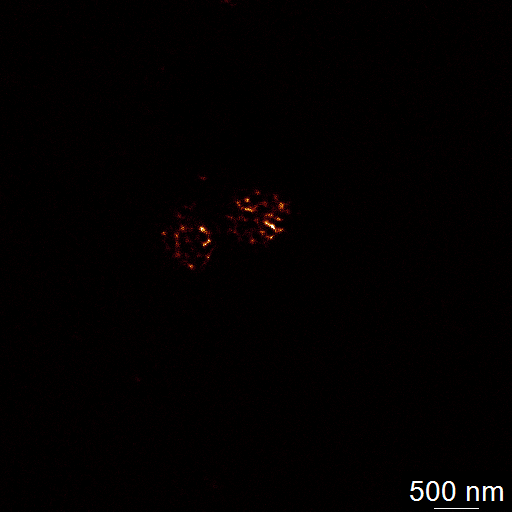

Supplement: Supplementary file 4 — Source data Fig. 1 [file 44319_2025_485_MOESM4_ESM.zip › Figure 1/1G/Fig. 1G_Control.tif]

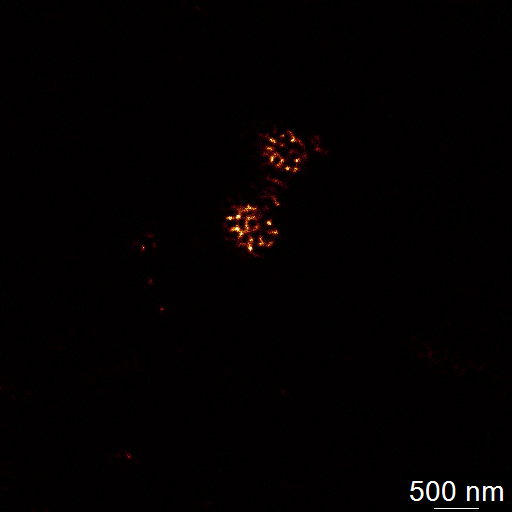

Supplement: Supplementary file 4 — Source data Fig. 1 [file 44319_2025_485_MOESM4_ESM.zip › Figure 1/1G/Fig. 1G_Hex.tif]

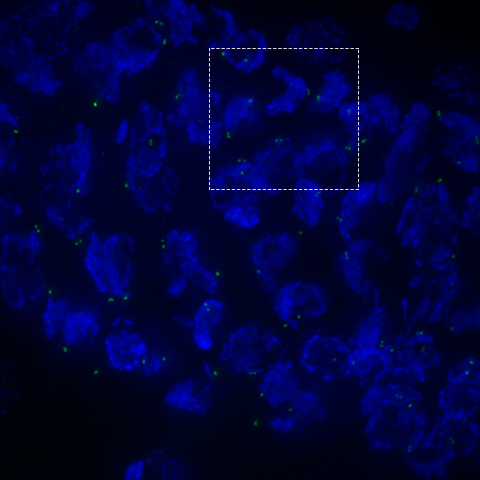

Supplement: Supplementary file 4 — Source data Fig. 1 [file 44319_2025_485_MOESM4_ESM.zip › Figure 1/1B/Fig.1B_gfp_sas_5_TZ.tif]

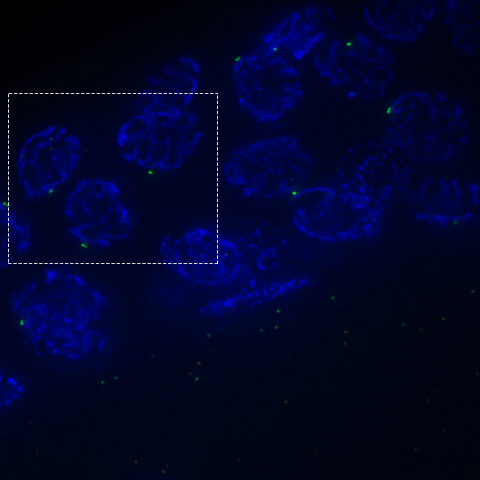

Supplement: Supplementary file 4 — Source data Fig. 1 [file 44319_2025_485_MOESM4_ESM.zip › Figure 1/1B/Fig.1B_gfp_sas_5_Pachytene.tif]

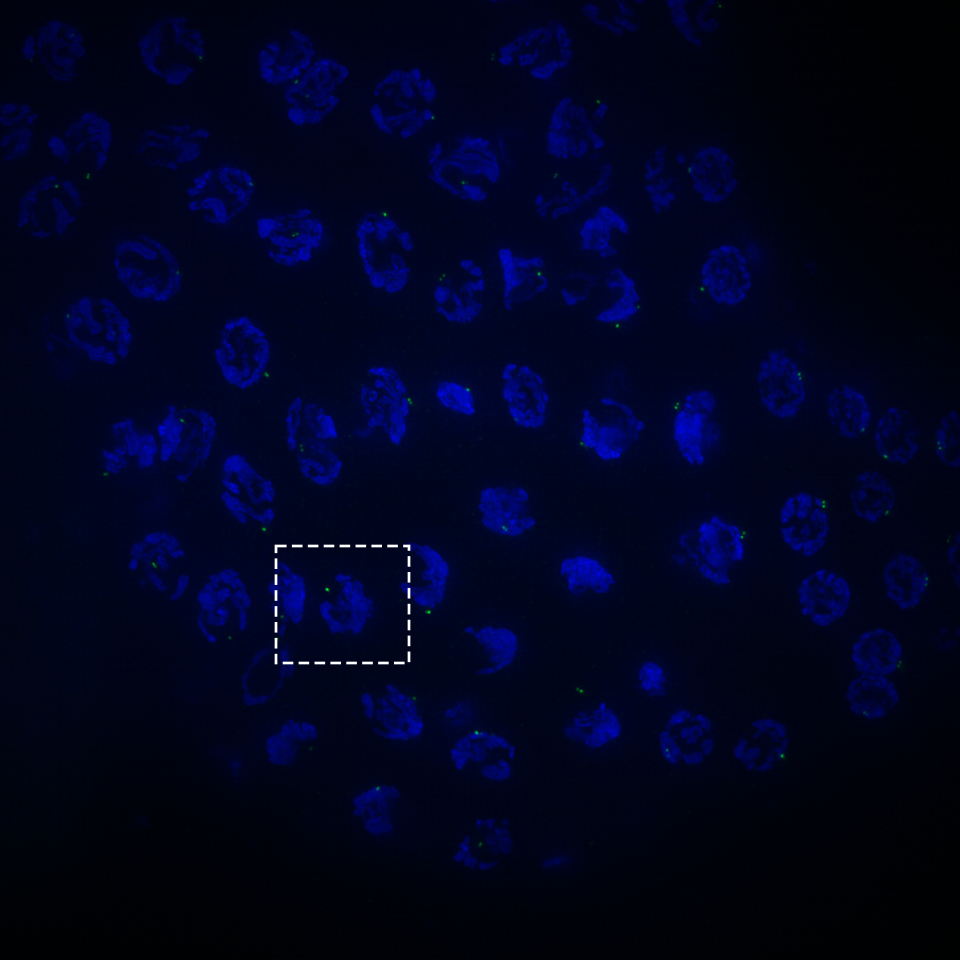

Supplement: Supplementary file 4 — Source data Fig. 1 [file 44319_2025_485_MOESM4_ESM.zip › Figure 1/1B/Fig.1B_sas6_gfp_TZ.tif]

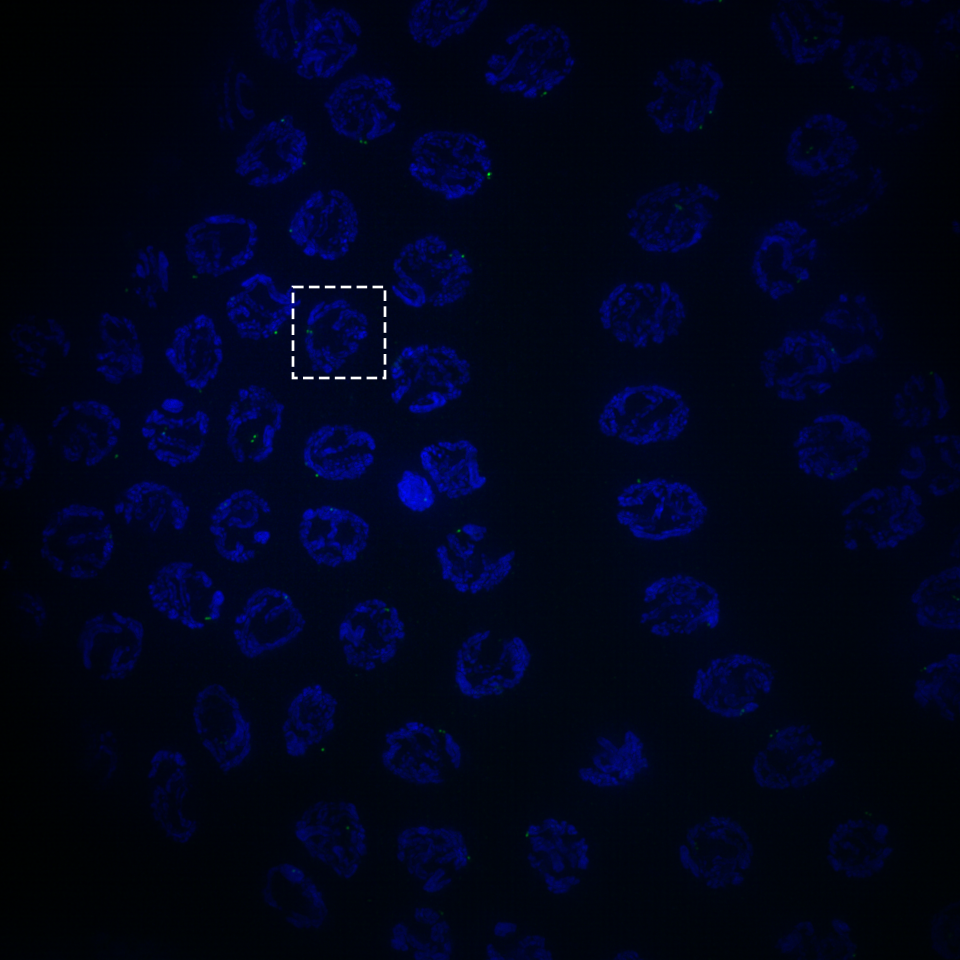

Supplement: Supplementary file 4 — Source data Fig. 1 [file 44319_2025_485_MOESM4_ESM.zip › Figure 1/1B/Fig.1B_sas6_gfp_Pachytene.tif]

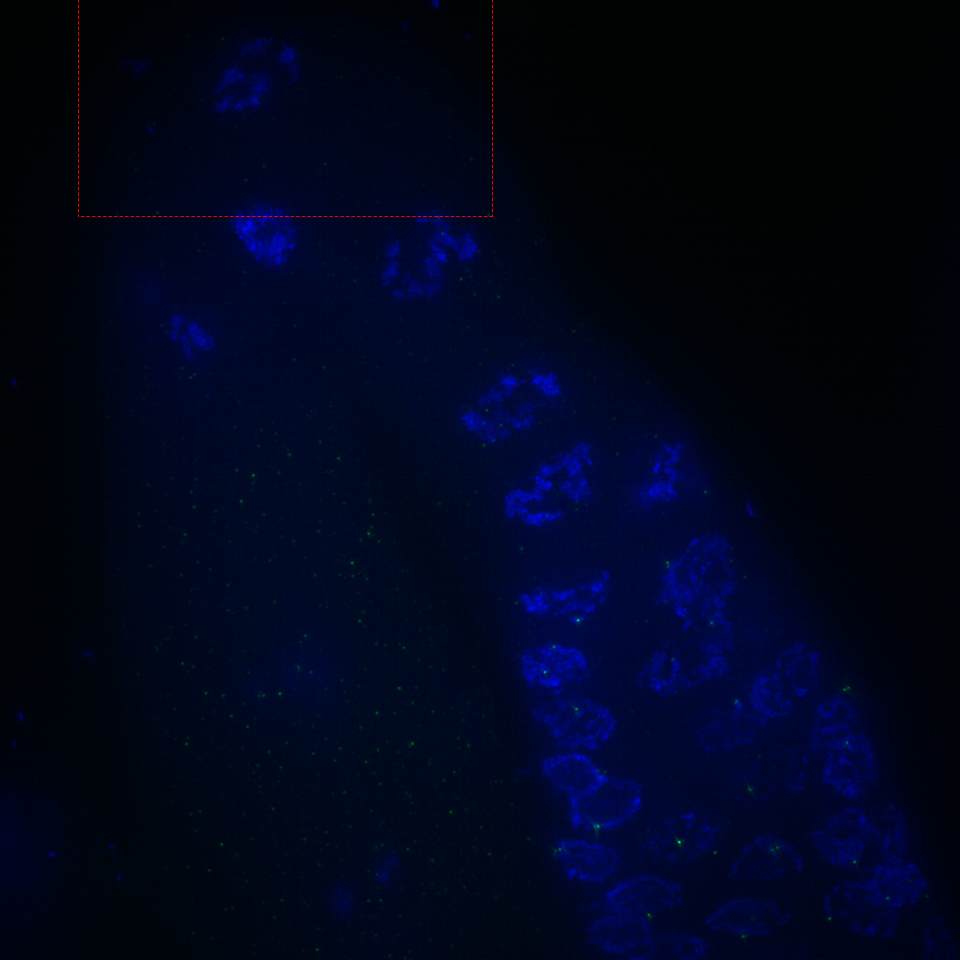

Supplement: Supplementary file 4 — Source data Fig. 1 [file 44319_2025_485_MOESM4_ESM.zip › Figure 1/1B/Fig.1B_gfp_sas_5_DIP.tif]

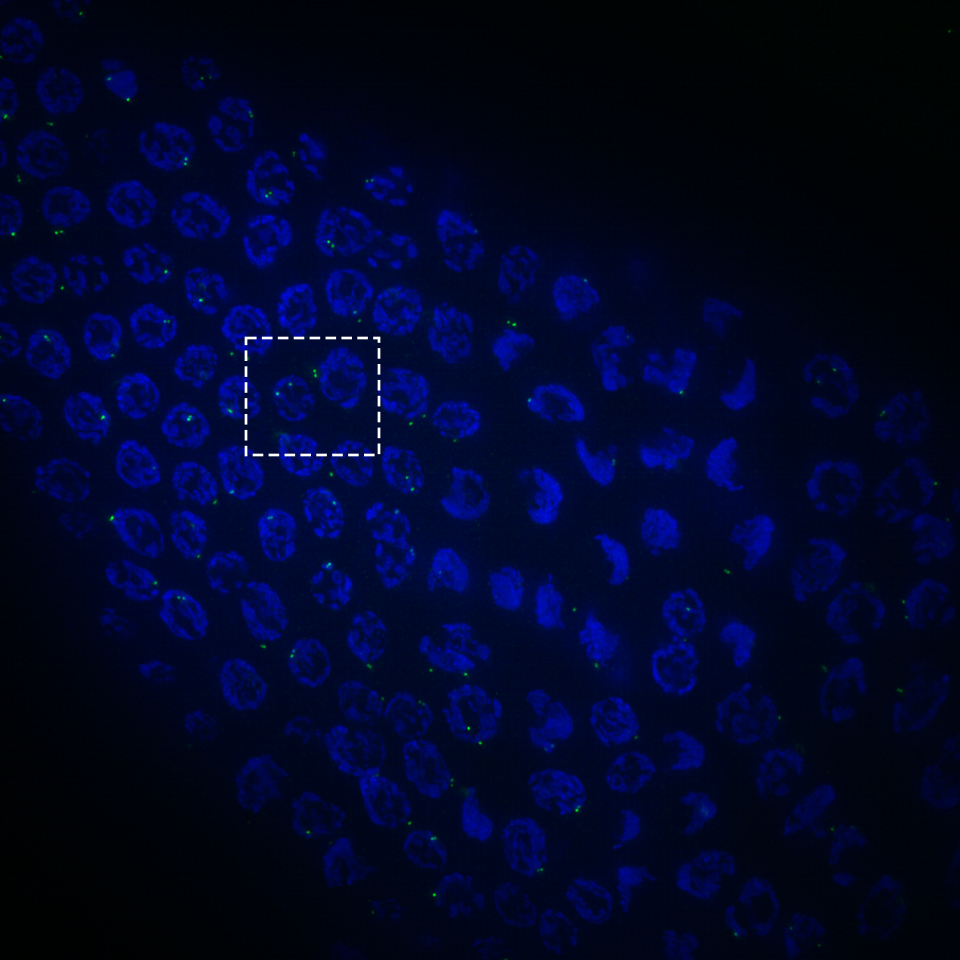

Supplement: Supplementary file 4 — Source data Fig. 1 [file 44319_2025_485_MOESM4_ESM.zip › Figure 1/1B/Fig.1B_sas6_gfp_PMT.tif]

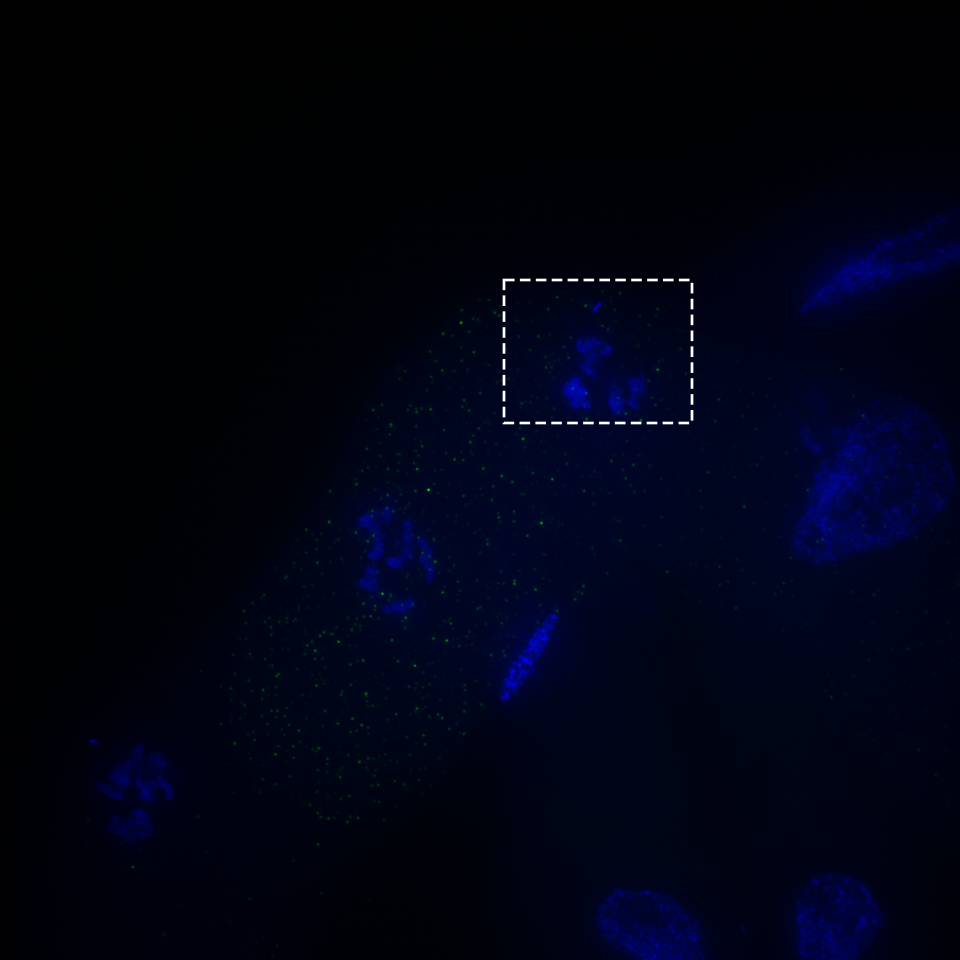

Supplement: Supplementary file 4 — Source data Fig. 1 [file 44319_2025_485_MOESM4_ESM.zip › Figure 1/1B/Fig.1B_sas6_gfp_DIP.tif]

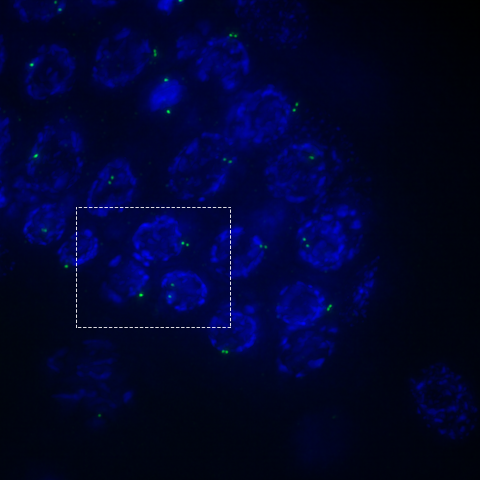

Supplement: Supplementary file 4 — Source data Fig. 1 [file 44319_2025_485_MOESM4_ESM.zip › Figure 1/1B/Fig.1B_gfp_sas_5_PMT.tif]

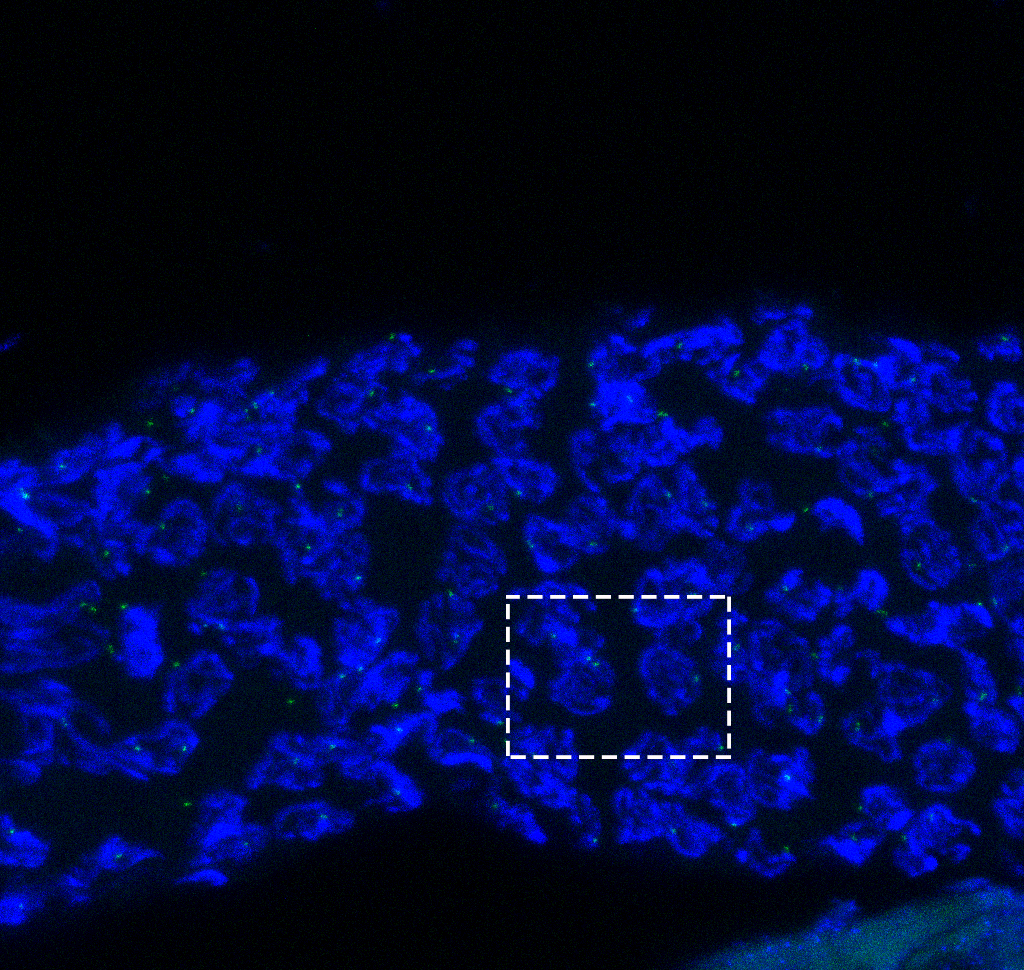

Supplement: Supplementary file 4 — Source data Fig. 1 [file 44319_2025_485_MOESM4_ESM.zip › Figure 1/1E/Fig. 1E_gfp_sas_5_0min.tif]

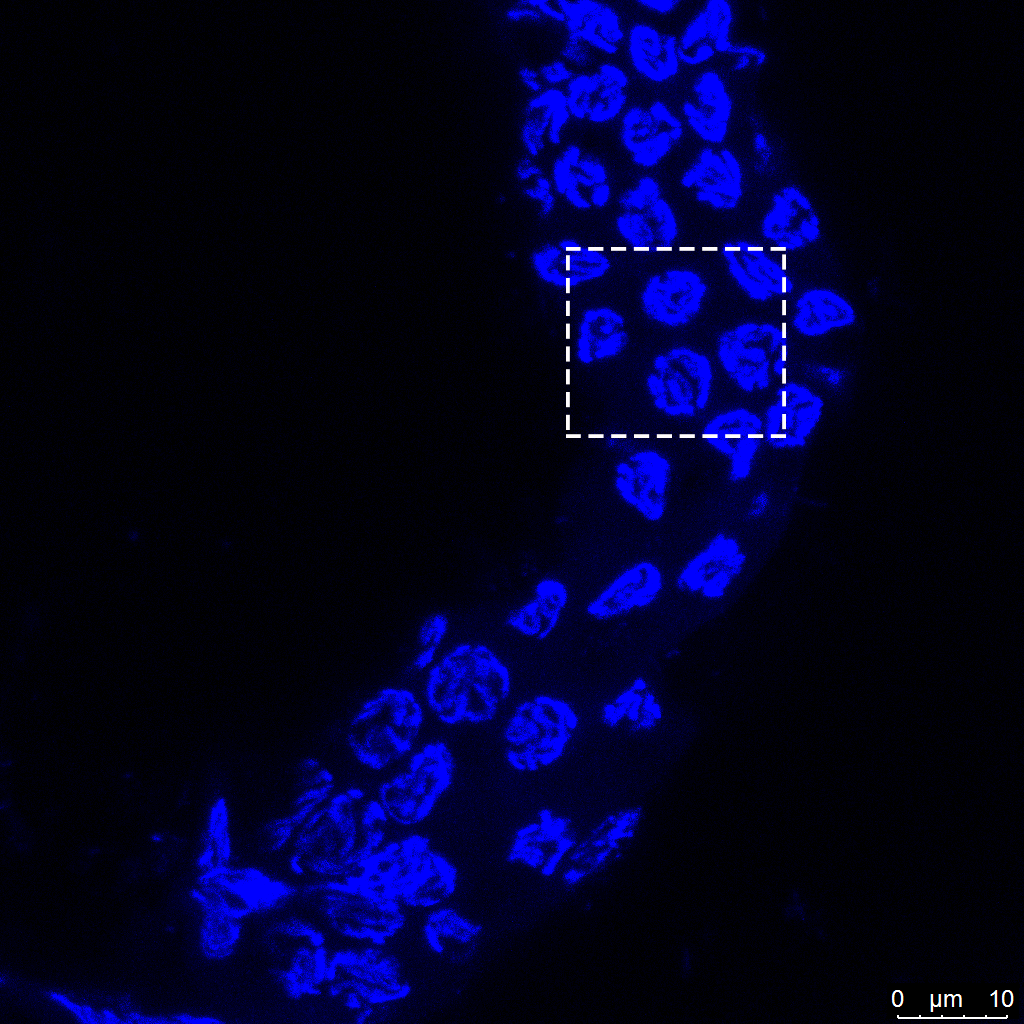

Supplement: Supplementary file 4 — Source data Fig. 1 [file 44319_2025_485_MOESM4_ESM.zip › Figure 1/1E/Fig. 1E_sas6_gfp_washout 15min.tif]

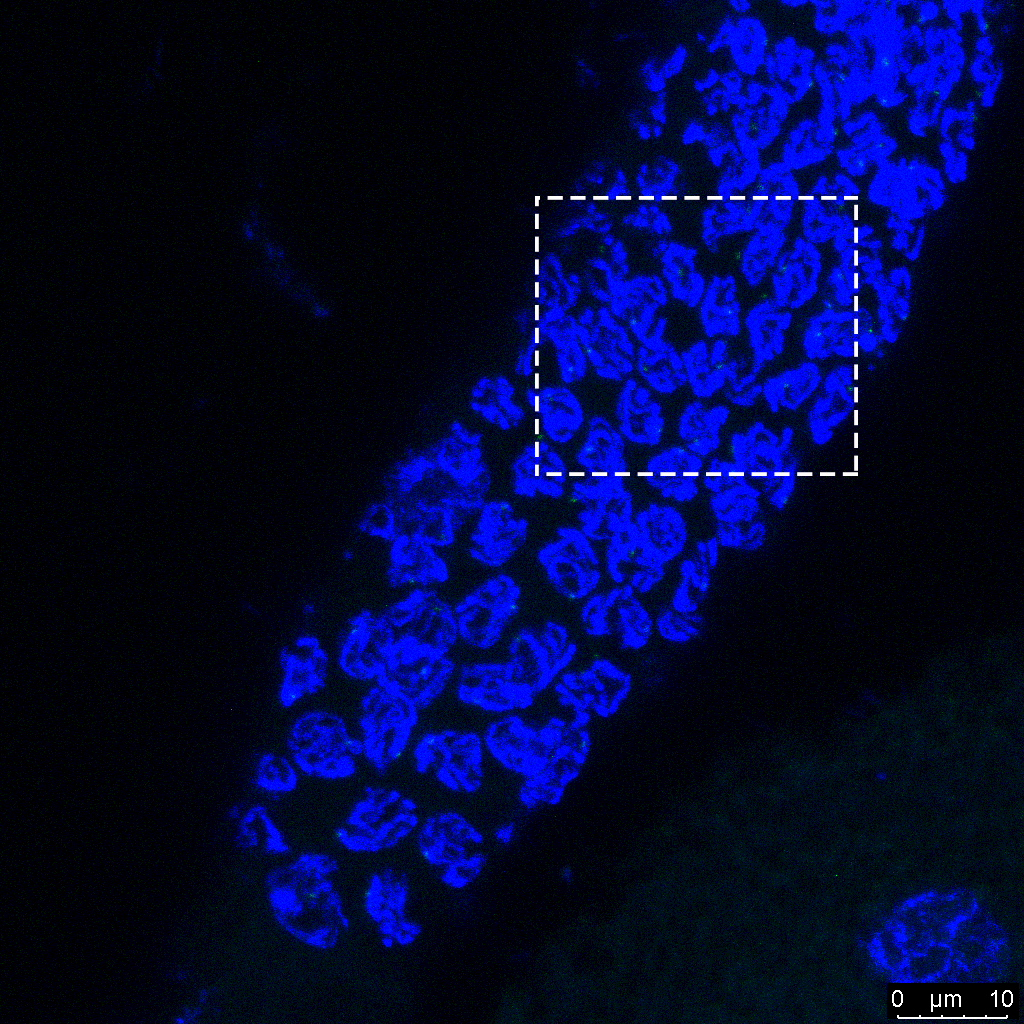

Supplement: Supplementary file 4 — Source data Fig. 1 [file 44319_2025_485_MOESM4_ESM.zip › Figure 1/1E/Fig. 1E_sas6_gfp_0 min.tif]

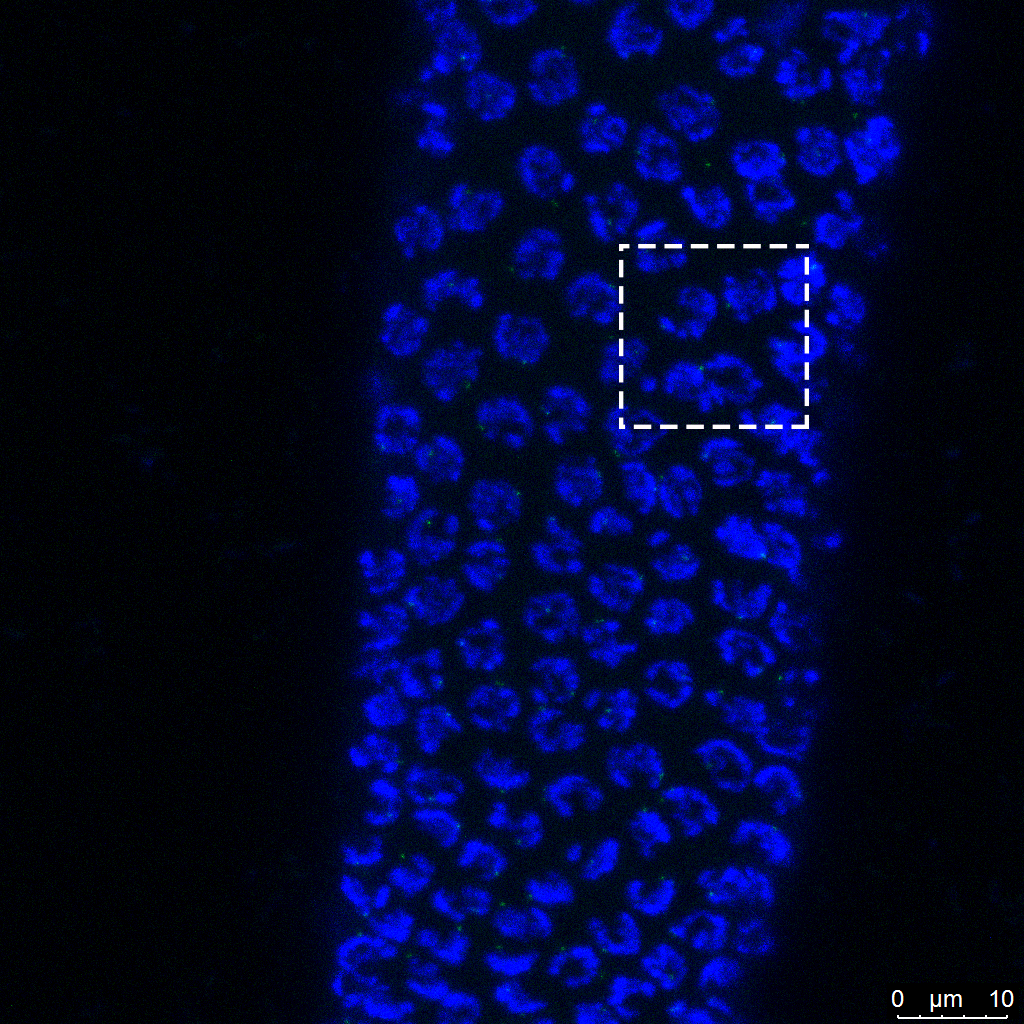

Supplement: Supplementary file 4 — Source data Fig. 1 [file 44319_2025_485_MOESM4_ESM.zip › Figure 1/1E/Fig. 1E_gfp_sas_5_10min.tif]

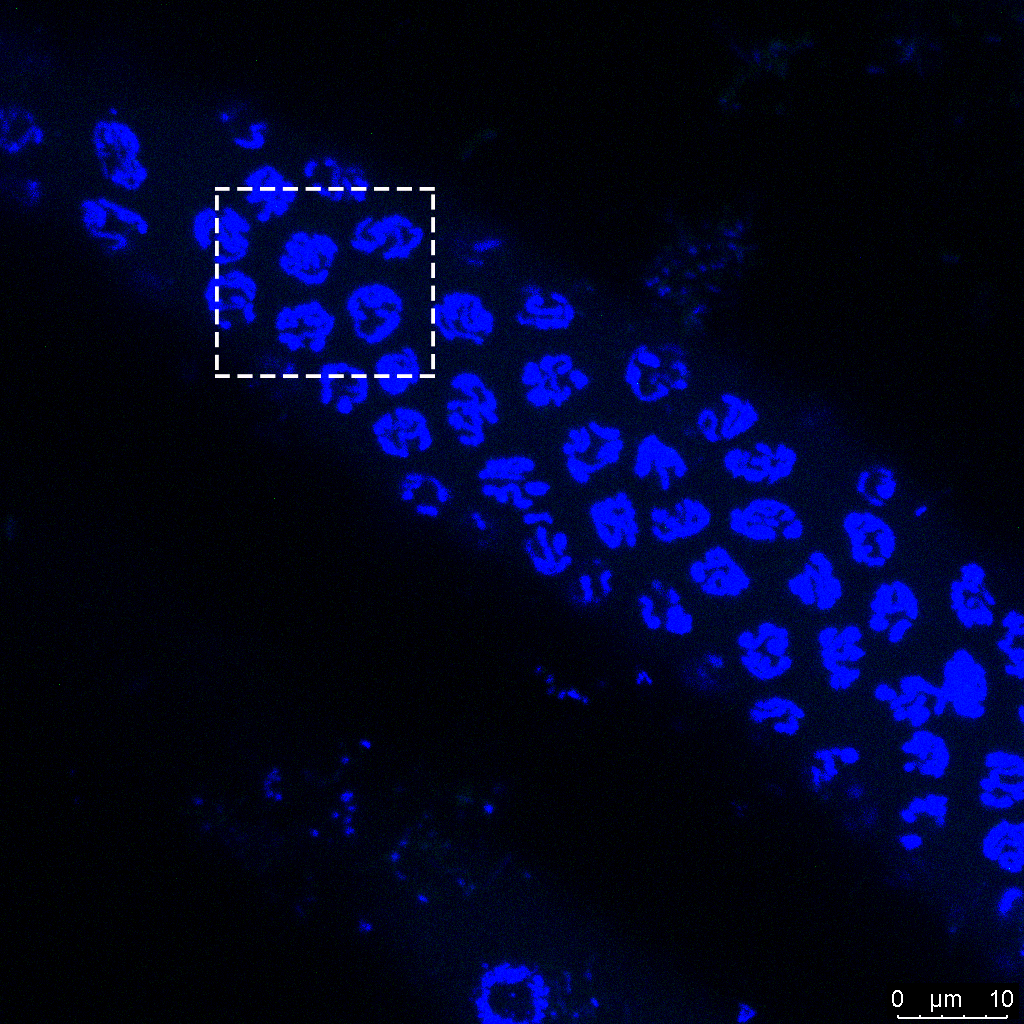

Supplement: Supplementary file 4 — Source data Fig. 1 [file 44319_2025_485_MOESM4_ESM.zip › Figure 1/1E/Fig. 1E_sas6_gfp_5min.tif]

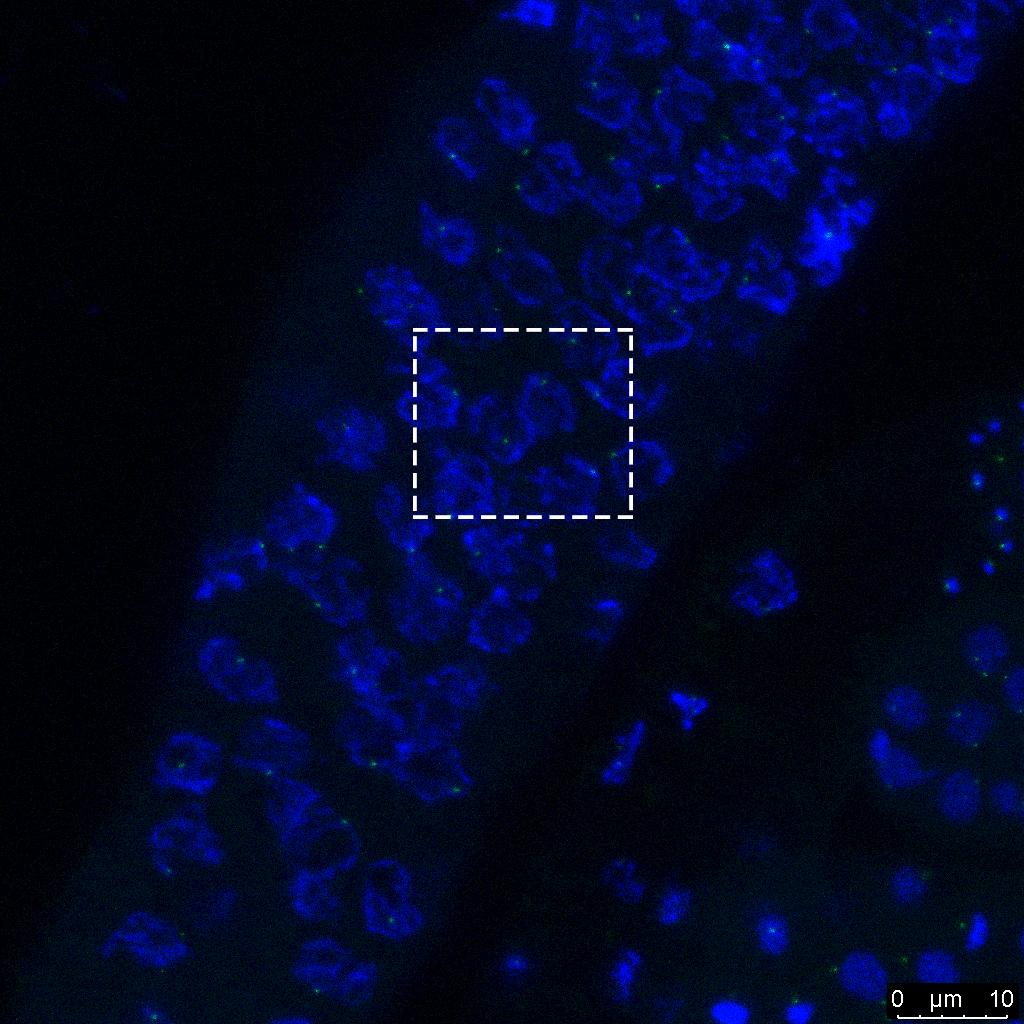

Supplement: Supplementary file 4 — Source data Fig. 1 [file 44319_2025_485_MOESM4_ESM.zip › Figure 1/1E/Fig. 1E_gfp_sas_5_15min.tif]

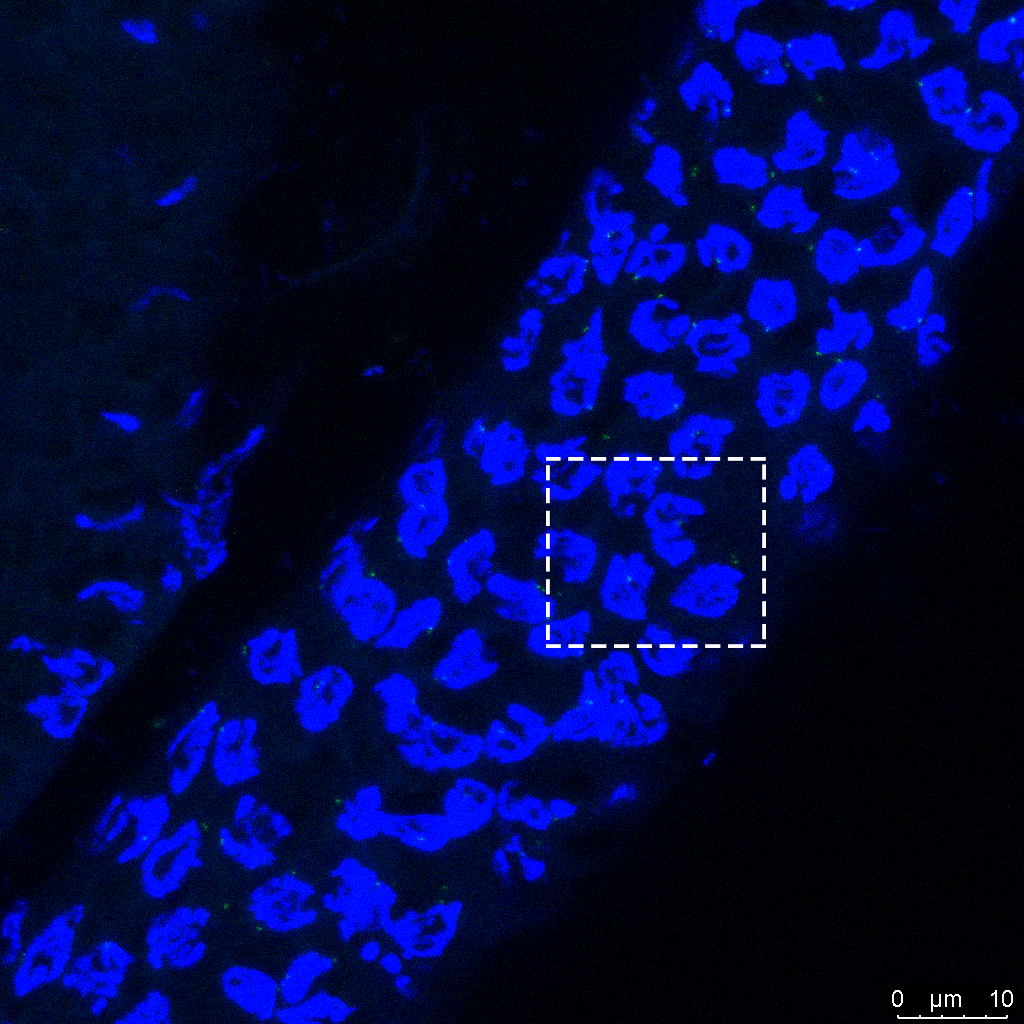

Supplement: Supplementary file 4 — Source data Fig. 1 [file 44319_2025_485_MOESM4_ESM.zip › Figure 1/1E/Fig. 1E_sas6_gfp_washout 10min.tif]

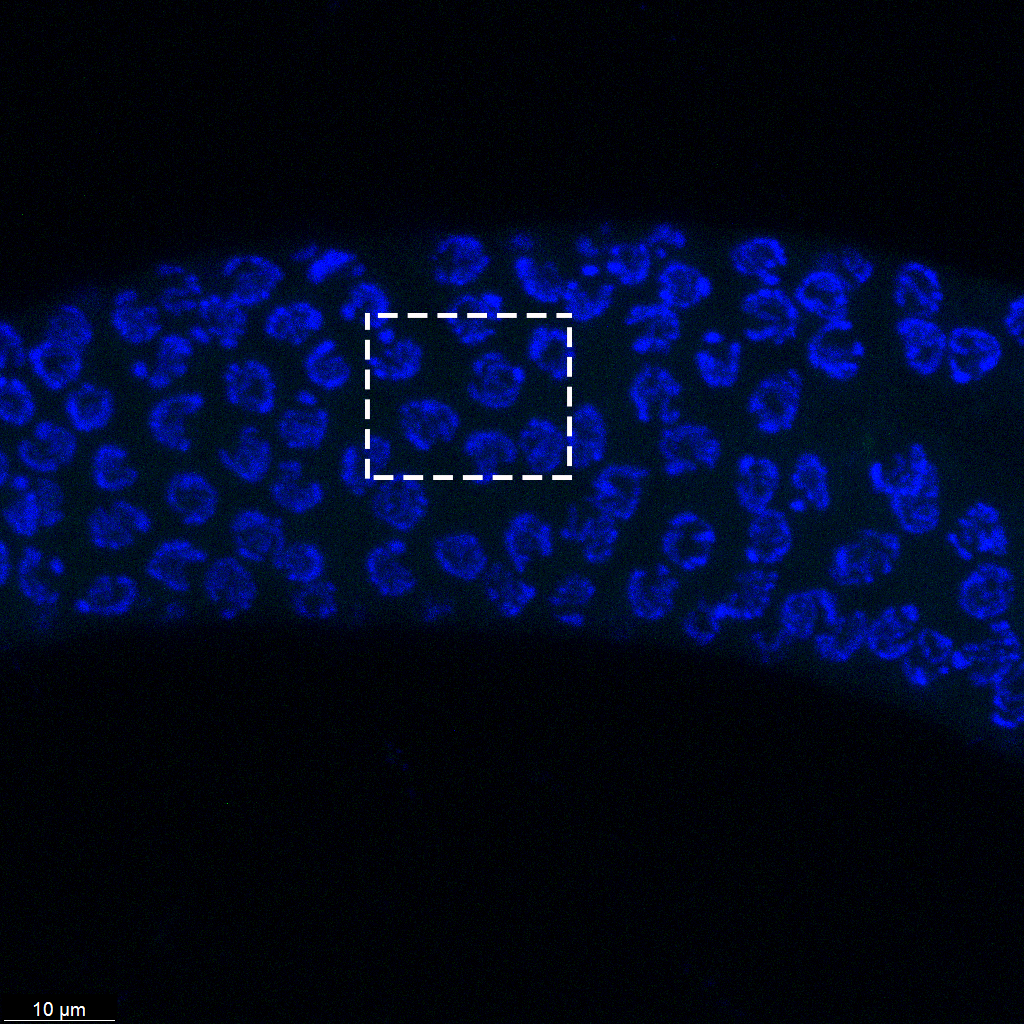

Supplement: Supplementary file 4 — Source data Fig. 1 [file 44319_2025_485_MOESM4_ESM.zip › Figure 1/1E/Fig. 1E_gfp_sas_5_5min.tif]

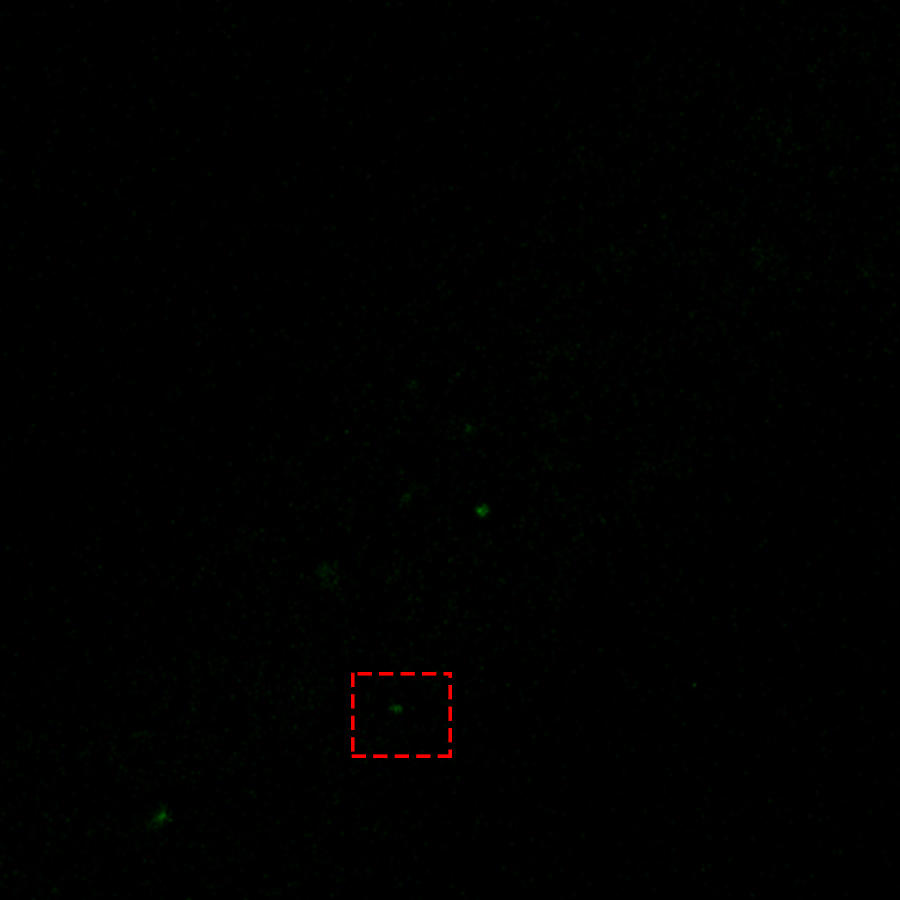

Supplement: Supplementary file 4 — Source data Fig. 1 [file 44319_2025_485_MOESM4_ESM.zip › Figure 1/1I/1I_sas6_gfp_FRAP/1I_sas6_gfp_prebleach.tif]

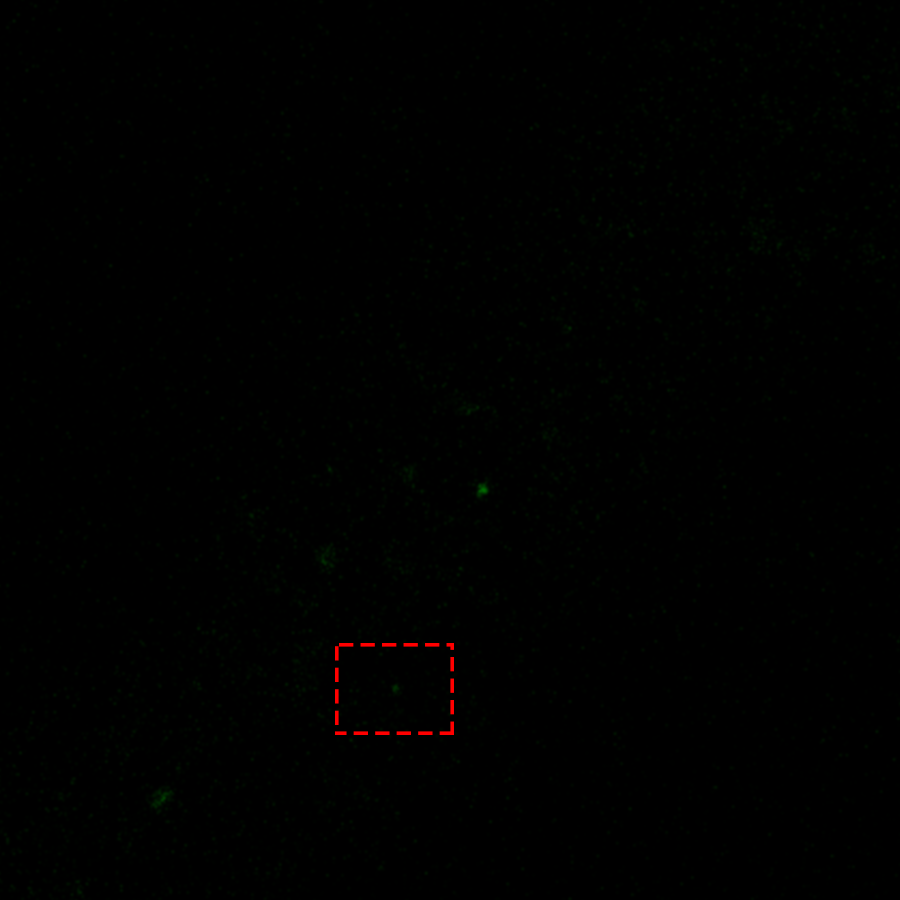

Supplement: Supplementary file 4 — Source data Fig. 1 [file 44319_2025_485_MOESM4_ESM.zip › Figure 1/1I/1I_sas6_gfp_FRAP/1I_sas6_gfp_40''.tif]

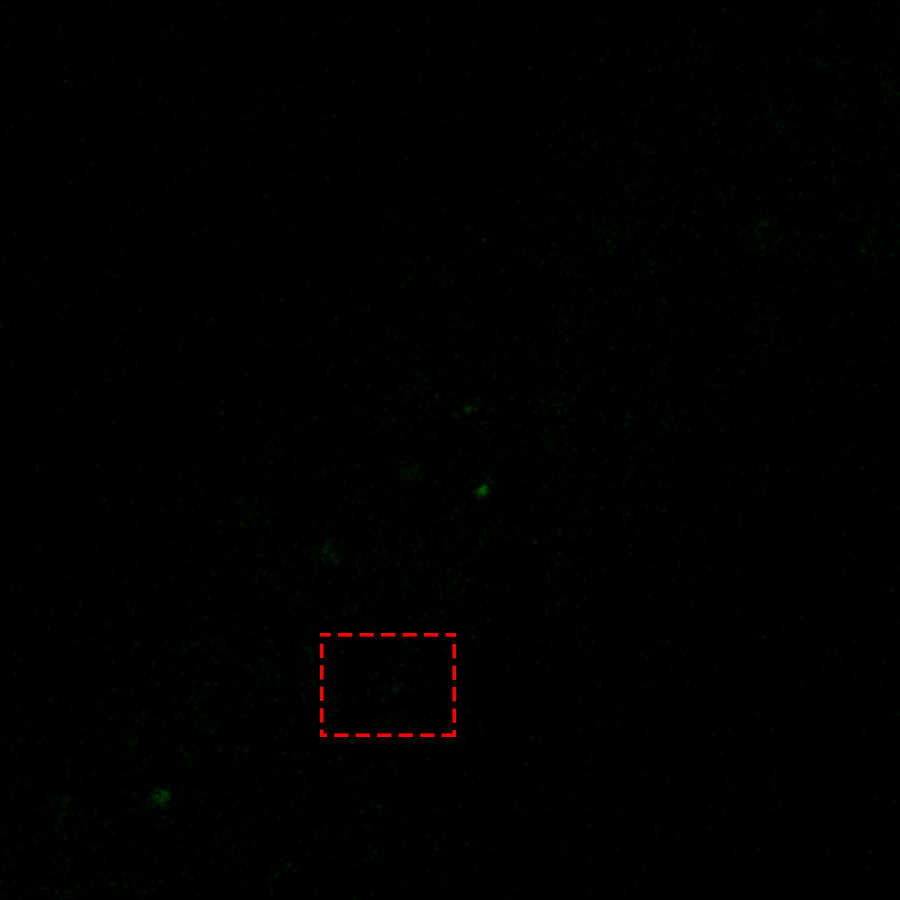

Supplement: Supplementary file 4 — Source data Fig. 1 [file 44319_2025_485_MOESM4_ESM.zip › Figure 1/1I/1I_sas6_gfp_FRAP/1I_sas6_gfp_0''.tif]

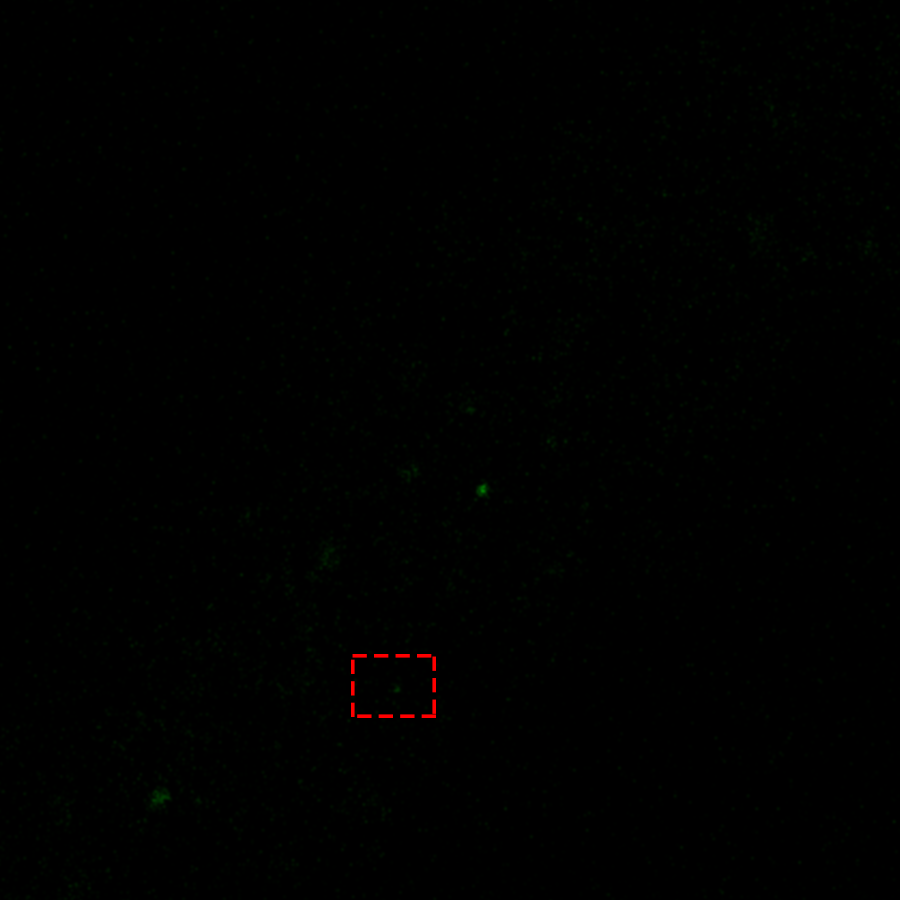

Supplement: Supplementary file 4 — Source data Fig. 1 [file 44319_2025_485_MOESM4_ESM.zip › Figure 1/1I/1I_sas6_gfp_FRAP/1I_sas6_gfp_5''.tif]

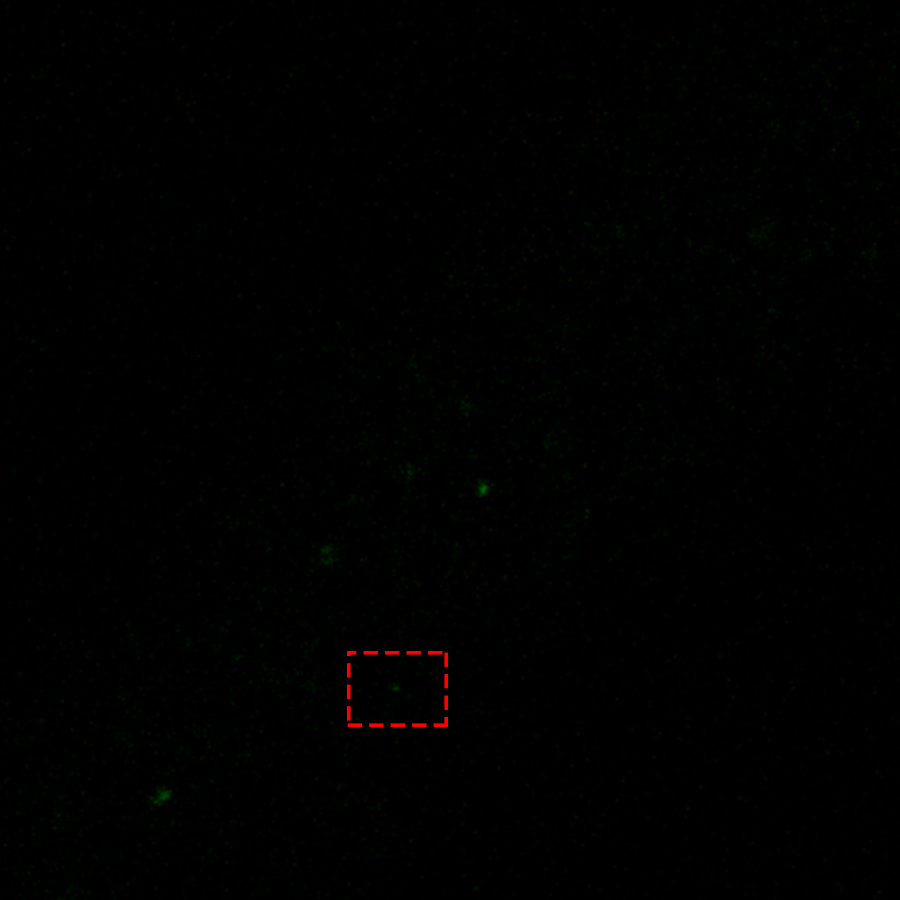

Supplement: Supplementary file 4 — Source data Fig. 1 [file 44319_2025_485_MOESM4_ESM.zip › Figure 1/1I/1I_sas6_gfp_FRAP/1I_sas6_gfp_20''.tif]

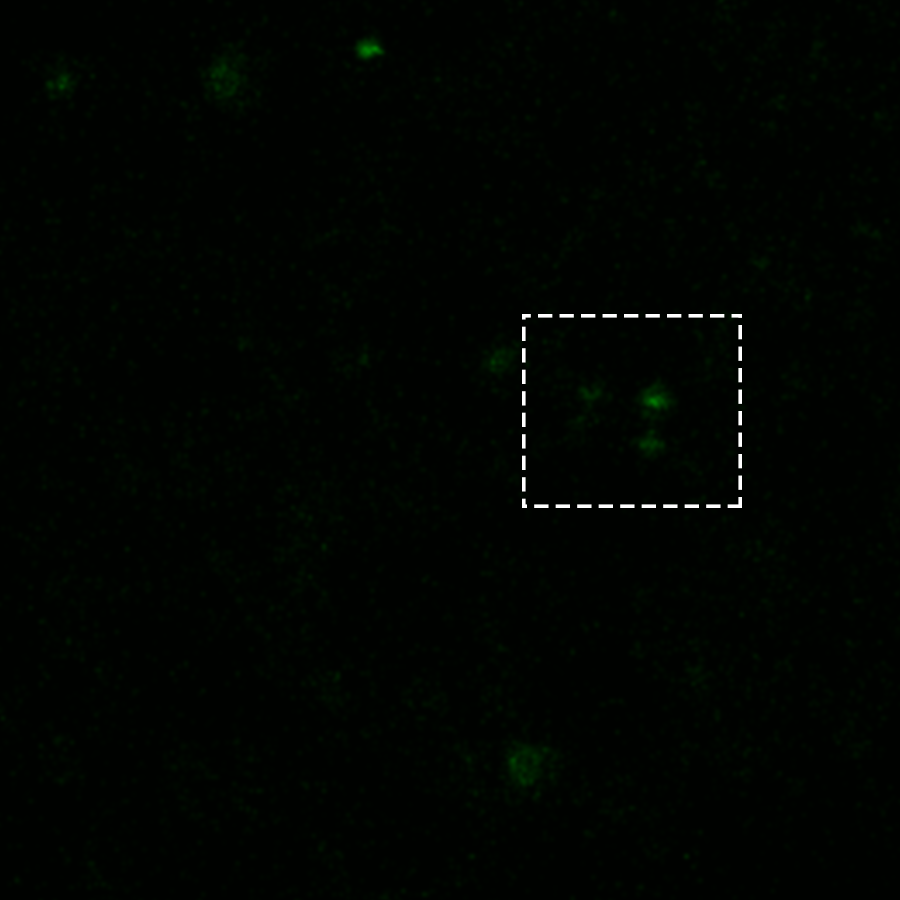

Supplement: Supplementary file 4 — Source data Fig. 1 [file 44319_2025_485_MOESM4_ESM.zip › Figure 1/1I/1I_gfp_sas_5_FRAP/1I_gfp_sas5_0''.tif]

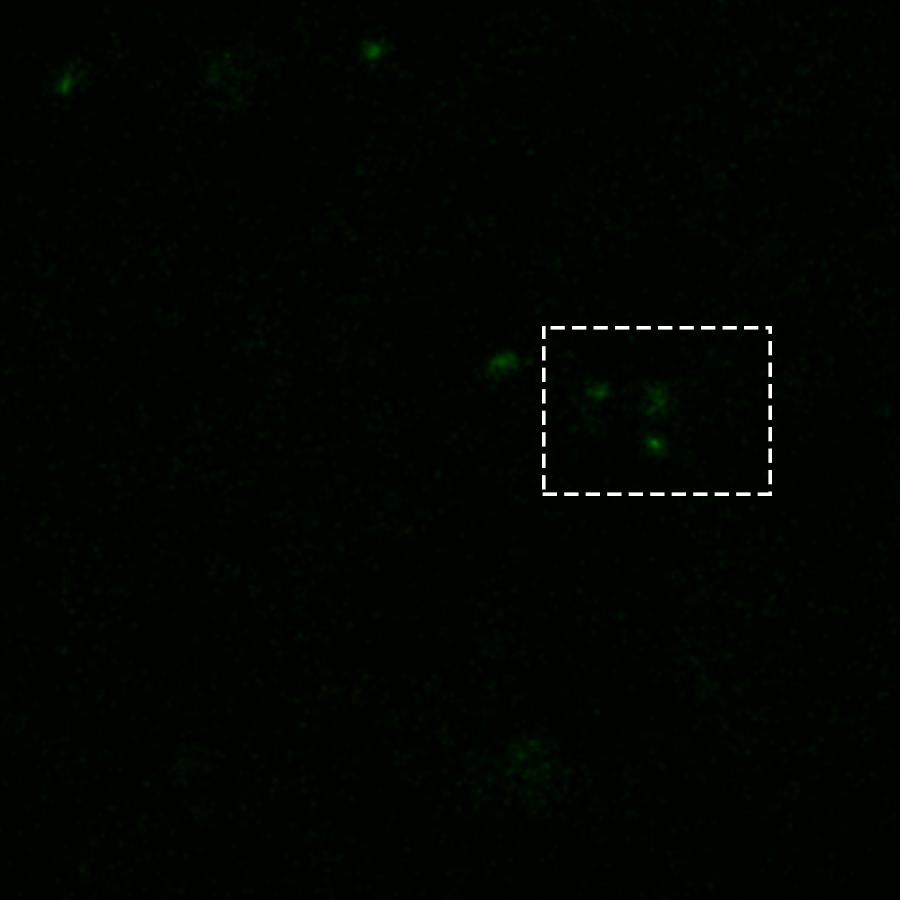

Supplement: Supplementary file 4 — Source data Fig. 1 [file 44319_2025_485_MOESM4_ESM.zip › Figure 1/1I/1I_gfp_sas_5_FRAP/1I_gfp_sas5_50''.tif]

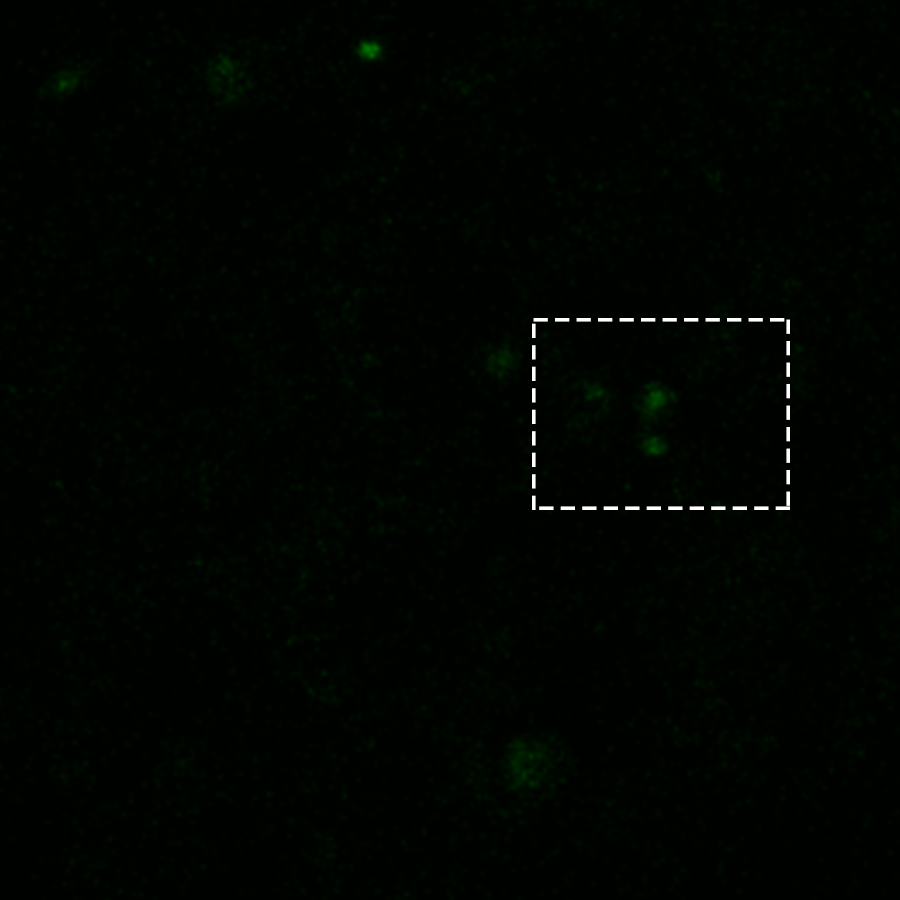

Supplement: Supplementary file 4 — Source data Fig. 1 [file 44319_2025_485_MOESM4_ESM.zip › Figure 1/1I/1I_gfp_sas_5_FRAP/1I_gfp_sas5_10''.tif]

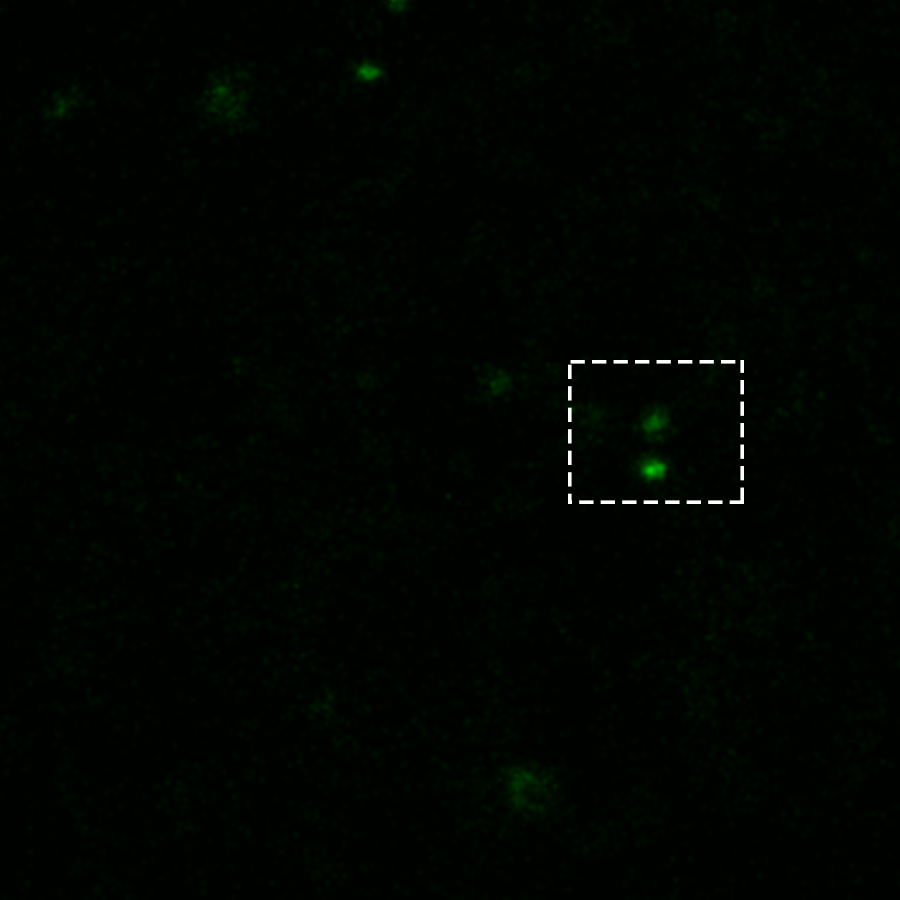

Supplement: Supplementary file 4 — Source data Fig. 1 [file 44319_2025_485_MOESM4_ESM.zip › Figure 1/1I/1I_gfp_sas_5_FRAP/1I_gfp_sas5_prebleach.tif]

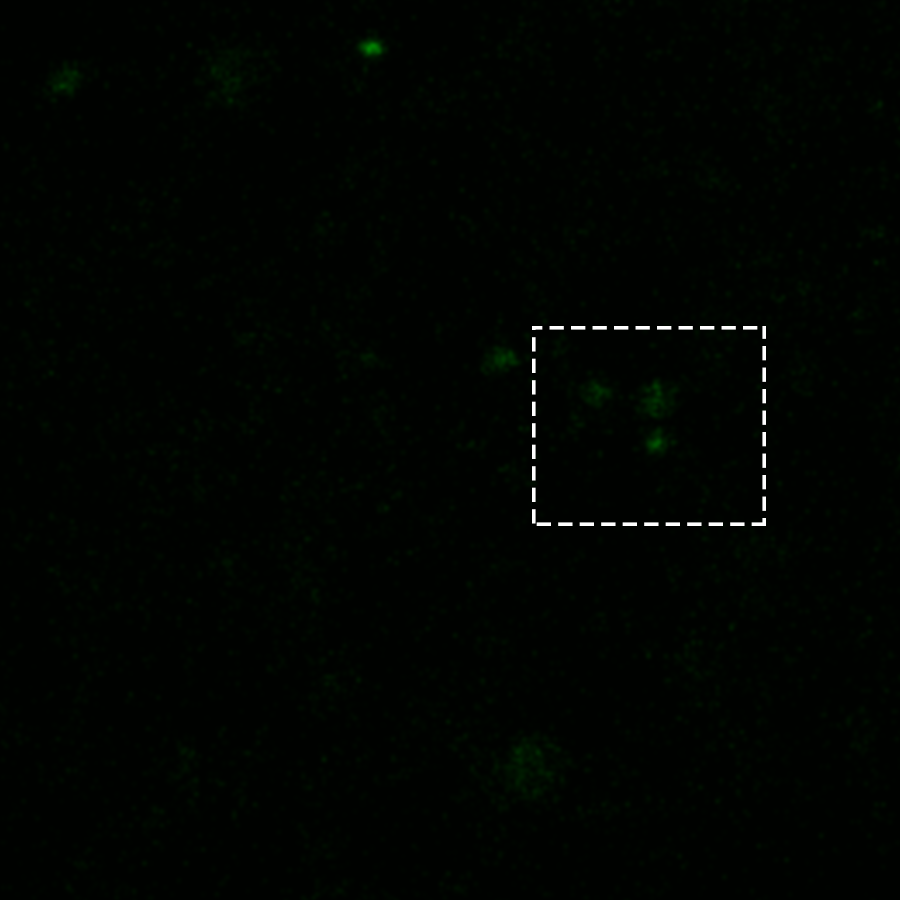

Supplement: Supplementary file 4 — Source data Fig. 1 [file 44319_2025_485_MOESM4_ESM.zip › Figure 1/1I/1I_gfp_sas_5_FRAP/1I_gfp_sas5_20''.tif]

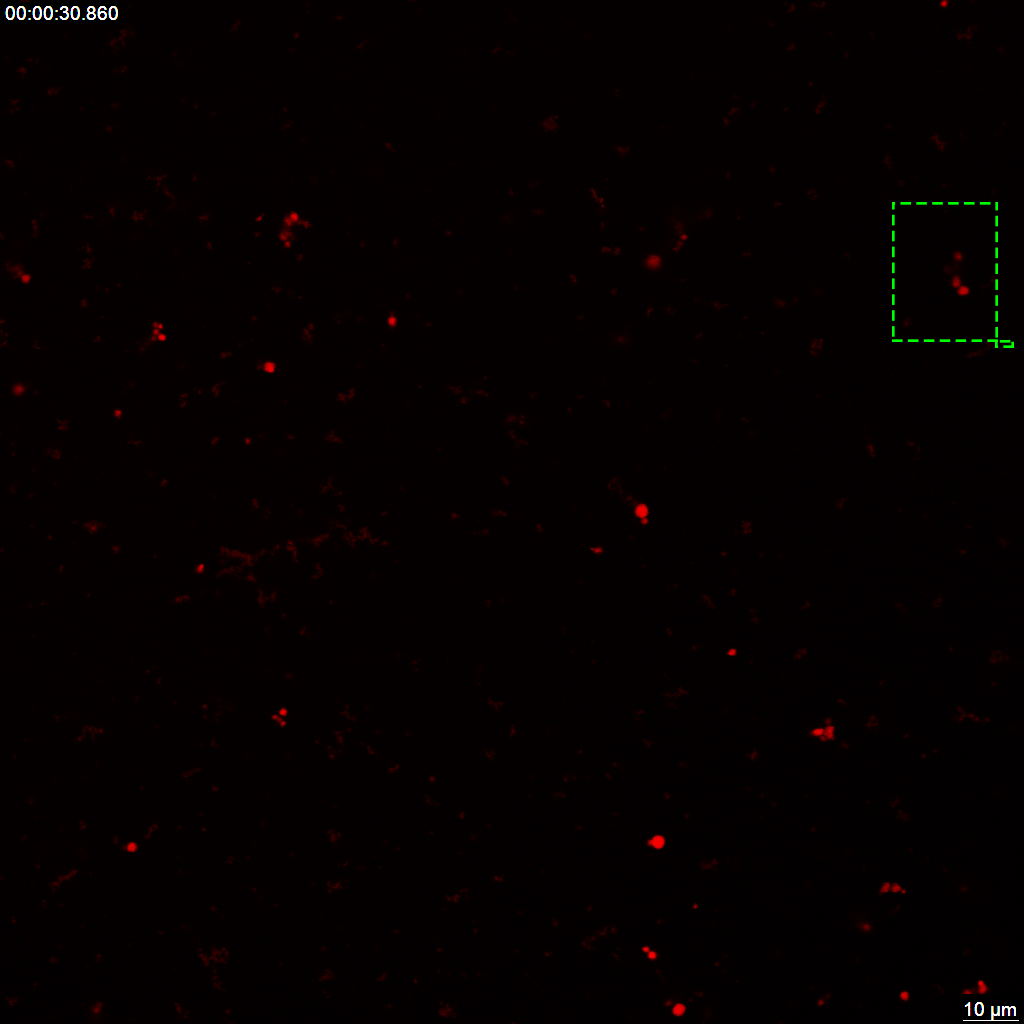

Supplement: Supplementary file 5 — Source data Fig. 2 [file 44319_2025_485_MOESM5_ESM.zip › Figure 2/2G/Fig. 2G_SAS_6_1,6Hex_30S.tif]

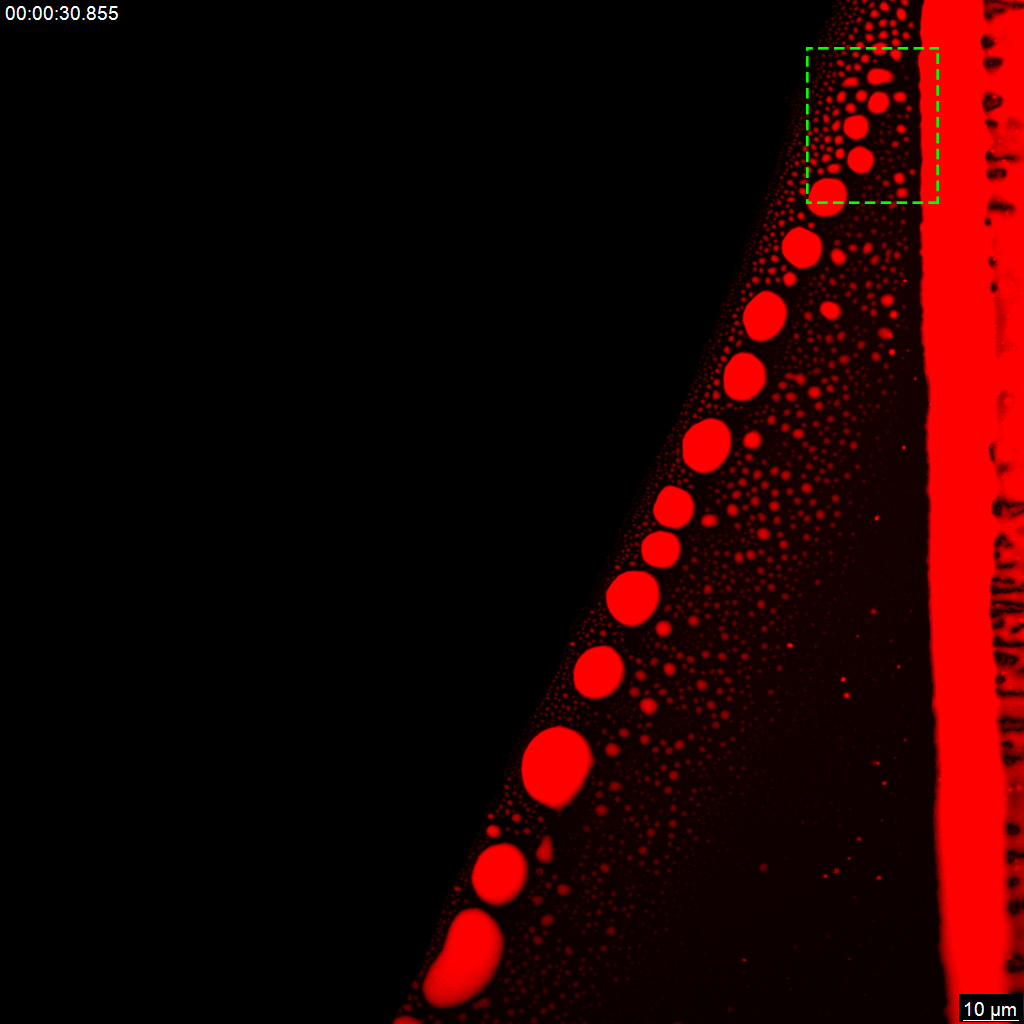

Supplement: Supplementary file 5 — Source data Fig. 2 [file 44319_2025_485_MOESM5_ESM.zip › Figure 2/2G/Fig. 2G_SAS_6_30S.tif]

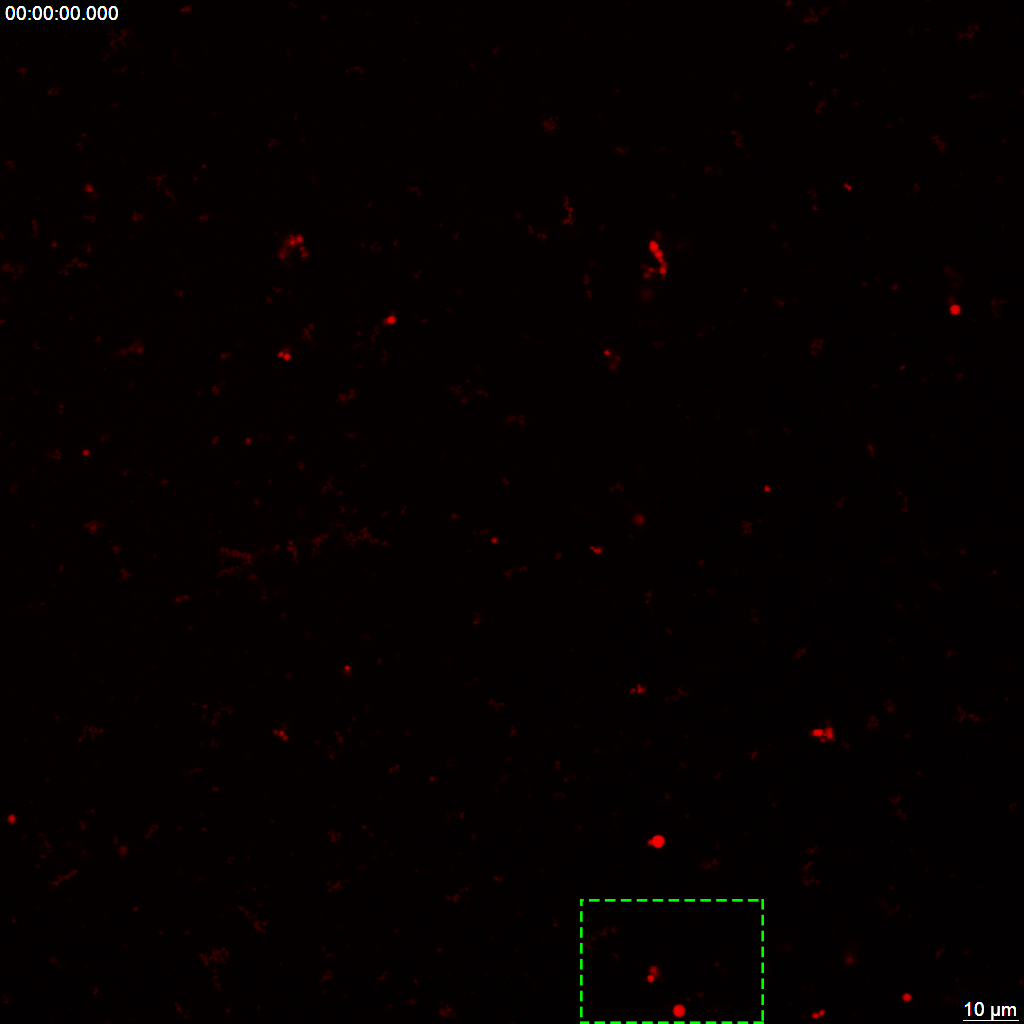

Supplement: Supplementary file 5 — Source data Fig. 2 [file 44319_2025_485_MOESM5_ESM.zip › Figure 2/2G/Fig. 2G_SAS_6_1,6Hex_0S.tif]

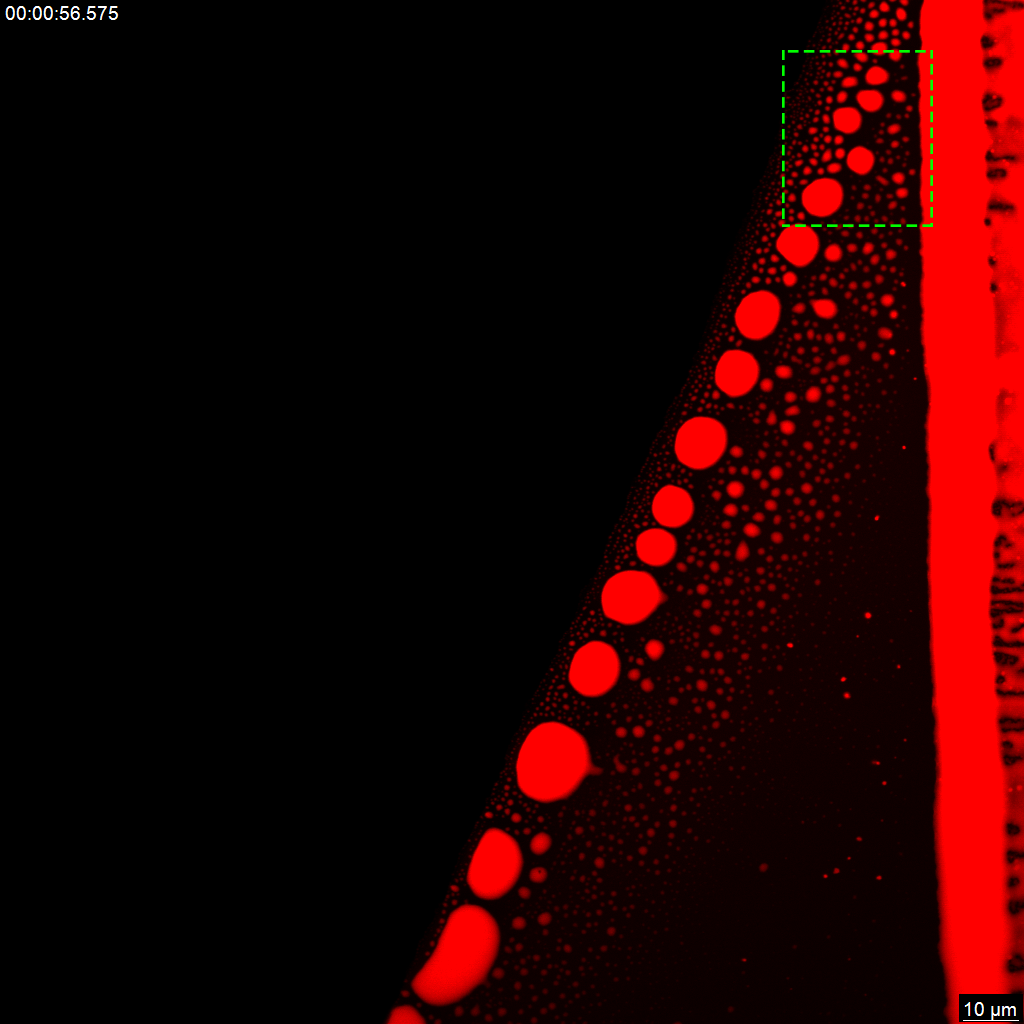

Supplement: Supplementary file 5 — Source data Fig. 2 [file 44319_2025_485_MOESM5_ESM.zip › Figure 2/2G/Fig. 2G_SAS_6_60S.tif]

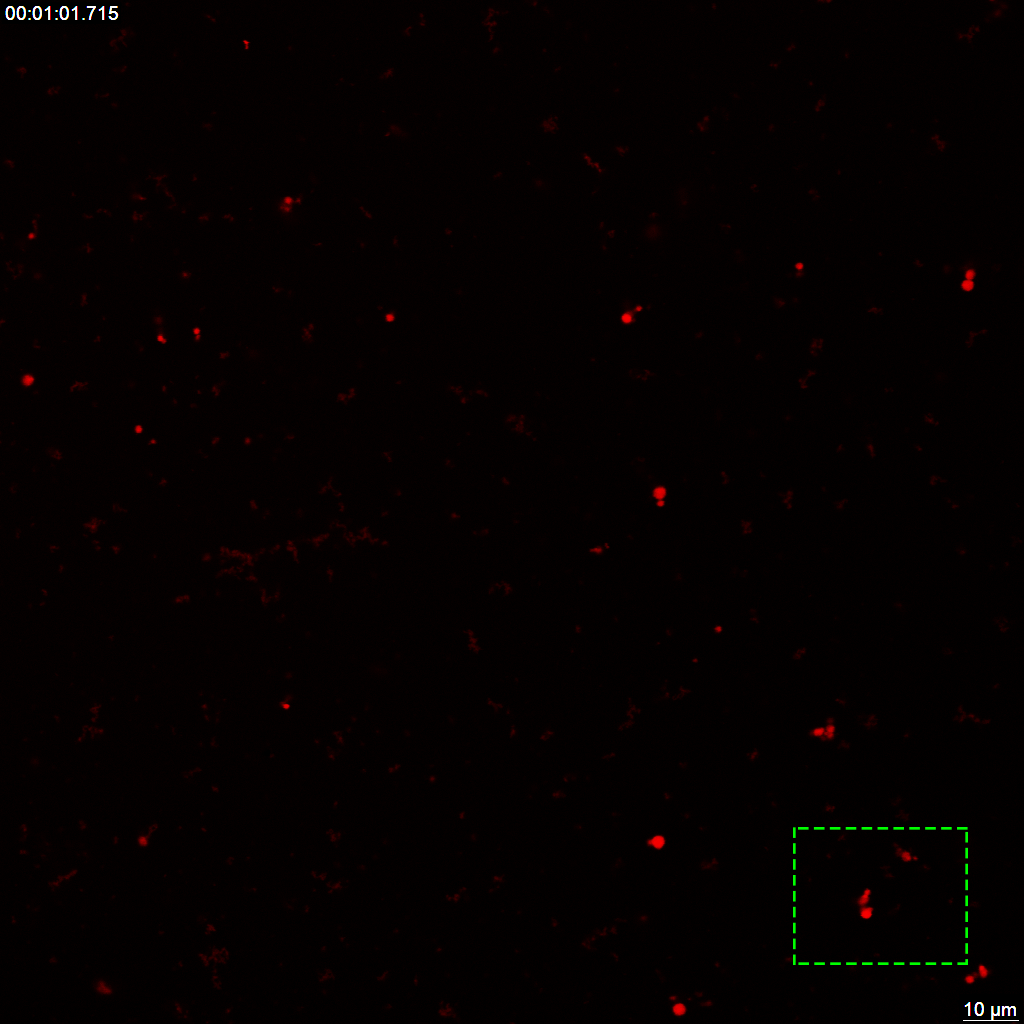

Supplement: Supplementary file 5 — Source data Fig. 2 [file 44319_2025_485_MOESM5_ESM.zip › Figure 2/2G/Fig. 2G_SAS_6_1,6Hex_60S.tif]

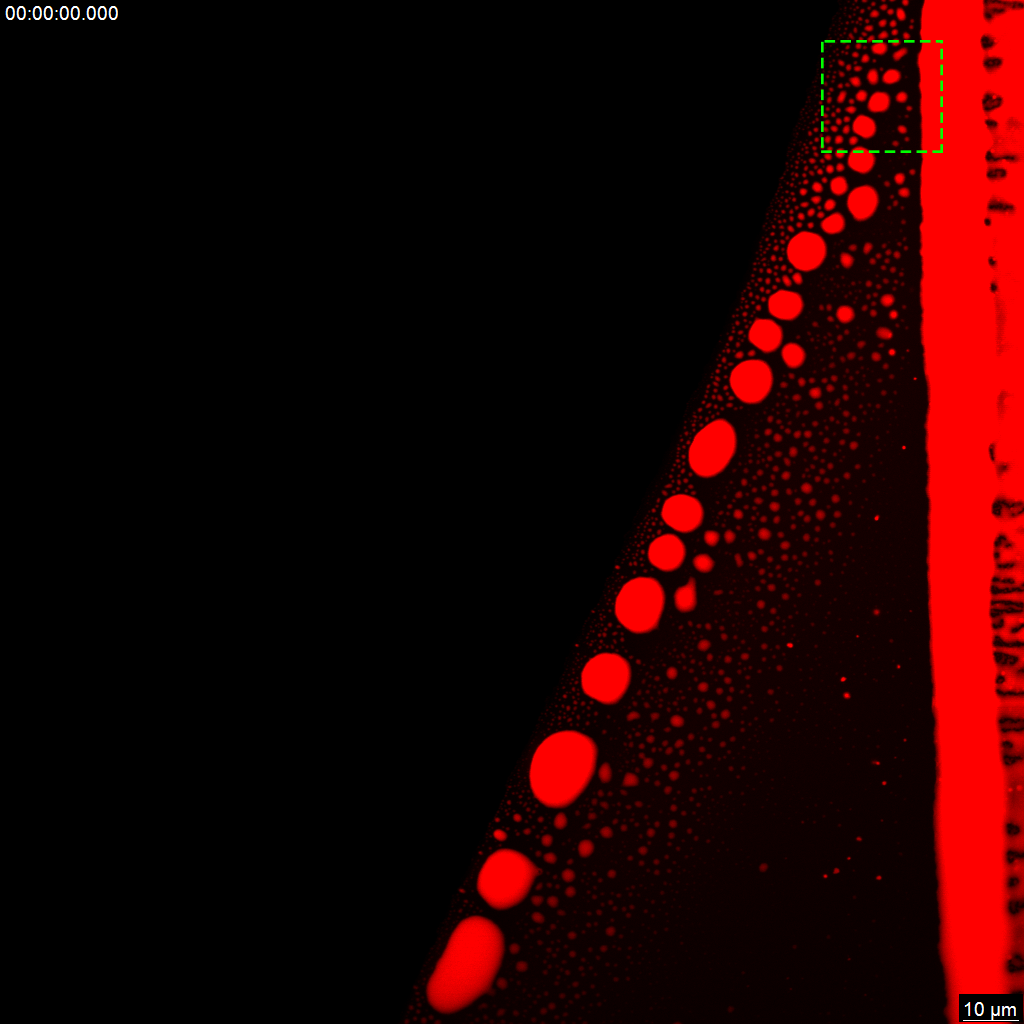

Supplement: Supplementary file 5 — Source data Fig. 2 [file 44319_2025_485_MOESM5_ESM.zip › Figure 2/2G/Fig. 2G_SAS_6_0S.tif]

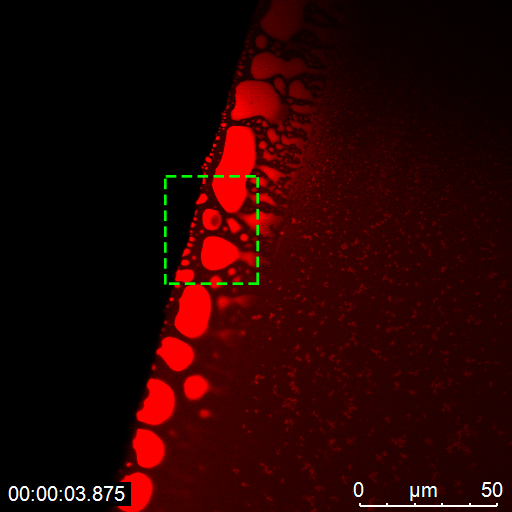

Supplement: Supplementary file 5 — Source data Fig. 2 [file 44319_2025_485_MOESM5_ESM.zip › Figure 2/2H/Fig. 2H_SAS_6_mCherry_FRAP_3S.tif]

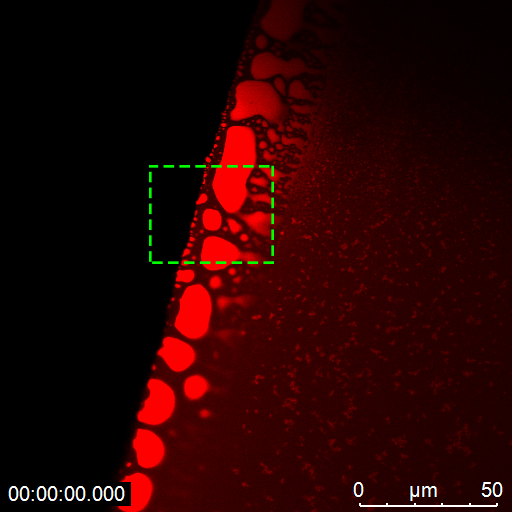

Supplement: Supplementary file 5 — Source data Fig. 2 [file 44319_2025_485_MOESM5_ESM.zip › Figure 2/2H/Fig. 2H_SAS_6_mCherry_FRAP_minus2S.tif]

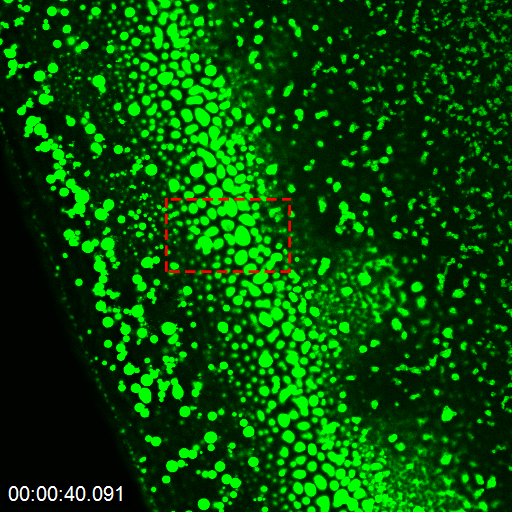

Supplement: Supplementary file 5 — Source data Fig. 2 [file 44319_2025_485_MOESM5_ESM.zip › Figure 2/2H/Fig. 2H_SAS_6_GFP_FRAP_40S.tif]

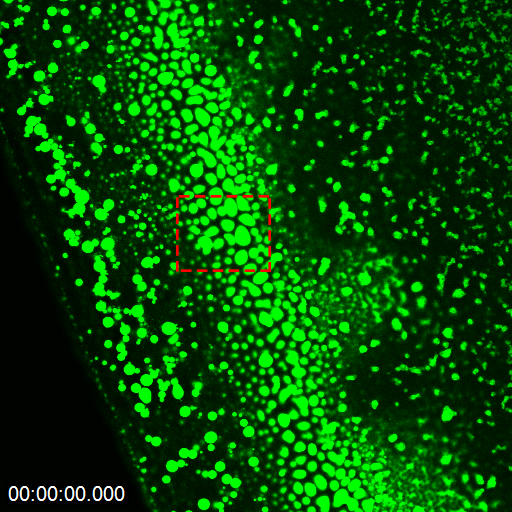

Supplement: Supplementary file 5 — Source data Fig. 2 [file 44319_2025_485_MOESM5_ESM.zip › Figure 2/2H/Fig. 2H_SAS_6_GFP_FRAP_minus2S.tif]

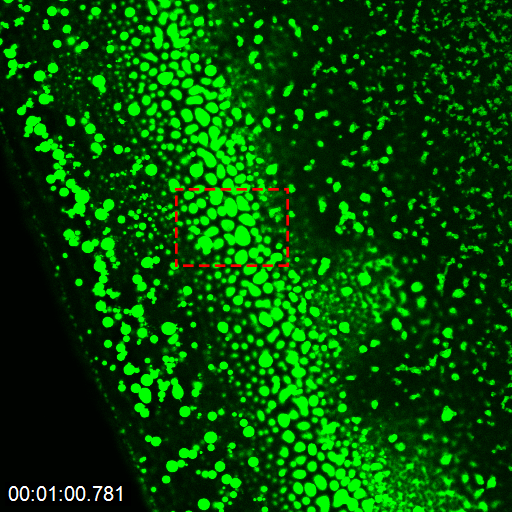

Supplement: Supplementary file 5 — Source data Fig. 2 [file 44319_2025_485_MOESM5_ESM.zip › Figure 2/2H/Fig. 2H_SAS_6_GFP_FRAP_60S.tif]

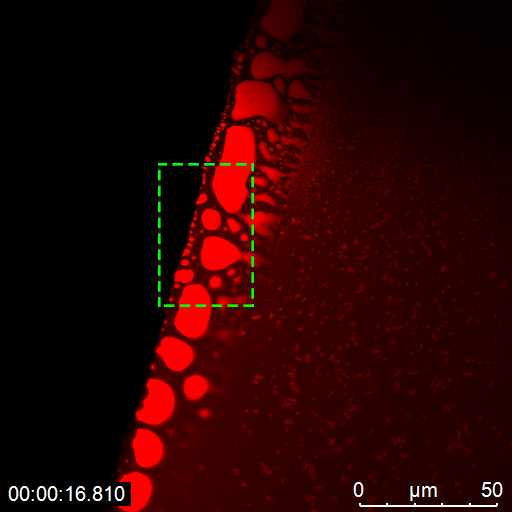

Supplement: Supplementary file 5 — Source data Fig. 2 [file 44319_2025_485_MOESM5_ESM.zip › Figure 2/2H/Fig. 2H_SAS_6_mCherry_FRAP_16S.tif]

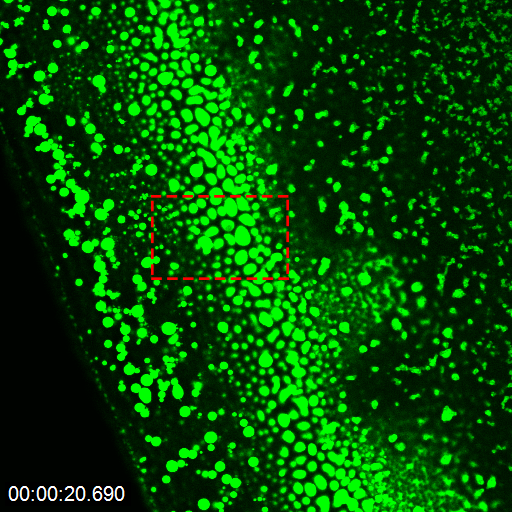

Supplement: Supplementary file 5 — Source data Fig. 2 [file 44319_2025_485_MOESM5_ESM.zip › Figure 2/2H/Fig. 2H_SAS_6_GFP_FRAP_20S.tif]

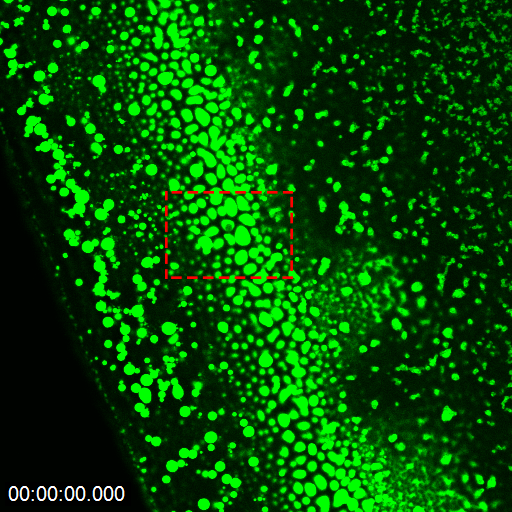

Supplement: Supplementary file 5 — Source data Fig. 2 [file 44319_2025_485_MOESM5_ESM.zip › Figure 2/2H/Fig. 2H_SAS_6_GFP_FRAP_0S.tif]

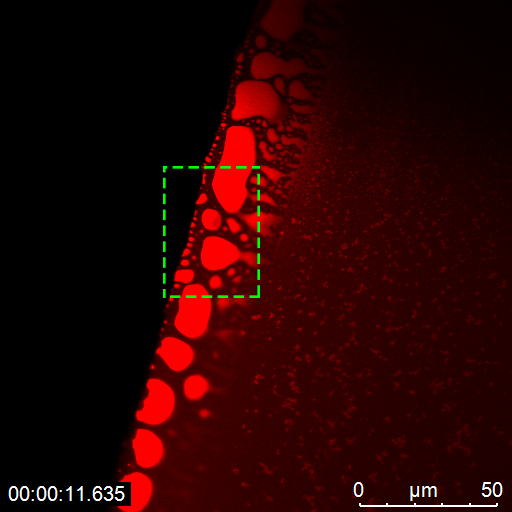

Supplement: Supplementary file 5 — Source data Fig. 2 [file 44319_2025_485_MOESM5_ESM.zip › Figure 2/2H/Fig. 2H_SAS_6_mCherry_FRAP_11S.tif]

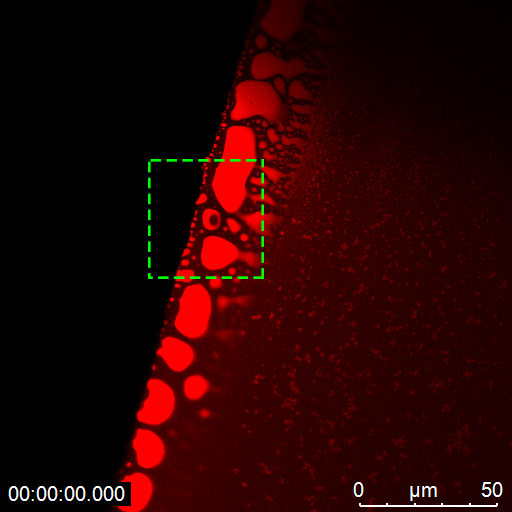

Supplement: Supplementary file 5 — Source data Fig. 2 [file 44319_2025_485_MOESM5_ESM.zip › Figure 2/2H/Fig. 2H_SAS_6_mCherry_FRAP_0S.tif]

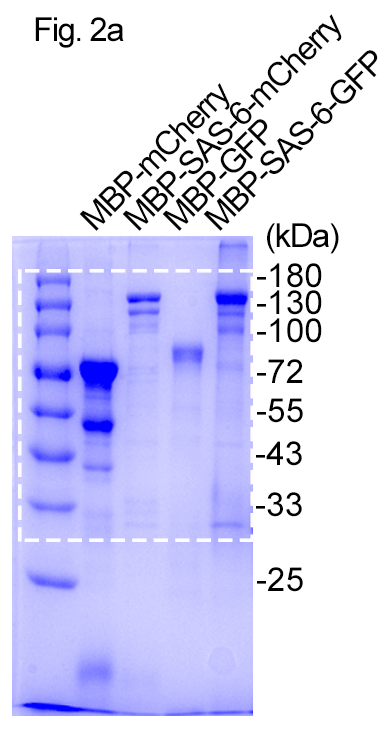

Supplement: Supplementary file 5 — Source data Fig. 2 [file 44319_2025_485_MOESM5_ESM.zip › Figure 2/2A/Fig. 2A.tif]

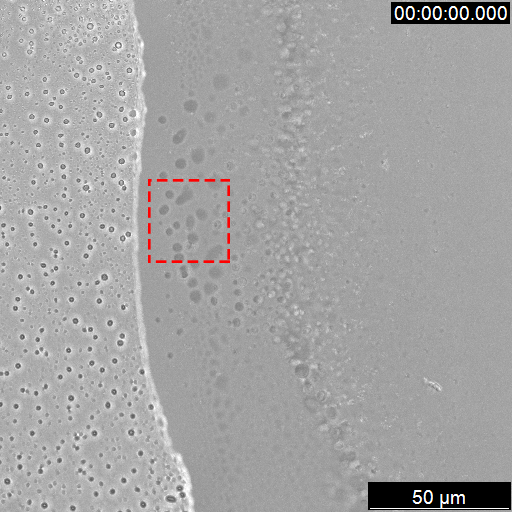

Supplement: Supplementary file 5 — Source data Fig. 2 [file 44319_2025_485_MOESM5_ESM.zip › Figure 2/2F/Fig. 2F_SAS_6_GFP_0S_BF.tif]

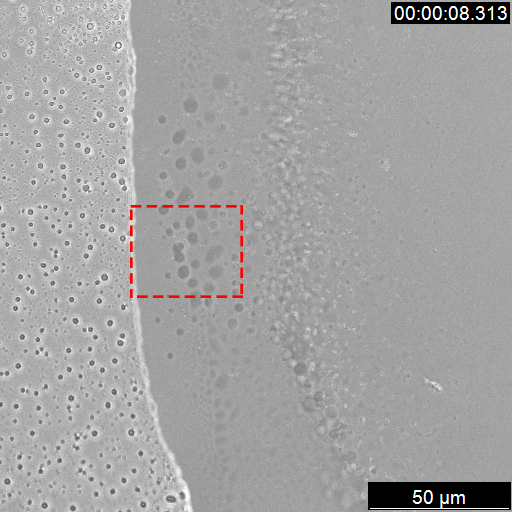

Supplement: Supplementary file 5 — Source data Fig. 2 [file 44319_2025_485_MOESM5_ESM.zip › Figure 2/2F/Fig. 2F_SAS_6_GFP_8S_BF.tif]

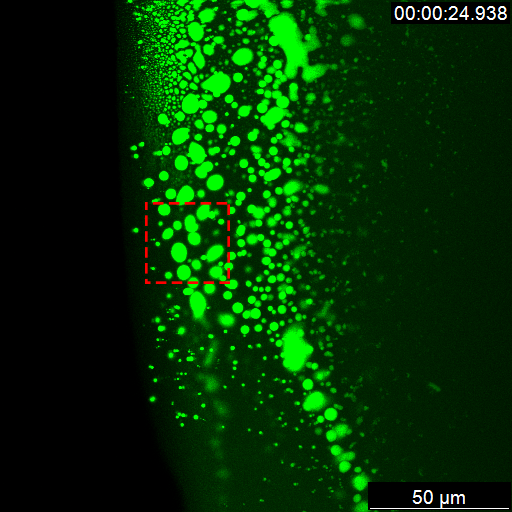

Supplement: Supplementary file 5 — Source data Fig. 2 [file 44319_2025_485_MOESM5_ESM.zip › Figure 2/2F/Fig. 2F_SAS_6_GFP_24S.tif]

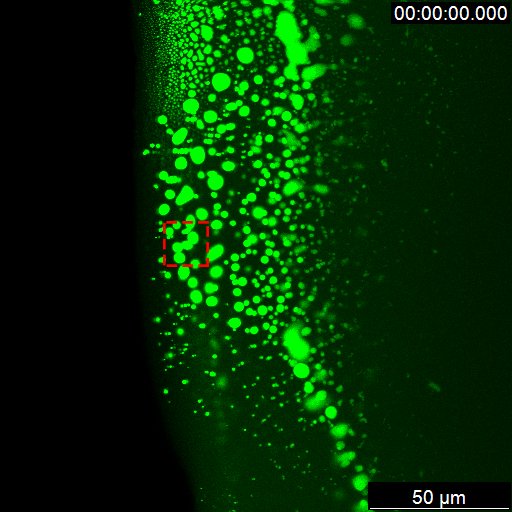

Supplement: Supplementary file 5 — Source data Fig. 2 [file 44319_2025_485_MOESM5_ESM.zip › Figure 2/2F/Fig. 2F_SAS_6_GFP_0S.tif]

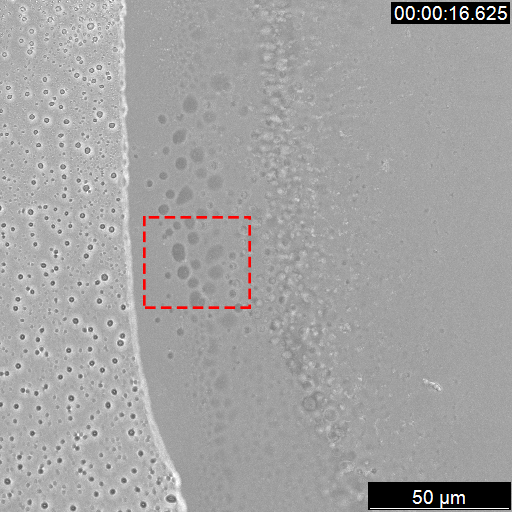

Supplement: Supplementary file 5 — Source data Fig. 2 [file 44319_2025_485_MOESM5_ESM.zip › Figure 2/2F/Fig. 2F_SAS_6_GFP_16S_BF.tif]

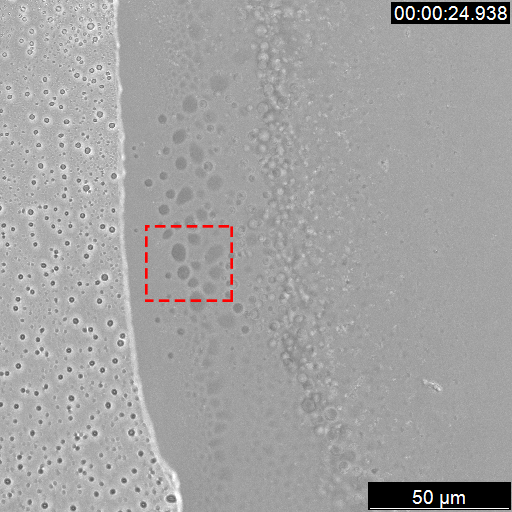

Supplement: Supplementary file 5 — Source data Fig. 2 [file 44319_2025_485_MOESM5_ESM.zip › Figure 2/2F/Fig. 2F_SAS_6_GFP_24S_BF.tif]

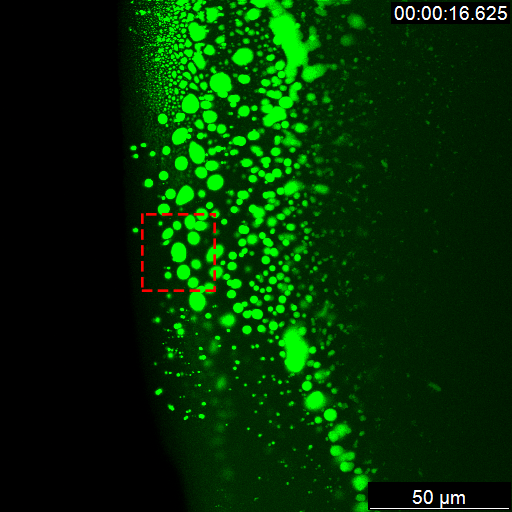

Supplement: Supplementary file 5 — Source data Fig. 2 [file 44319_2025_485_MOESM5_ESM.zip › Figure 2/2F/Fig. 2F_SAS_6_GFP_16S.tif]

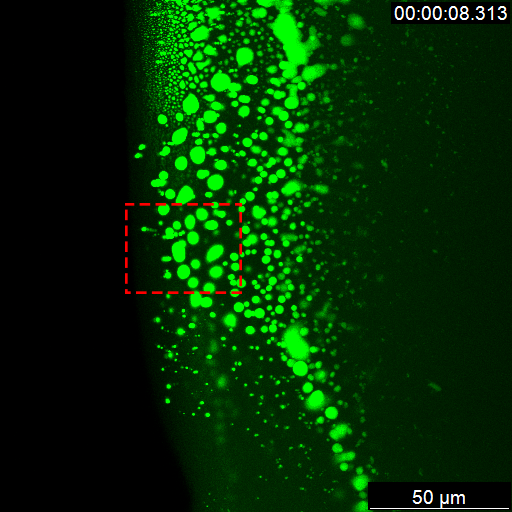

Supplement: Supplementary file 5 — Source data Fig. 2 [file 44319_2025_485_MOESM5_ESM.zip › Figure 2/2F/Fig. 2F_SAS_6_GFP_8S.tif]

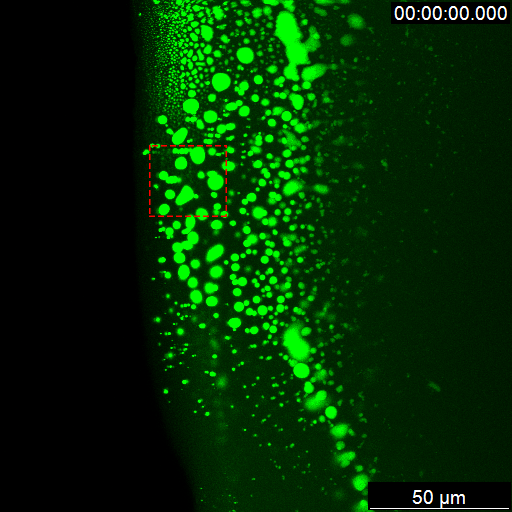

Supplement: Supplementary file 5 — Source data Fig. 2 [file 44319_2025_485_MOESM5_ESM.zip › Figure 2/2C/Fig. 2C_SAS_6_GFP_0min.tif]

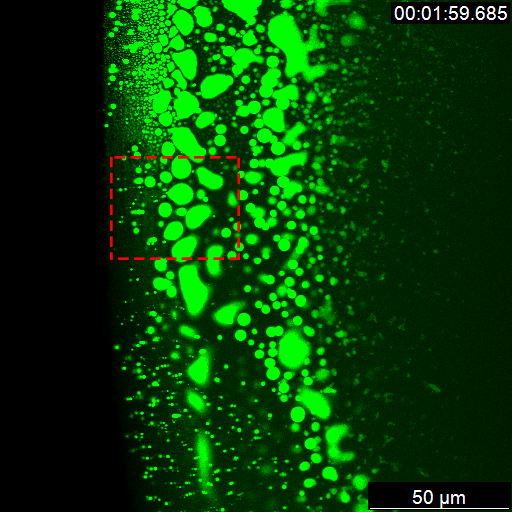

Supplement: Supplementary file 5 — Source data Fig. 2 [file 44319_2025_485_MOESM5_ESM.zip › Figure 2/2C/Fig. 2C_SAS_6_GFP_2min.tif]

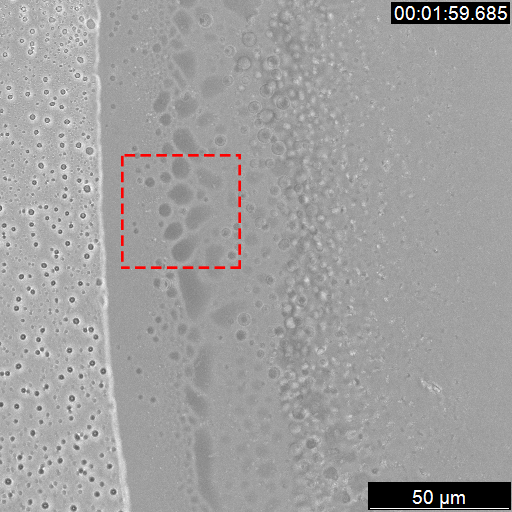

Supplement: Supplementary file 5 — Source data Fig. 2 [file 44319_2025_485_MOESM5_ESM.zip › Figure 2/2C/Fig. 2C_SAS_6_GFP_2min_BF.tif]

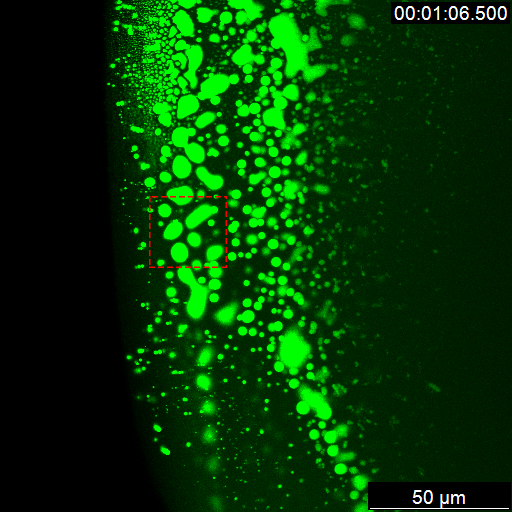

Supplement: Supplementary file 5 — Source data Fig. 2 [file 44319_2025_485_MOESM5_ESM.zip › Figure 2/2C/Fig. 2C_SAS_6_GFP_1min.tif]

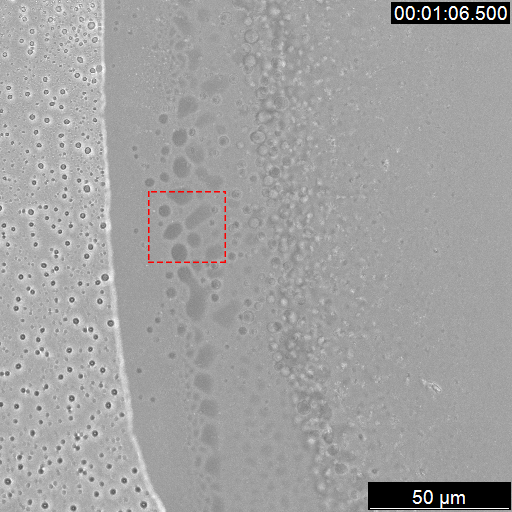

Supplement: Supplementary file 5 — Source data Fig. 2 [file 44319_2025_485_MOESM5_ESM.zip › Figure 2/2C/Fig. 2C_SAS_6_GFP_1min_BF.tif]

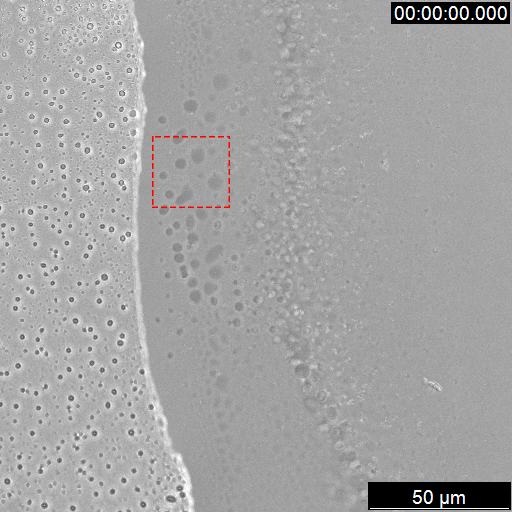

Supplement: Supplementary file 5 — Source data Fig. 2 [file 44319_2025_485_MOESM5_ESM.zip › Figure 2/2C/Fig. 2C_SAS_6_GFP_0min_BF.tif]

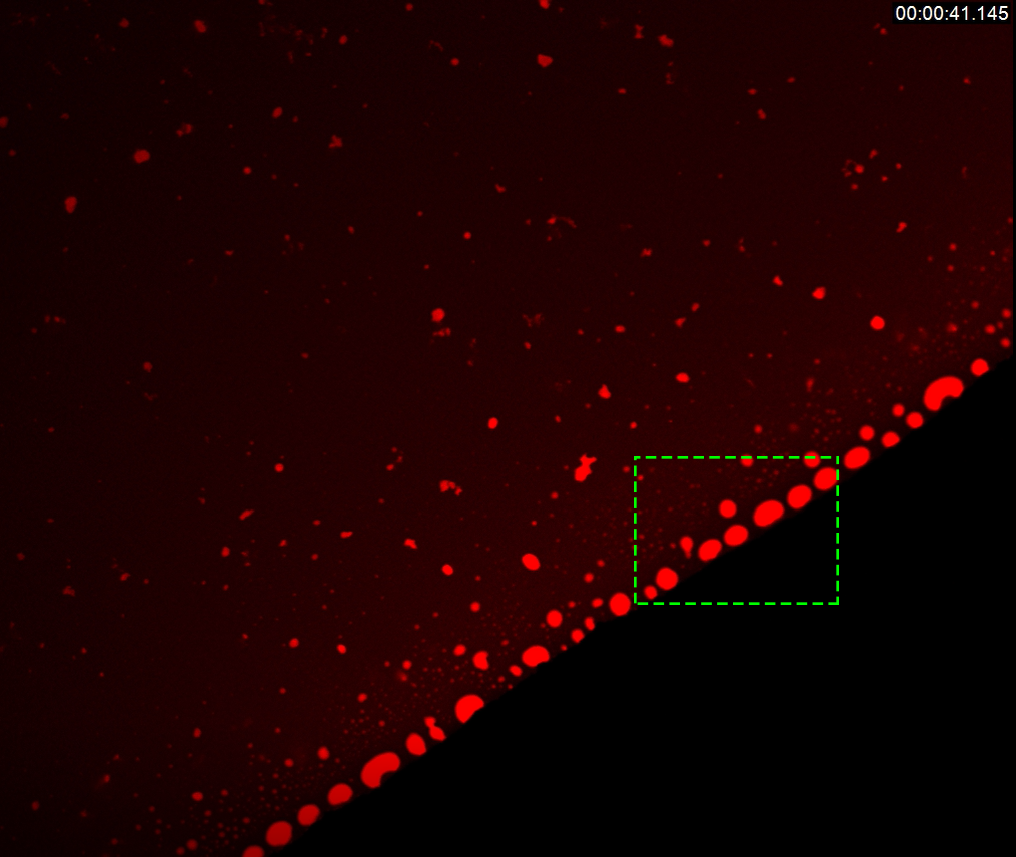

Supplement: Supplementary file 5 — Source data Fig. 2 [file 44319_2025_485_MOESM5_ESM.zip › Figure 2/2M/Fig. 2M_SAS_6_C_mCherry.tif]

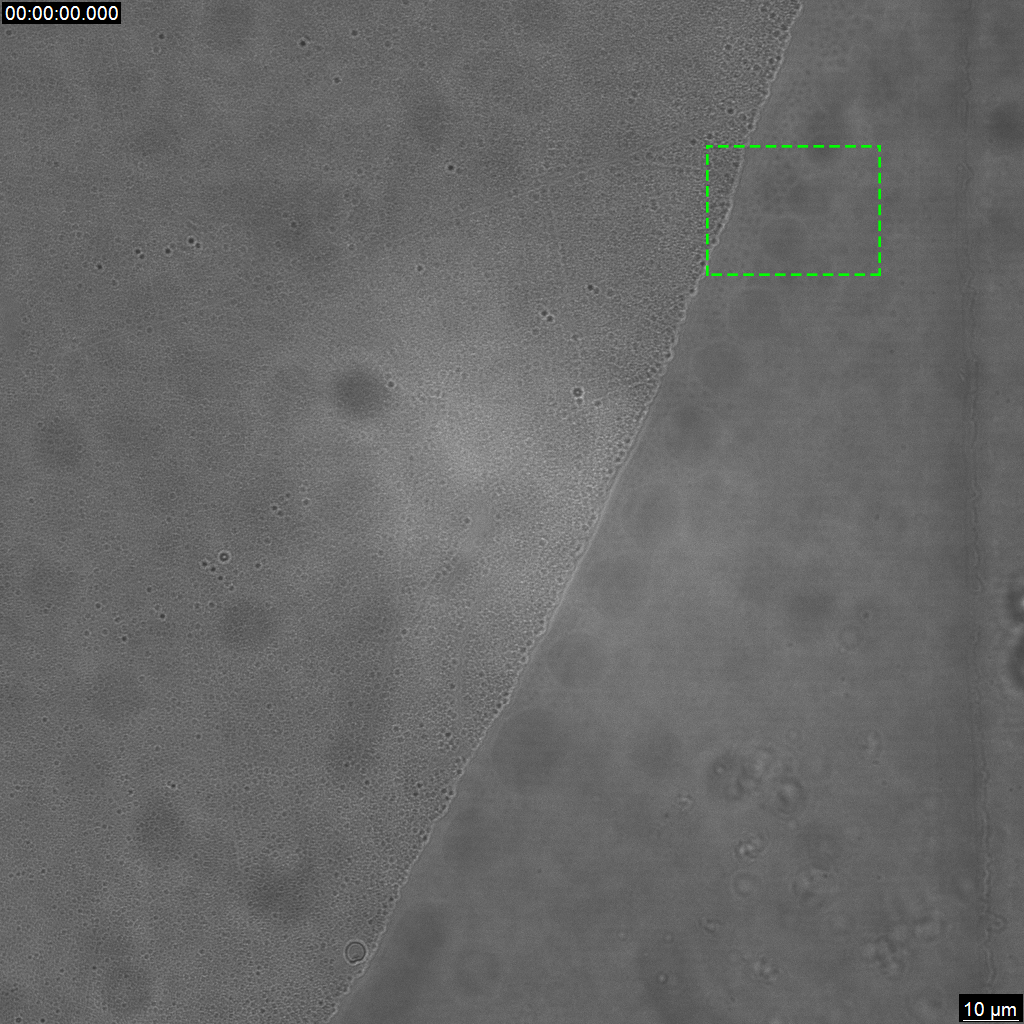

Supplement: Supplementary file 5 — Source data Fig. 2 [file 44319_2025_485_MOESM5_ESM.zip › Figure 2/2M/Fig. 2M_SAS_6_FL_mCherry_BF.tif]

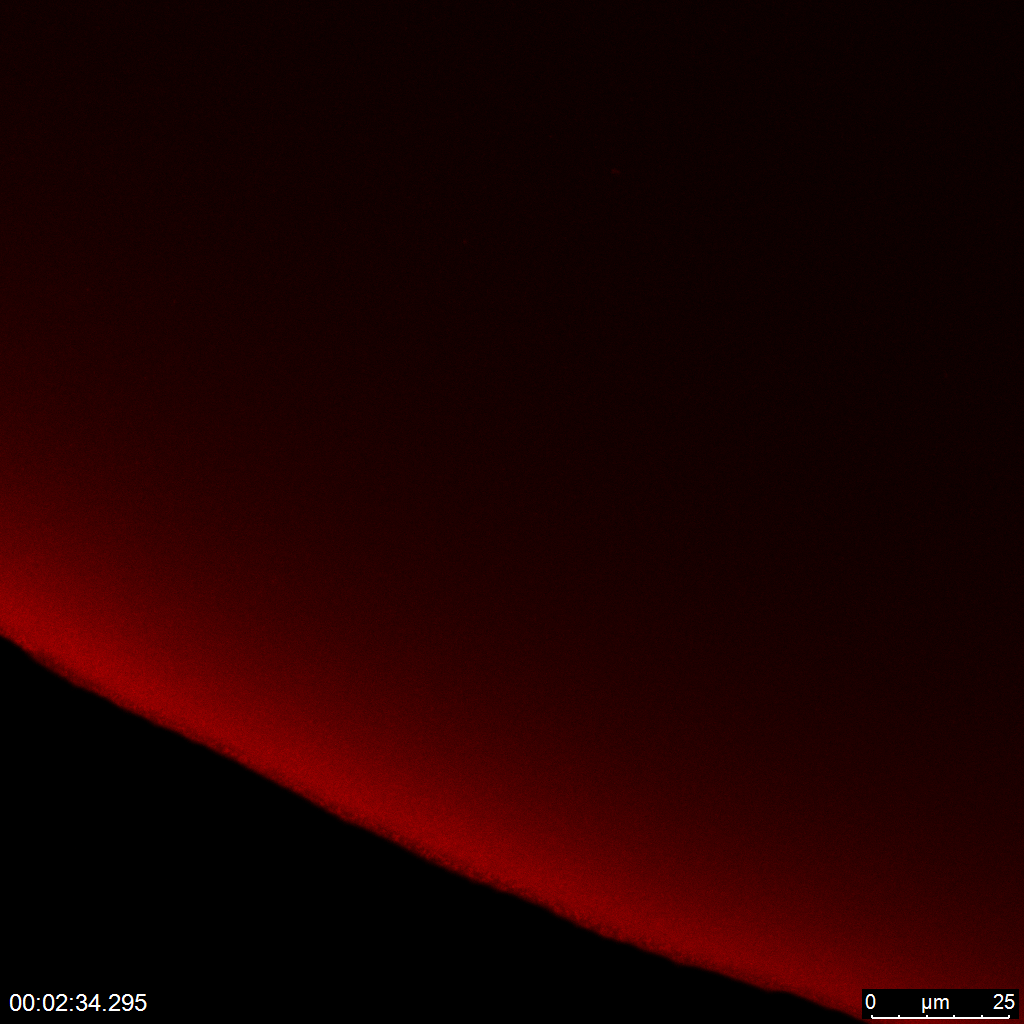

Supplement: Supplementary file 5 — Source data Fig. 2 [file 44319_2025_485_MOESM5_ESM.zip › Figure 2/2M/Fig. 2M_SAS_6_N_mCherry.tif]

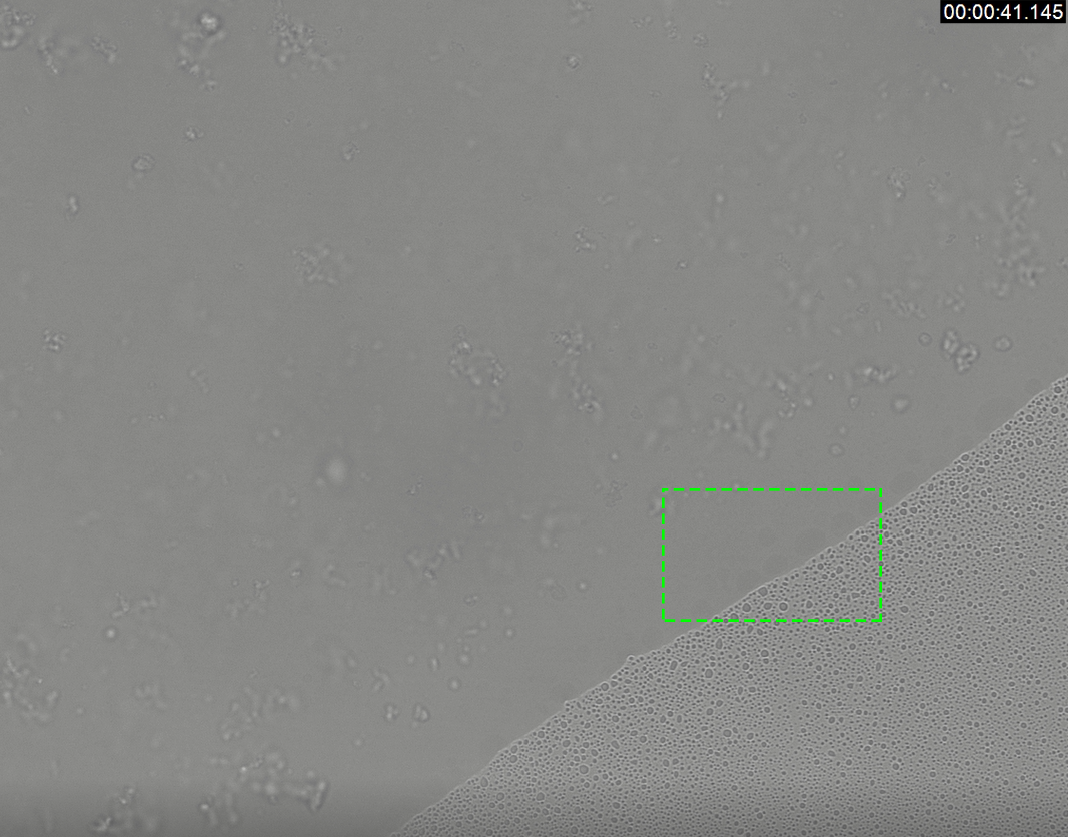

Supplement: Supplementary file 5 — Source data Fig. 2 [file 44319_2025_485_MOESM5_ESM.zip › Figure 2/2M/Fig. 2M_SAS_6_C_mCherry_BF.tif]

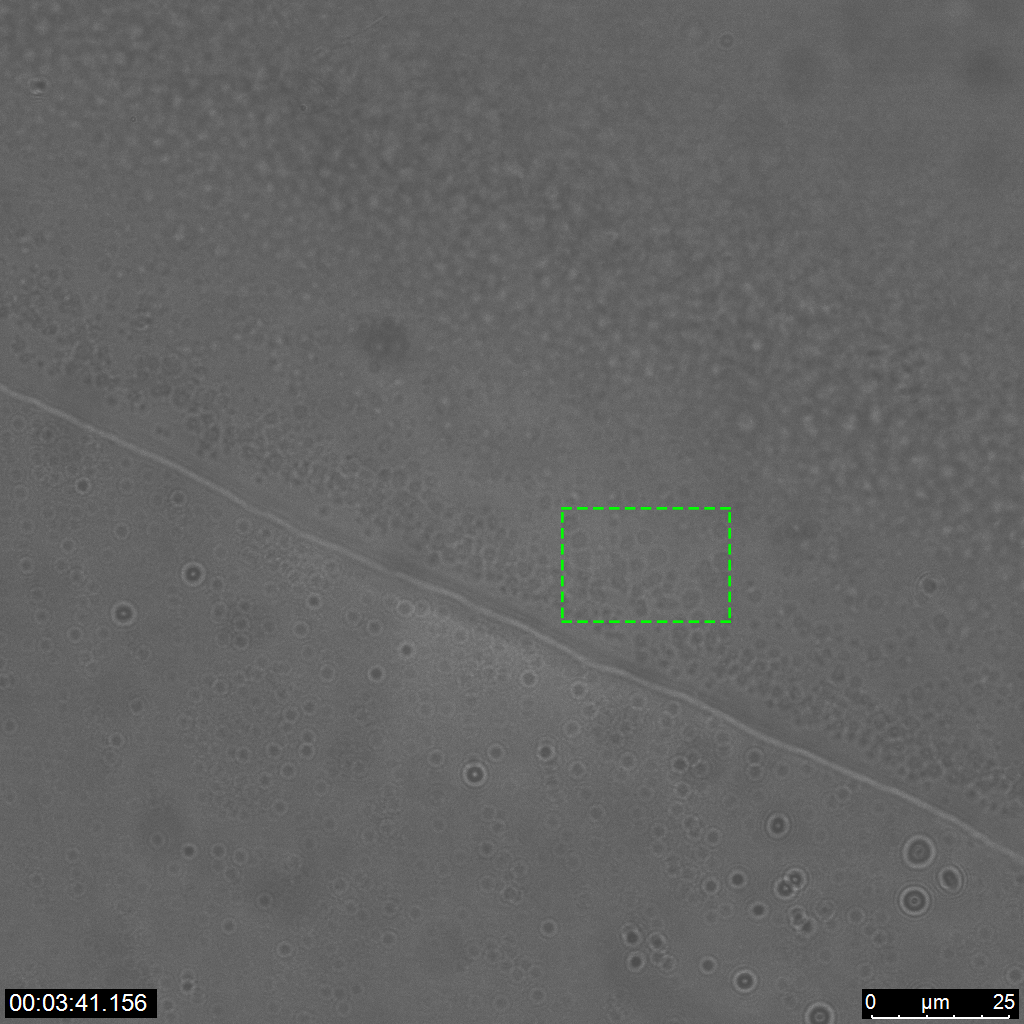

Supplement: Supplementary file 5 — Source data Fig. 2 [file 44319_2025_485_MOESM5_ESM.zip › Figure 2/2M/Fig. 2M_SAS_6_M_mCherry_BF.tif]

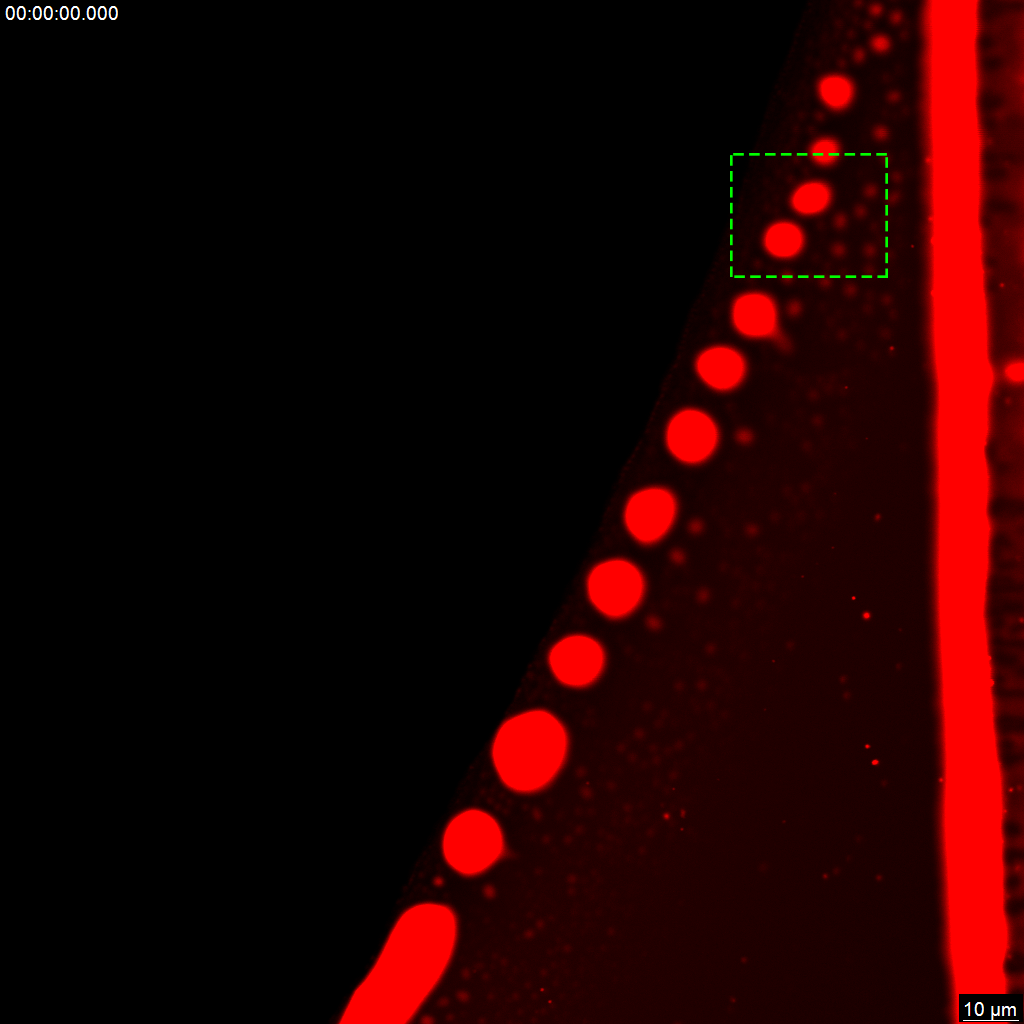

Supplement: Supplementary file 5 — Source data Fig. 2 [file 44319_2025_485_MOESM5_ESM.zip › Figure 2/2M/Fig. 2M_SAS_6_FL_mCherry.tif]

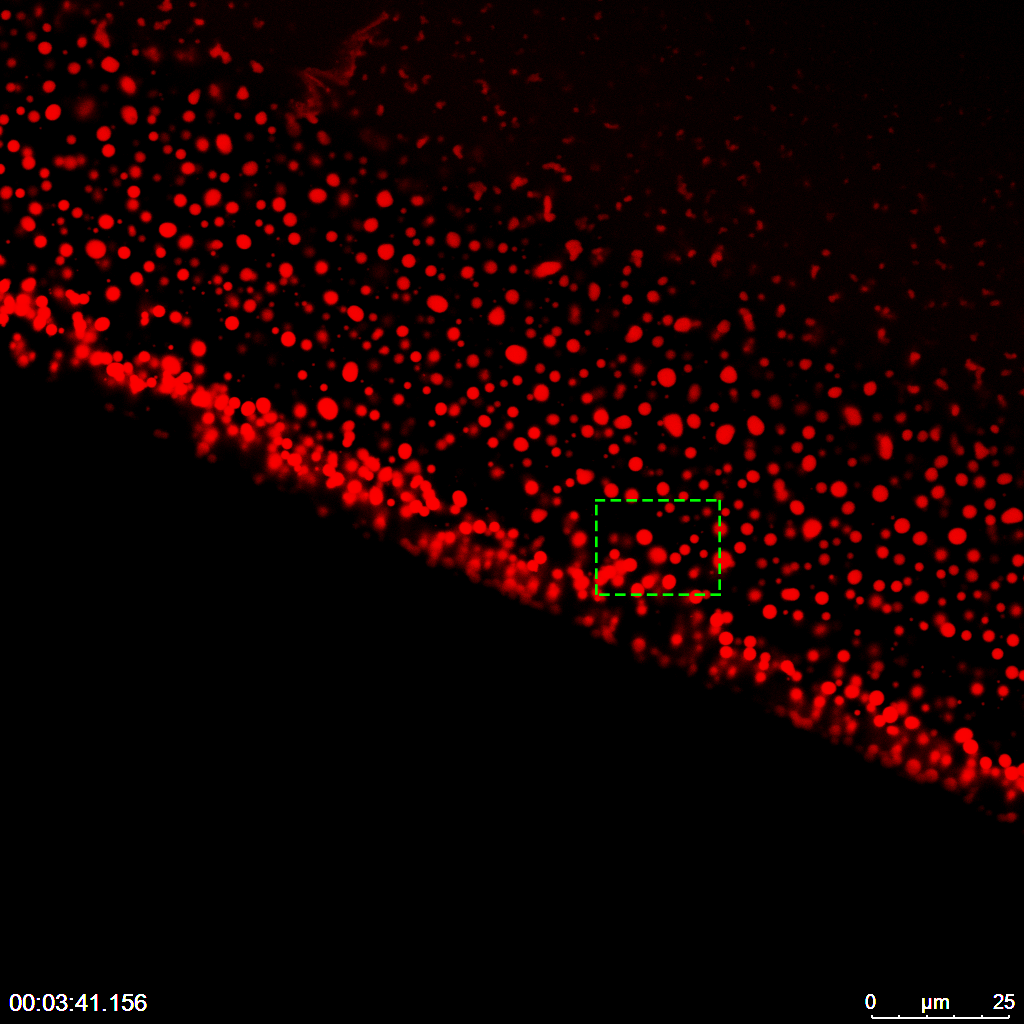

Supplement: Supplementary file 5 — Source data Fig. 2 [file 44319_2025_485_MOESM5_ESM.zip › Figure 2/2M/Fig. 2M_SAS_6_M_mCherry.tif]

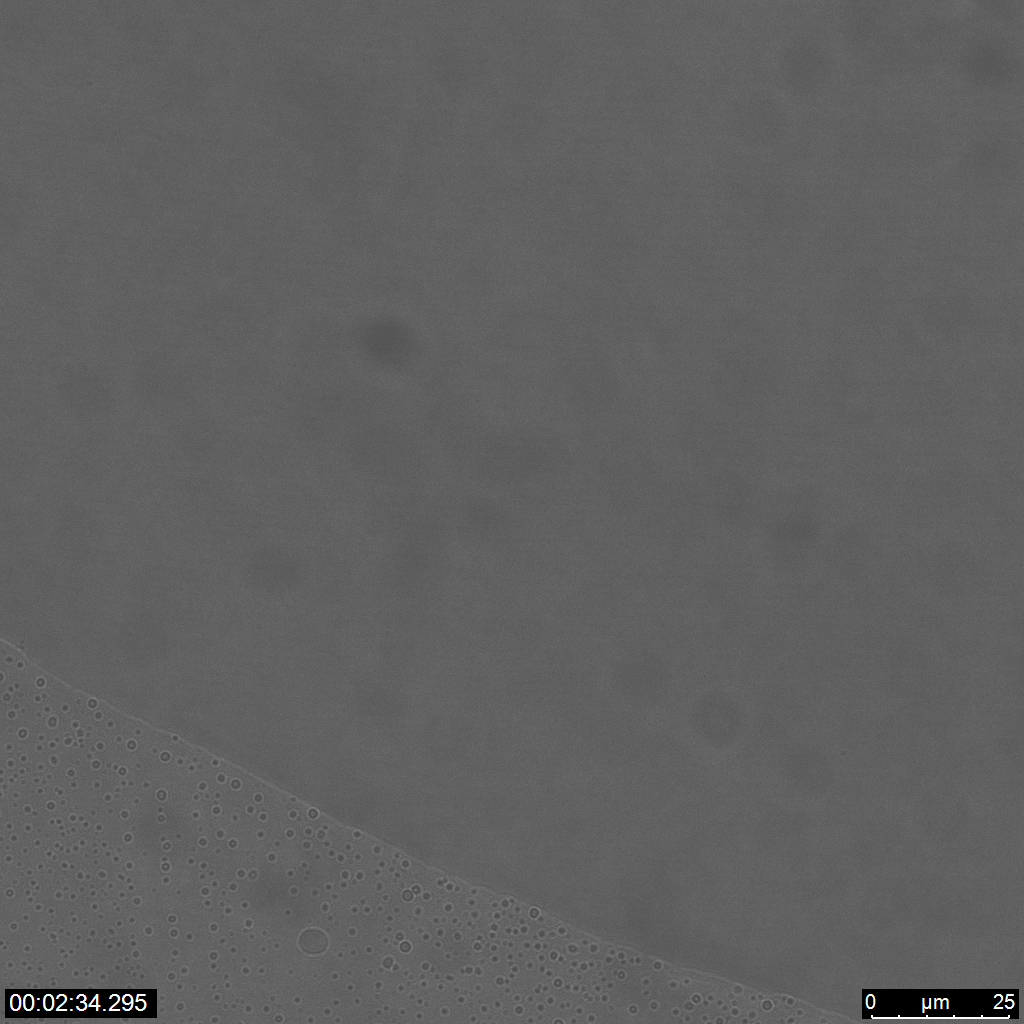

Supplement: Supplementary file 5 — Source data Fig. 2 [file 44319_2025_485_MOESM5_ESM.zip › Figure 2/2M/Fig. 2M_SAS_6_N_mCherry_BF.tif]

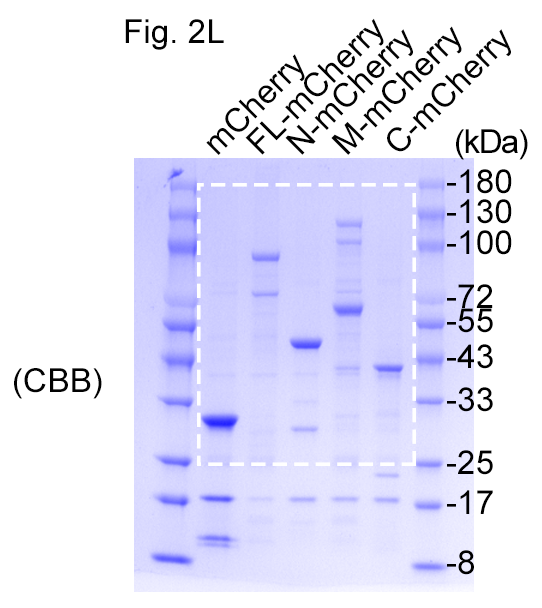

Supplement: Supplementary file 5 — Source data Fig. 2 [file 44319_2025_485_MOESM5_ESM.zip › Figure 2/2L/Fig. 2L.tif]

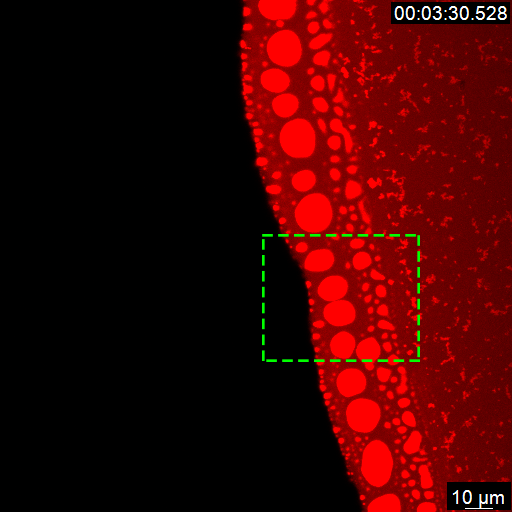

Supplement: Supplementary file 5 — Source data Fig. 2 [file 44319_2025_485_MOESM5_ESM.zip › Figure 2/2E/Fig. 2E_SAS_6_mCherry_0S.tif]

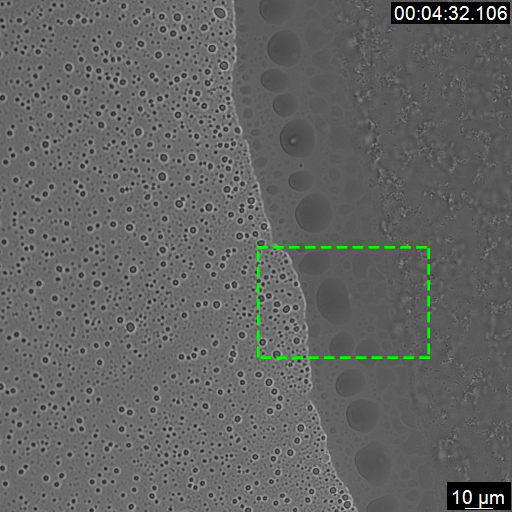

Supplement: Supplementary file 5 — Source data Fig. 2 [file 44319_2025_485_MOESM5_ESM.zip › Figure 2/2E/Fig. 2E_SAS_6_mCherry_60S_BF.tif]

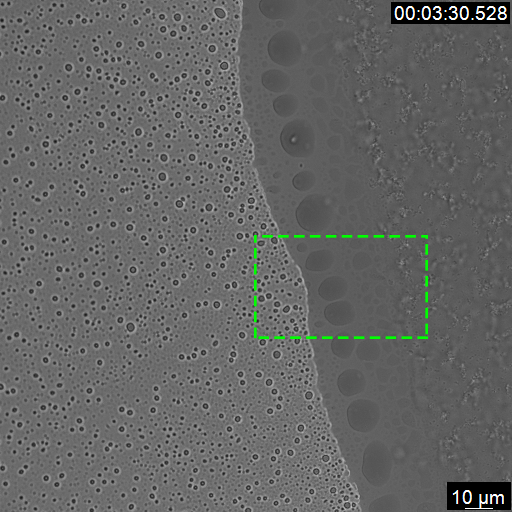

Supplement: Supplementary file 5 — Source data Fig. 2 [file 44319_2025_485_MOESM5_ESM.zip › Figure 2/2E/Fig. 2E_SAS_6_mCherry_0S_BF.tif]

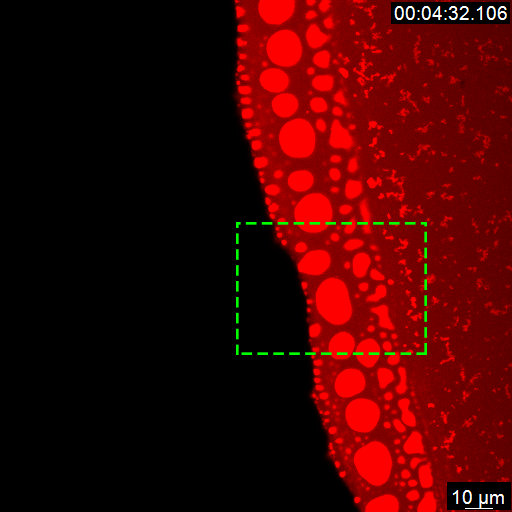

Supplement: Supplementary file 5 — Source data Fig. 2 [file 44319_2025_485_MOESM5_ESM.zip › Figure 2/2E/Fig. 2E_SAS_6_mCherry_60S.tif]

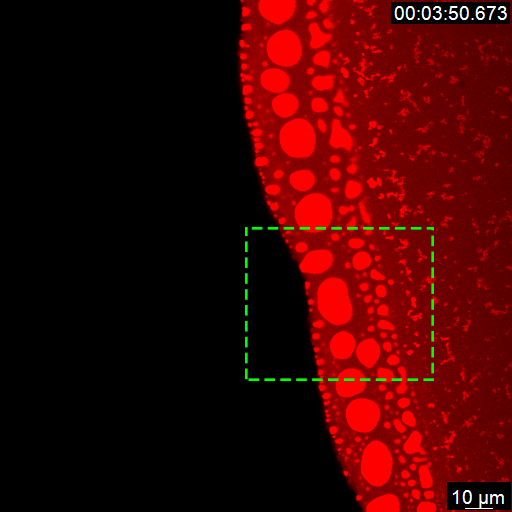

Supplement: Supplementary file 5 — Source data Fig. 2 [file 44319_2025_485_MOESM5_ESM.zip › Figure 2/2E/Fig. 2E_SAS_6_mCherry_20S.tif]

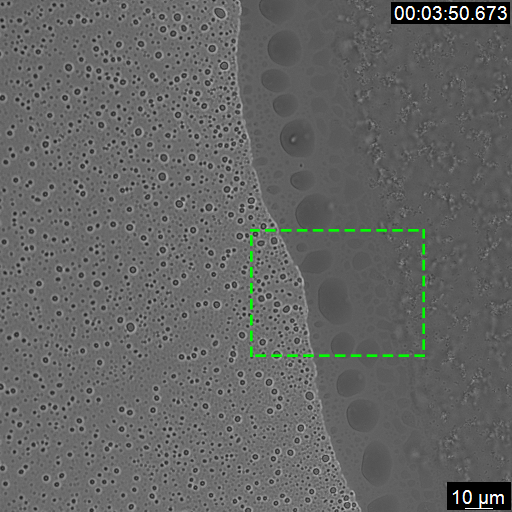

Supplement: Supplementary file 5 — Source data Fig. 2 [file 44319_2025_485_MOESM5_ESM.zip › Figure 2/2E/Fig. 2E_SAS_6_mCherry_20S_BF.tif]

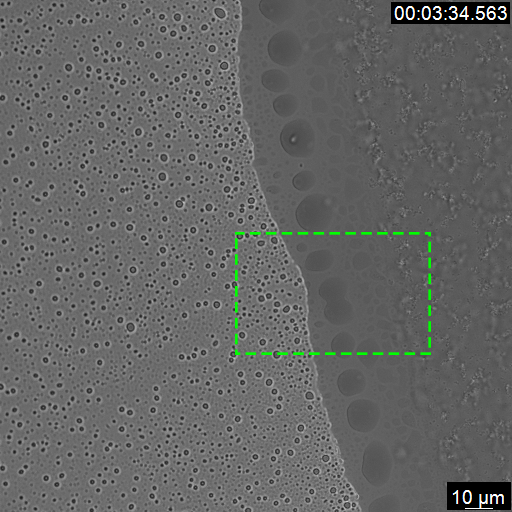

Supplement: Supplementary file 5 — Source data Fig. 2 [file 44319_2025_485_MOESM5_ESM.zip › Figure 2/2E/Fig. 2E_SAS_6_mCherry_5S_BF.tif]

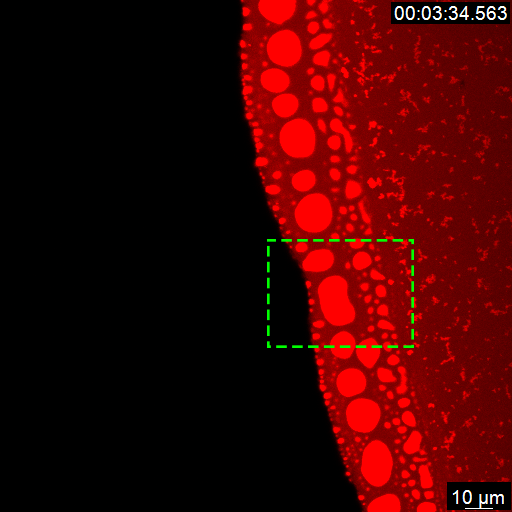

Supplement: Supplementary file 5 — Source data Fig. 2 [file 44319_2025_485_MOESM5_ESM.zip › Figure 2/2E/Fig. 2E_SAS_6_mCherry_5S.tif]

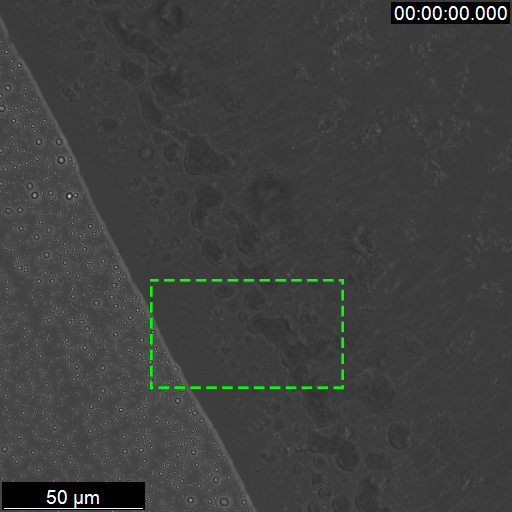

Supplement: Supplementary file 5 — Source data Fig. 2 [file 44319_2025_485_MOESM5_ESM.zip › Figure 2/2B/Fig. 2B_SAS_6_mCherry_0min_BF.tif]

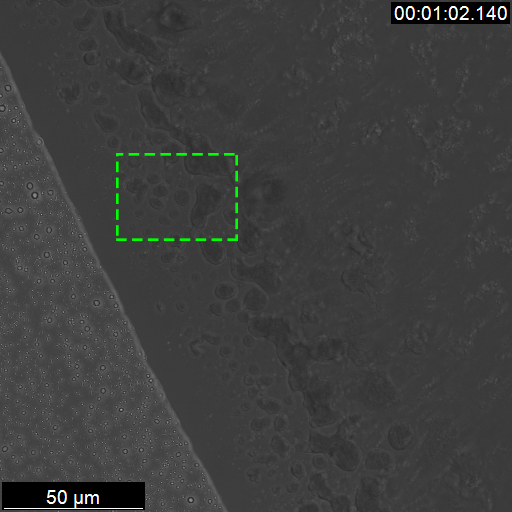

Supplement: Supplementary file 5 — Source data Fig. 2 [file 44319_2025_485_MOESM5_ESM.zip › Figure 2/2B/Fig. 2B_SAS_6_mCherry_1min_BF.tif]

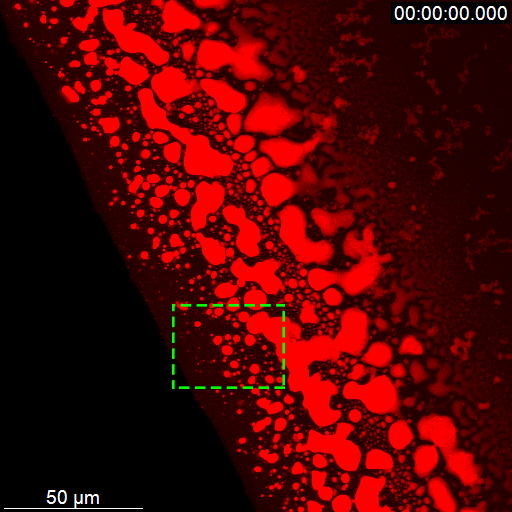

Supplement: Supplementary file 5 — Source data Fig. 2 [file 44319_2025_485_MOESM5_ESM.zip › Figure 2/2B/Fig. 2B_SAS_6_mCherry_0min.tif]

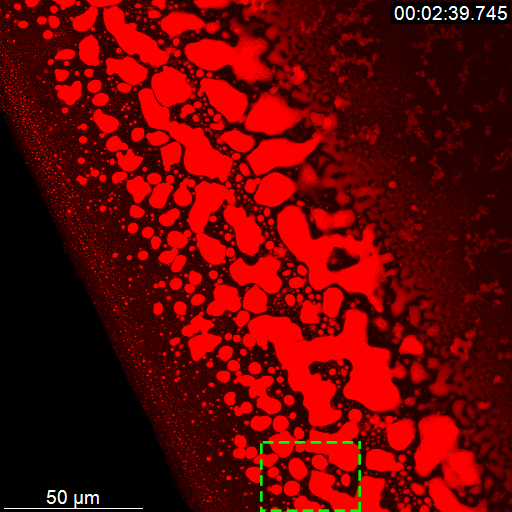

Supplement: Supplementary file 5 — Source data Fig. 2 [file 44319_2025_485_MOESM5_ESM.zip › Figure 2/2B/Fig. 2B_SAS_6_mCherry_2min.tif]

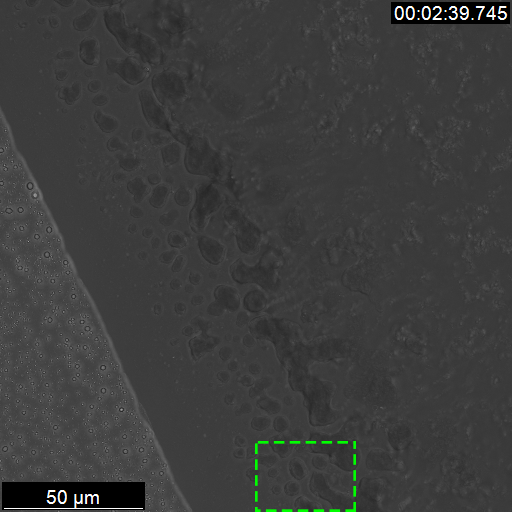

Supplement: Supplementary file 5 — Source data Fig. 2 [file 44319_2025_485_MOESM5_ESM.zip › Figure 2/2B/Fig. 2B_SAS_6_mCherry_2min_BF.tif]

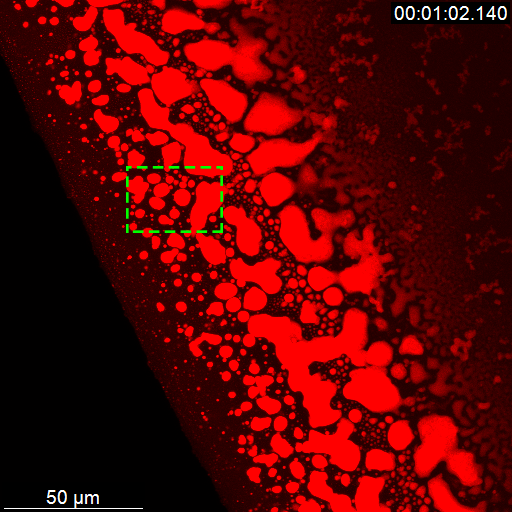

Supplement: Supplementary file 5 — Source data Fig. 2 [file 44319_2025_485_MOESM5_ESM.zip › Figure 2/2B/Fig. 2B_SAS_6_mCherry_1min.tif]

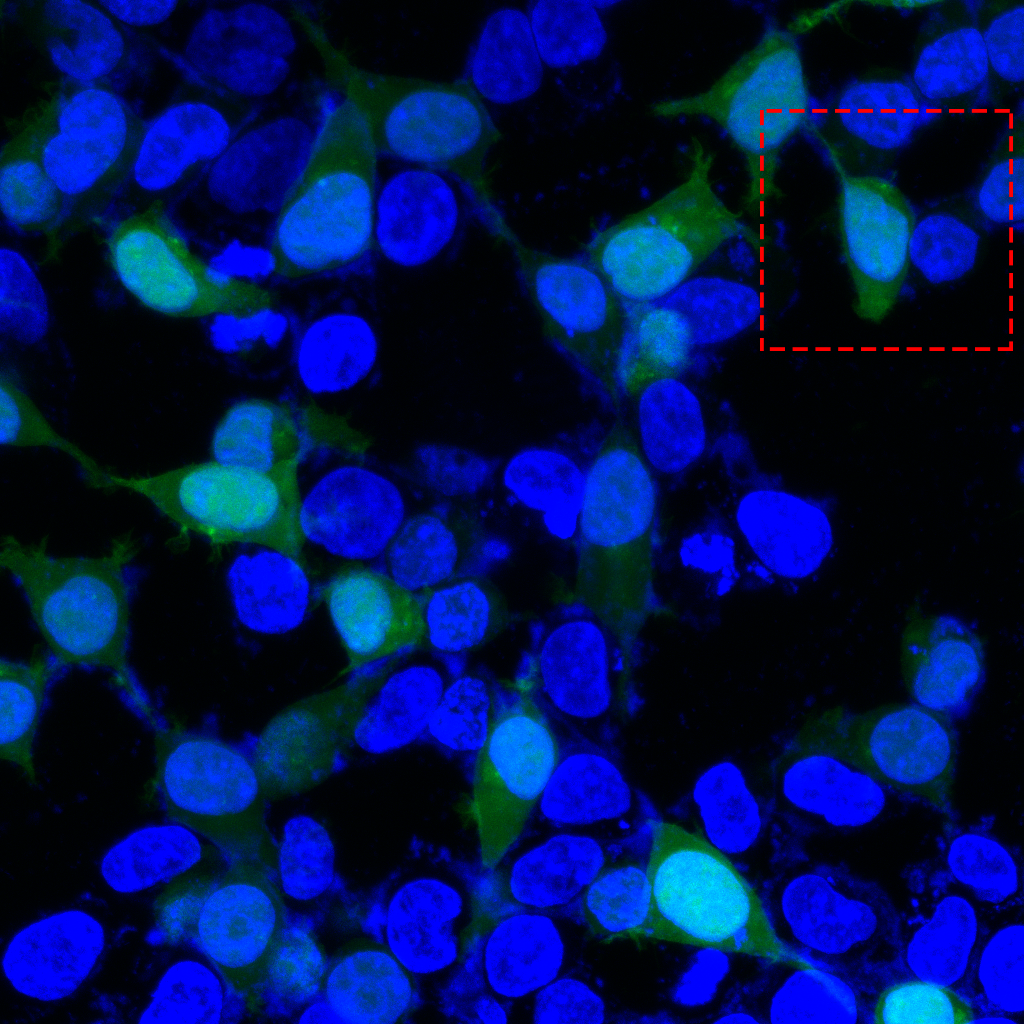

Supplement: Supplementary file 6 — Source data Fig. 3 [file 44319_2025_485_MOESM6_ESM.zip › Figure 3/3B/Fig. 3B_GFP_Vector.tif]

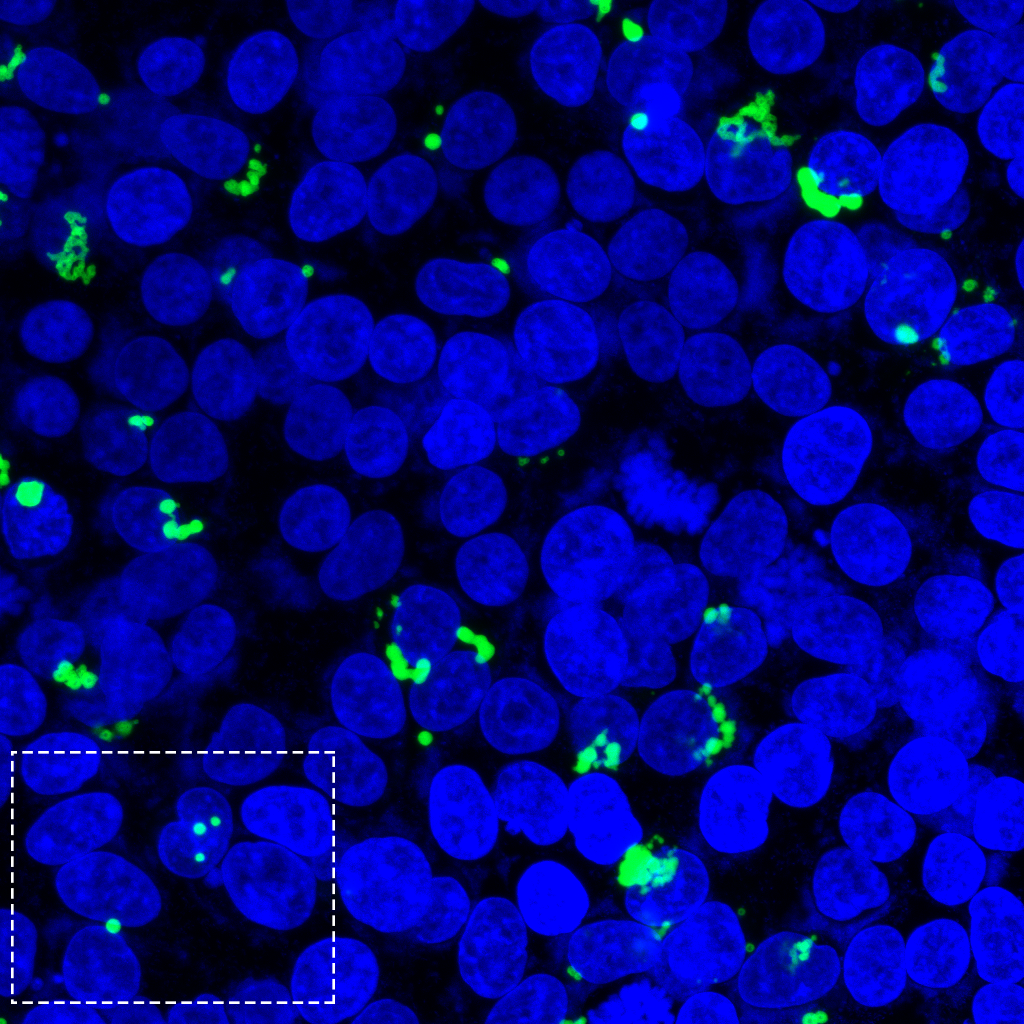

Supplement: Supplementary file 6 — Source data Fig. 3 [file 44319_2025_485_MOESM6_ESM.zip › Figure 3/3B/Fig. 3B_SAS6_GFP.tif]

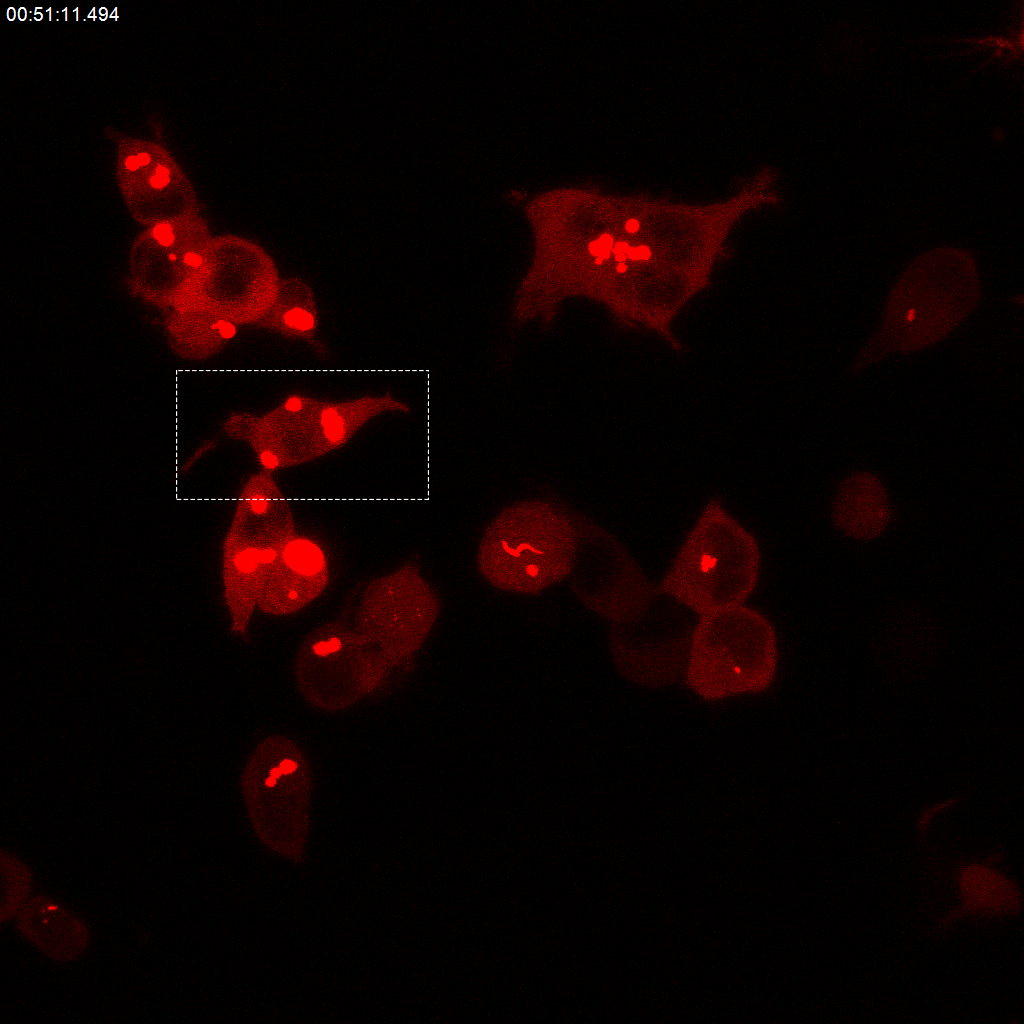

Supplement: Supplementary file 6 — Source data Fig. 3 [file 44319_2025_485_MOESM6_ESM.zip › Figure 3/3C/Fig. 3C_50min.tif]

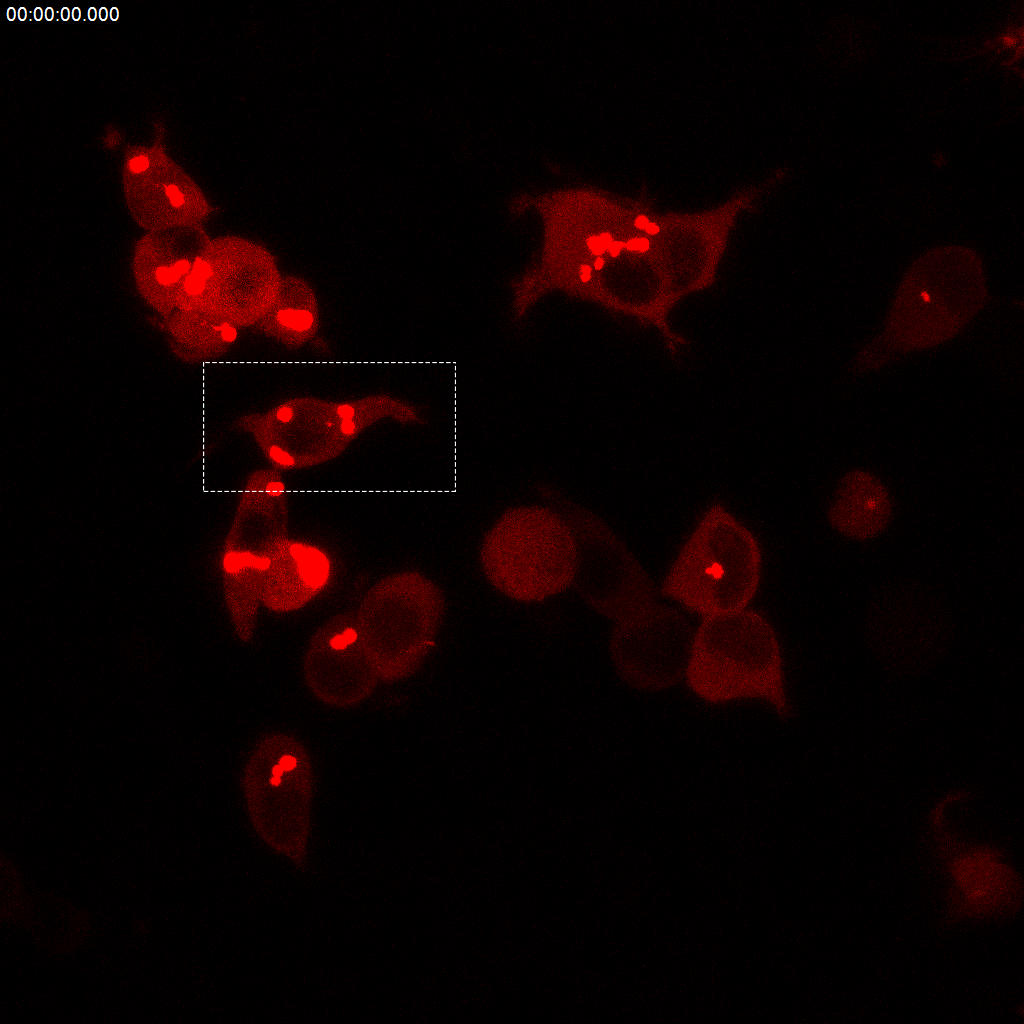

Supplement: Supplementary file 6 — Source data Fig. 3 [file 44319_2025_485_MOESM6_ESM.zip › Figure 3/3C/Fig. 3C_0min.tif]

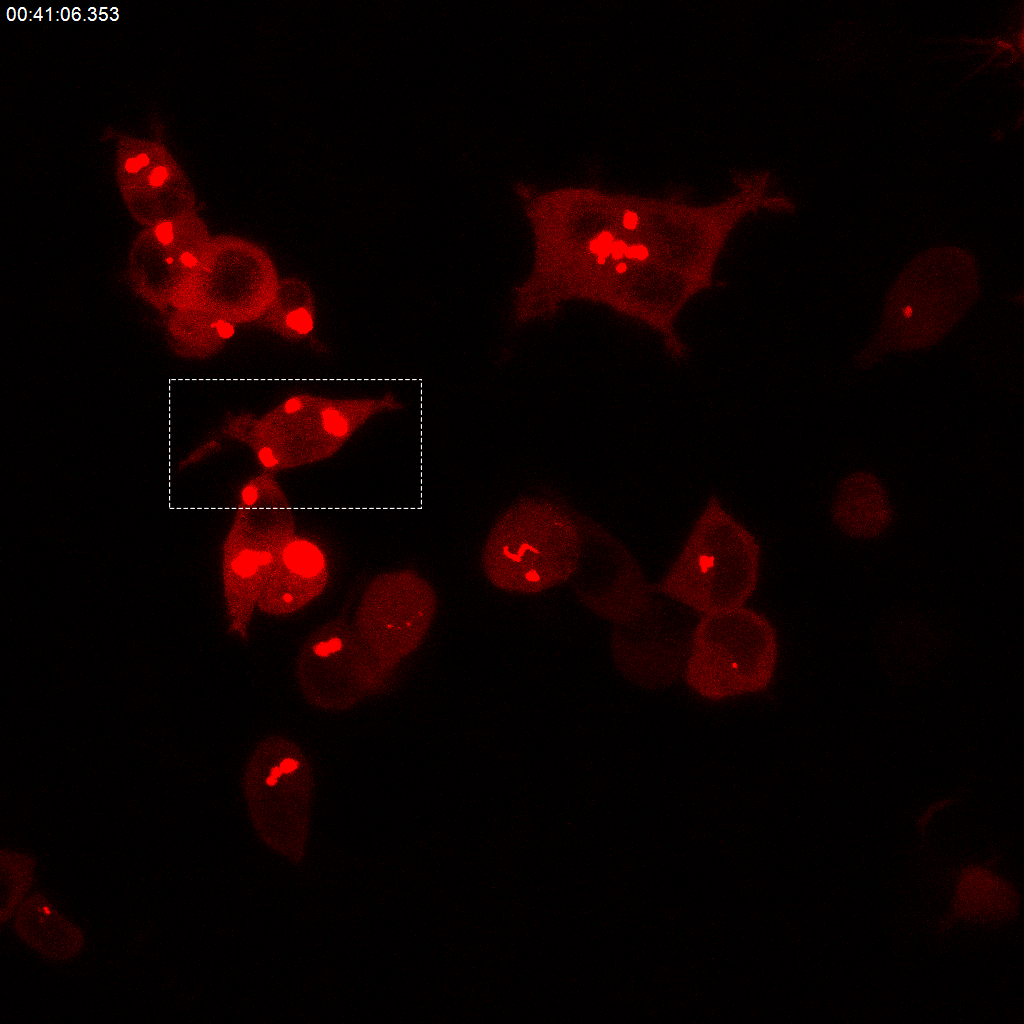

Supplement: Supplementary file 6 — Source data Fig. 3 [file 44319_2025_485_MOESM6_ESM.zip › Figure 3/3C/Fig. 3C_40min.tif]

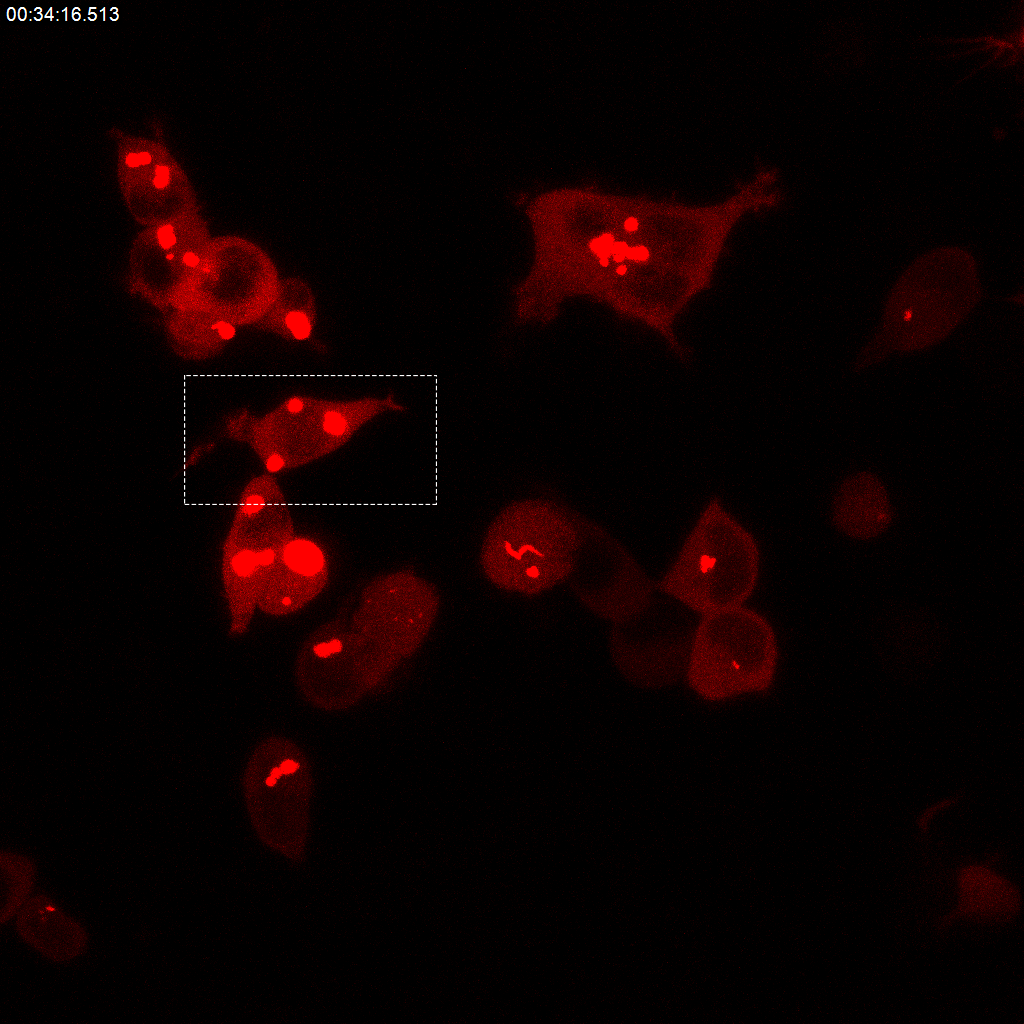

Supplement: Supplementary file 6 — Source data Fig. 3 [file 44319_2025_485_MOESM6_ESM.zip › Figure 3/3C/Fig. 3C_35min.tif]

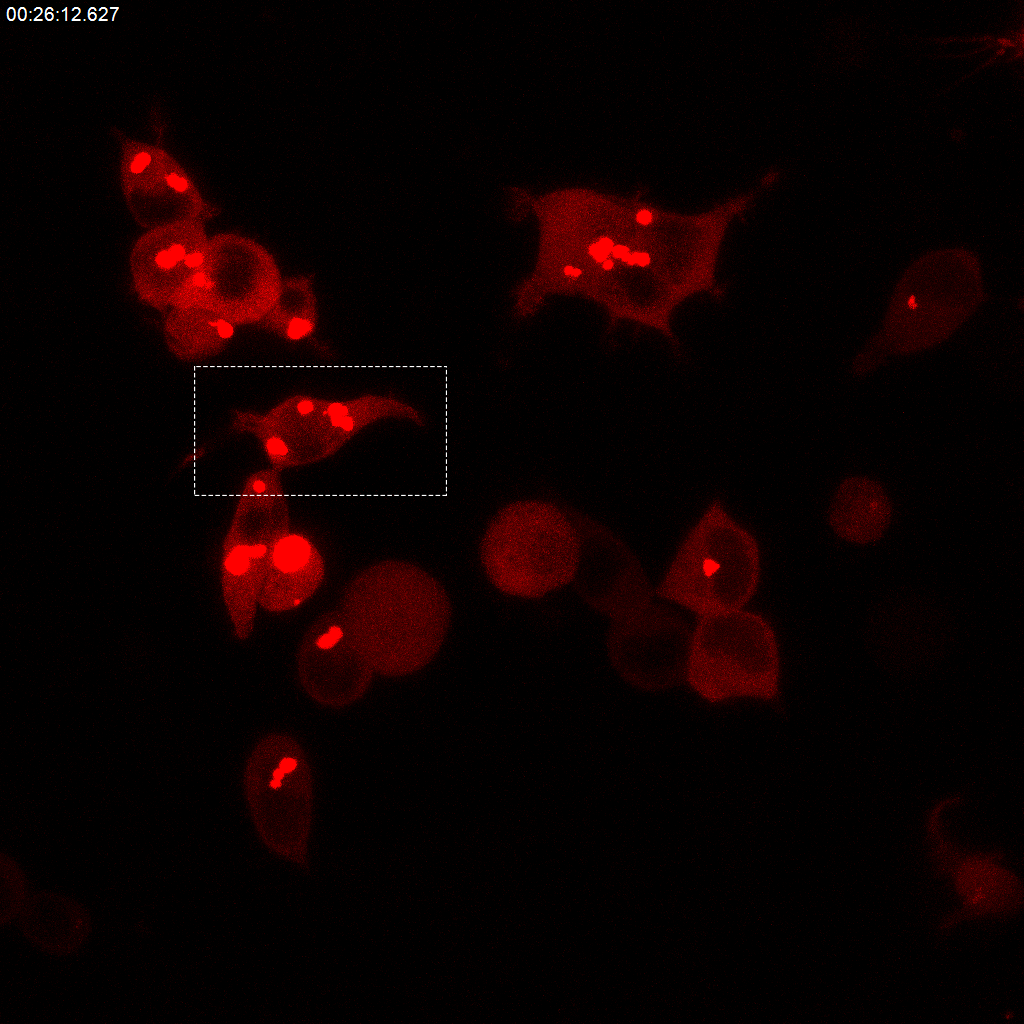

Supplement: Supplementary file 6 — Source data Fig. 3 [file 44319_2025_485_MOESM6_ESM.zip › Figure 3/3C/Fig. 3C_25min.tif]

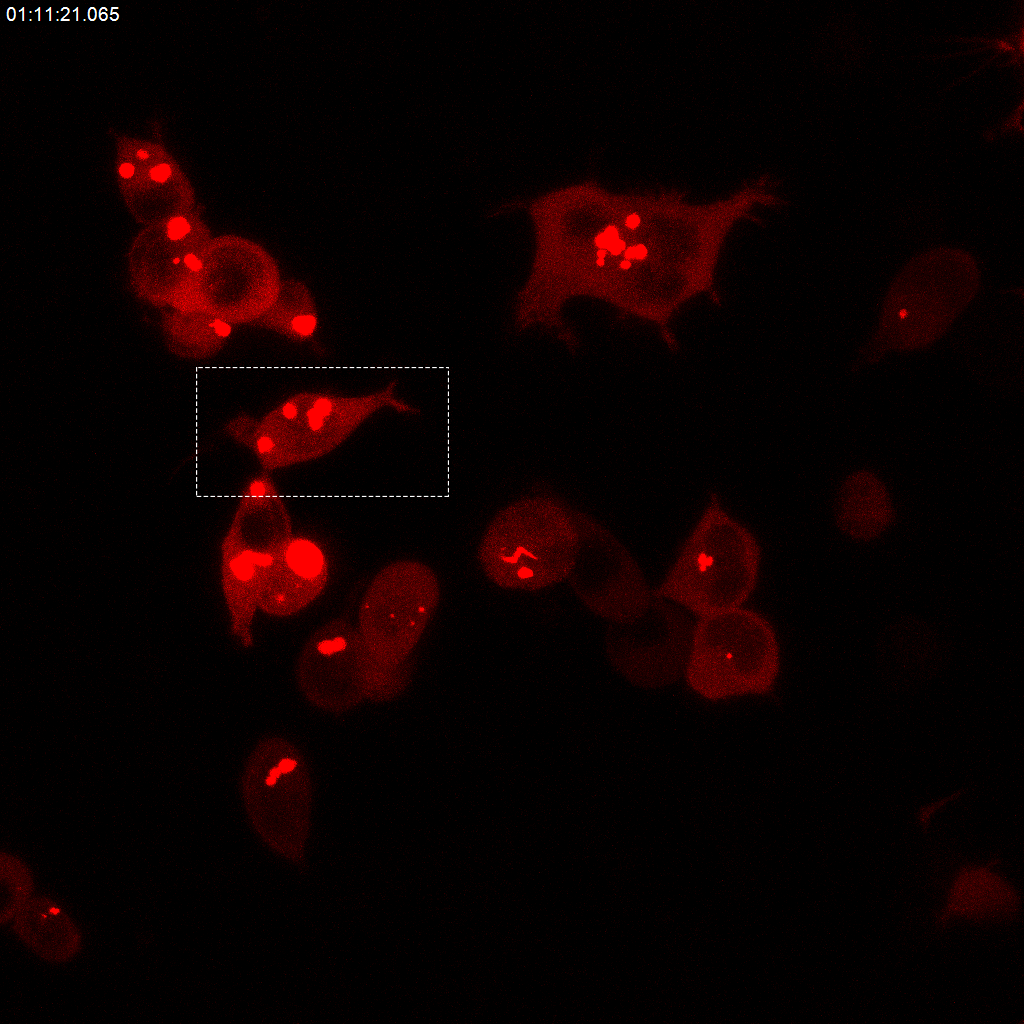

Supplement: Supplementary file 6 — Source data Fig. 3 [file 44319_2025_485_MOESM6_ESM.zip › Figure 3/3C/Fig. 3C_70min.tif]

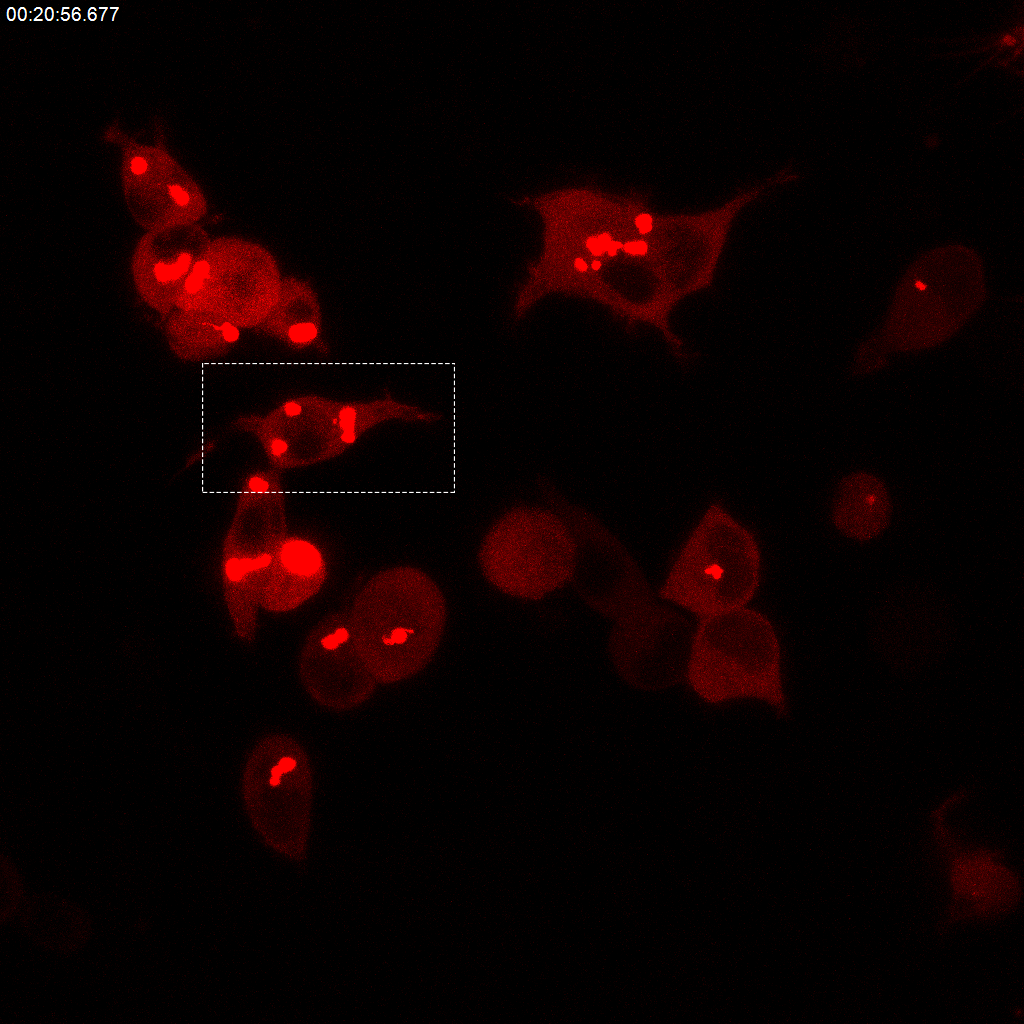

Supplement: Supplementary file 6 — Source data Fig. 3 [file 44319_2025_485_MOESM6_ESM.zip › Figure 3/3C/Fig. 3C_20min.tif]

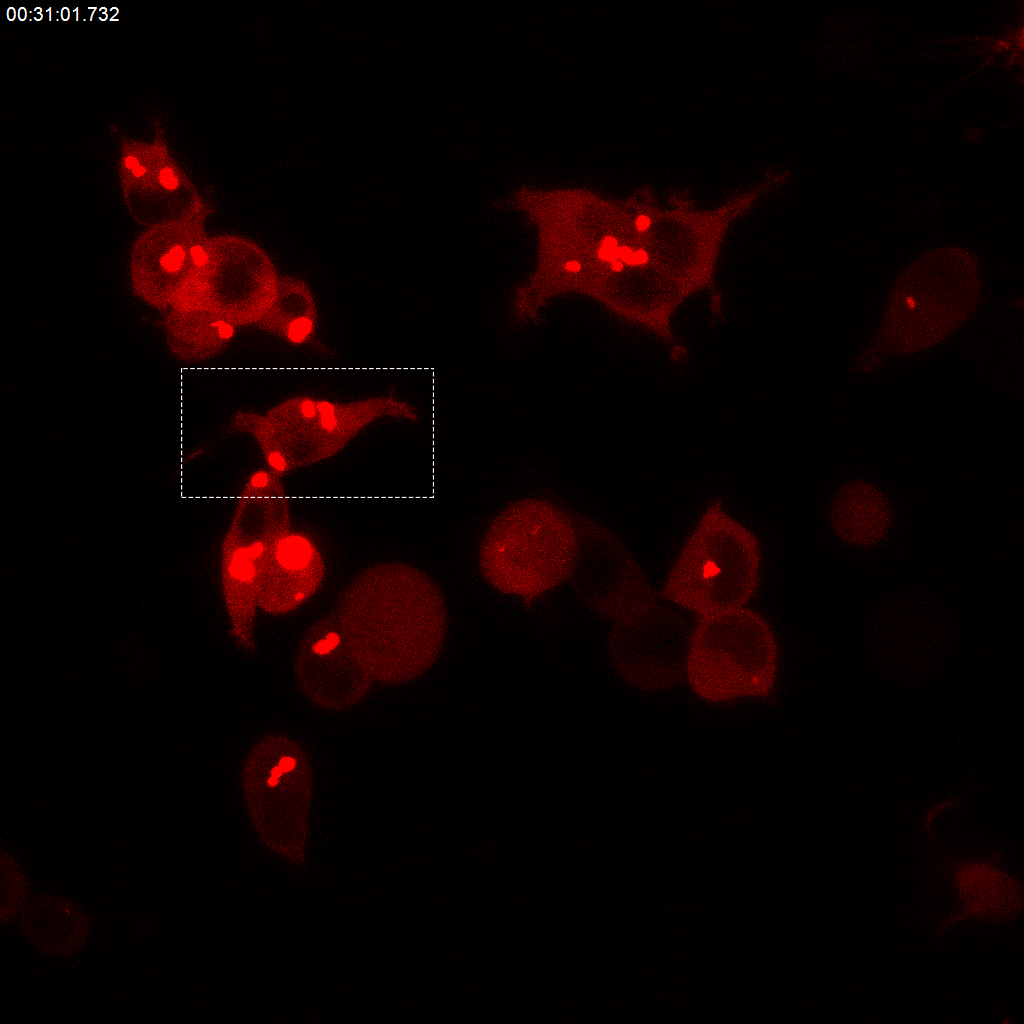

Supplement: Supplementary file 6 — Source data Fig. 3 [file 44319_2025_485_MOESM6_ESM.zip › Figure 3/3C/Fig. 3C_30min.tif]

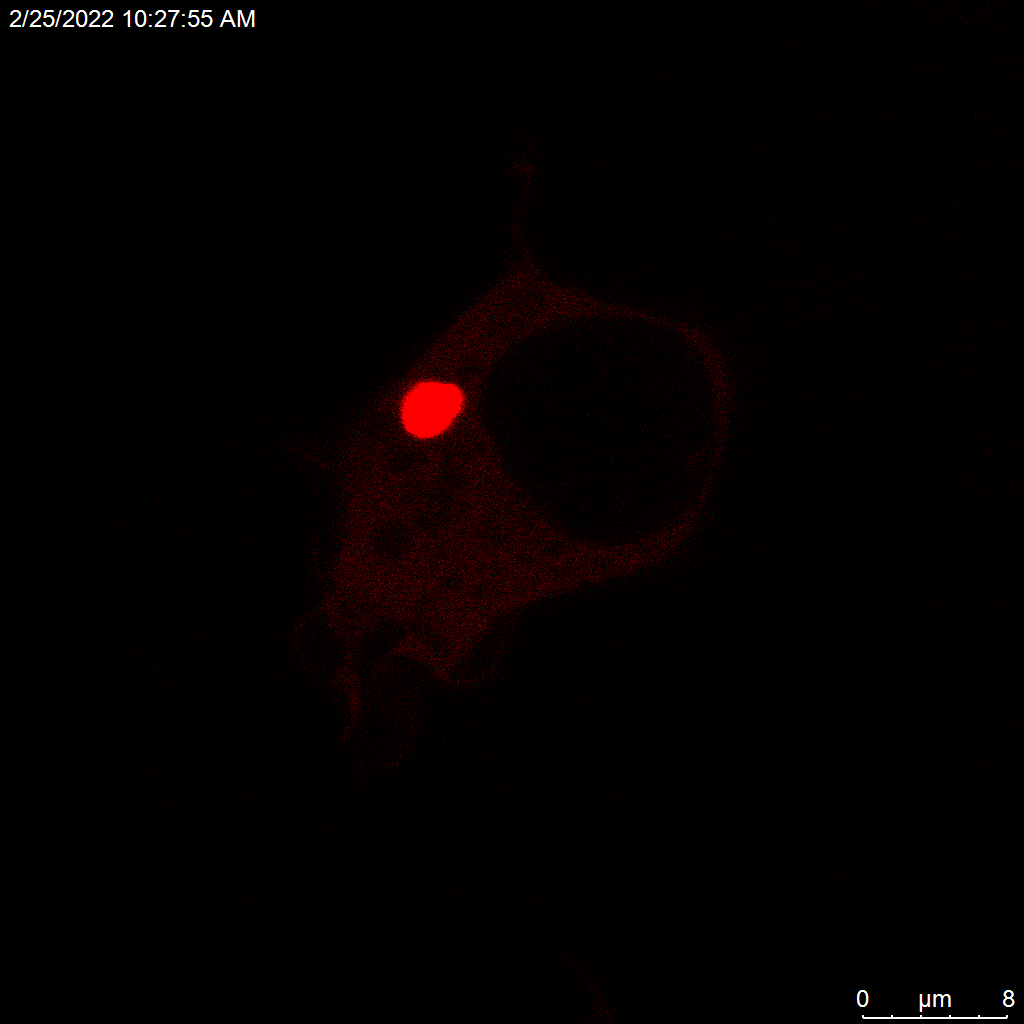

Supplement: Supplementary file 6 — Source data Fig. 3 [file 44319_2025_485_MOESM6_ESM.zip › Figure 3/3D/Fig. 3D_minus30s.tif]

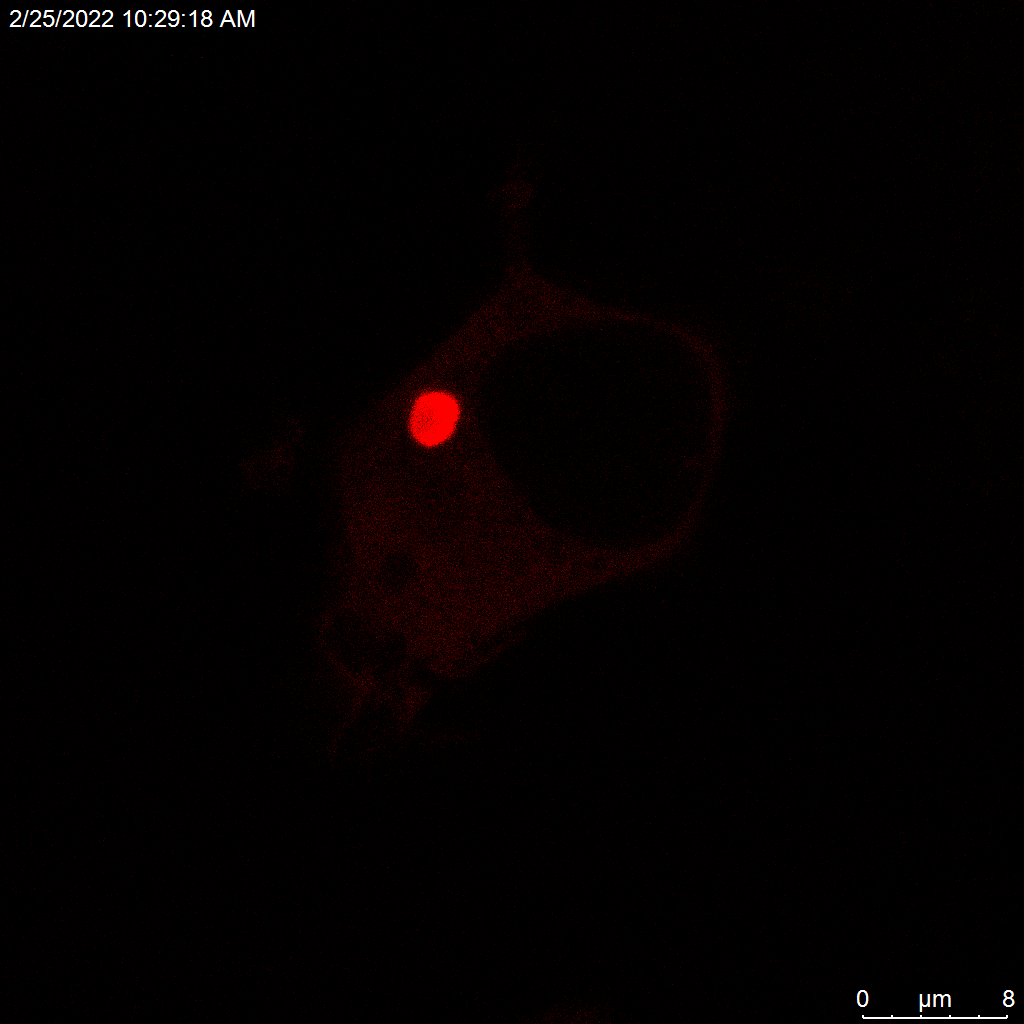

Supplement: Supplementary file 6 — Source data Fig. 3 [file 44319_2025_485_MOESM6_ESM.zip › Figure 3/3D/Fig. 3D_50s.tif]

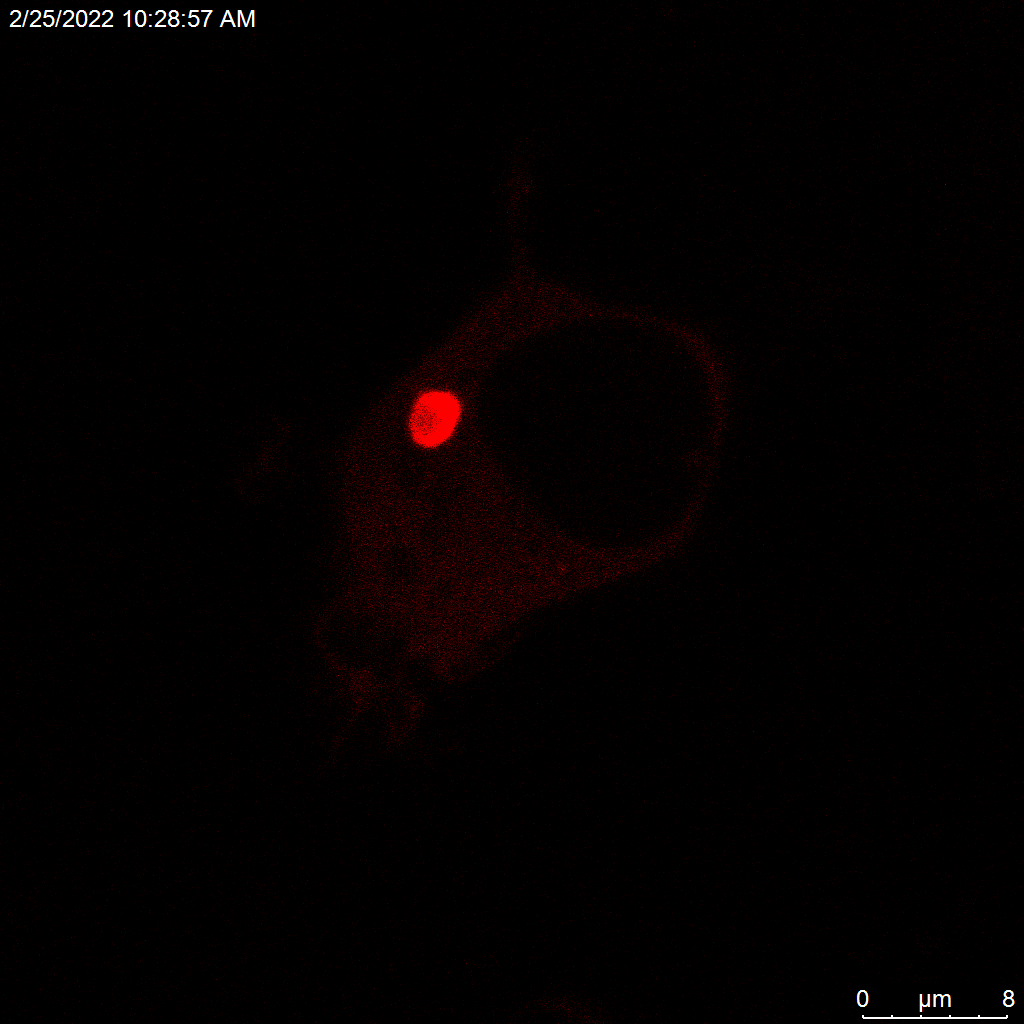

Supplement: Supplementary file 6 — Source data Fig. 3 [file 44319_2025_485_MOESM6_ESM.zip › Figure 3/3D/Fig. 3D_30s.tif]

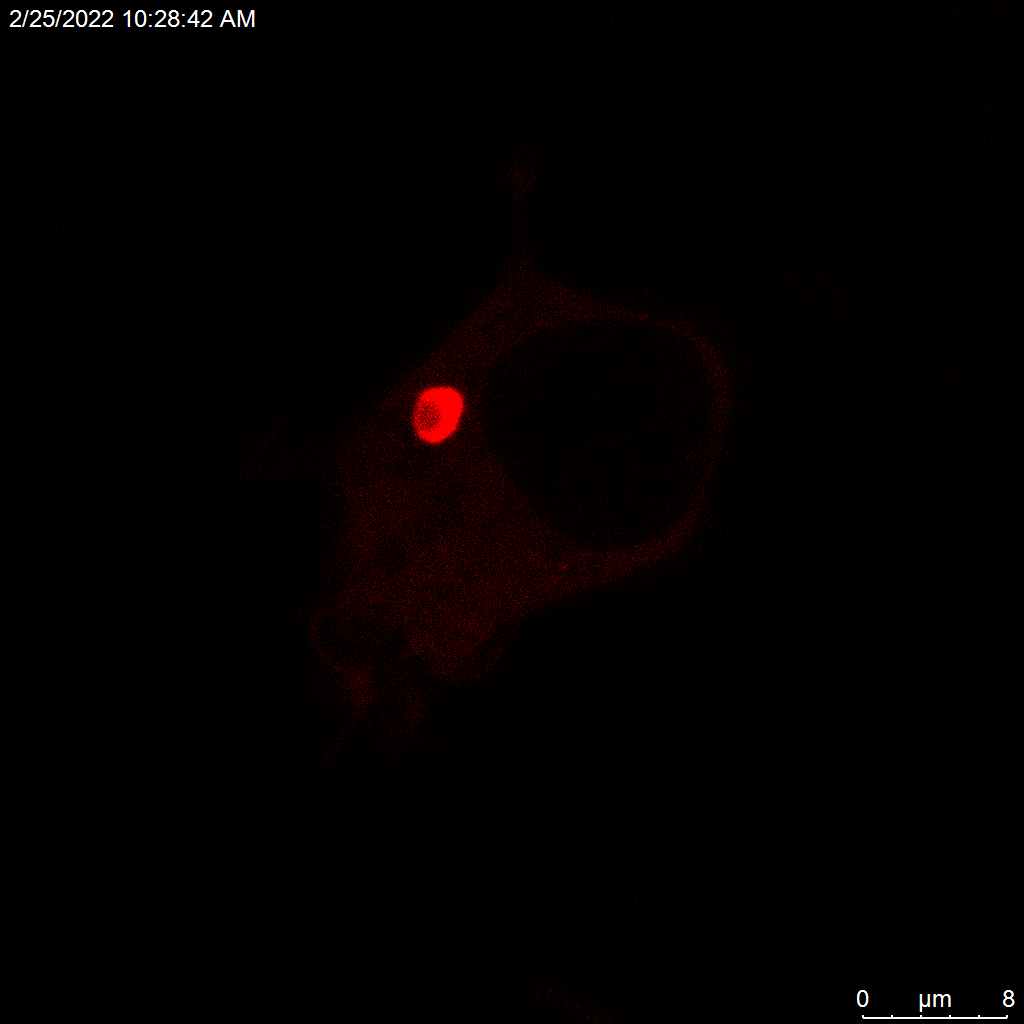

Supplement: Supplementary file 6 — Source data Fig. 3 [file 44319_2025_485_MOESM6_ESM.zip › Figure 3/3D/Fig. 3D_15s.tif]

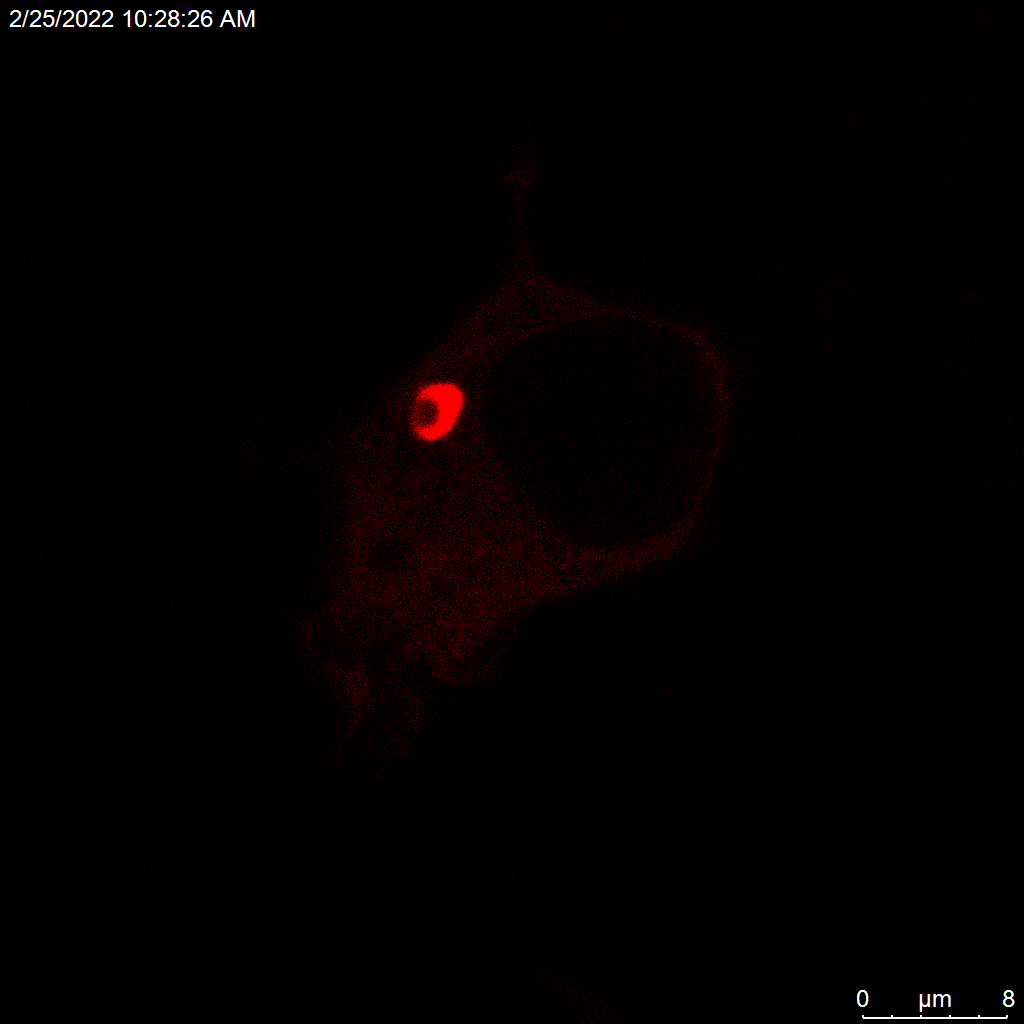

Supplement: Supplementary file 6 — Source data Fig. 3 [file 44319_2025_485_MOESM6_ESM.zip › Figure 3/3D/Fig. 3D_0s.tif]

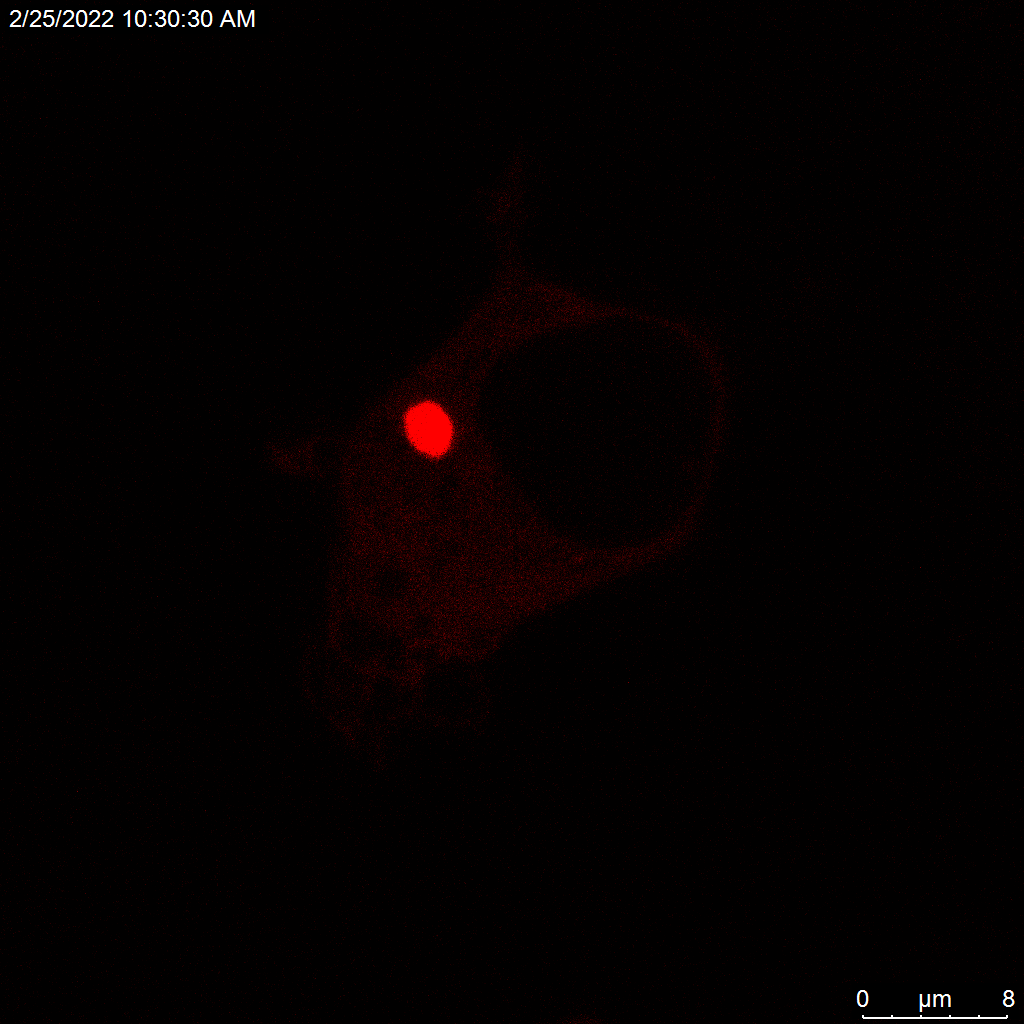

Supplement: Supplementary file 6 — Source data Fig. 3 [file 44319_2025_485_MOESM6_ESM.zip › Figure 3/3D/Fig. 3D_120s.tif]

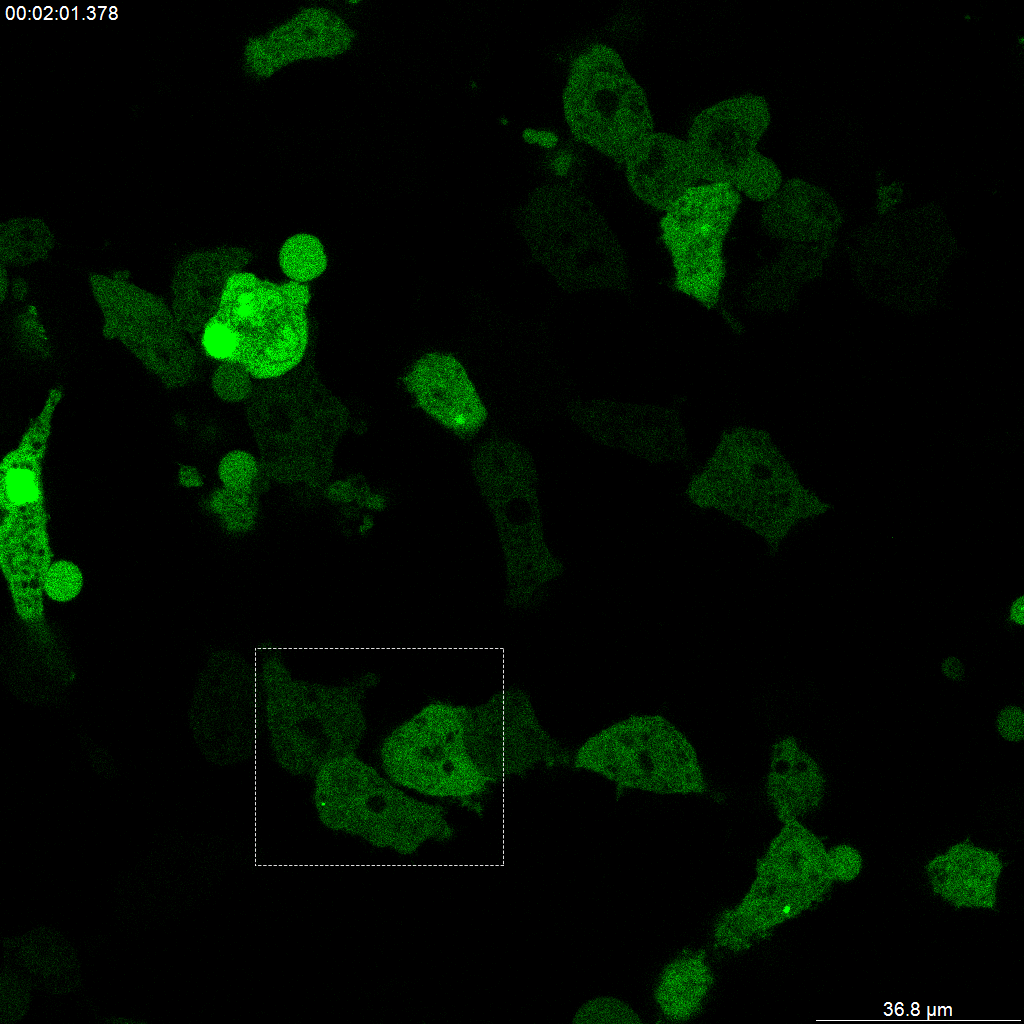

Supplement: Supplementary file 6 — Source data Fig. 3 [file 44319_2025_485_MOESM6_ESM.zip › Figure 3/3H/Fig. 3H_GFP_120S.tif]

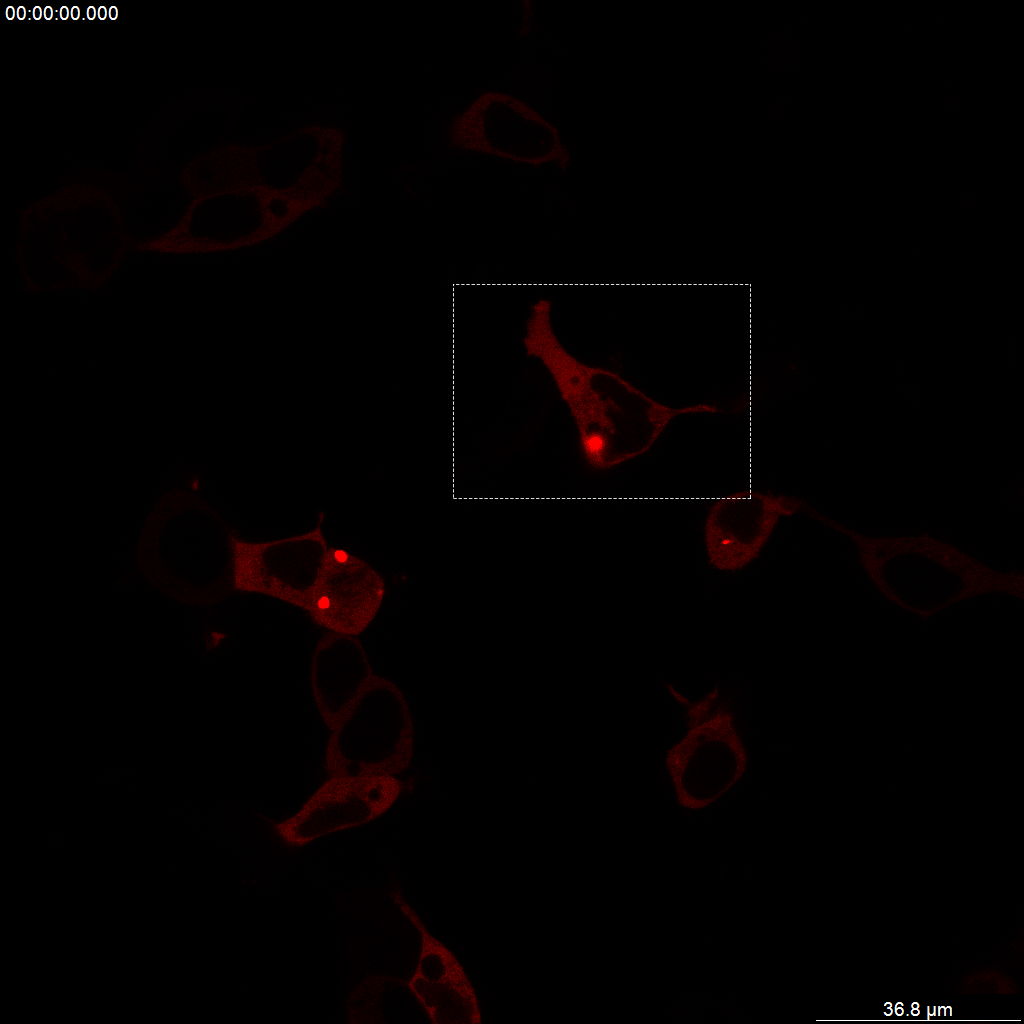

Supplement: Supplementary file 6 — Source data Fig. 3 [file 44319_2025_485_MOESM6_ESM.zip › Figure 3/3H/Fig. 3H_mCherry_0S.tif]
