# Supplementary figures and images for: Dynamic SAS-6 phosphorylation aids centrosome duplication and elimination in C. elegans oogenesis (part 2 of 3)
Source: EMBO Rep. 2025 May 23;26(13):3411–44. doi: 10.1038/s44319-025-00485-7 (PMC12238530; doi:10.1038/s44319-025-00485-7)

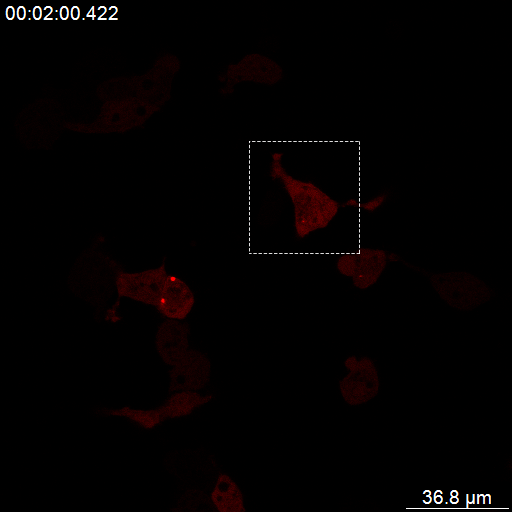

Supplement: Supplementary file 6 — Source data Fig. 3 [file 44319_2025_485_MOESM6_ESM.zip › Figure 3/3H/Fig. 3H_mCherry_120S.tif]

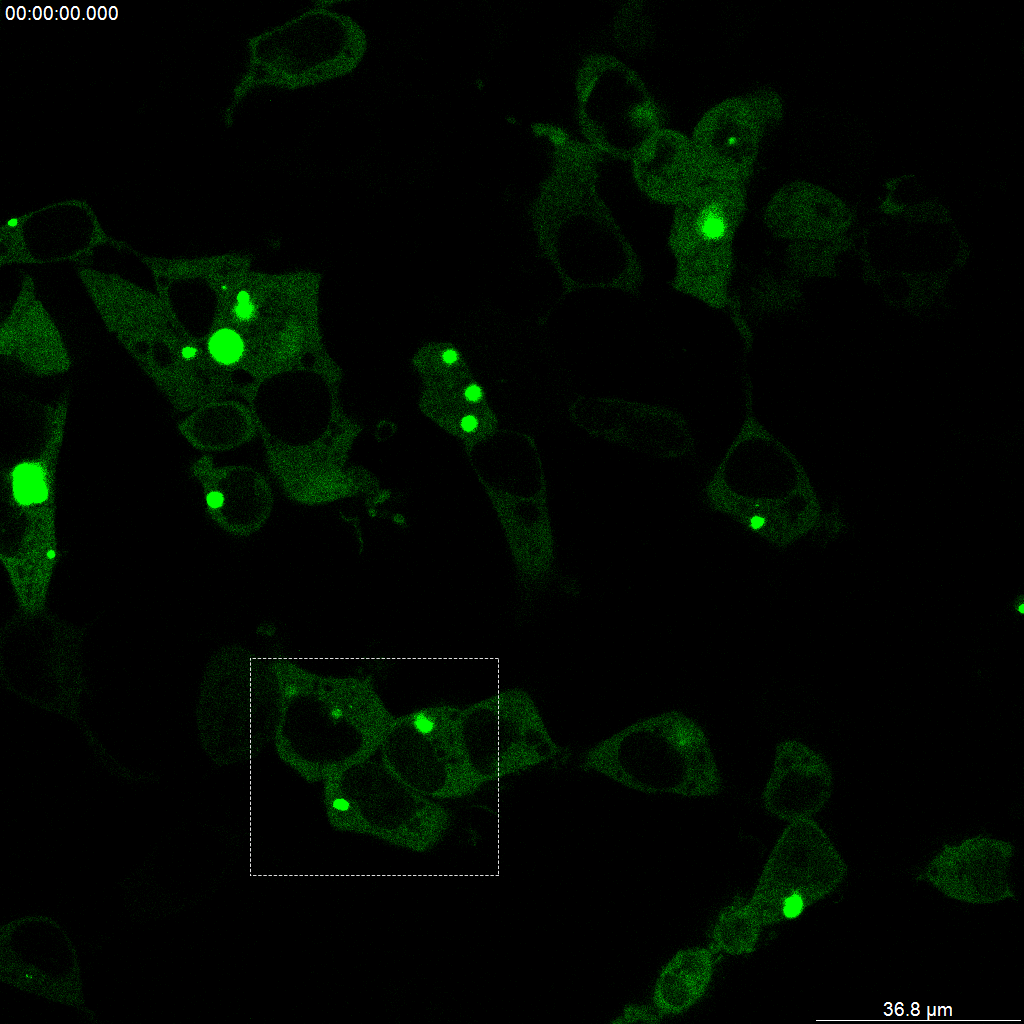

Supplement: Supplementary file 6 — Source data Fig. 3 [file 44319_2025_485_MOESM6_ESM.zip › Figure 3/3H/Fig. 3H_GFP_0S.tif]

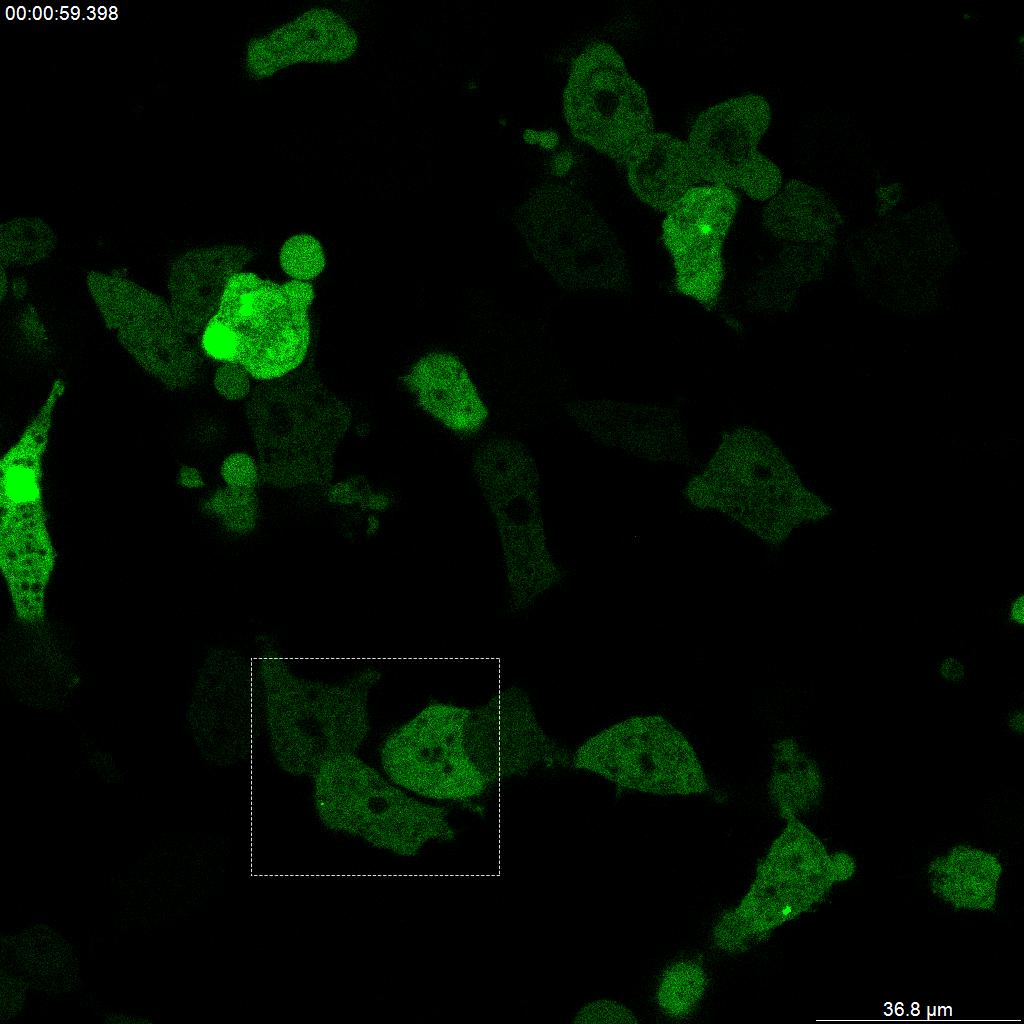

Supplement: Supplementary file 6 — Source data Fig. 3 [file 44319_2025_485_MOESM6_ESM.zip › Figure 3/3H/Fig. 3H_GFP_60S.tif]

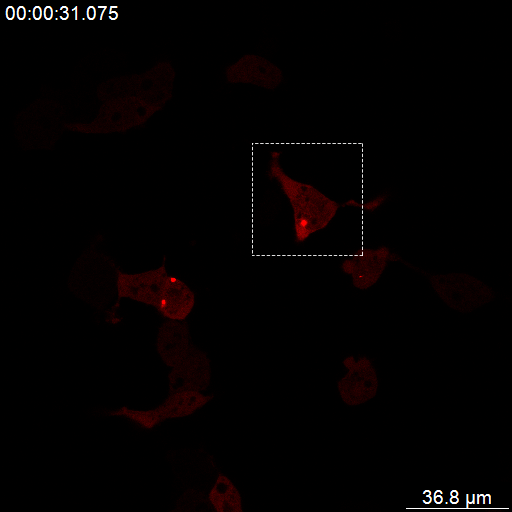

Supplement: Supplementary file 6 — Source data Fig. 3 [file 44319_2025_485_MOESM6_ESM.zip › Figure 3/3H/Fig. 3H_mCherry_30S.tif]

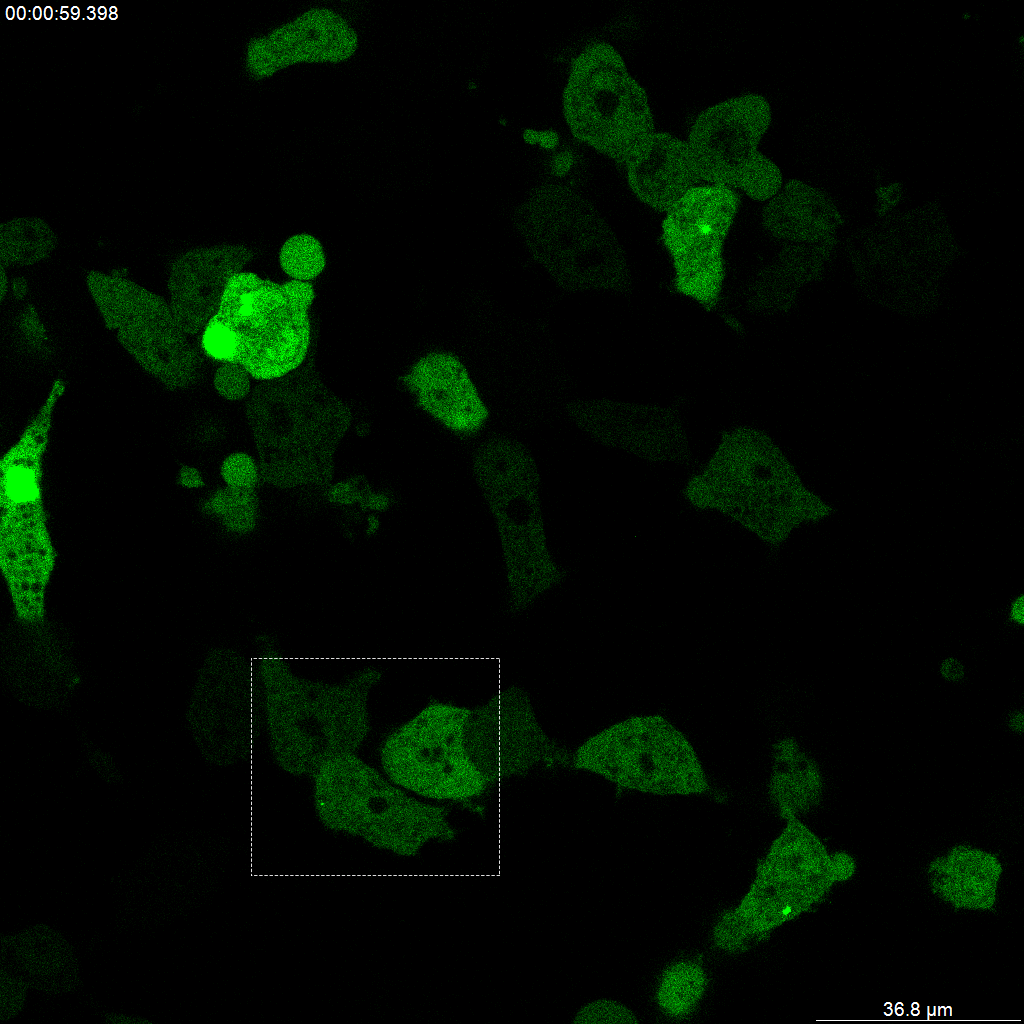

Supplement: Supplementary file 6 — Source data Fig. 3 [file 44319_2025_485_MOESM6_ESM.zip › Figure 3/3H/SAS-6-gfp 10% hexanediol_Series001_t023_z00_ch00.tif]

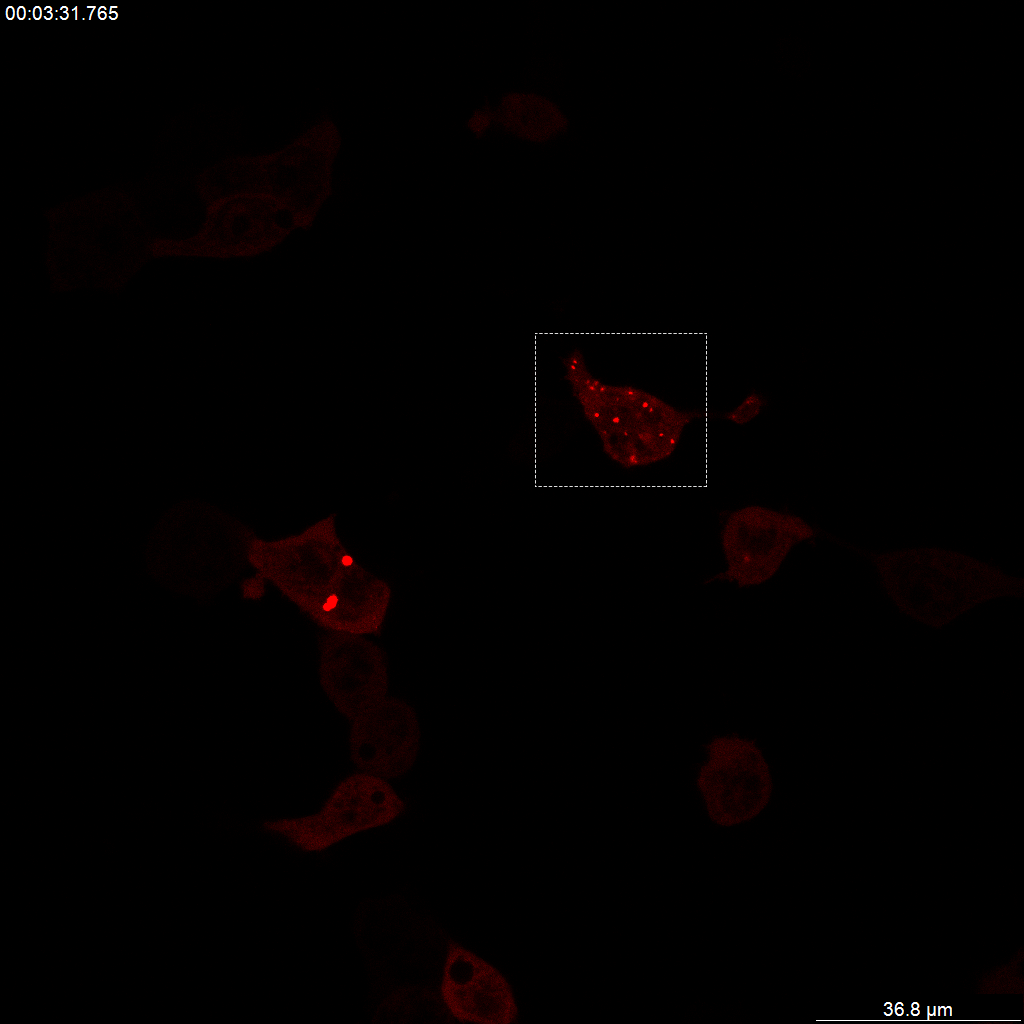

Supplement: Supplementary file 6 — Source data Fig. 3 [file 44319_2025_485_MOESM6_ESM.zip › Figure 3/3H/Fig. 3H_mCherry_210S.tif]

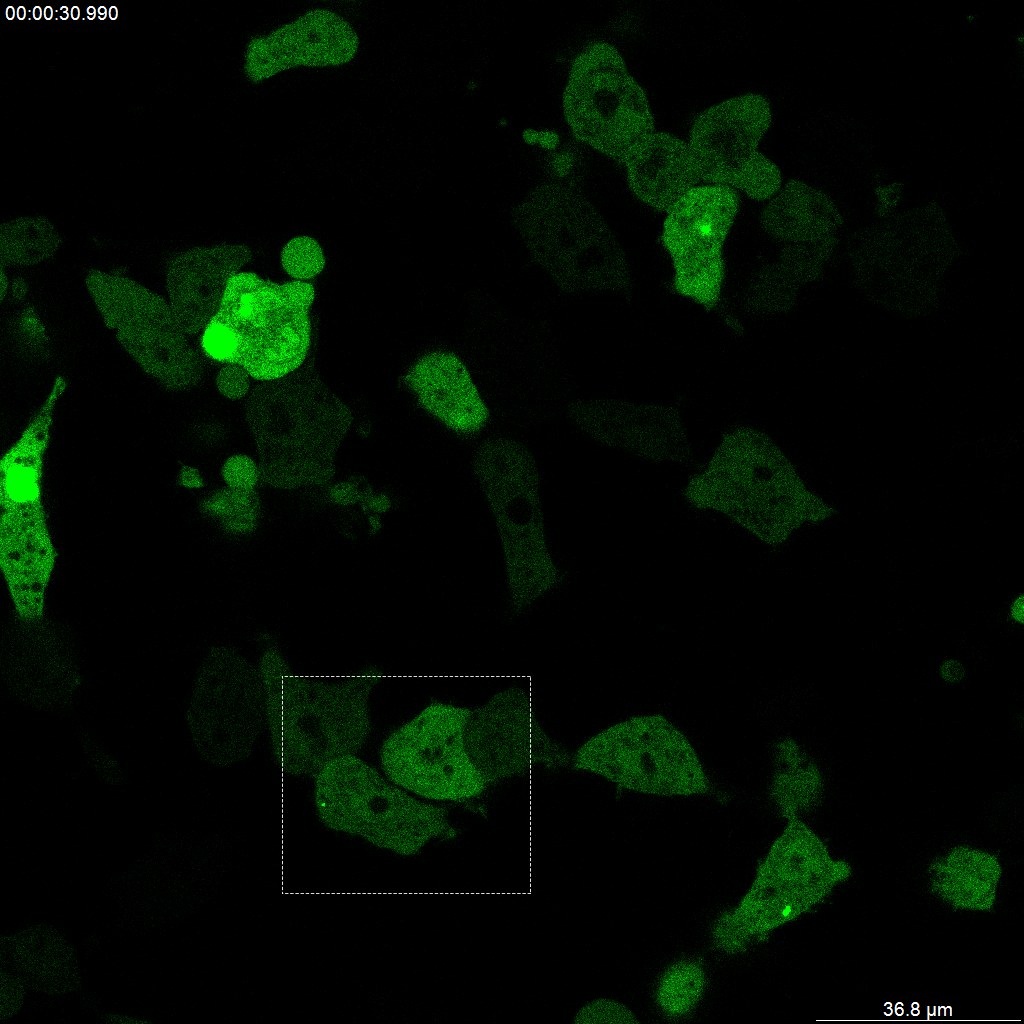

Supplement: Supplementary file 6 — Source data Fig. 3 [file 44319_2025_485_MOESM6_ESM.zip › Figure 3/3H/Fig. 3H_GFP_30S.tif]

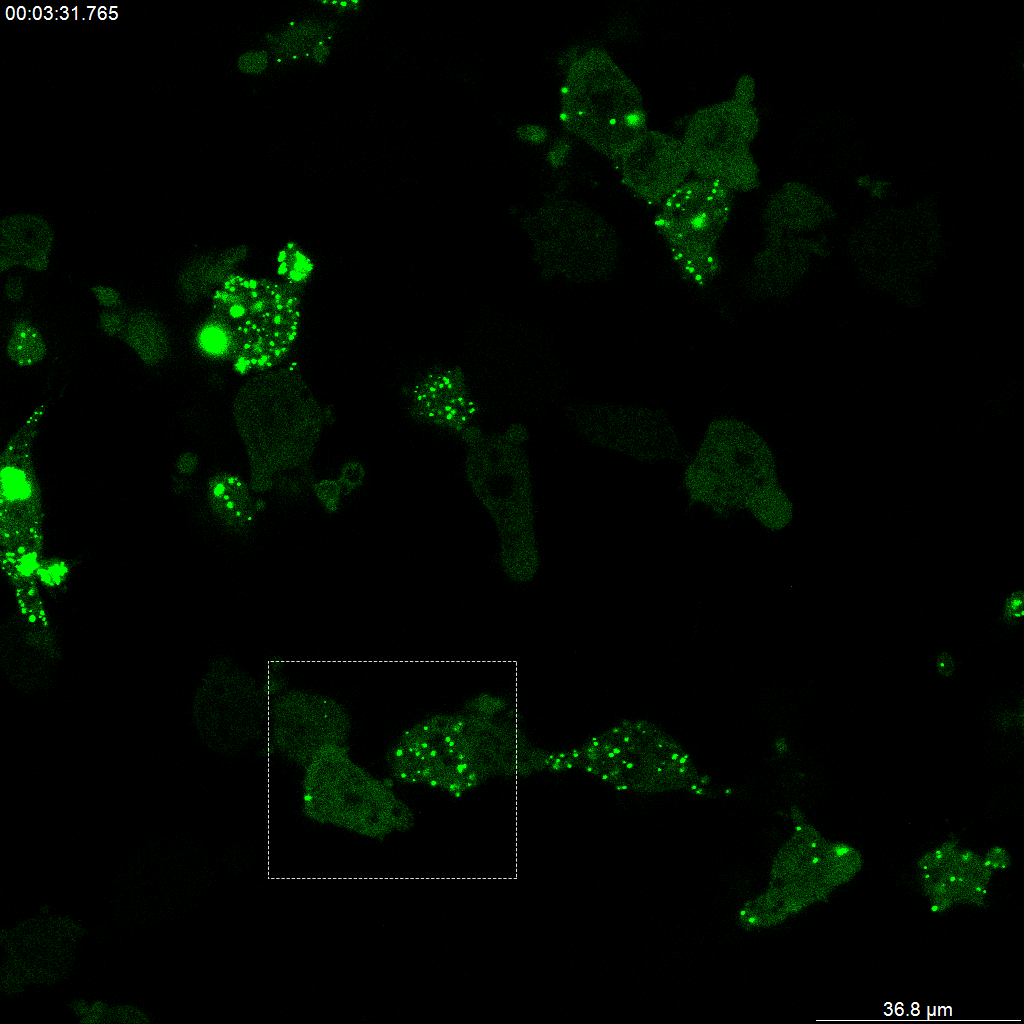

Supplement: Supplementary file 6 — Source data Fig. 3 [file 44319_2025_485_MOESM6_ESM.zip › Figure 3/3H/Fig. 3H_GFP_210S.tif]

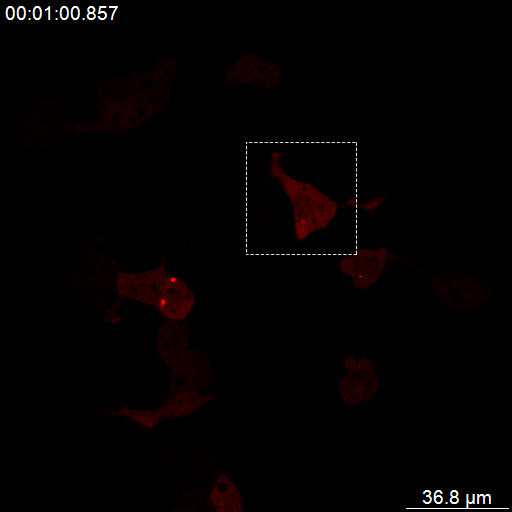

Supplement: Supplementary file 6 — Source data Fig. 3 [file 44319_2025_485_MOESM6_ESM.zip › Figure 3/3H/Fig. 3H_mCherry_60S.tif]

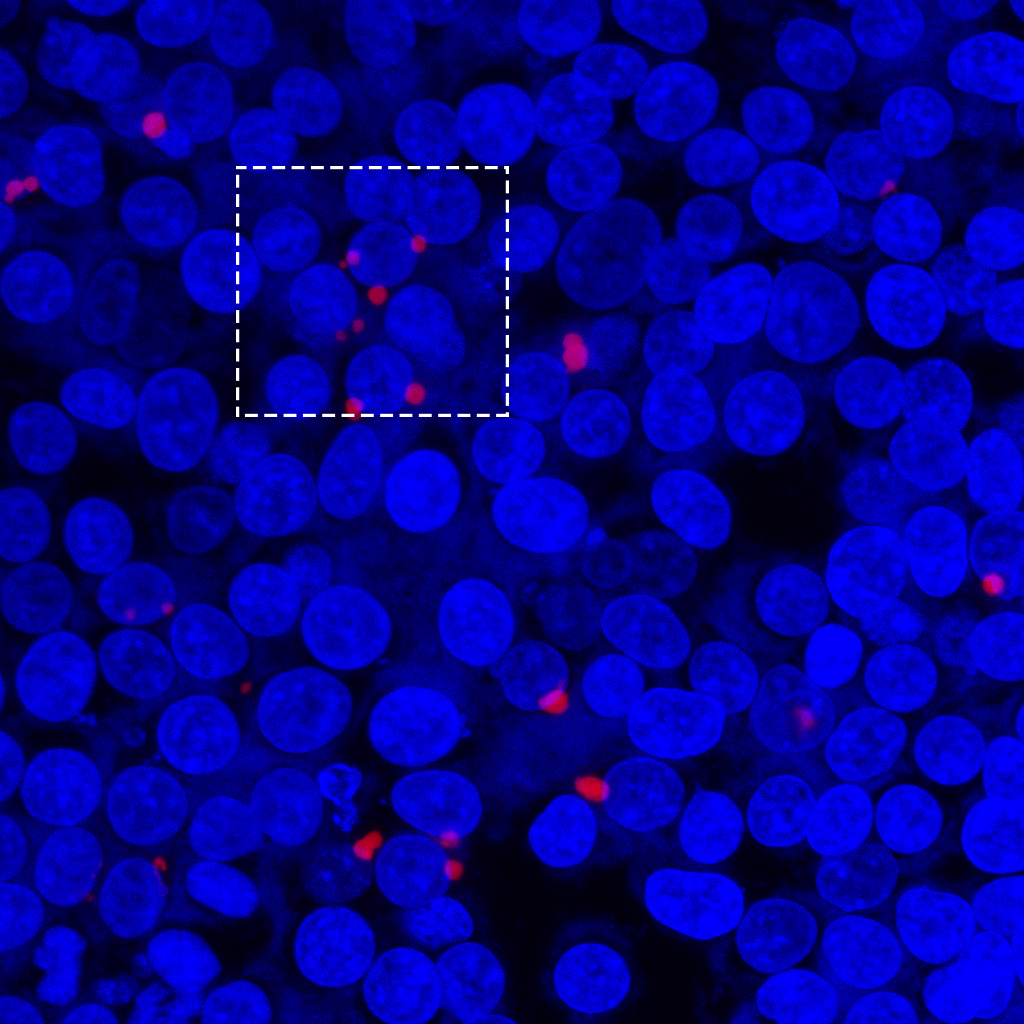

Supplement: Supplementary file 6 — Source data Fig. 3 [file 44319_2025_485_MOESM6_ESM.zip › Figure 3/3A/Fig. 3A_SAS6_mCherry.tif]

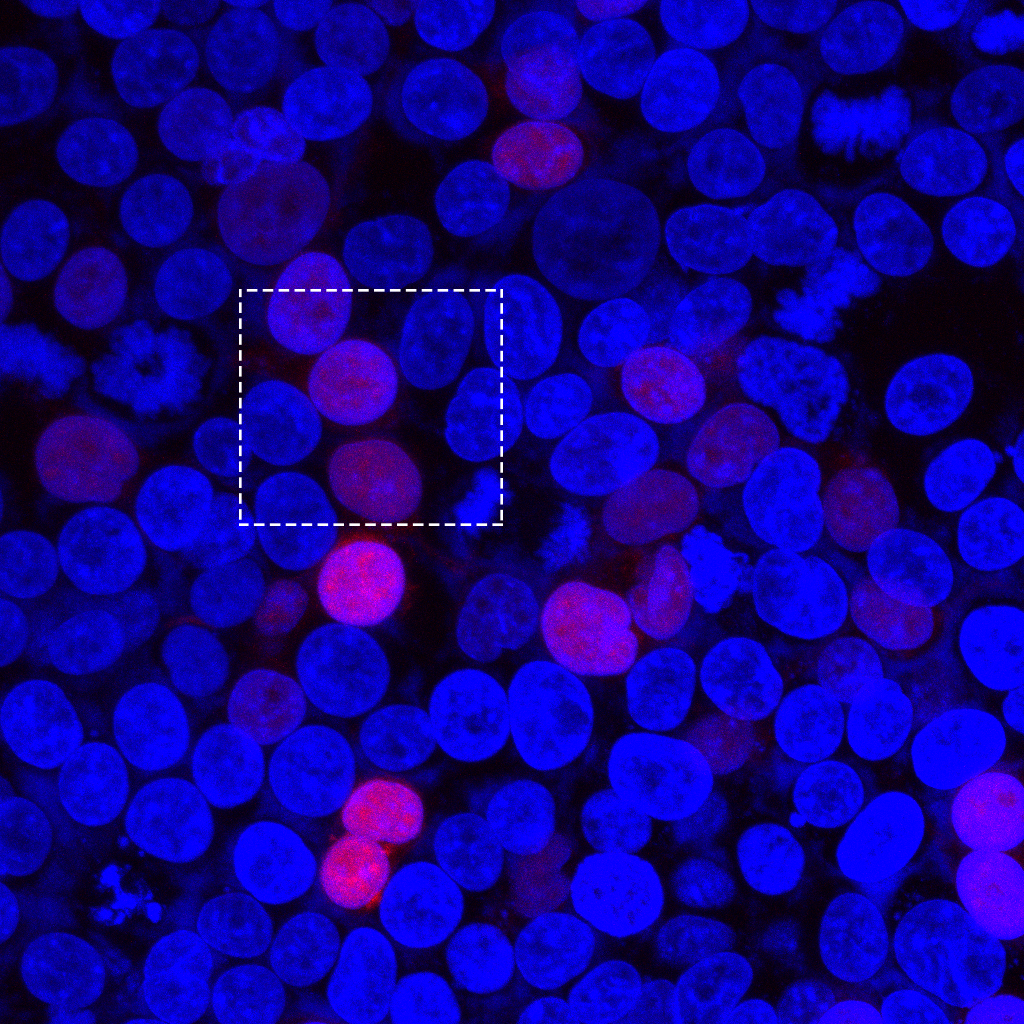

Supplement: Supplementary file 6 — Source data Fig. 3 [file 44319_2025_485_MOESM6_ESM.zip › Figure 3/3A/Fig. 3A_mCherry_vector.tif]

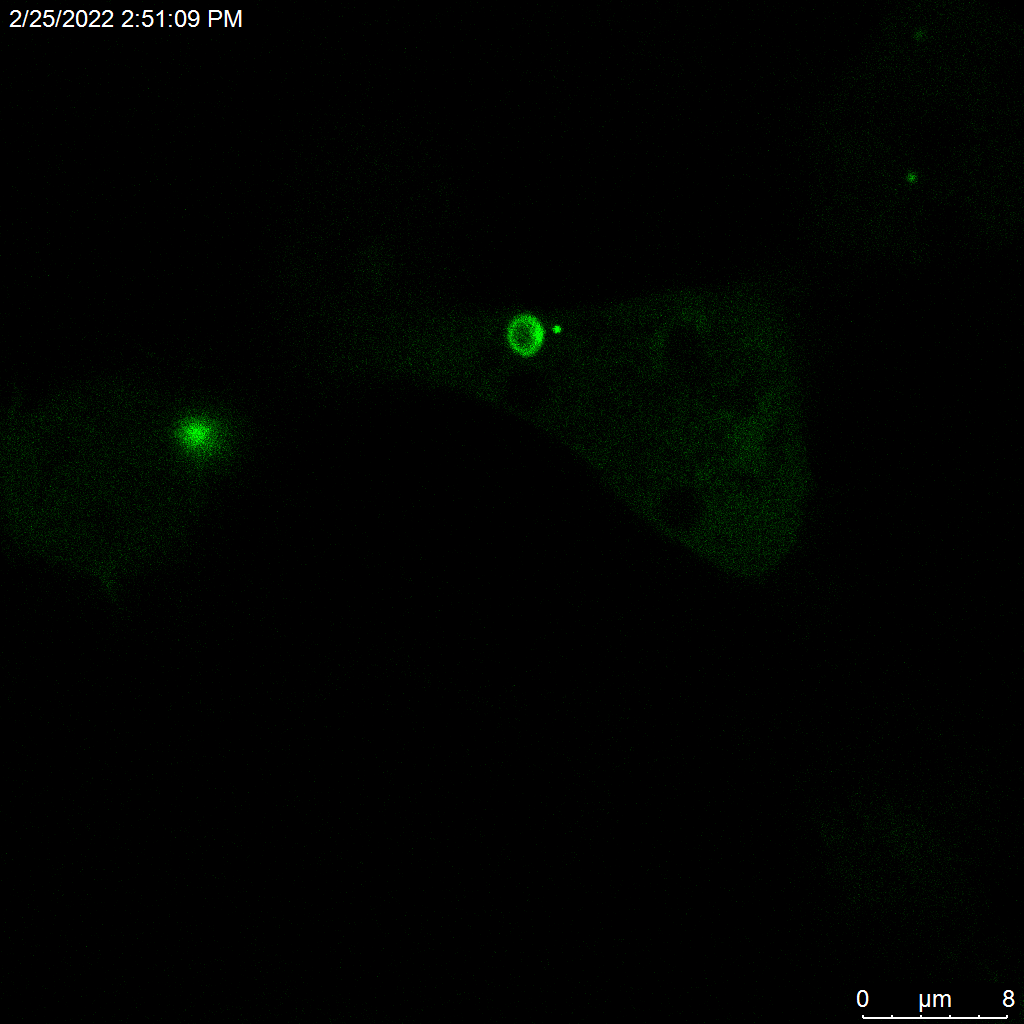

Supplement: Supplementary file 6 — Source data Fig. 3 [file 44319_2025_485_MOESM6_ESM.zip › Figure 3/3F/Fig. 3F_015.tif]

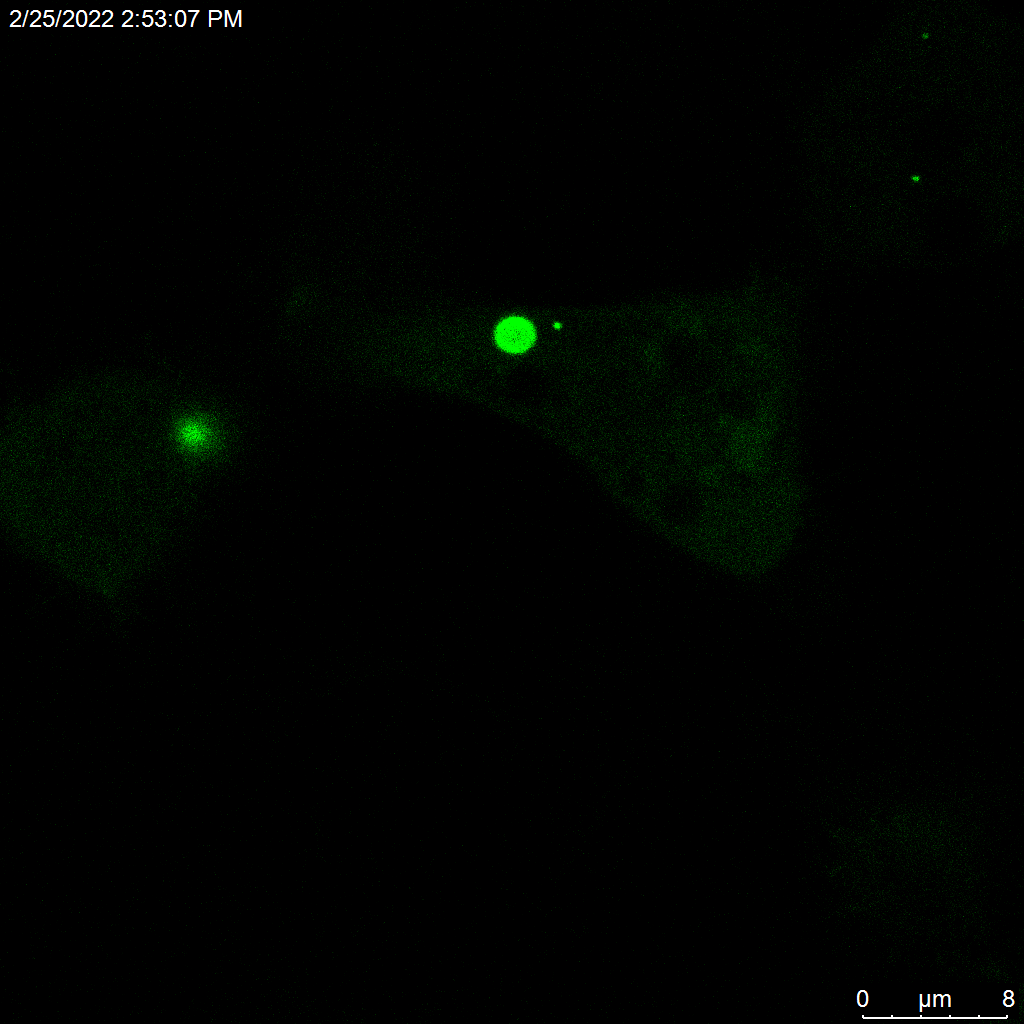

Supplement: Supplementary file 6 — Source data Fig. 3 [file 44319_2025_485_MOESM6_ESM.zip › Figure 3/3F/Fig. 3F_130s.tif]

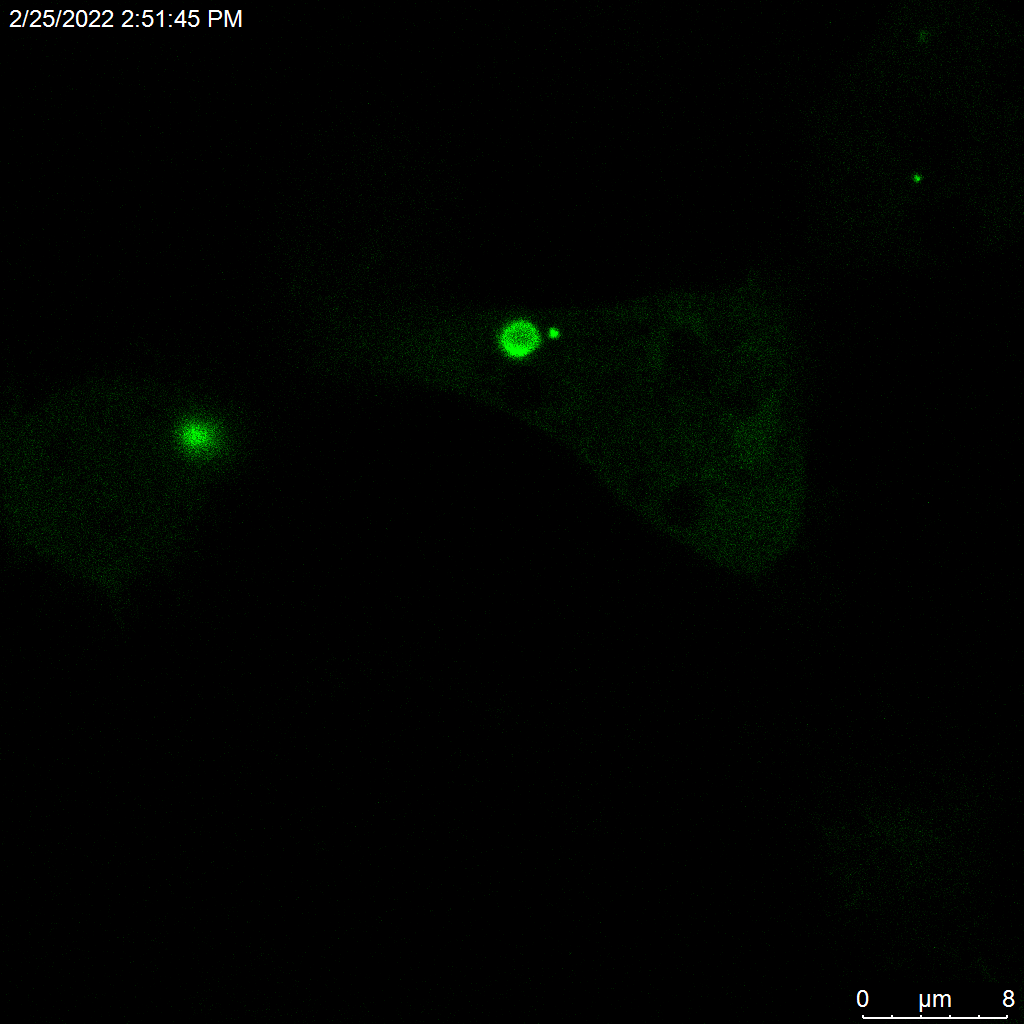

Supplement: Supplementary file 6 — Source data Fig. 3 [file 44319_2025_485_MOESM6_ESM.zip › Figure 3/3F/Fig. 3F_50s.tif]

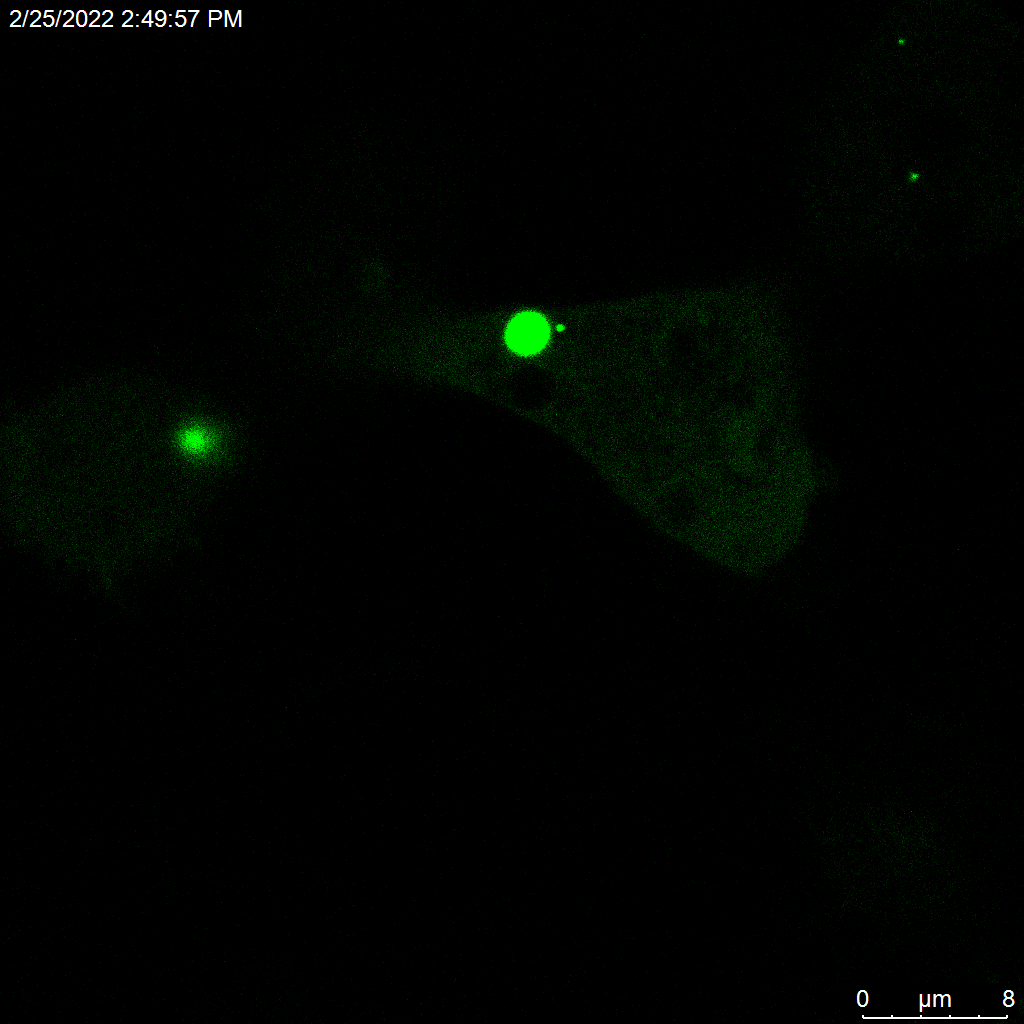

Supplement: Supplementary file 6 — Source data Fig. 3 [file 44319_2025_485_MOESM6_ESM.zip › Figure 3/3F/Fig. 3F_minus60s.tif]

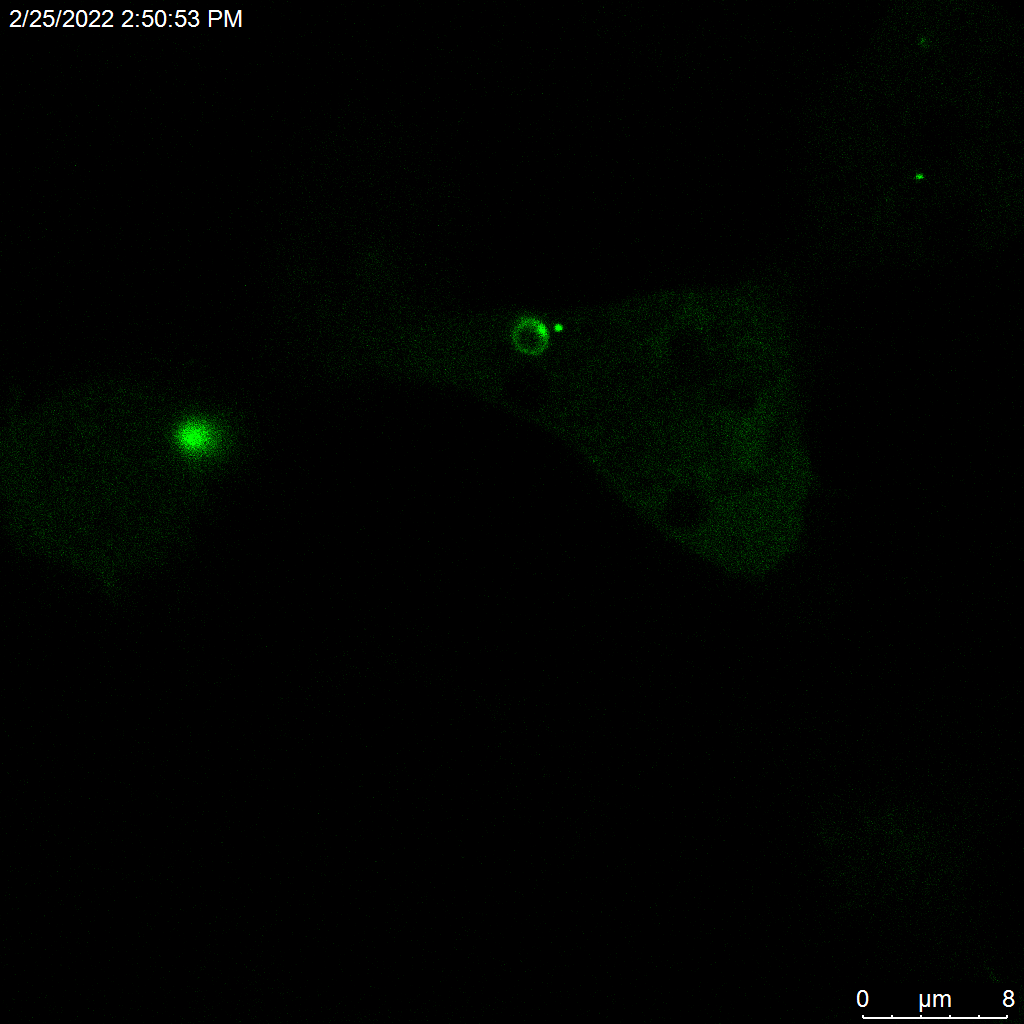

Supplement: Supplementary file 6 — Source data Fig. 3 [file 44319_2025_485_MOESM6_ESM.zip › Figure 3/3F/Fig. 3F_0s.tif]

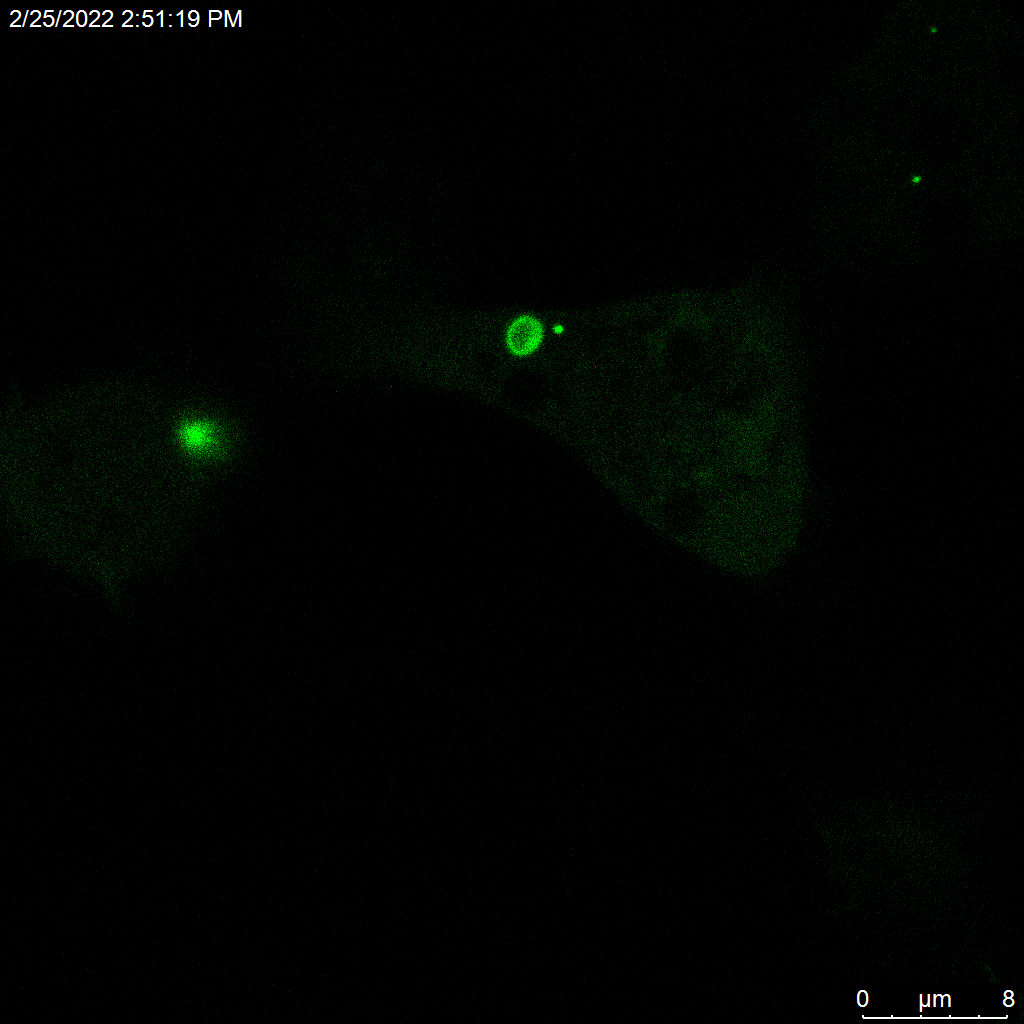

Supplement: Supplementary file 6 — Source data Fig. 3 [file 44319_2025_485_MOESM6_ESM.zip › Figure 3/3F/Fig. 3F_25s.tif]

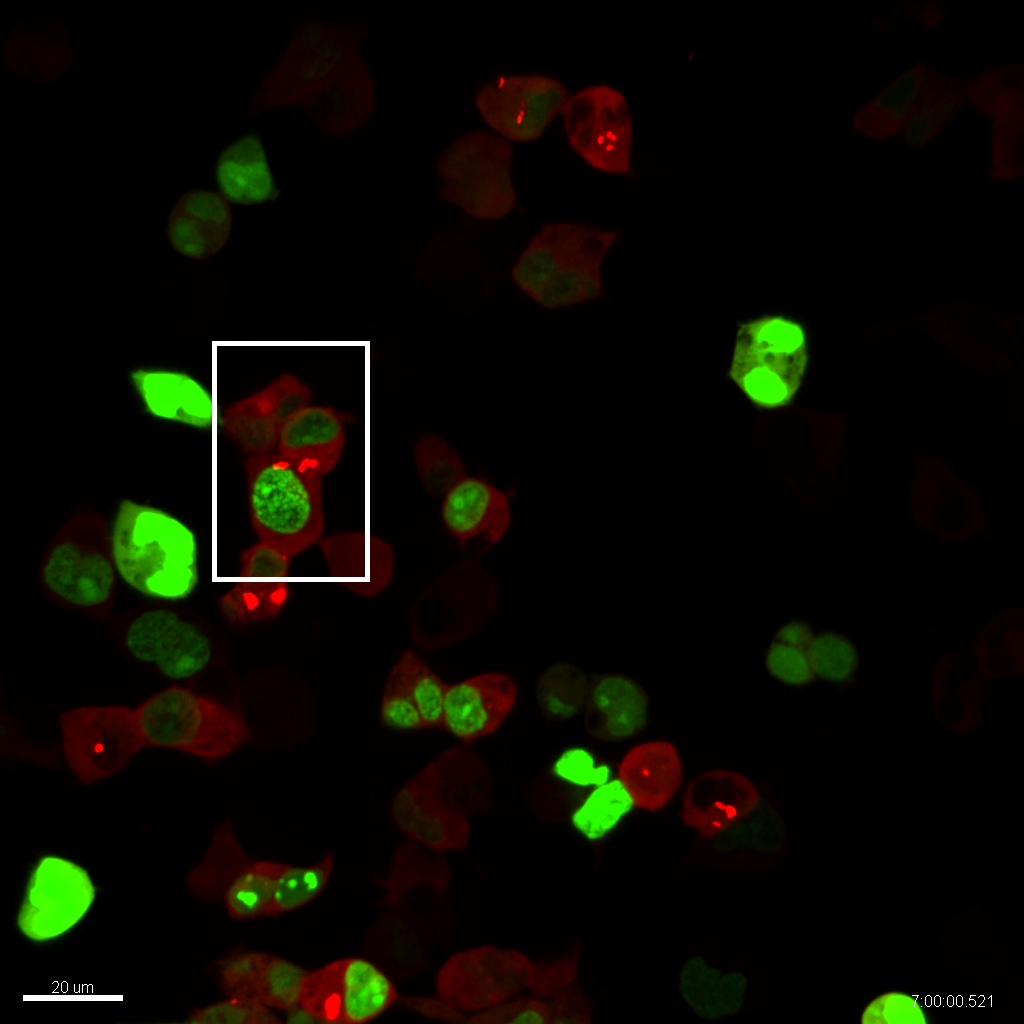

Supplement: Supplementary file 6 — Source data Fig. 3 [file 44319_2025_485_MOESM6_ESM.zip › Figure 3/3I/Fig. 3I_0S.tif]

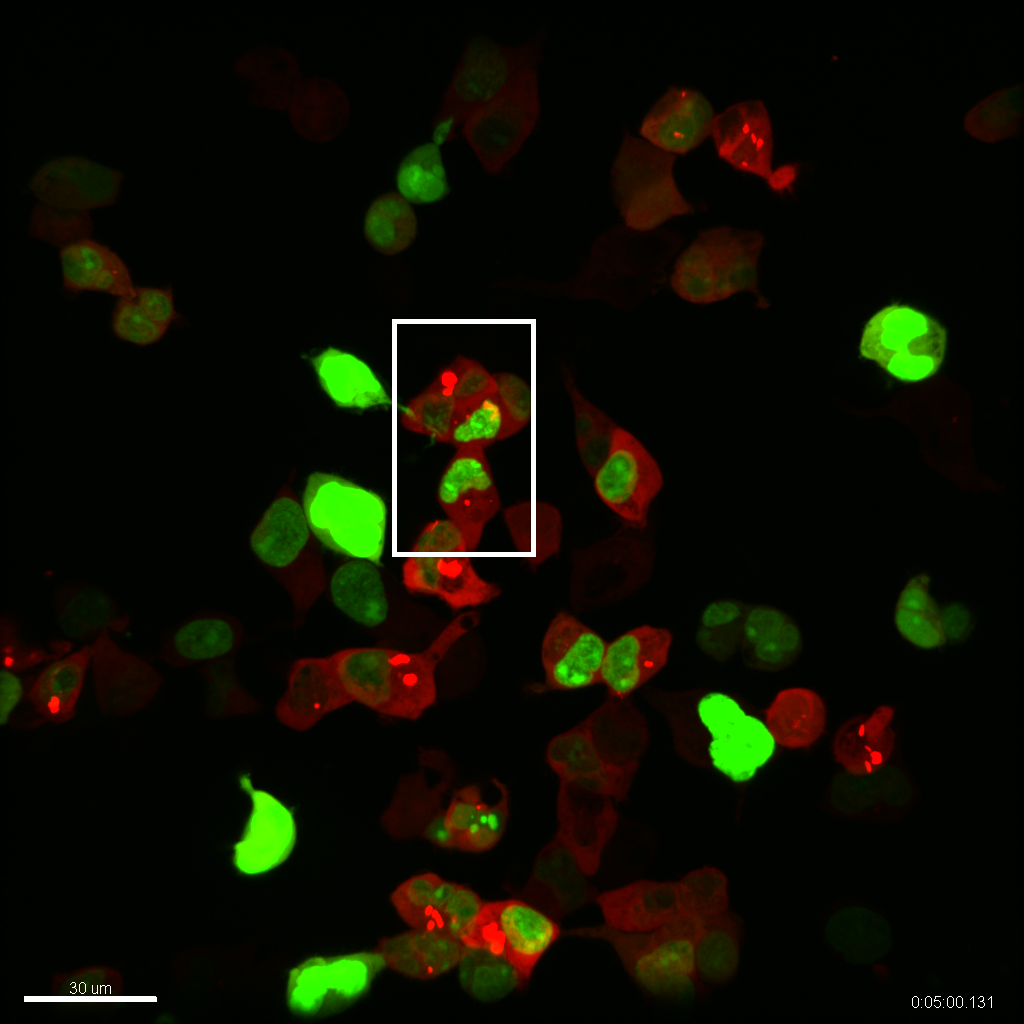

Supplement: Supplementary file 6 — Source data Fig. 3 [file 44319_2025_485_MOESM6_ESM.zip › Figure 3/3I/Fig. 3I_125S.tif]

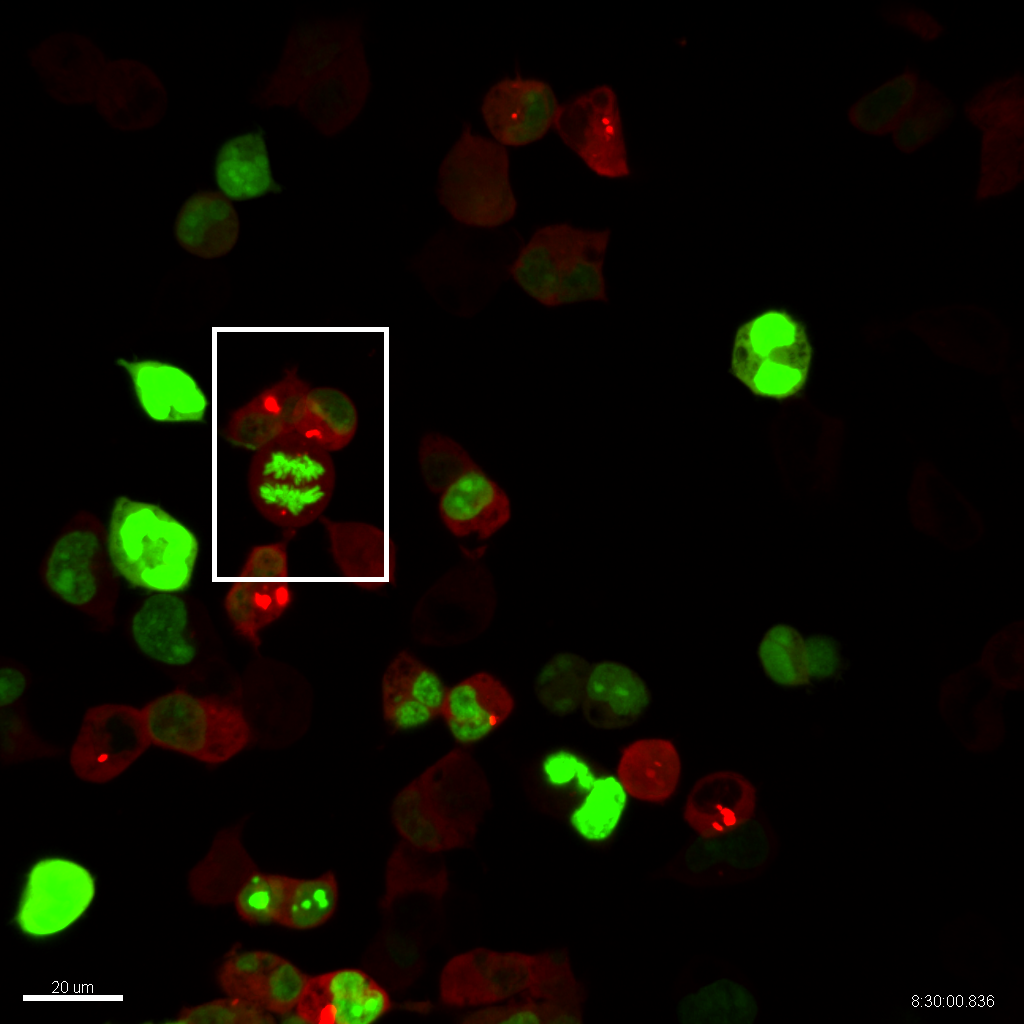

Supplement: Supplementary file 6 — Source data Fig. 3 [file 44319_2025_485_MOESM6_ESM.zip › Figure 3/3I/Fig. 3I_90S.tif]

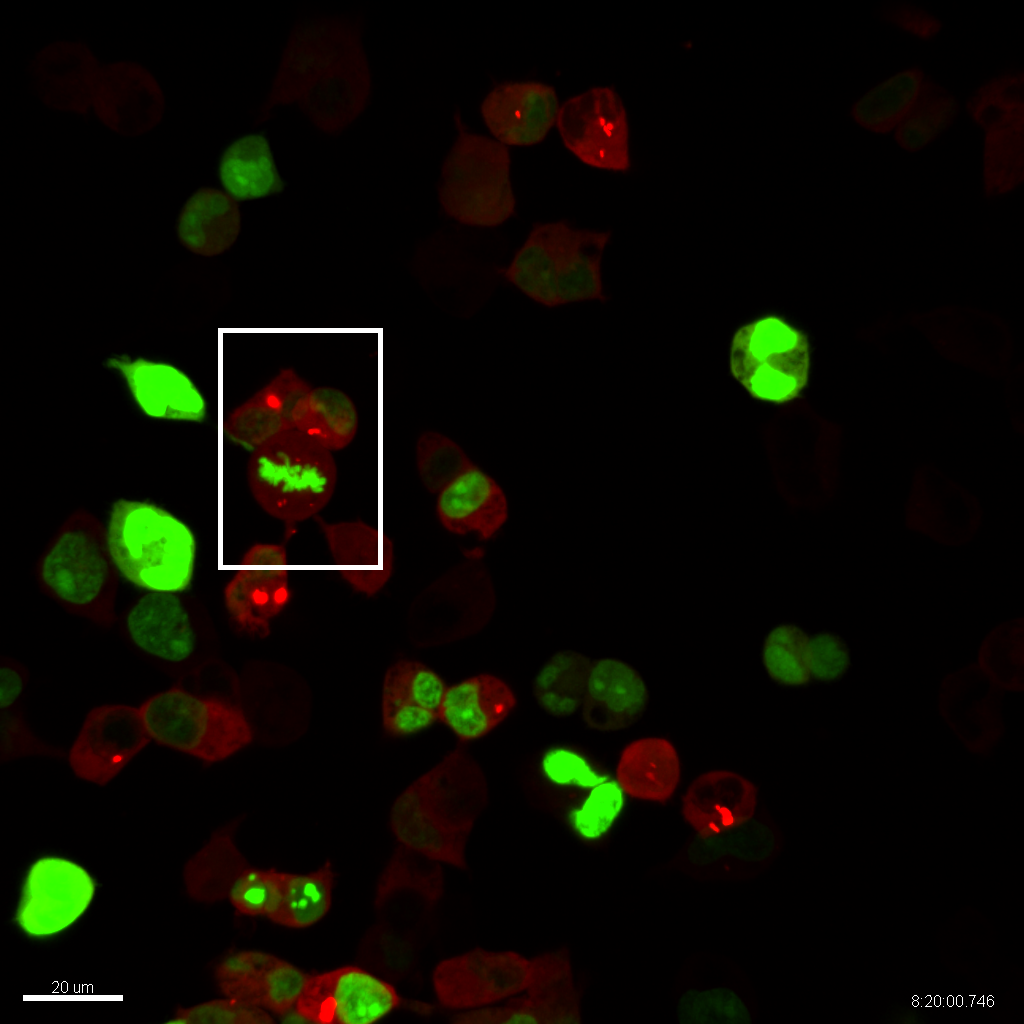

Supplement: Supplementary file 6 — Source data Fig. 3 [file 44319_2025_485_MOESM6_ESM.zip › Figure 3/3I/Fig. 3I_80S.tif]

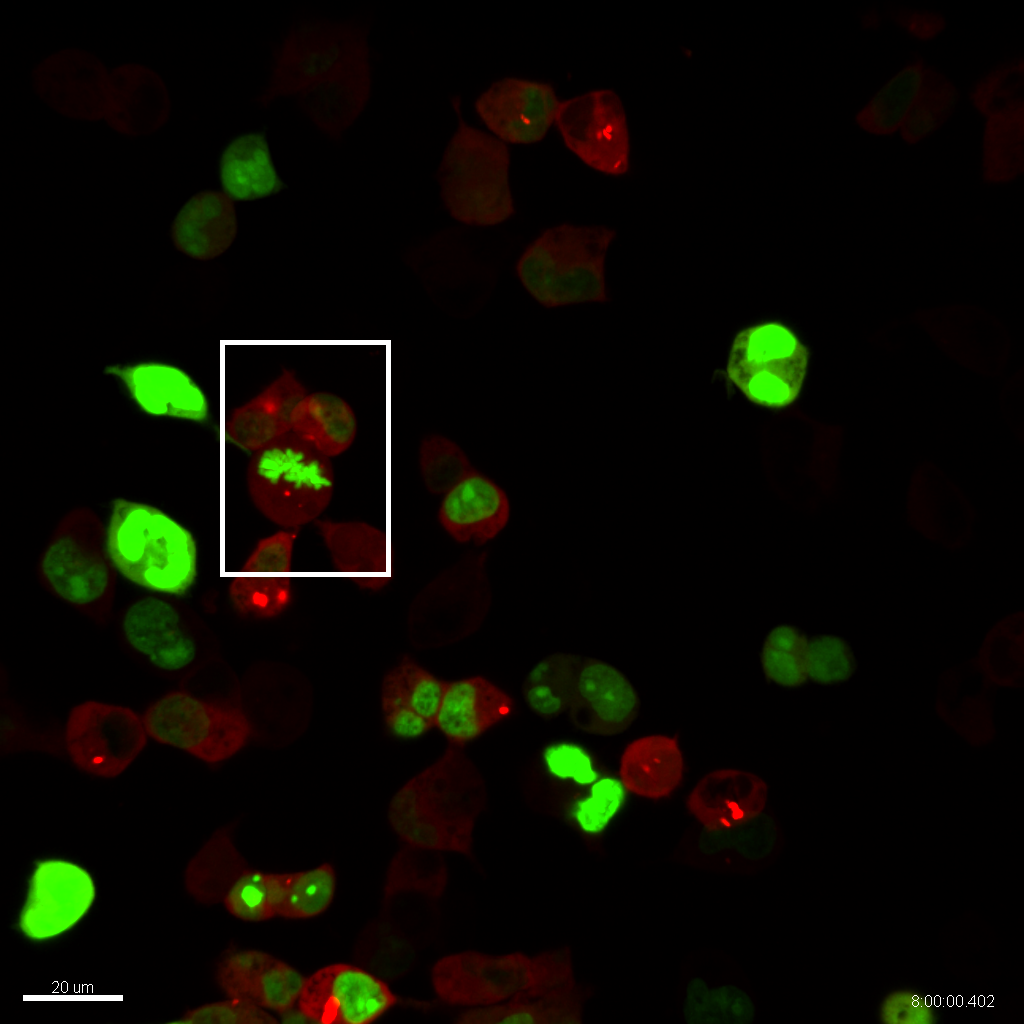

Supplement: Supplementary file 6 — Source data Fig. 3 [file 44319_2025_485_MOESM6_ESM.zip › Figure 3/3I/Fig. 3I_60S.tif]

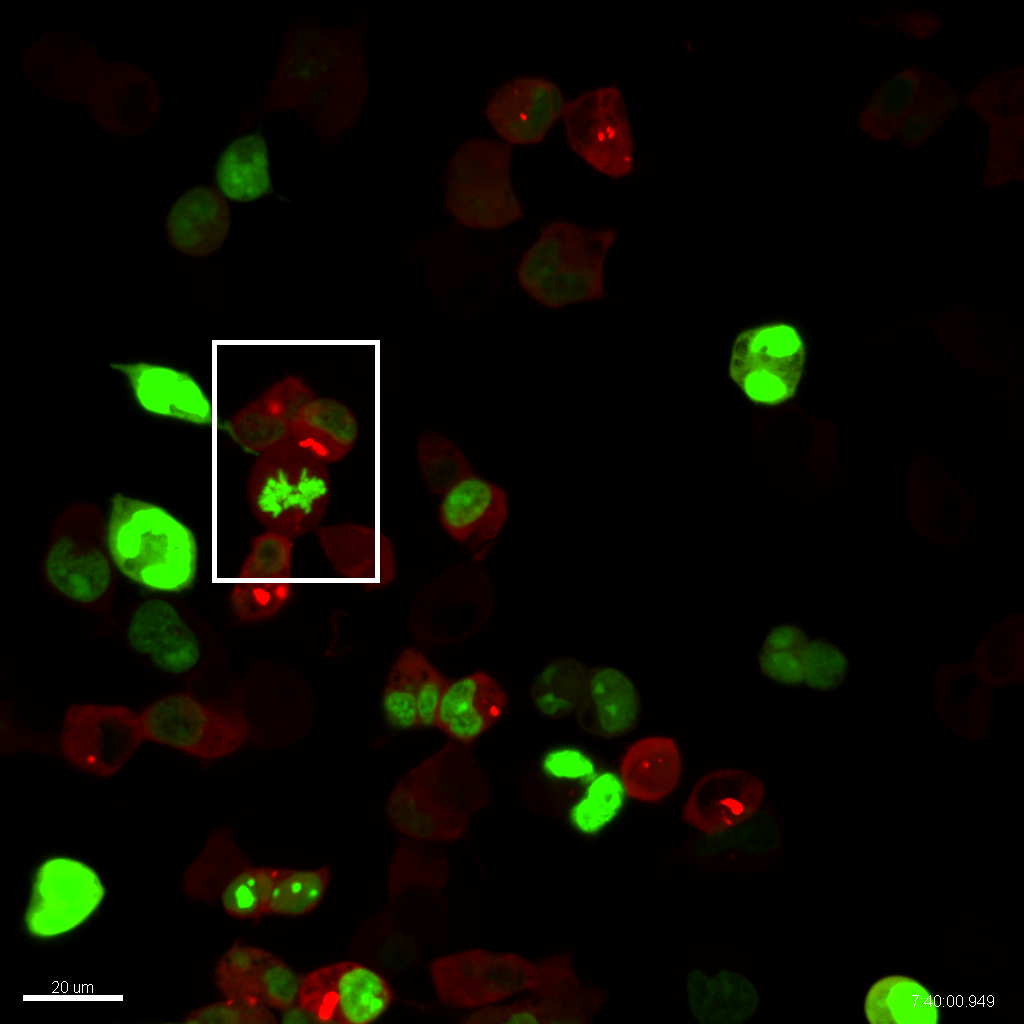

Supplement: Supplementary file 6 — Source data Fig. 3 [file 44319_2025_485_MOESM6_ESM.zip › Figure 3/3I/Fig. 3I_40S.tif]

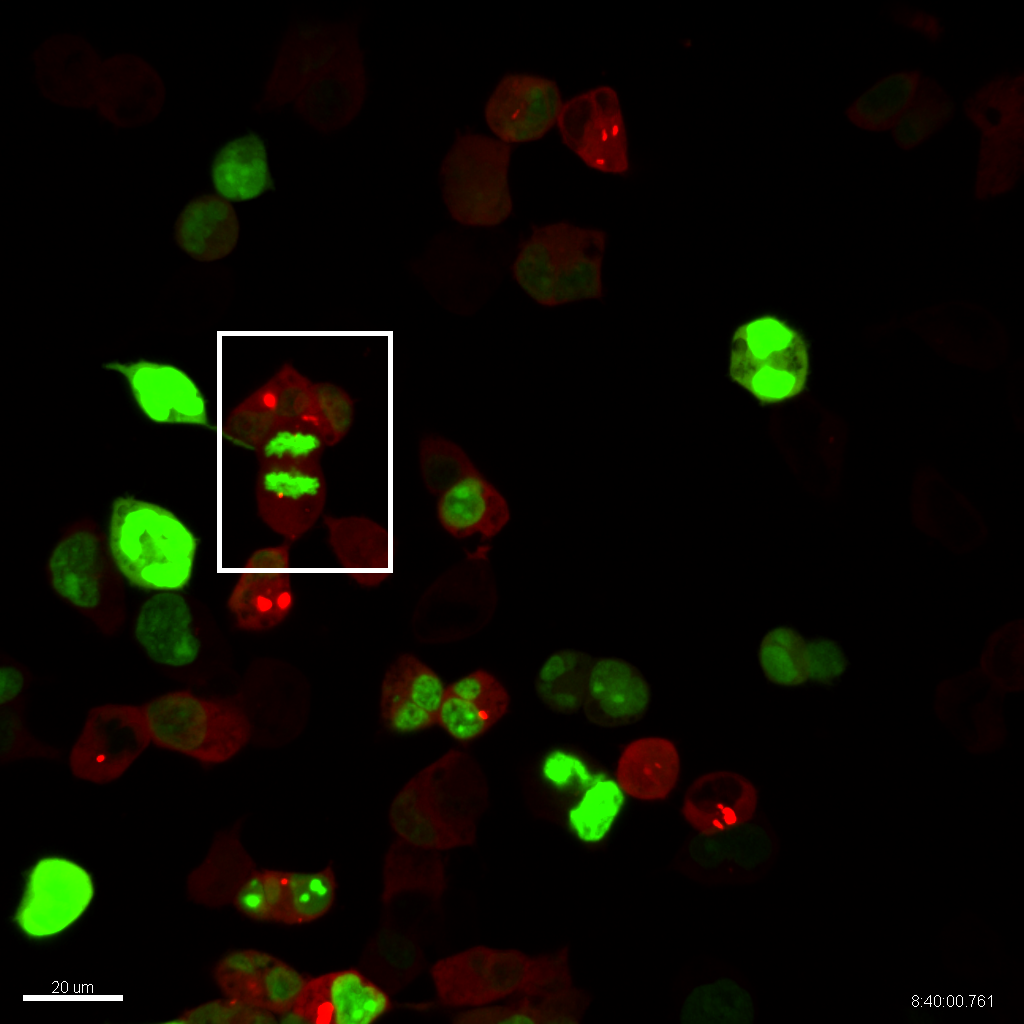

Supplement: Supplementary file 6 — Source data Fig. 3 [file 44319_2025_485_MOESM6_ESM.zip › Figure 3/3I/Fig. 3I_95S.tif]

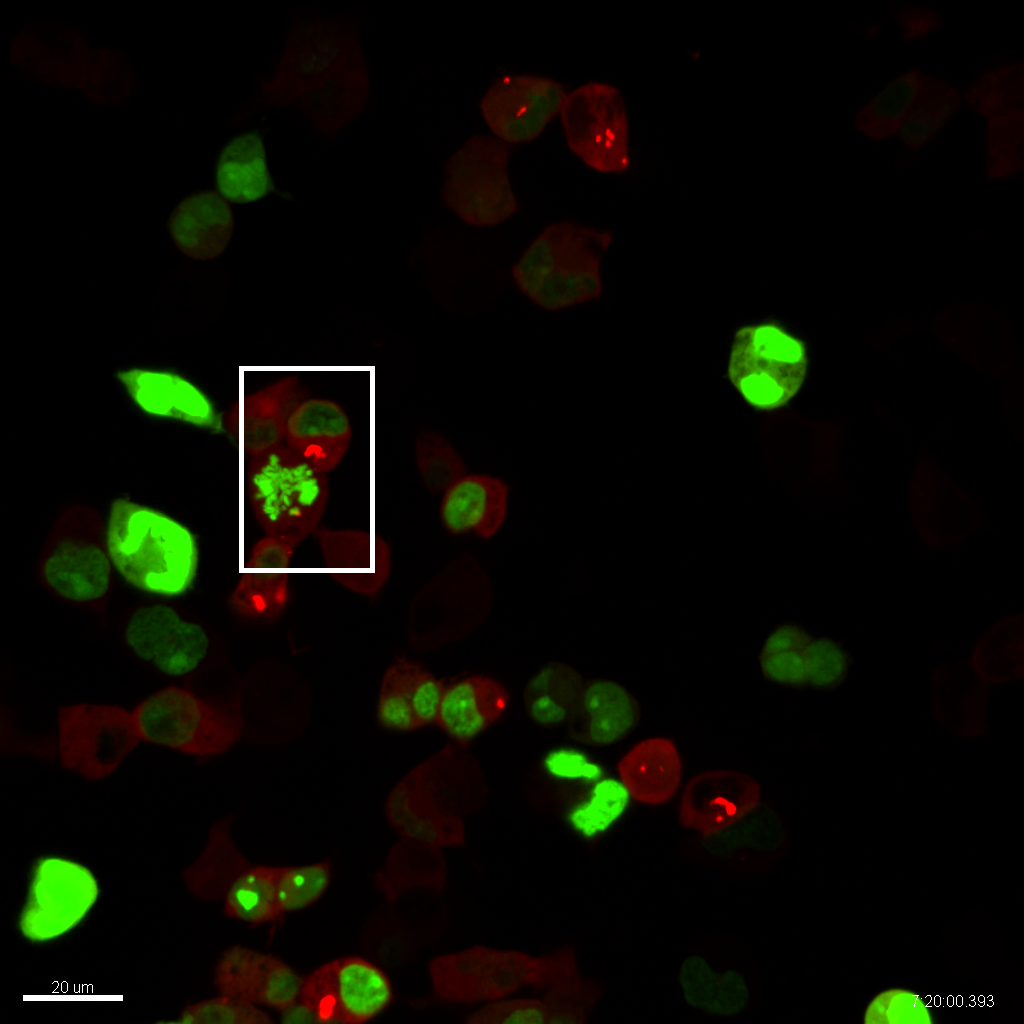

Supplement: Supplementary file 6 — Source data Fig. 3 [file 44319_2025_485_MOESM6_ESM.zip › Figure 3/3I/Fig. 3I_20S.tif]

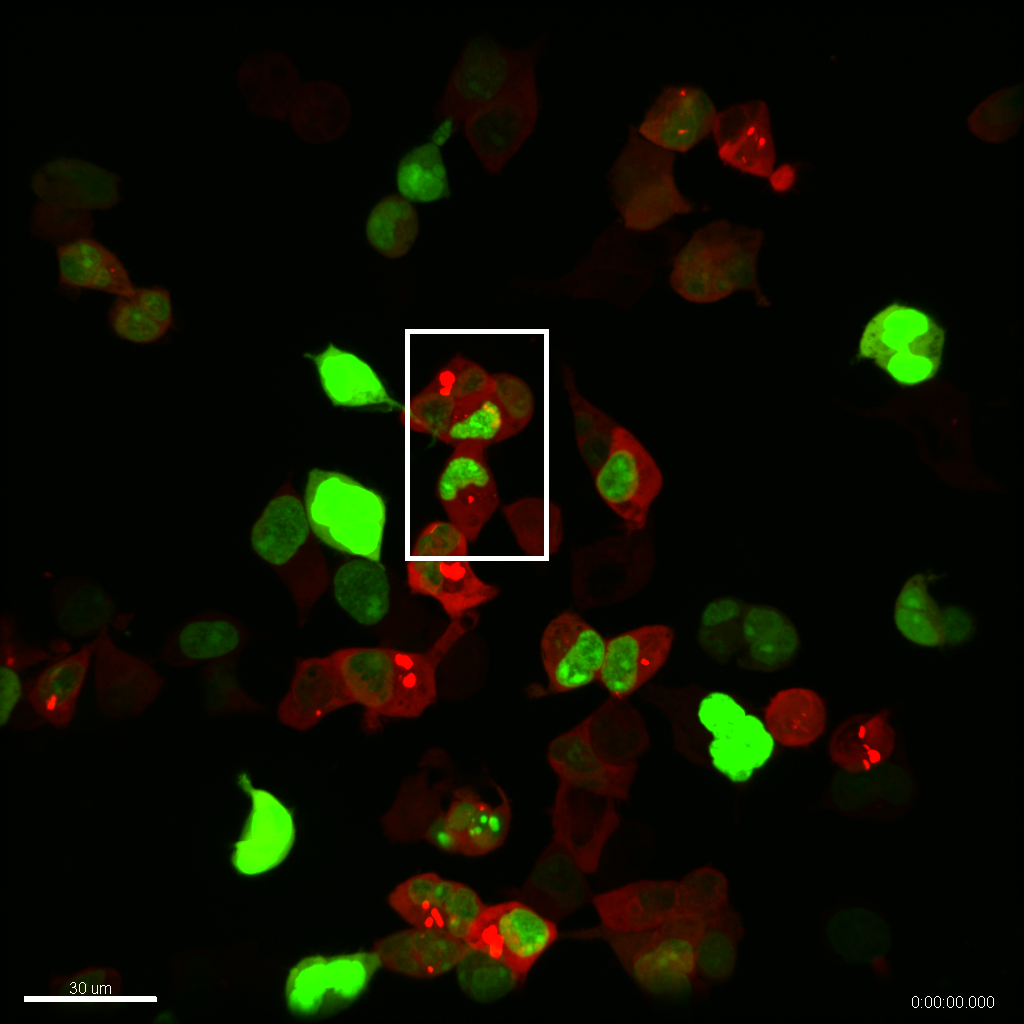

Supplement: Supplementary file 6 — Source data Fig. 3 [file 44319_2025_485_MOESM6_ESM.zip › Figure 3/3I/Fig. 3I_120S.tif]

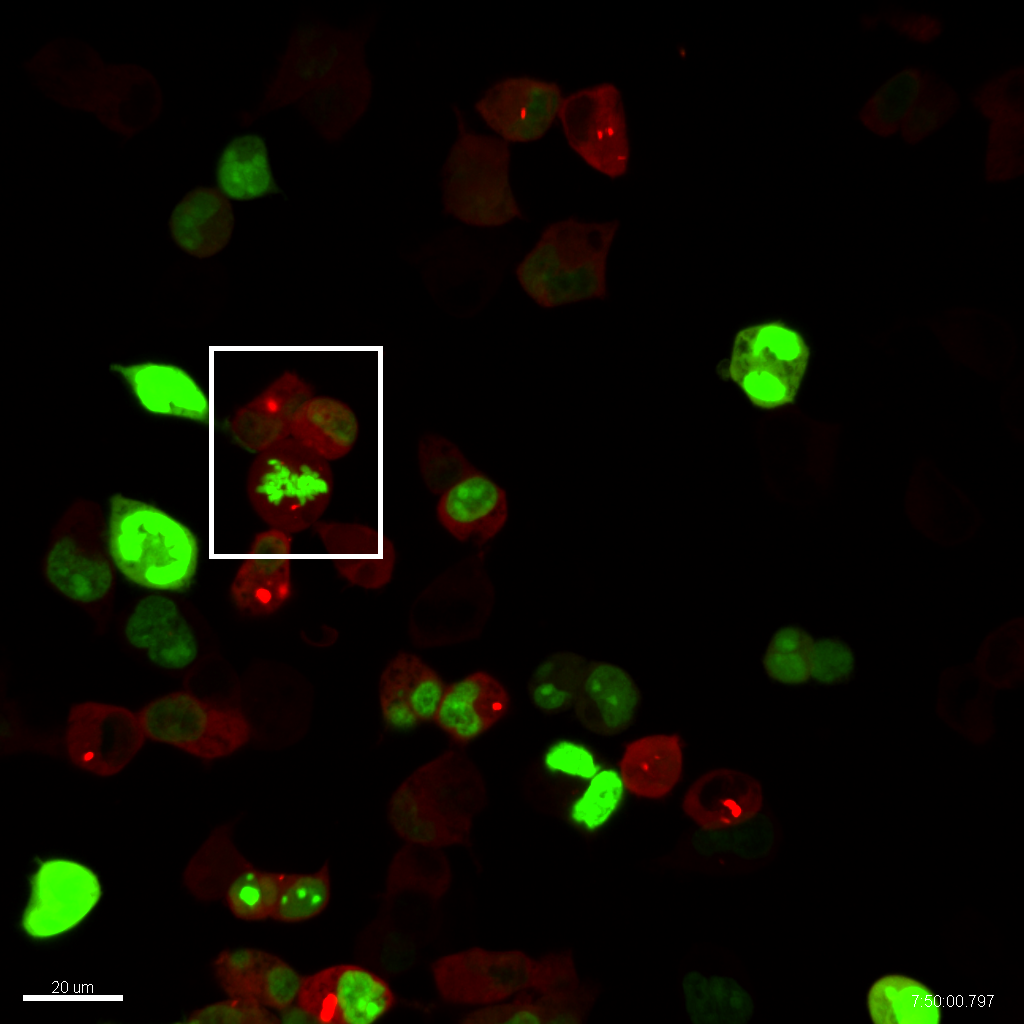

Supplement: Supplementary file 6 — Source data Fig. 3 [file 44319_2025_485_MOESM6_ESM.zip › Figure 3/3I/Fig. 3I_50S.tif]

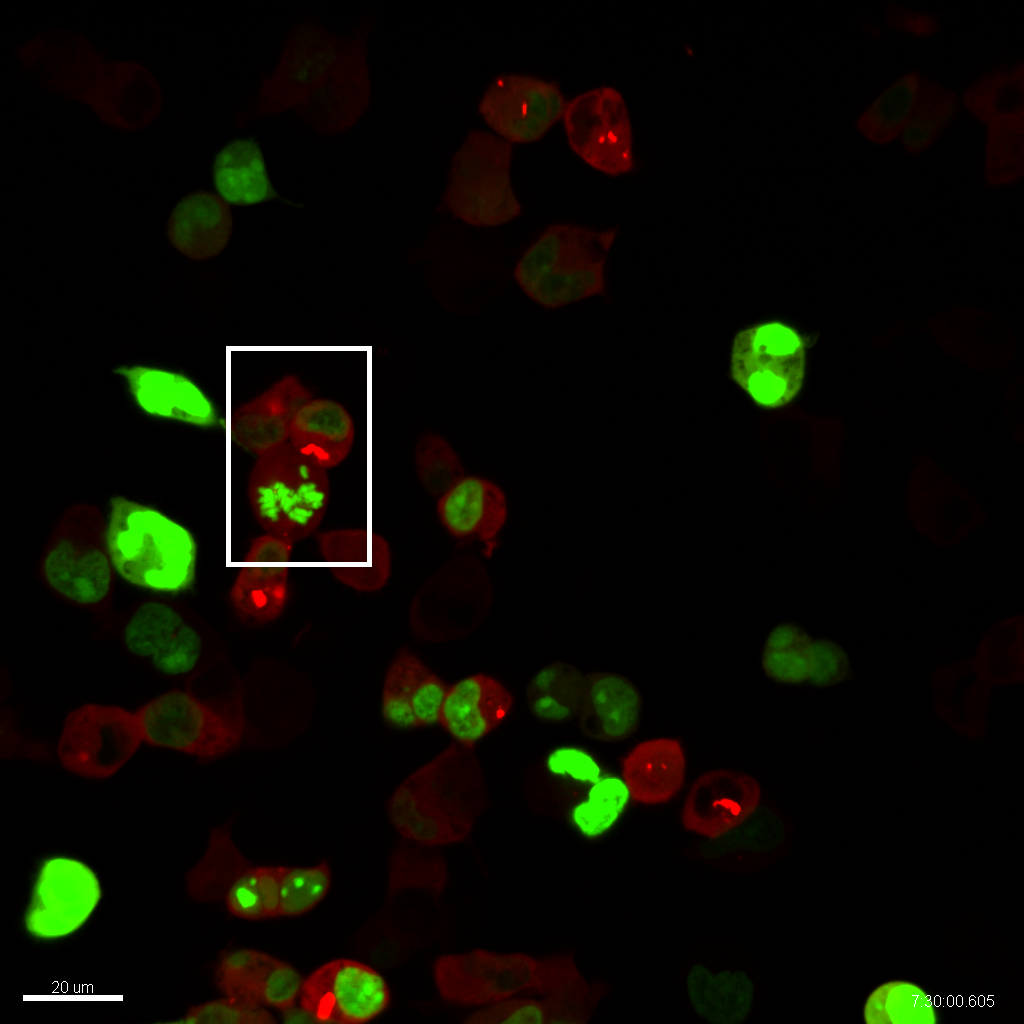

Supplement: Supplementary file 6 — Source data Fig. 3 [file 44319_2025_485_MOESM6_ESM.zip › Figure 3/3I/Fig. 3I_30S.tif]

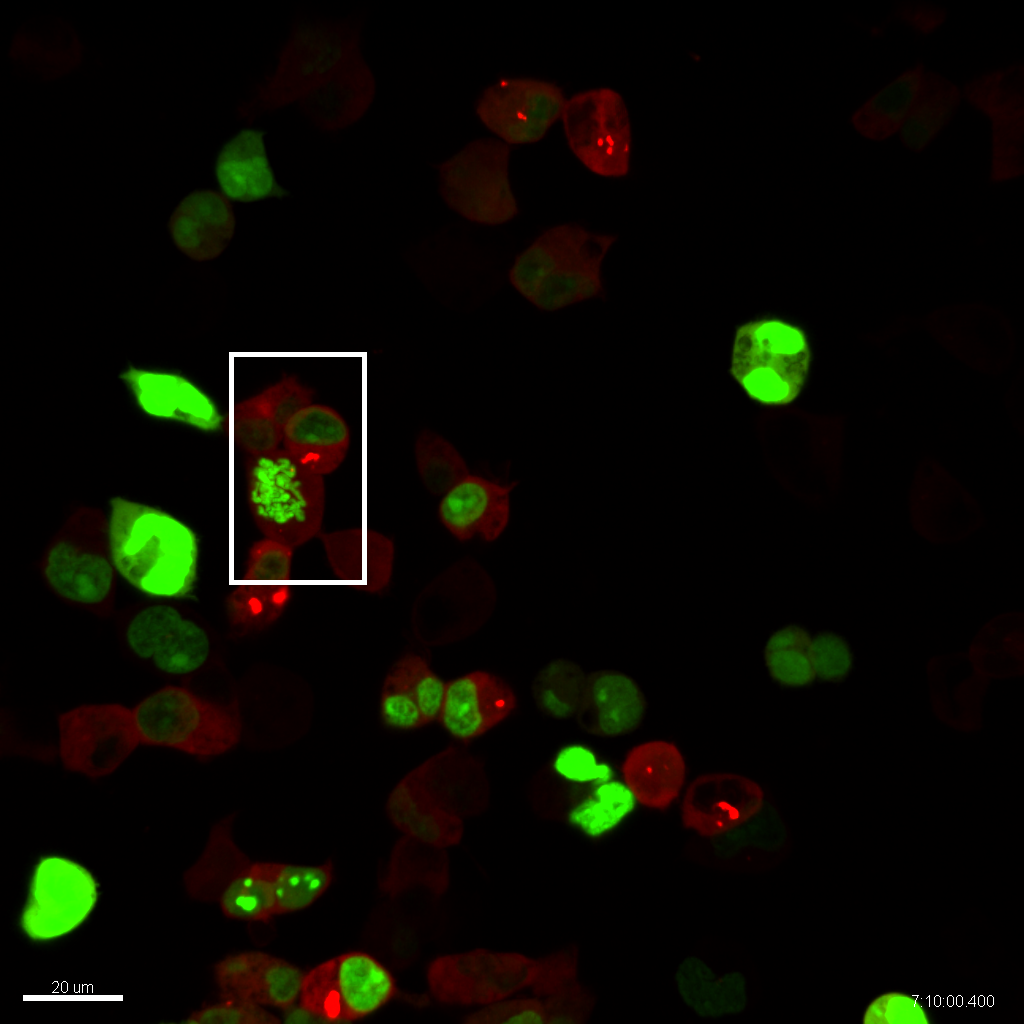

Supplement: Supplementary file 6 — Source data Fig. 3 [file 44319_2025_485_MOESM6_ESM.zip › Figure 3/3I/Fig. 3I_10S.tif]

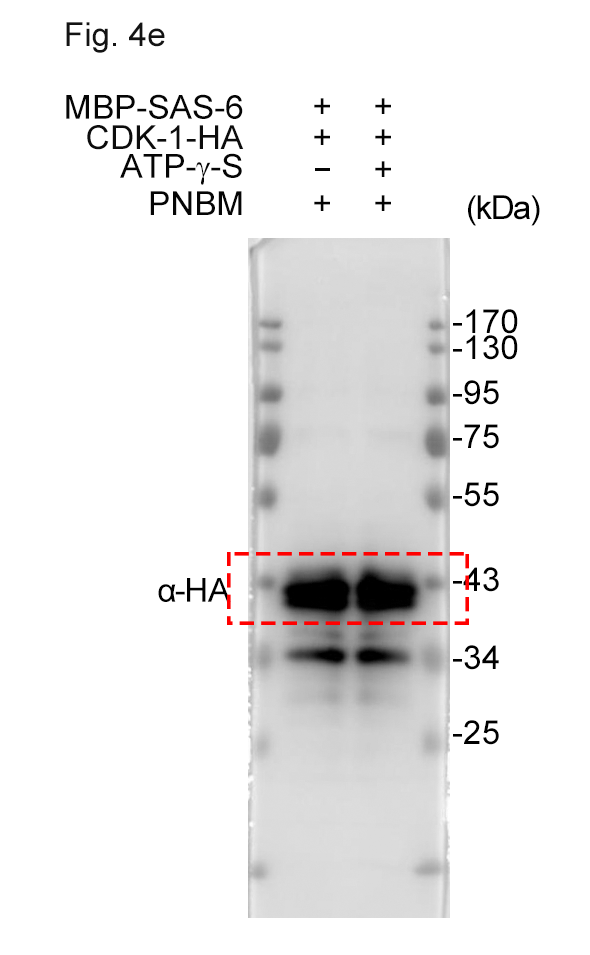

Supplement: Supplementary file 7 — Source data Fig. 4 [file 44319_2025_485_MOESM7_ESM.zip › Figure 4/4E/Fig. 4E_HA.tif]

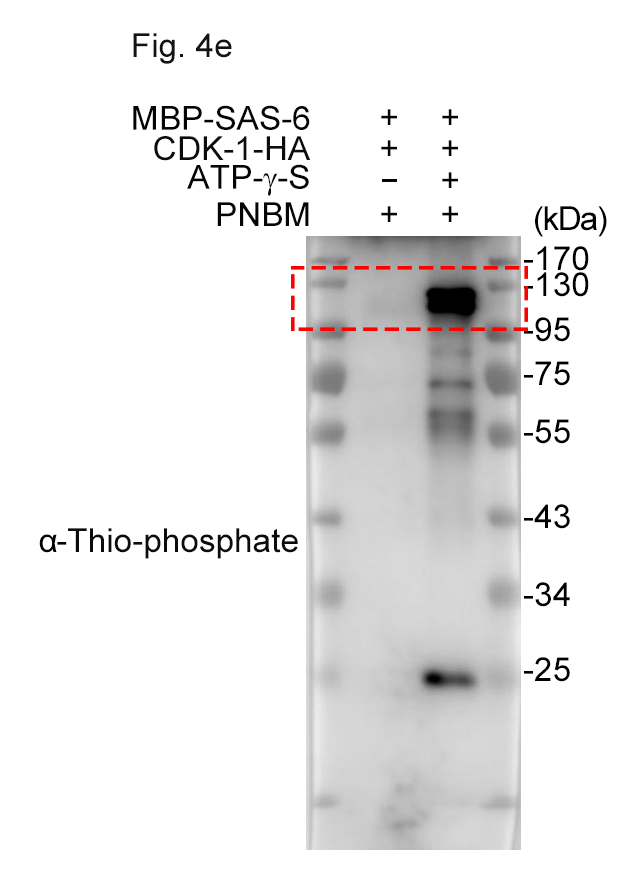

Supplement: Supplementary file 7 — Source data Fig. 4 [file 44319_2025_485_MOESM7_ESM.zip › Figure 4/4E/Fig. 4E_Thio.tif]

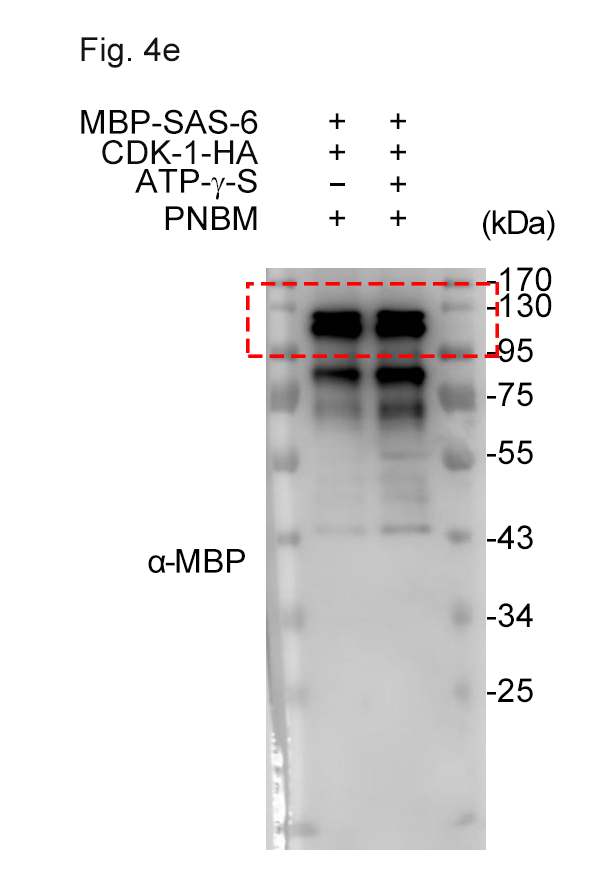

Supplement: Supplementary file 7 — Source data Fig. 4 [file 44319_2025_485_MOESM7_ESM.zip › Figure 4/4E/Fig. 4E_MBP.tif]

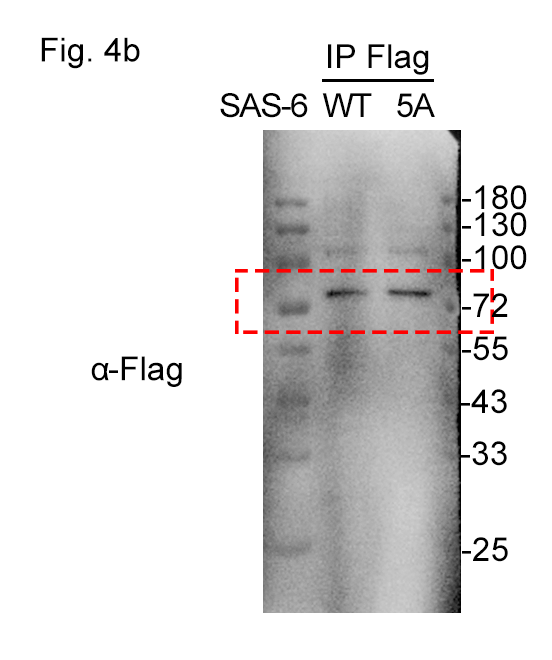

Supplement: Supplementary file 7 — Source data Fig. 4 [file 44319_2025_485_MOESM7_ESM.zip › Figure 4/4B/Fig. 4B_Flag.tif]

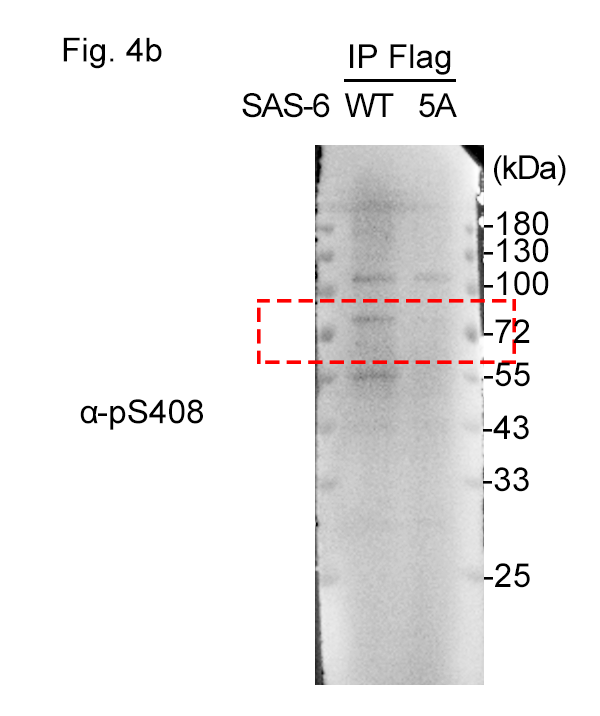

Supplement: Supplementary file 7 — Source data Fig. 4 [file 44319_2025_485_MOESM7_ESM.zip › Figure 4/4B/Fig. 4B_pS408.tif]

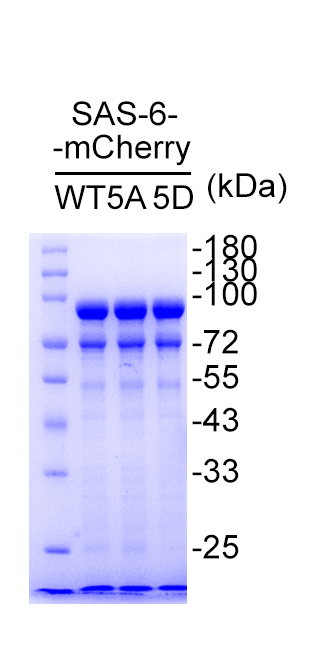

Supplement: Supplementary file 7 — Source data Fig. 4 [file 44319_2025_485_MOESM7_ESM.zip › Figure 4/4K/Fig. 4K_SAS6_FL_mCherry.tif]

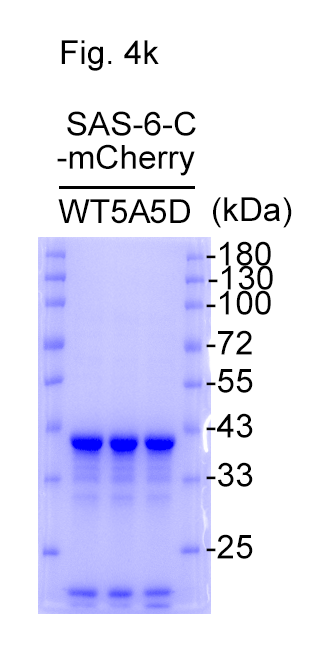

Supplement: Supplementary file 7 — Source data Fig. 4 [file 44319_2025_485_MOESM7_ESM.zip › Figure 4/4K/Fig. 4K_SAS6_C_mCherry.tif]

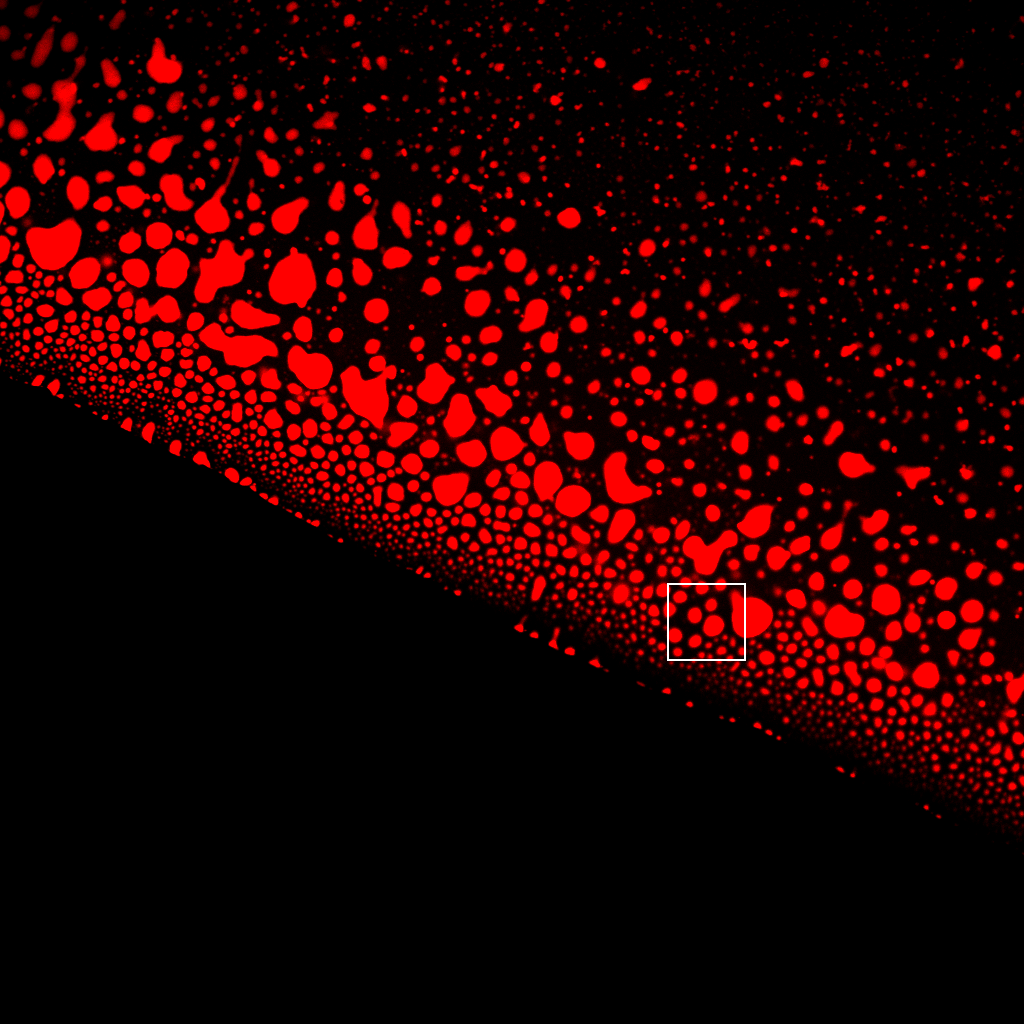

Supplement: Supplementary file 7 — Source data Fig. 4 [file 44319_2025_485_MOESM7_ESM.zip › Figure 4/4L/Fig. 4L_WT_50'.tif]

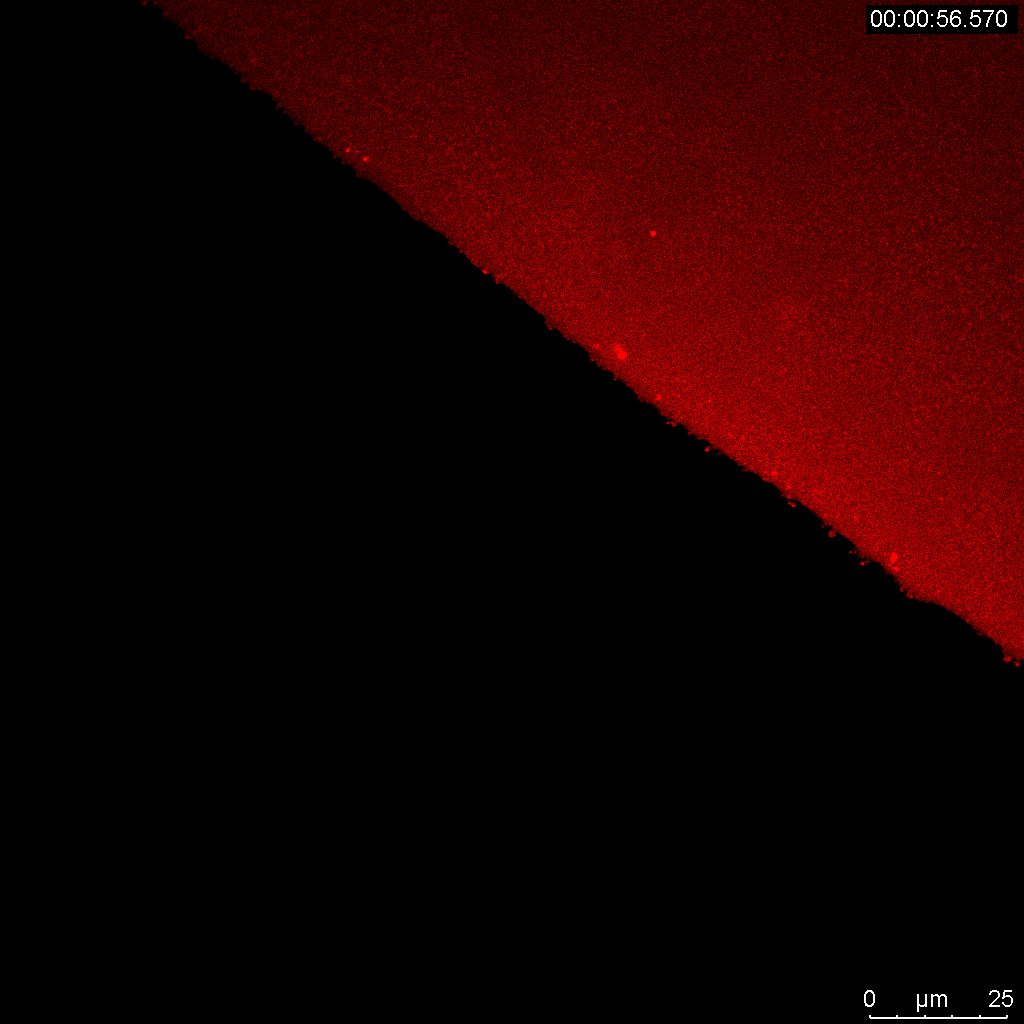

Supplement: Supplementary file 7 — Source data Fig. 4 [file 44319_2025_485_MOESM7_ESM.zip › Figure 4/4L/Fig. 4L_5D_60''.tif]

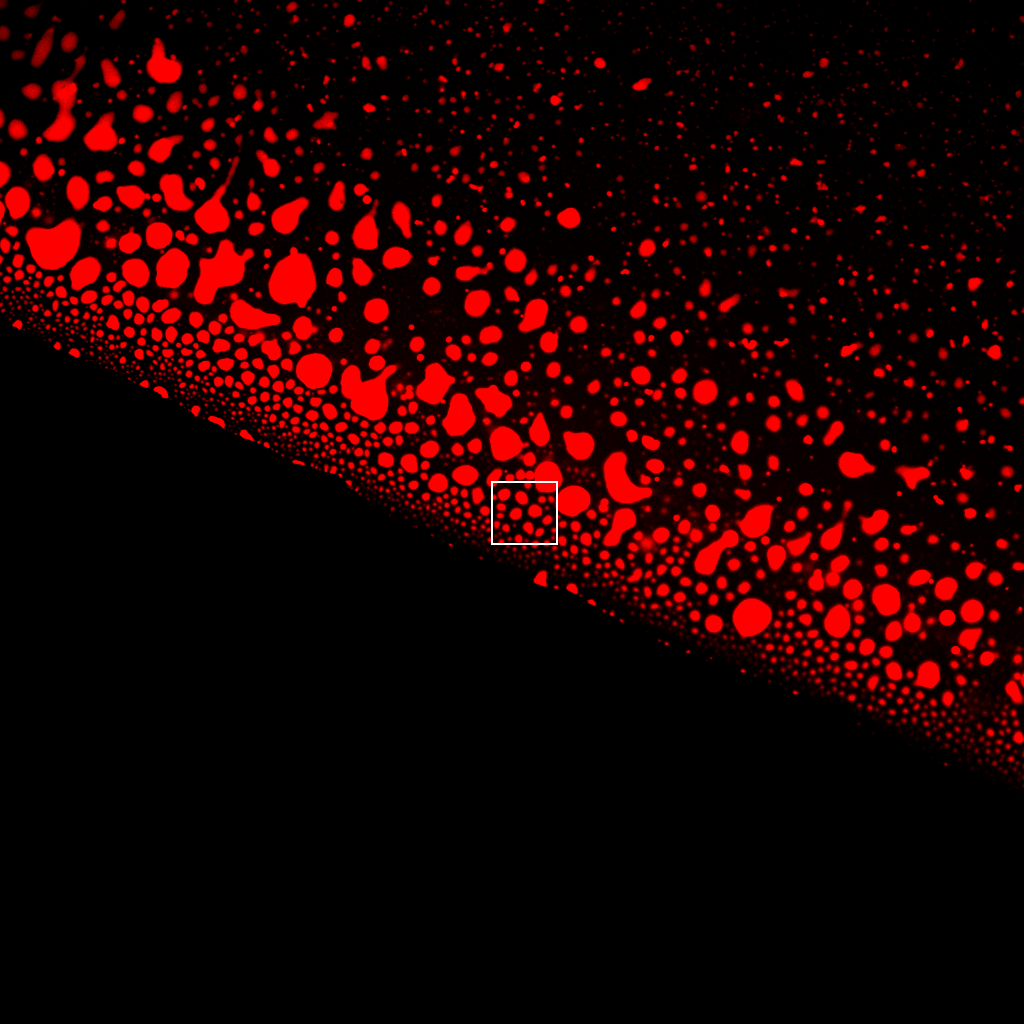

Supplement: Supplementary file 7 — Source data Fig. 4 [file 44319_2025_485_MOESM7_ESM.zip › Figure 4/4L/Fig. 4L_WT_30'.tif]

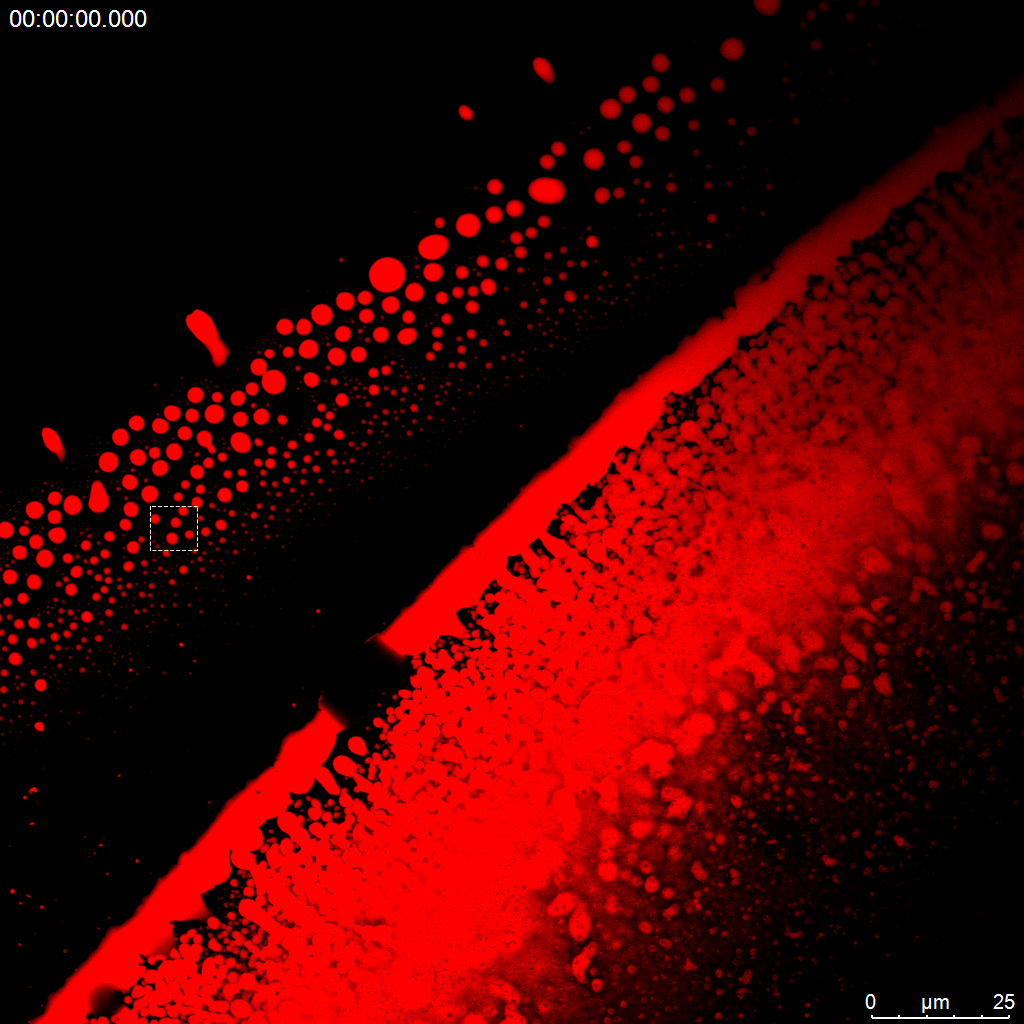

Supplement: Supplementary file 7 — Source data Fig. 4 [file 44319_2025_485_MOESM7_ESM.zip › Figure 4/4L/Fig. 4L_5A_0''.tif]

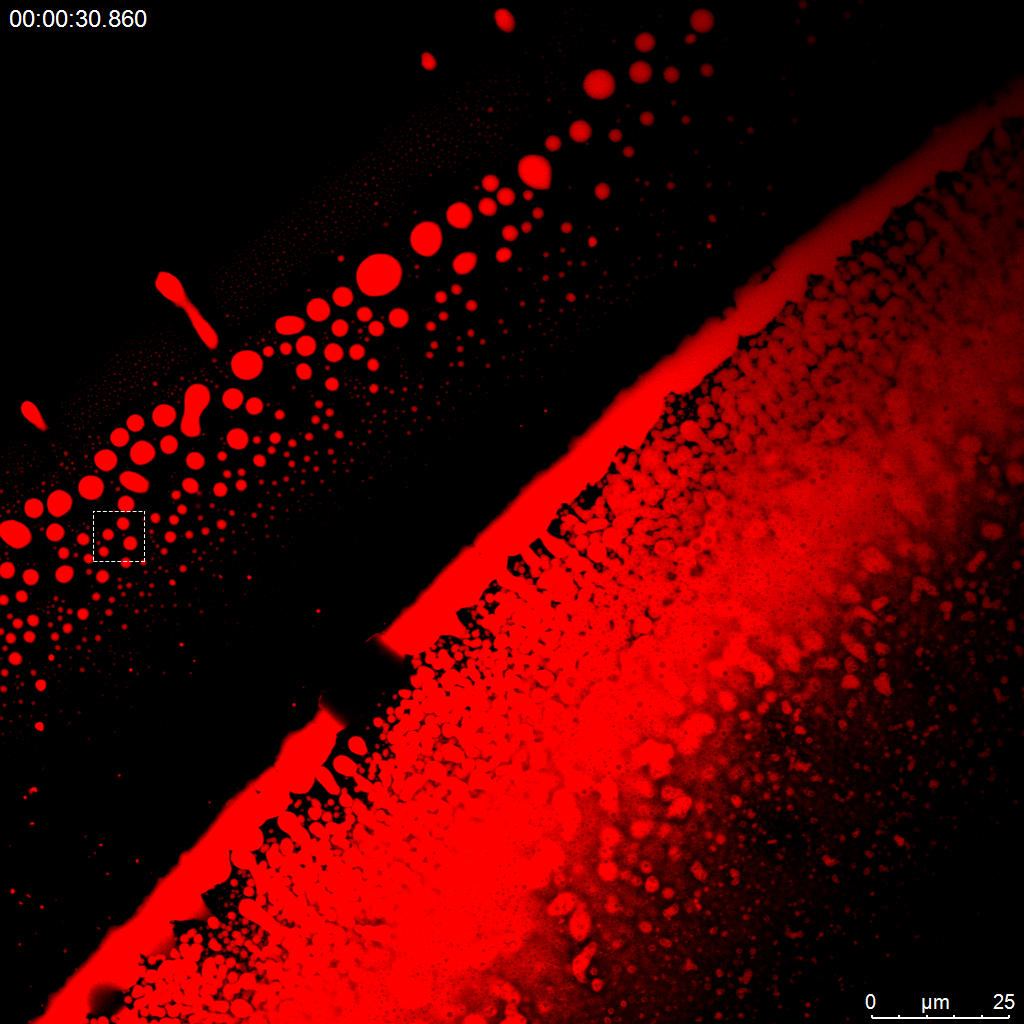

Supplement: Supplementary file 7 — Source data Fig. 4 [file 44319_2025_485_MOESM7_ESM.zip › Figure 4/4L/Fig. 4L_5A_30''.tif]

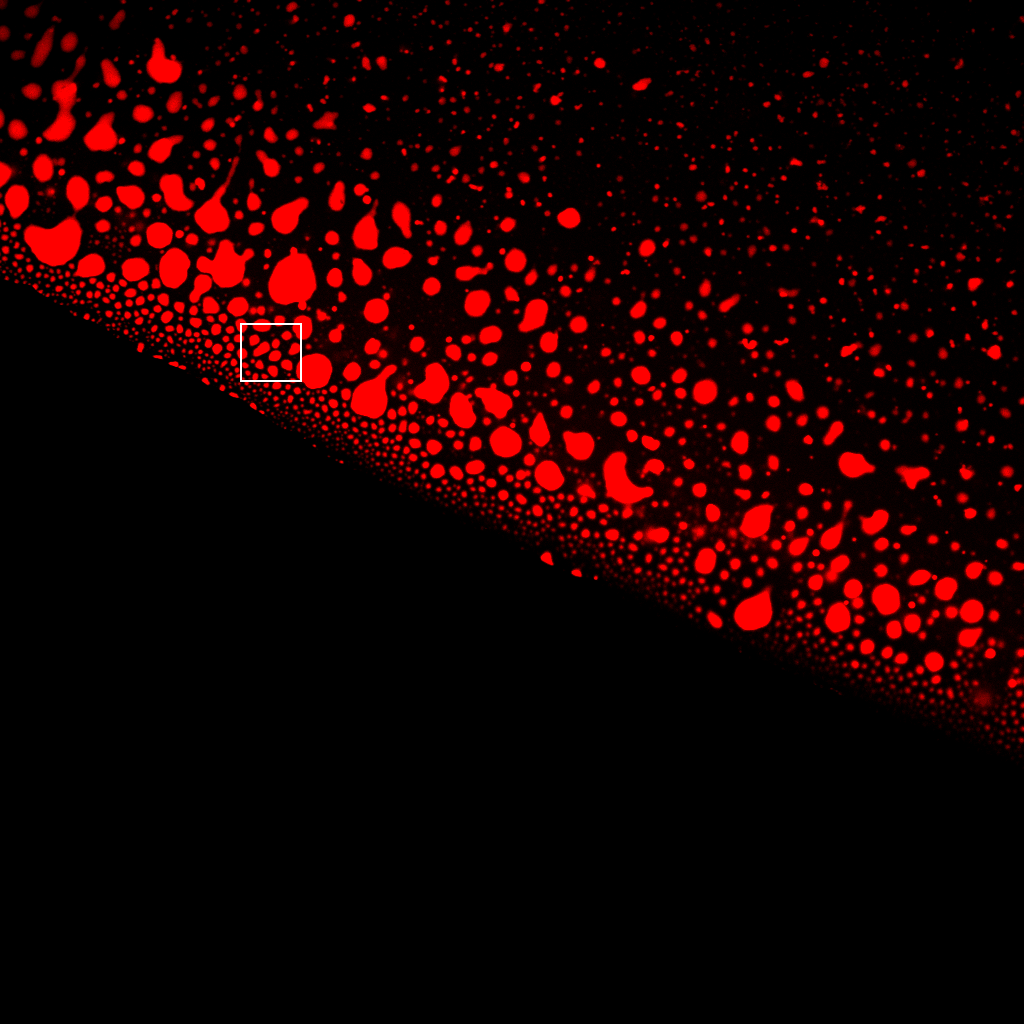

Supplement: Supplementary file 7 — Source data Fig. 4 [file 44319_2025_485_MOESM7_ESM.zip › Figure 4/4L/Fig. 4L_WT_0''.tif]

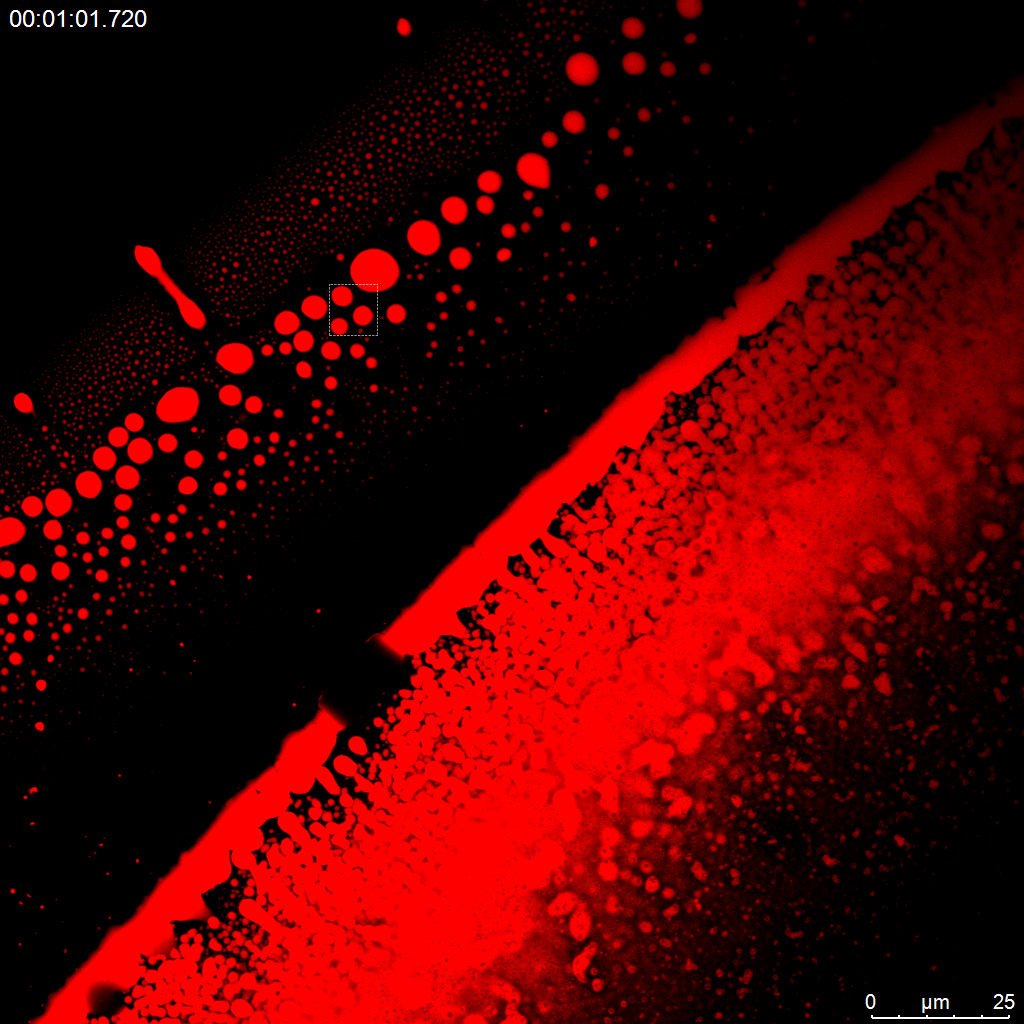

Supplement: Supplementary file 7 — Source data Fig. 4 [file 44319_2025_485_MOESM7_ESM.zip › Figure 4/4L/Fig. 4L_5A_60''.tif]

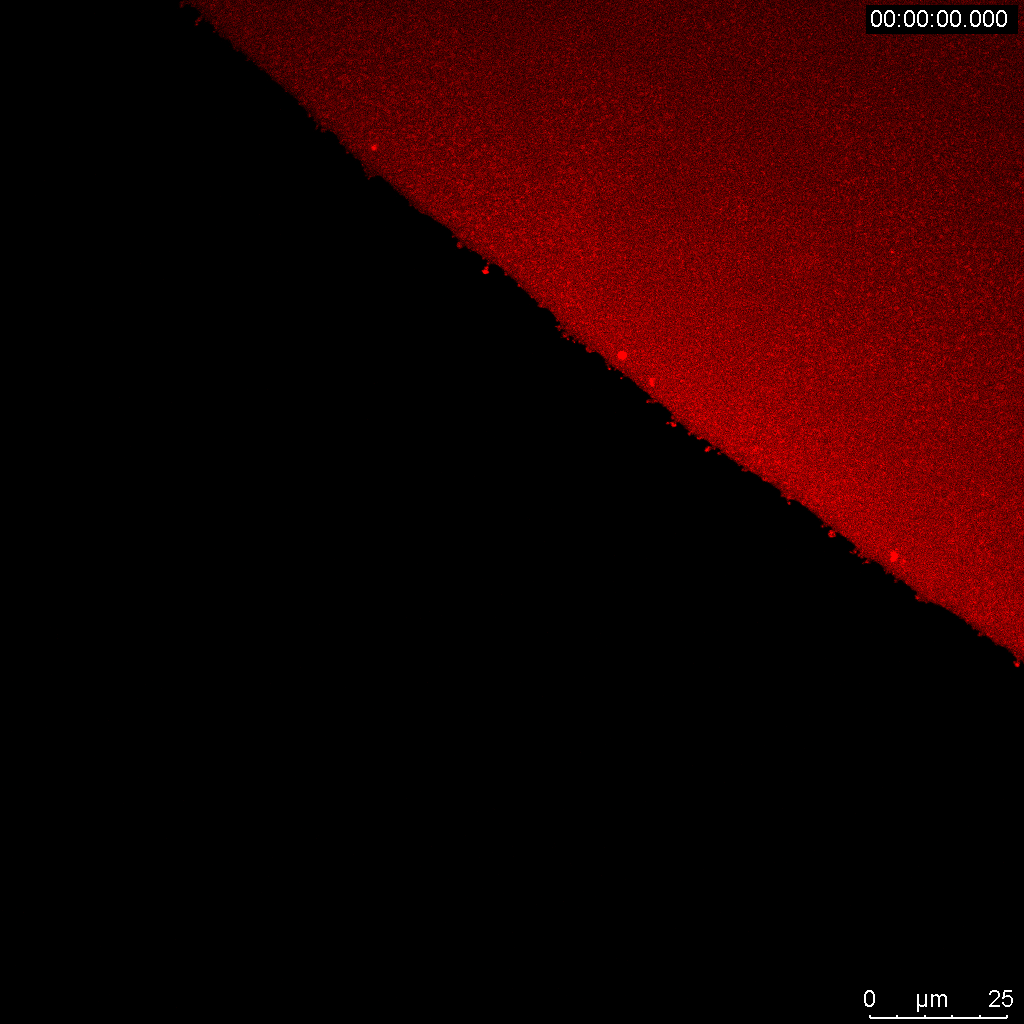

Supplement: Supplementary file 7 — Source data Fig. 4 [file 44319_2025_485_MOESM7_ESM.zip › Figure 4/4L/Fig. 4L_5D_0''.tif]

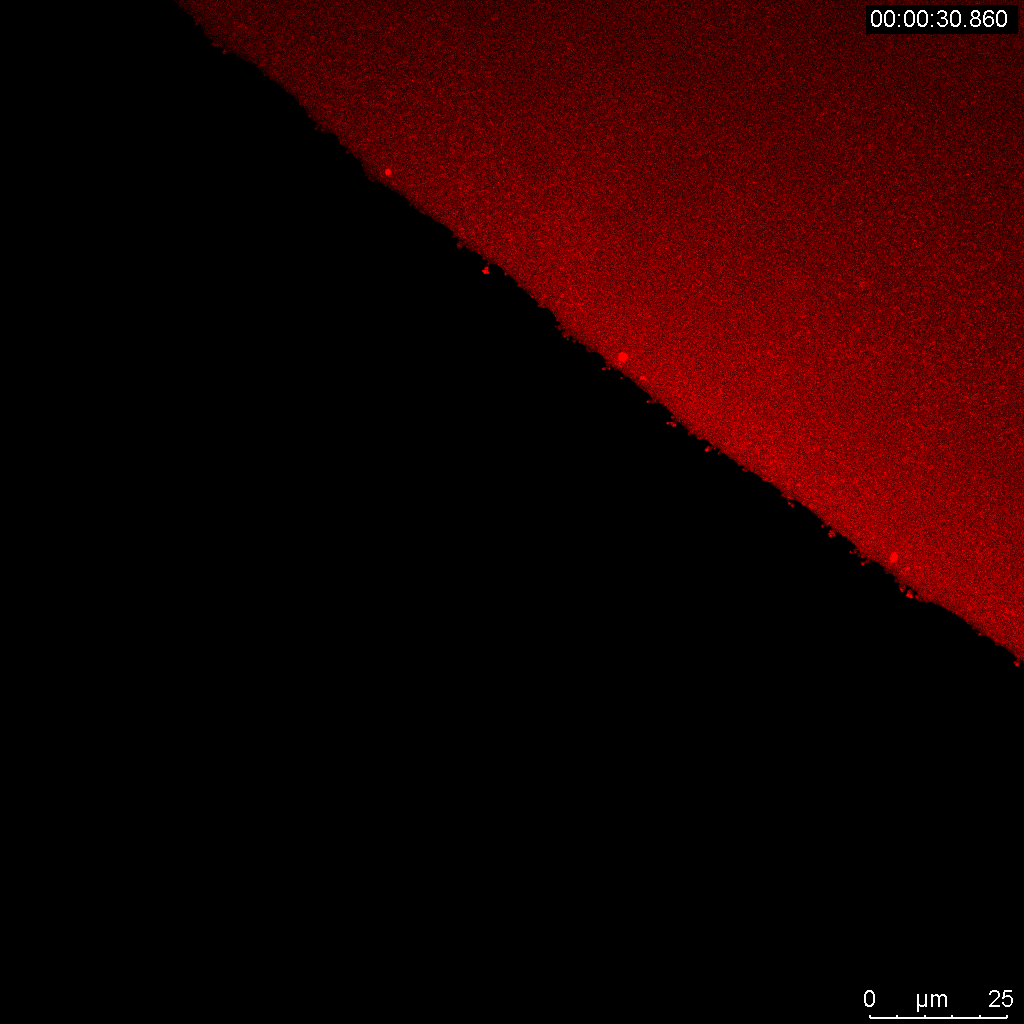

Supplement: Supplementary file 7 — Source data Fig. 4 [file 44319_2025_485_MOESM7_ESM.zip › Figure 4/4L/Fig. 4L_5D_30''.tif]

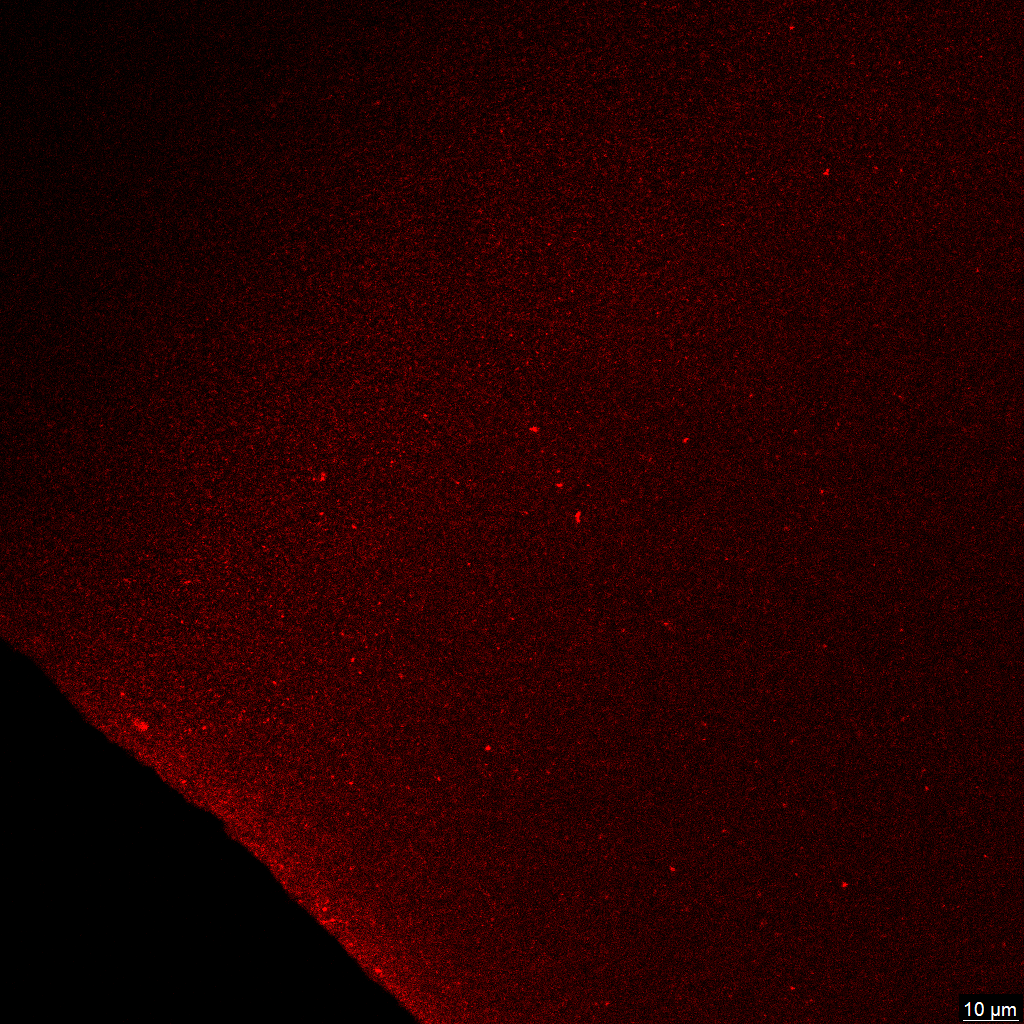

Supplement: Supplementary file 7 — Source data Fig. 4 [file 44319_2025_485_MOESM7_ESM.zip › Figure 4/4M/Fig. 4M_5D_30''.tif]

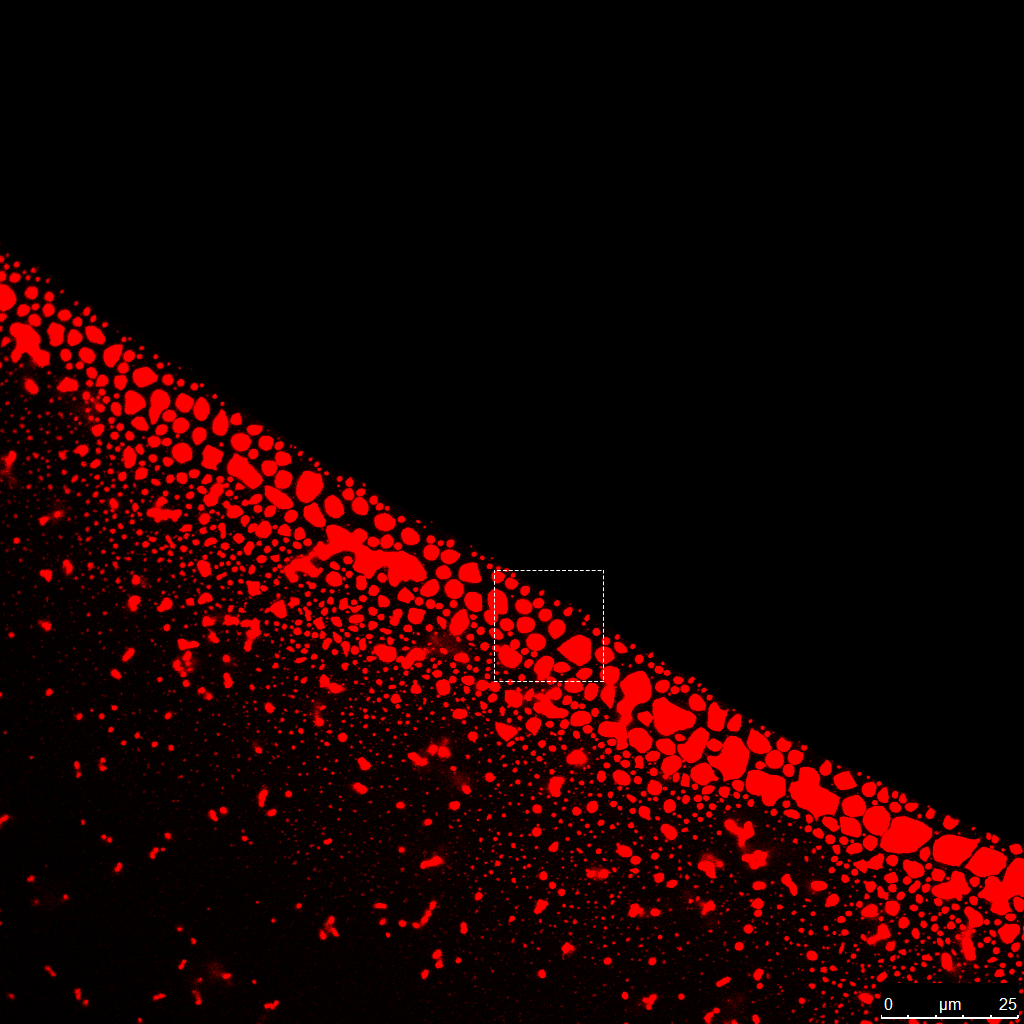

Supplement: Supplementary file 7 — Source data Fig. 4 [file 44319_2025_485_MOESM7_ESM.zip › Figure 4/4M/Fig. 4M_WT_60''.tif]

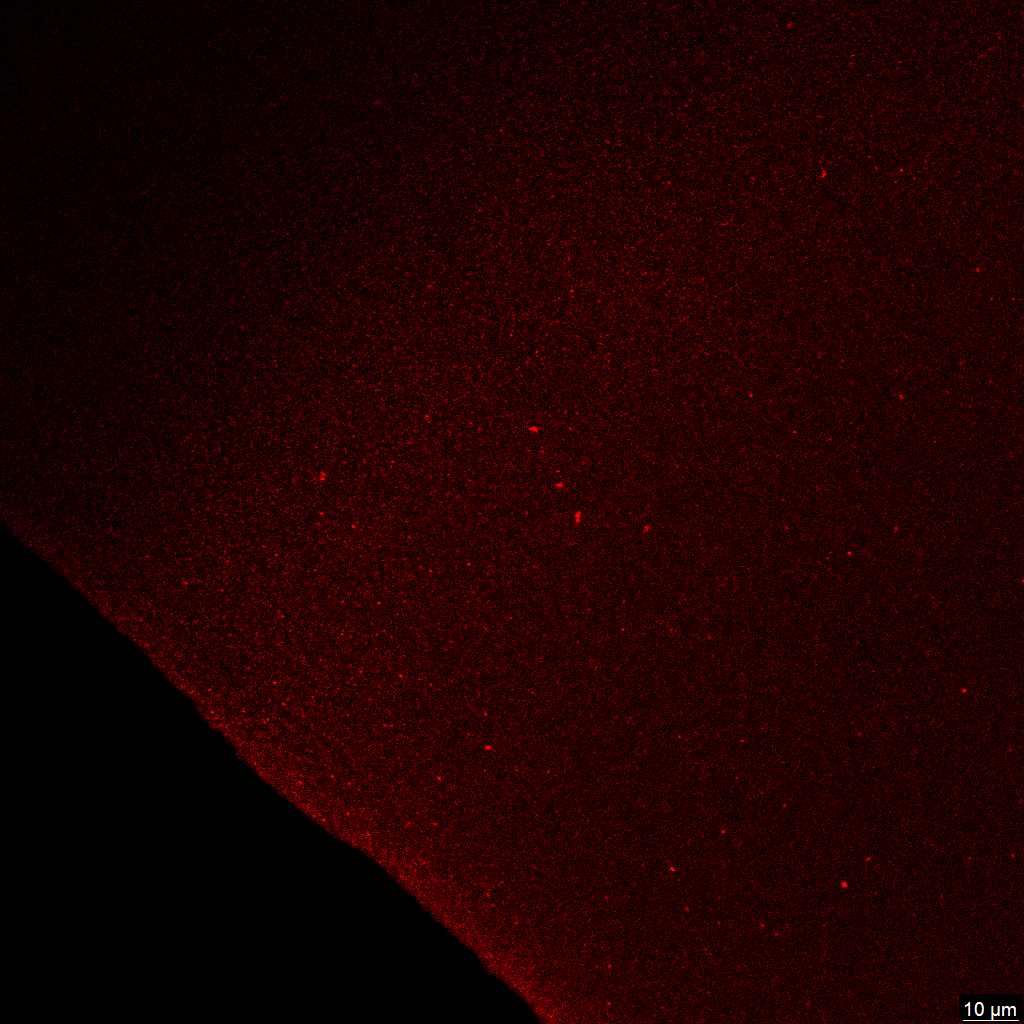

Supplement: Supplementary file 7 — Source data Fig. 4 [file 44319_2025_485_MOESM7_ESM.zip › Figure 4/4M/Fig. 4M_5D_0''.tif]

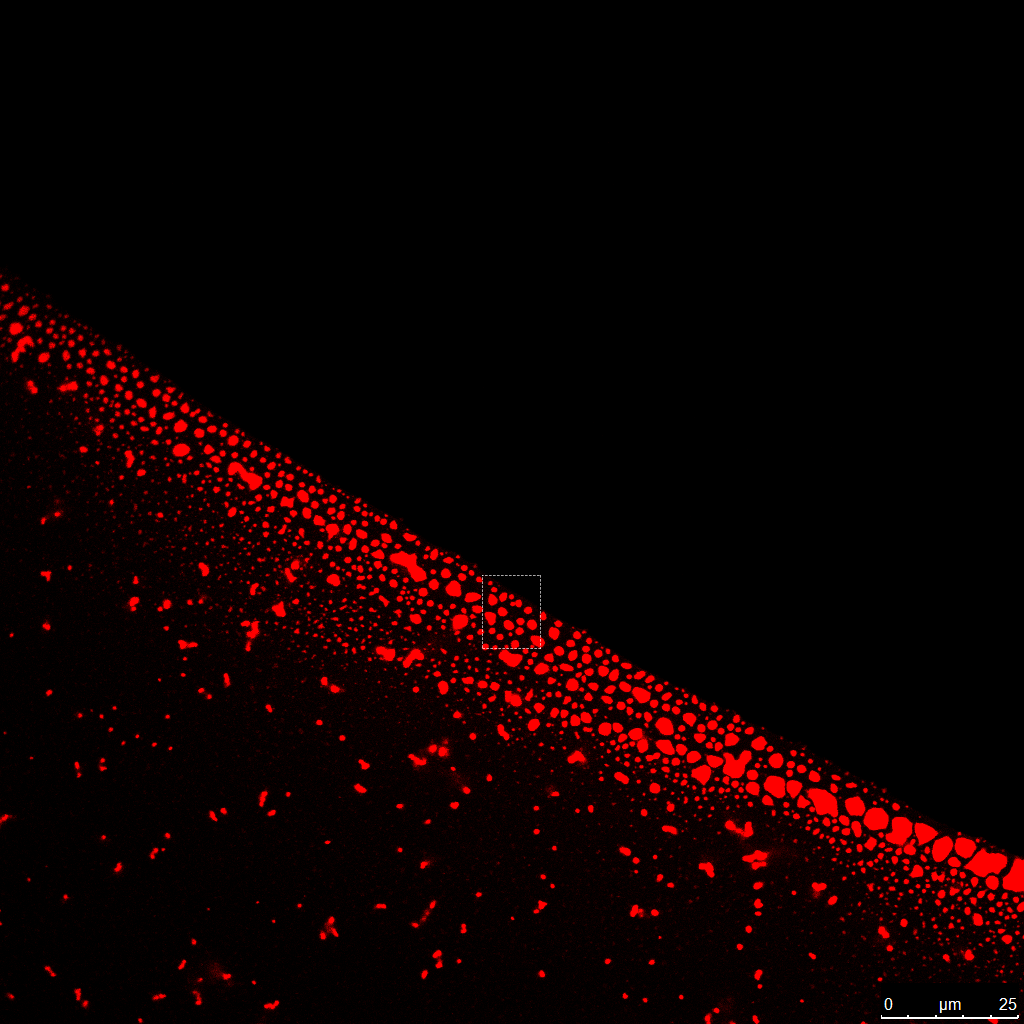

Supplement: Supplementary file 7 — Source data Fig. 4 [file 44319_2025_485_MOESM7_ESM.zip › Figure 4/4M/Fig. 4M_WT.tif]

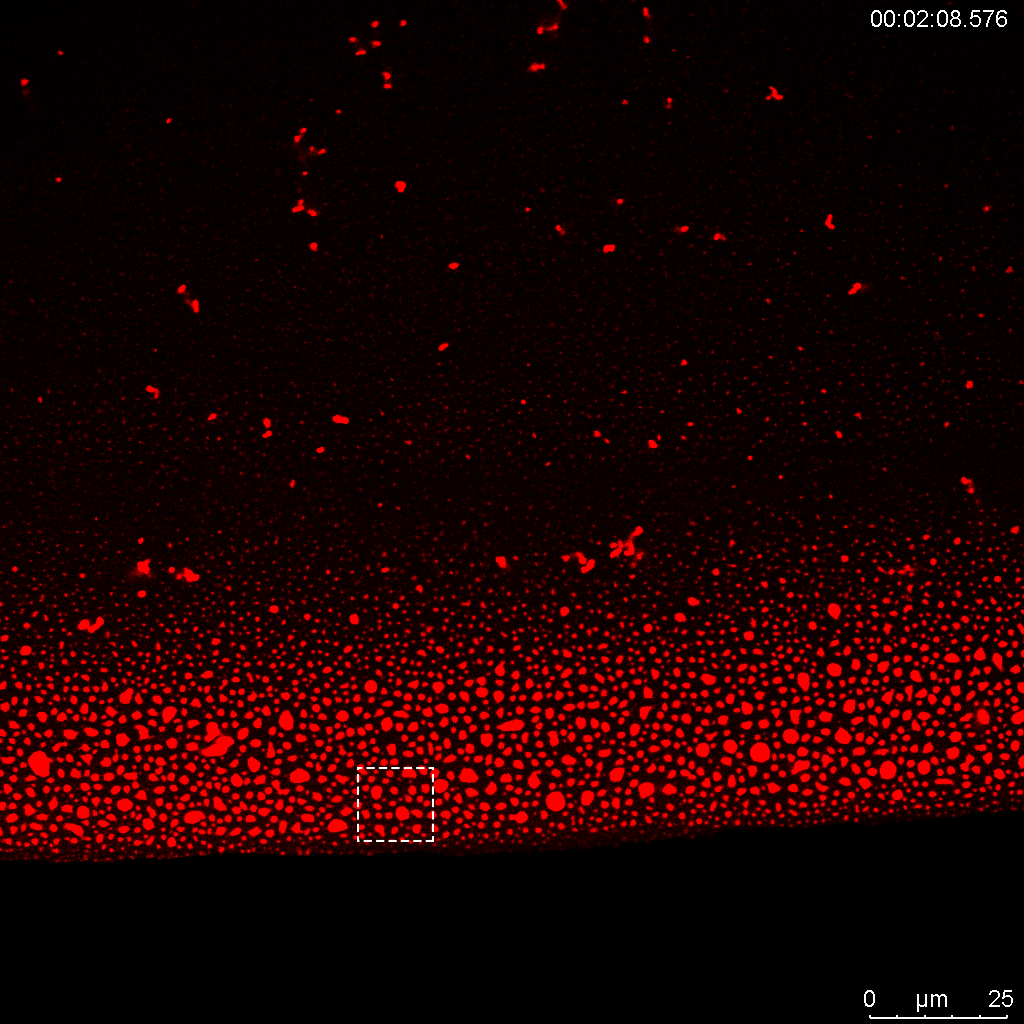

Supplement: Supplementary file 7 — Source data Fig. 4 [file 44319_2025_485_MOESM7_ESM.zip › Figure 4/4M/Fig. 4M_5A_60''.tif]

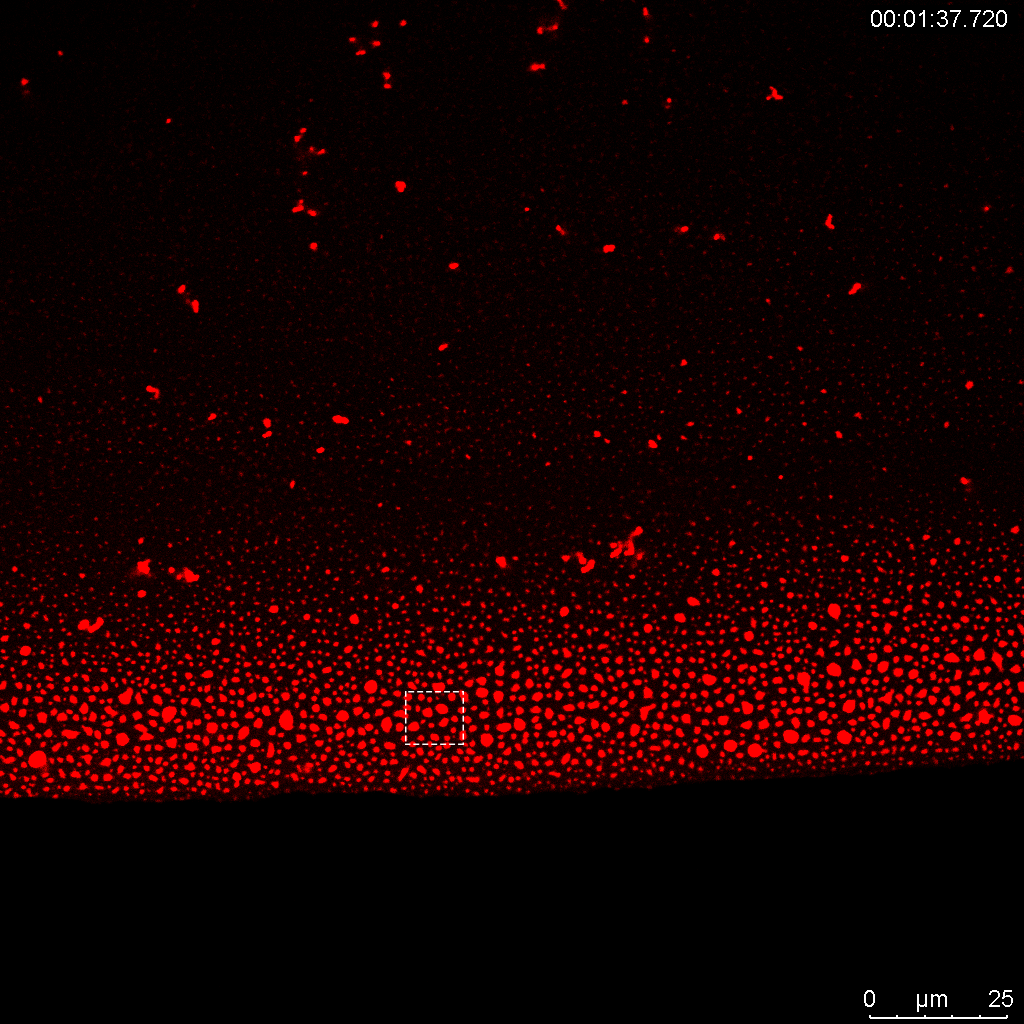

Supplement: Supplementary file 7 — Source data Fig. 4 [file 44319_2025_485_MOESM7_ESM.zip › Figure 4/4M/Fig. 4M_5A_30''.tif]

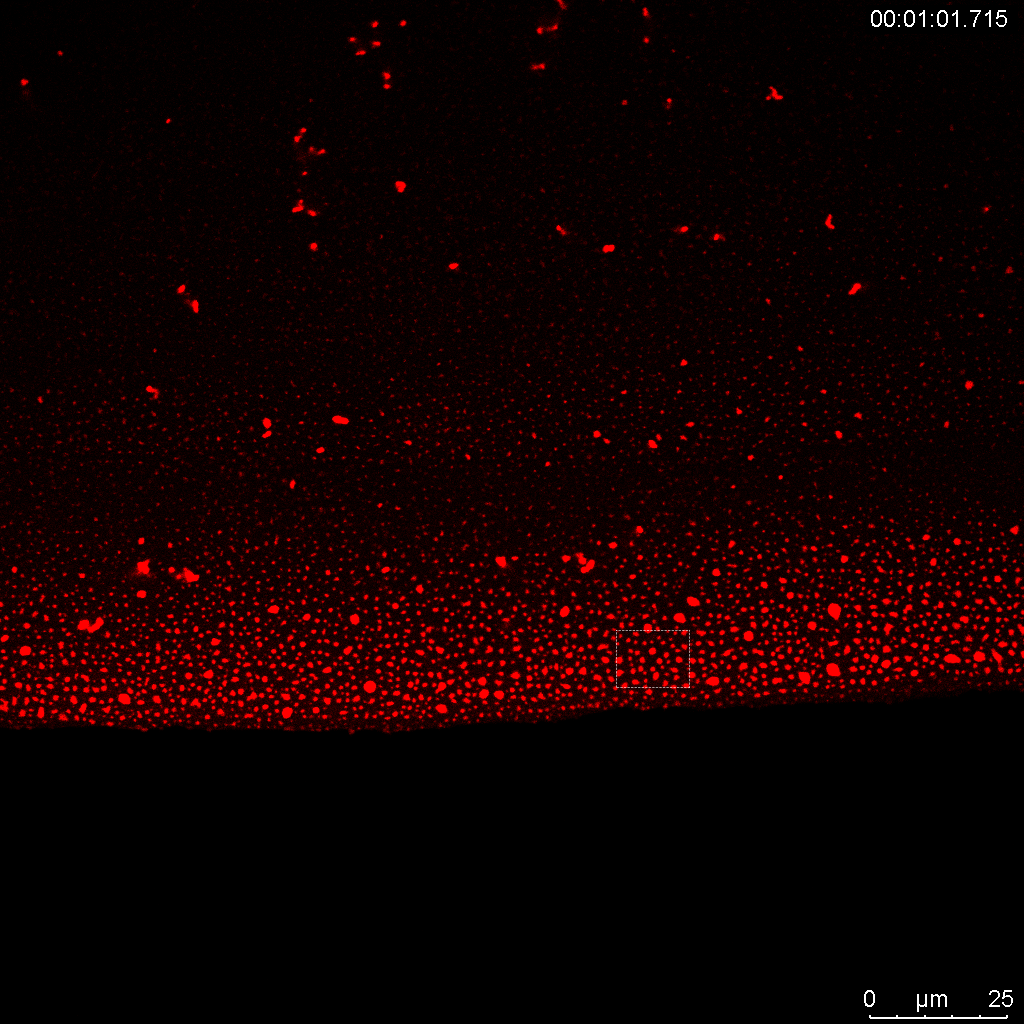

Supplement: Supplementary file 7 — Source data Fig. 4 [file 44319_2025_485_MOESM7_ESM.zip › Figure 4/4M/Fig. 4M_5A_0''.tif]

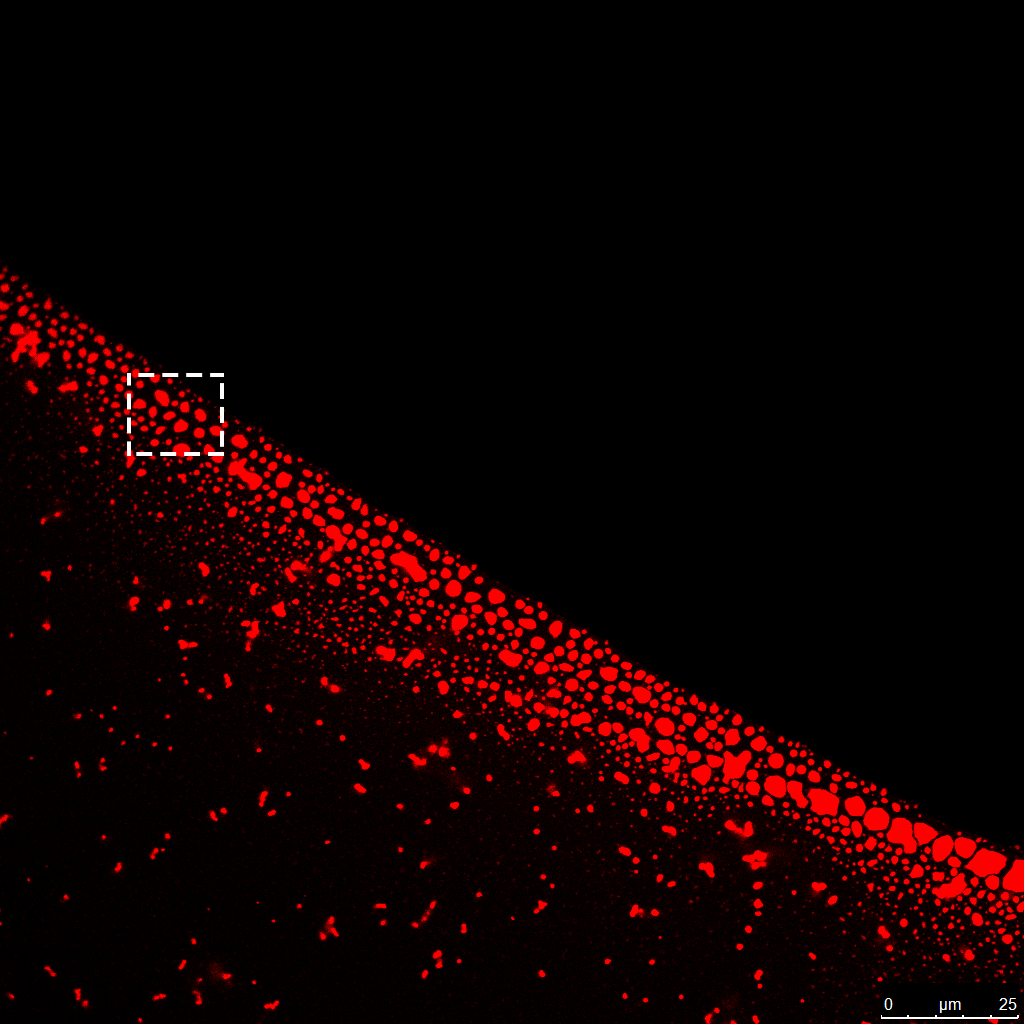

Supplement: Supplementary file 7 — Source data Fig. 4 [file 44319_2025_485_MOESM7_ESM.zip › Figure 4/4M/Fig. 4M_WT_30''.tif]

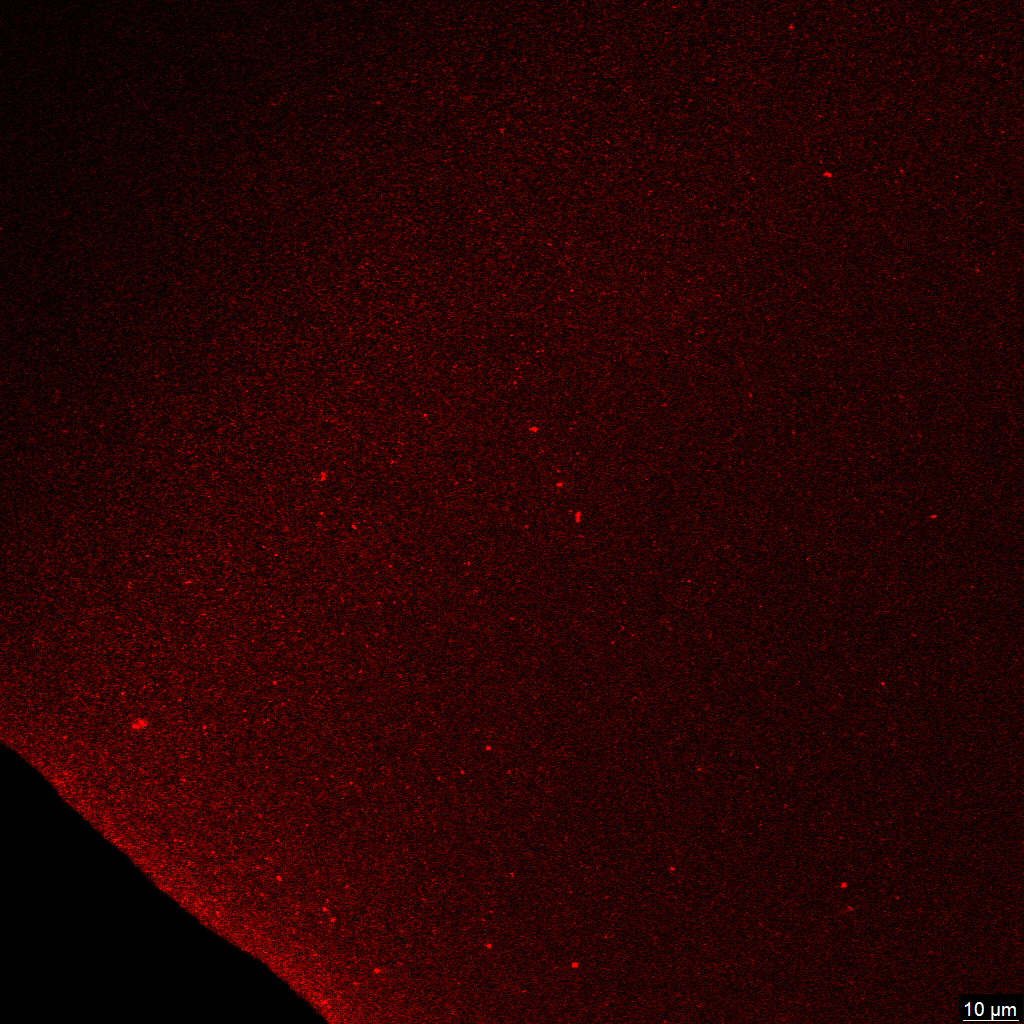

Supplement: Supplementary file 7 — Source data Fig. 4 [file 44319_2025_485_MOESM7_ESM.zip › Figure 4/4M/Fig. 4M_5D_60''.tif]

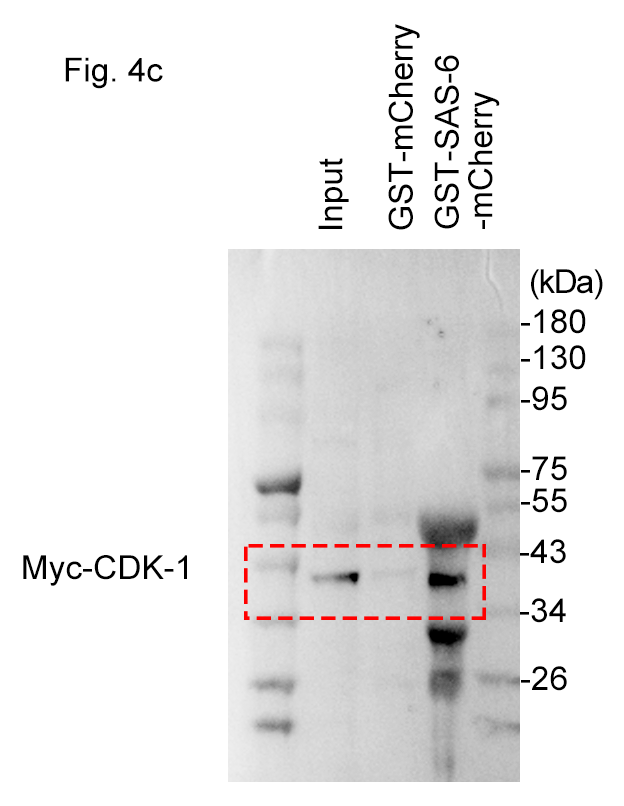

Supplement: Supplementary file 7 — Source data Fig. 4 [file 44319_2025_485_MOESM7_ESM.zip › Figure 4/4C/Fig. 4C_Myc_CDK1.tif]

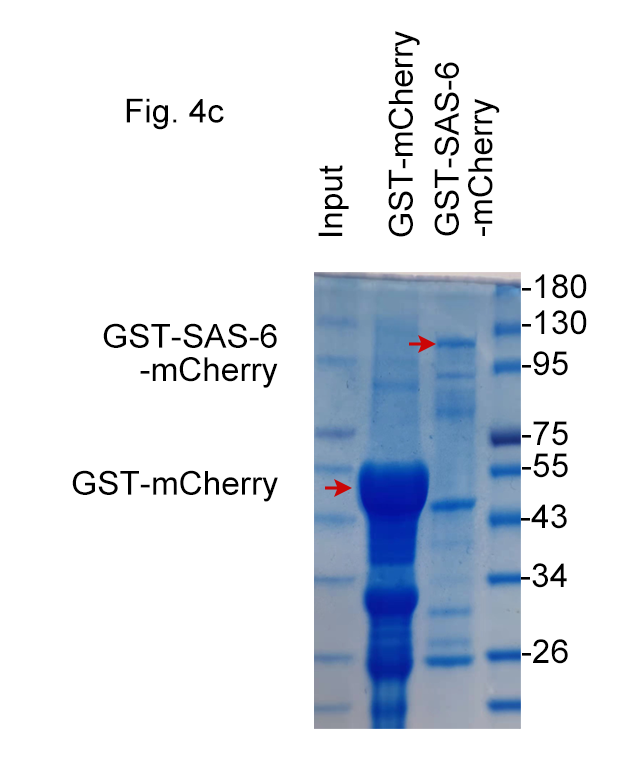

Supplement: Supplementary file 7 — Source data Fig. 4 [file 44319_2025_485_MOESM7_ESM.zip › Figure 4/4C/Fig. 4C_CBB.tif]

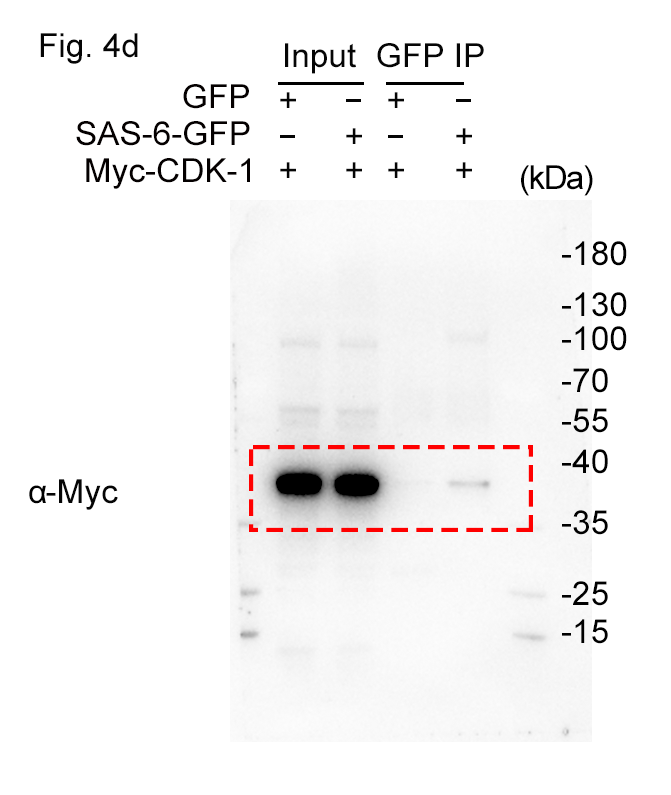

Supplement: Supplementary file 7 — Source data Fig. 4 [file 44319_2025_485_MOESM7_ESM.zip › Figure 4/4D/Fig. 4D_Myc.tif]

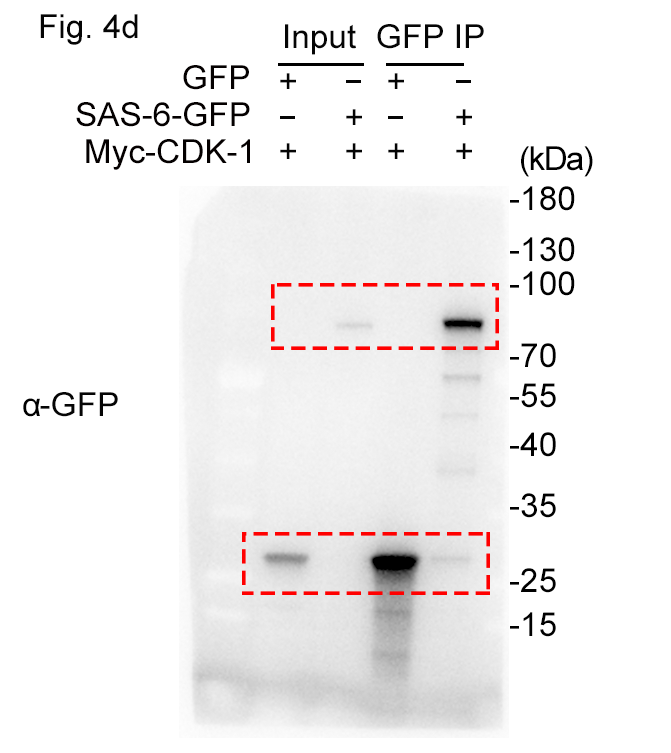

Supplement: Supplementary file 7 — Source data Fig. 4 [file 44319_2025_485_MOESM7_ESM.zip › Figure 4/4D/Fig. 4D_GFP.tif]

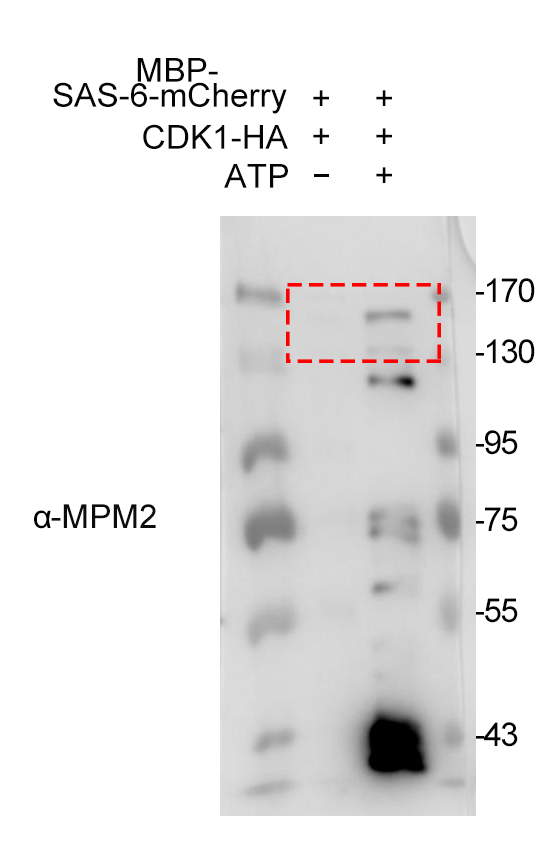

Supplement: Supplementary file 7 — Source data Fig. 4 [file 44319_2025_485_MOESM7_ESM.zip › Figure 4/4F/Fig. 4F_MPM2.tif]

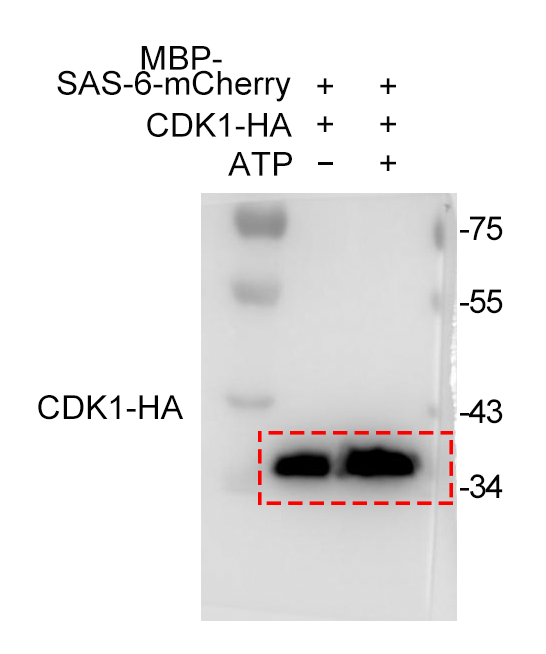

Supplement: Supplementary file 7 — Source data Fig. 4 [file 44319_2025_485_MOESM7_ESM.zip › Figure 4/4F/Fig. 4F_CDK1_HA.tif]

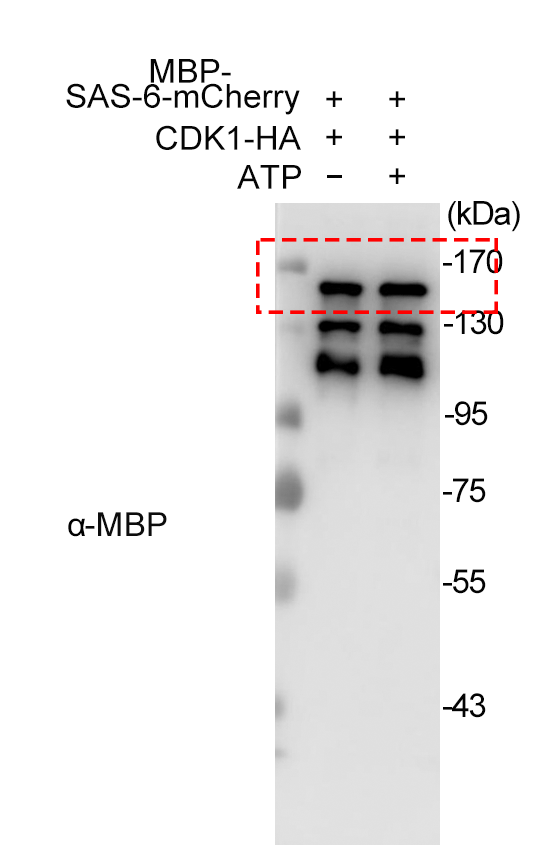

Supplement: Supplementary file 7 — Source data Fig. 4 [file 44319_2025_485_MOESM7_ESM.zip › Figure 4/4F/Fig. 4F_MBP.tif]

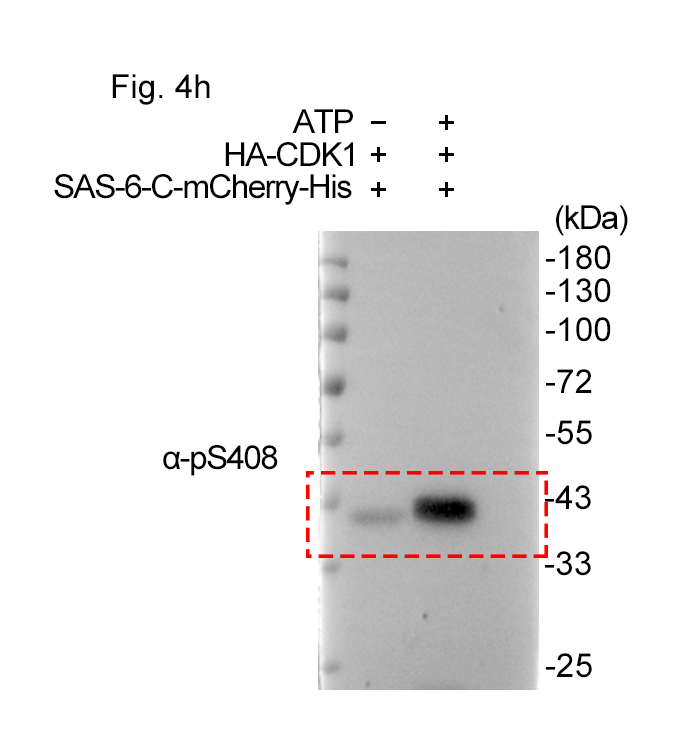

Supplement: Supplementary file 7 — Source data Fig. 4 [file 44319_2025_485_MOESM7_ESM.zip › Figure 4/4H/Fig. 4H_pS408.tif]

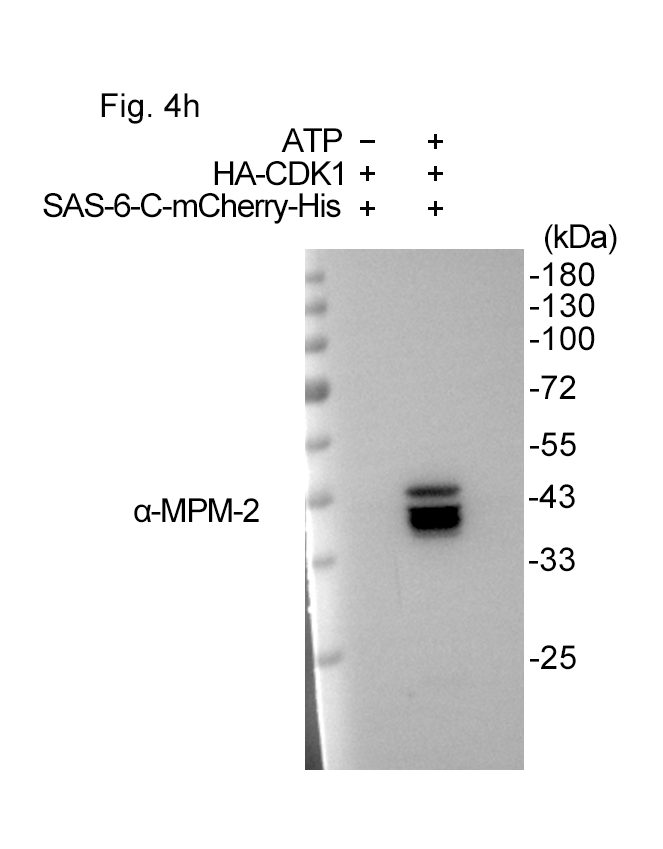

Supplement: Supplementary file 7 — Source data Fig. 4 [file 44319_2025_485_MOESM7_ESM.zip › Figure 4/4H/Fig. 4H_MPM2.tif]

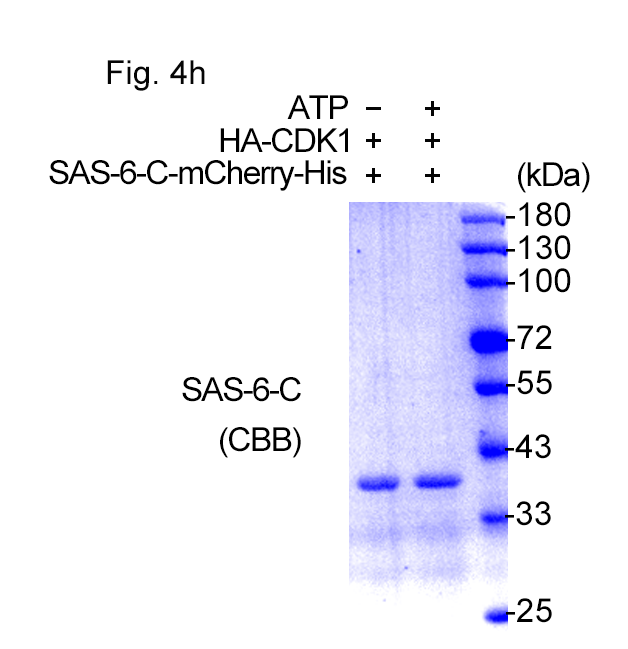

Supplement: Supplementary file 7 — Source data Fig. 4 [file 44319_2025_485_MOESM7_ESM.zip › Figure 4/4H/Fig. 4H_CBB.tif]

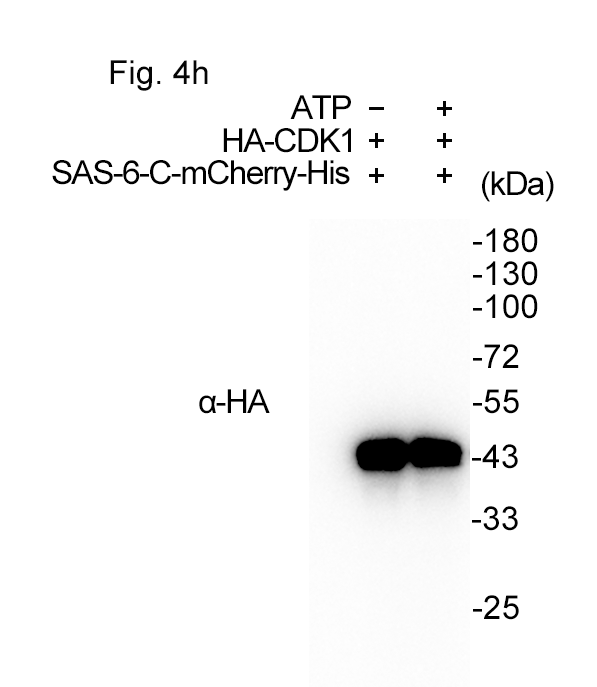

Supplement: Supplementary file 7 — Source data Fig. 4 [file 44319_2025_485_MOESM7_ESM.zip › Figure 4/4H/Fig. 4H_HA.tif]

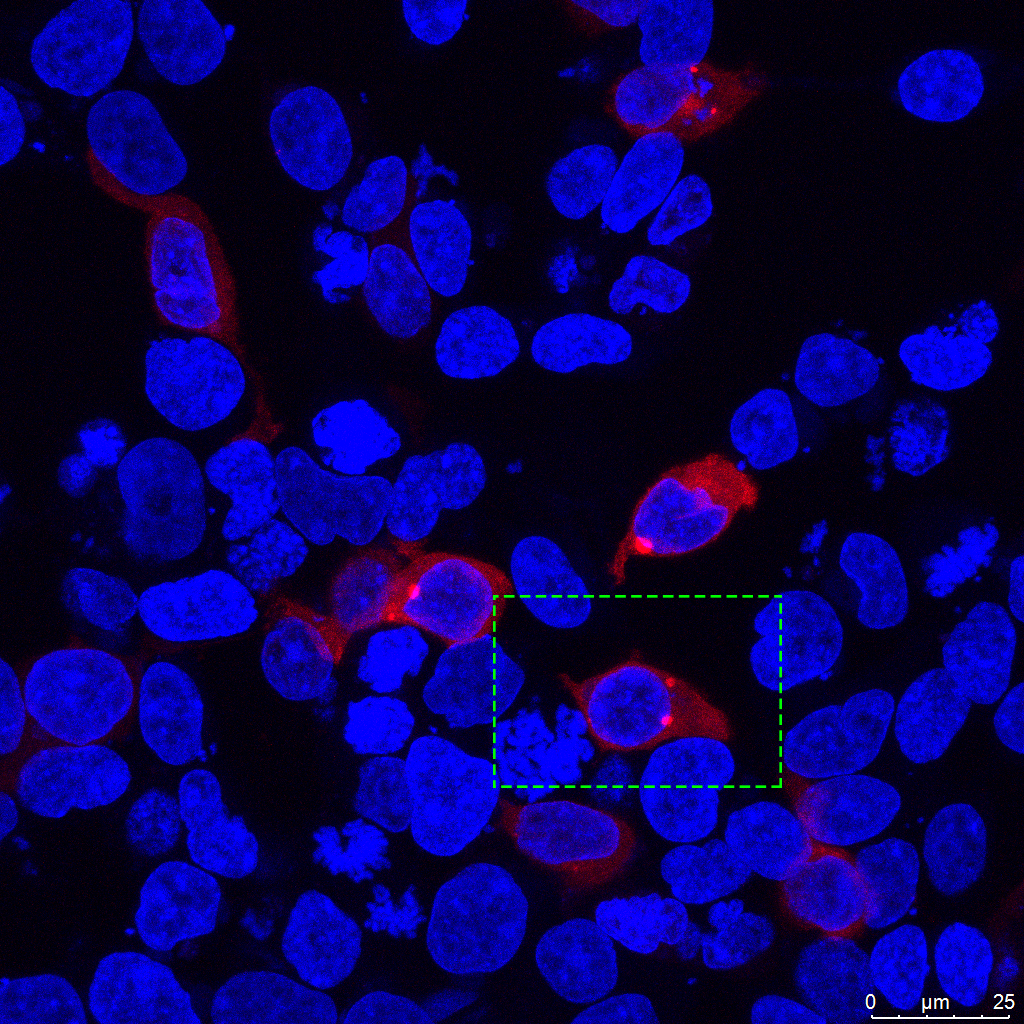

Supplement: Supplementary file 7 — Source data Fig. 4 [file 44319_2025_485_MOESM7_ESM.zip › Figure 4/4I/Fig. 4I_5A.tif]

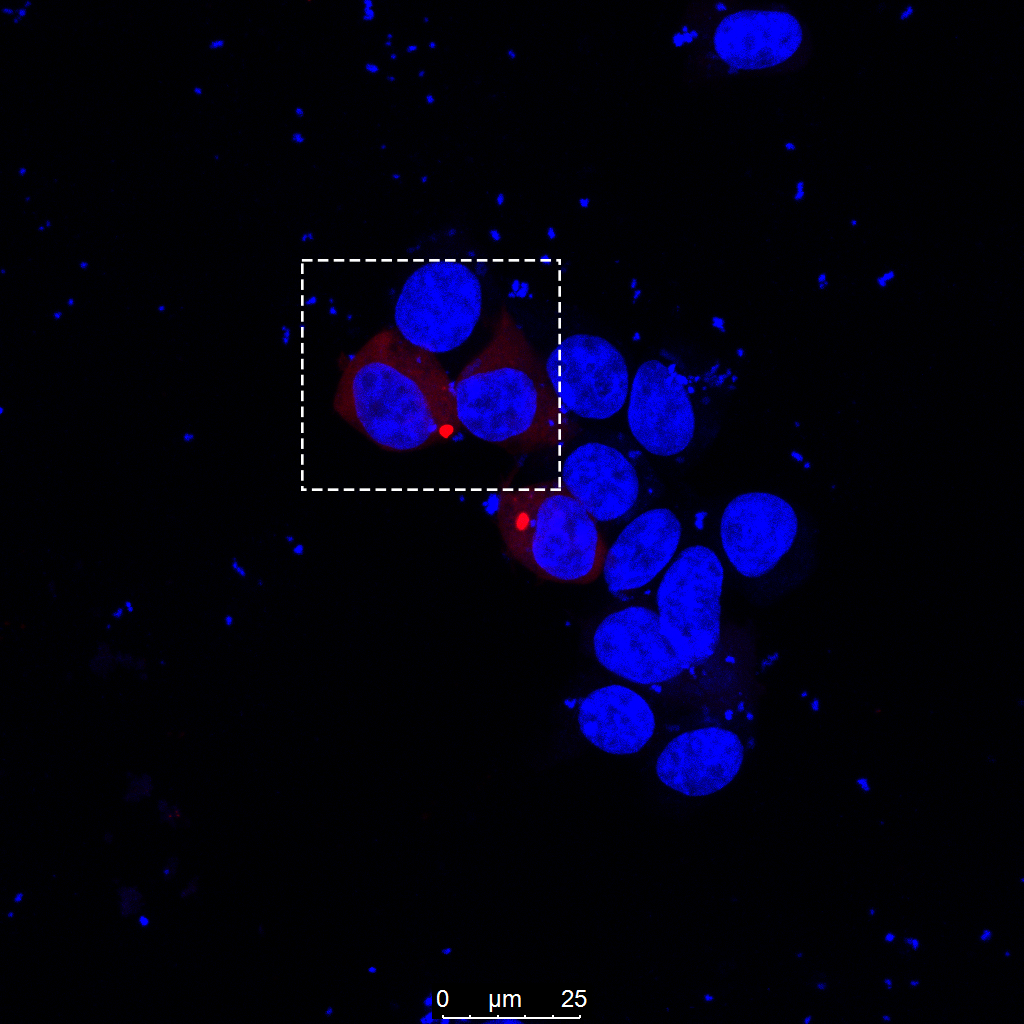

Supplement: Supplementary file 7 — Source data Fig. 4 [file 44319_2025_485_MOESM7_ESM.zip › Figure 4/4I/Fig. 4I_WT.tif]

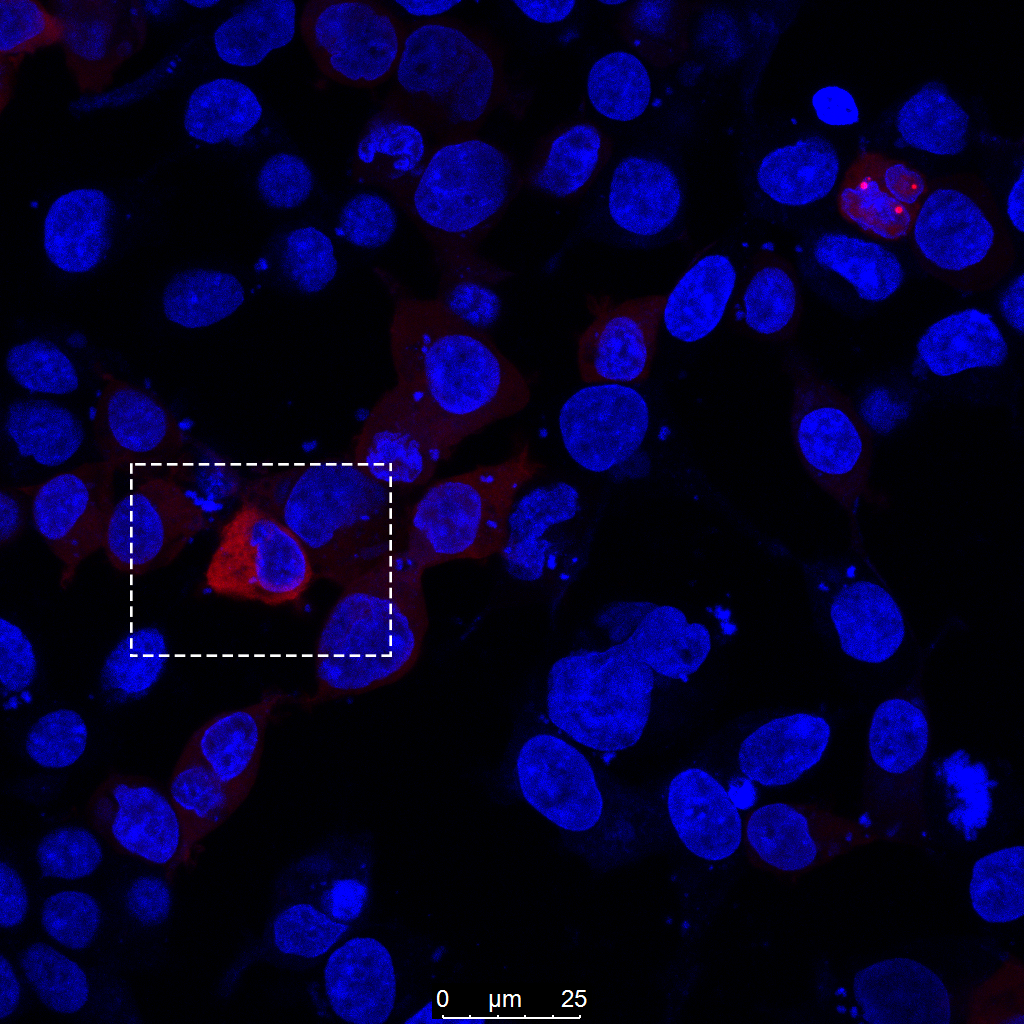

Supplement: Supplementary file 7 — Source data Fig. 4 [file 44319_2025_485_MOESM7_ESM.zip › Figure 4/4I/Fig. 4I_5D.tif]

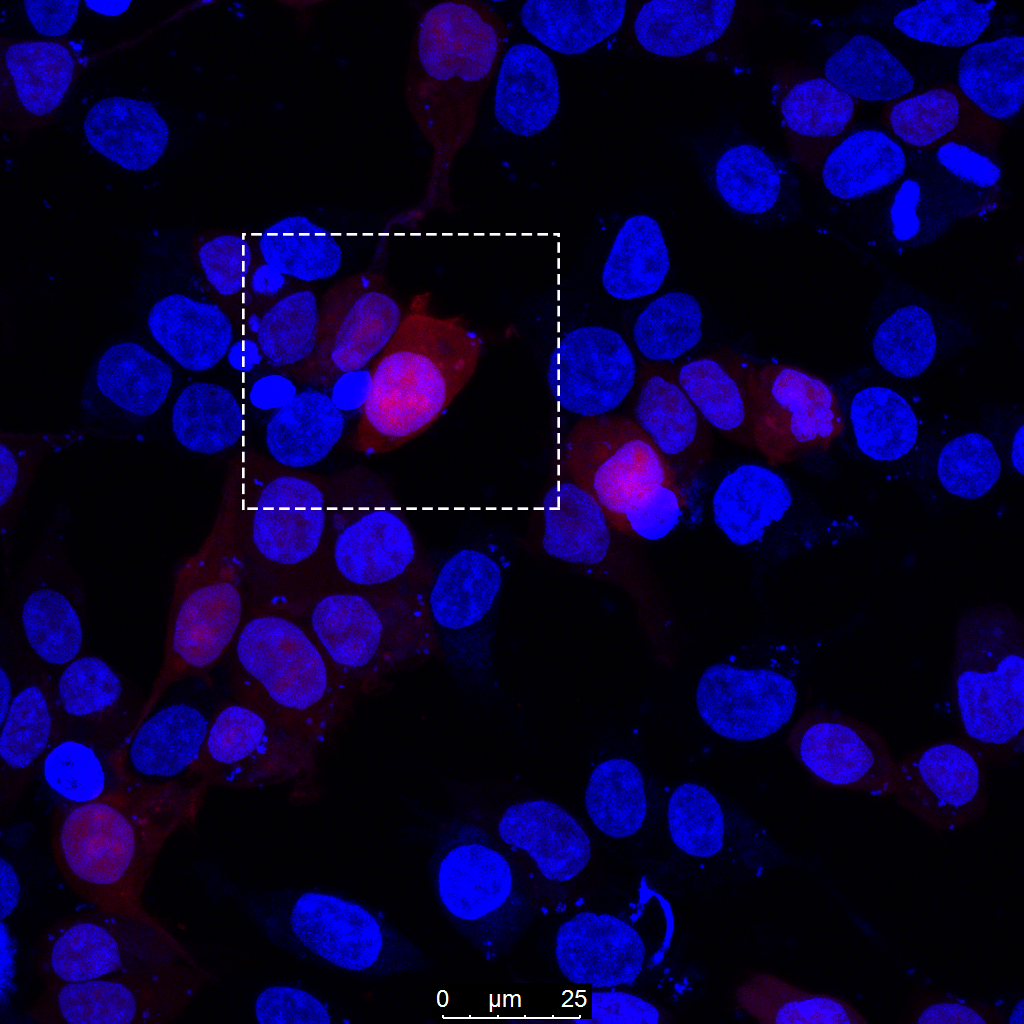

Supplement: Supplementary file 7 — Source data Fig. 4 [file 44319_2025_485_MOESM7_ESM.zip › Figure 4/4I/Fig. 4I_mCherry.tif]

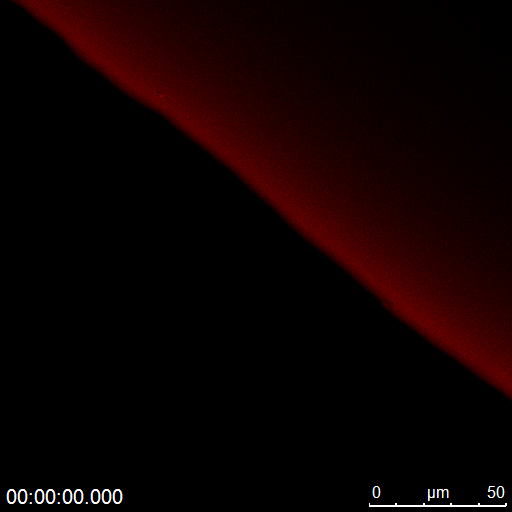

Supplement: Supplementary file 7 — Source data Fig. 4 [file 44319_2025_485_MOESM7_ESM.zip › Figure 4/4N/Fig. 4N_preP_0'.tif]

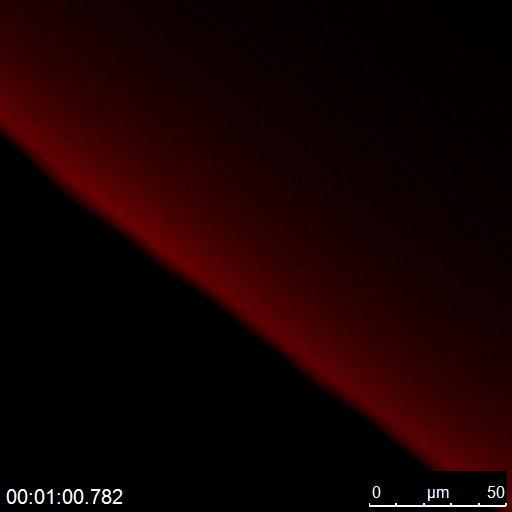

Supplement: Supplementary file 7 — Source data Fig. 4 [file 44319_2025_485_MOESM7_ESM.zip › Figure 4/4N/Fig. 4N_preP_1'.tif]

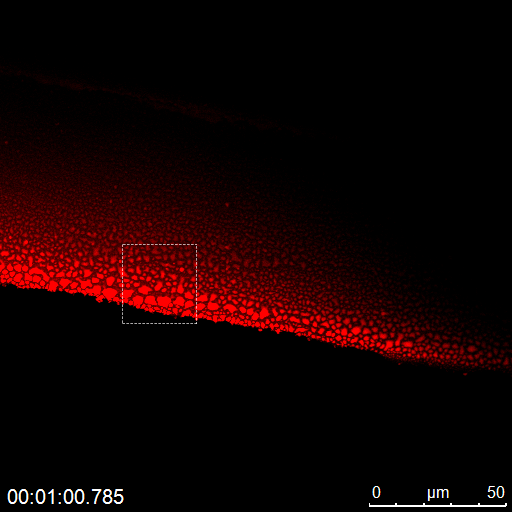

Supplement: Supplementary file 7 — Source data Fig. 4 [file 44319_2025_485_MOESM7_ESM.zip › Figure 4/4N/Fig. 4N_Control_1'.tif]

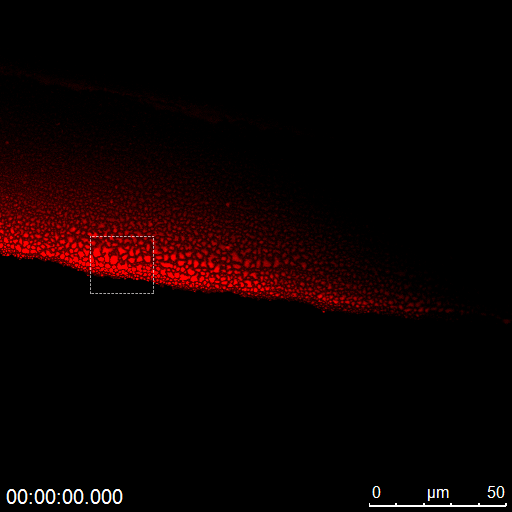

Supplement: Supplementary file 7 — Source data Fig. 4 [file 44319_2025_485_MOESM7_ESM.zip › Figure 4/4N/Fig. 4N_Control_0'.tif]

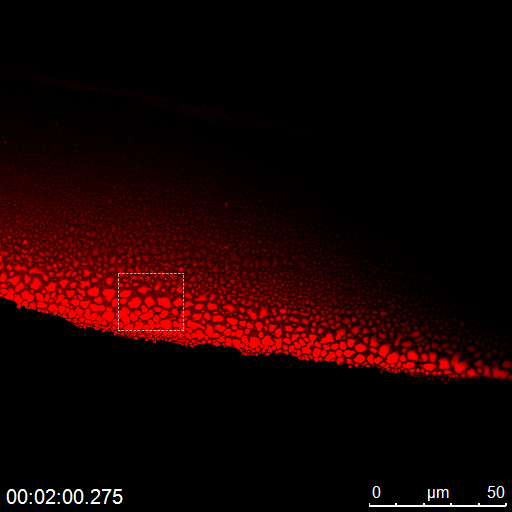

Supplement: Supplementary file 7 — Source data Fig. 4 [file 44319_2025_485_MOESM7_ESM.zip › Figure 4/4N/Fig. 4N_Control_2'.tif]

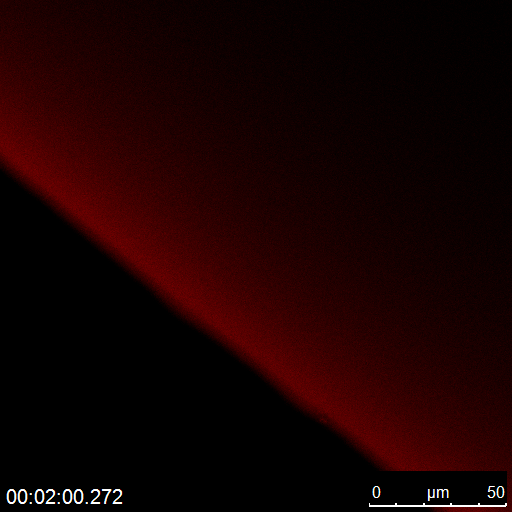

Supplement: Supplementary file 7 — Source data Fig. 4 [file 44319_2025_485_MOESM7_ESM.zip › Figure 4/4N/Fig. 4N_preP_2'.tif]

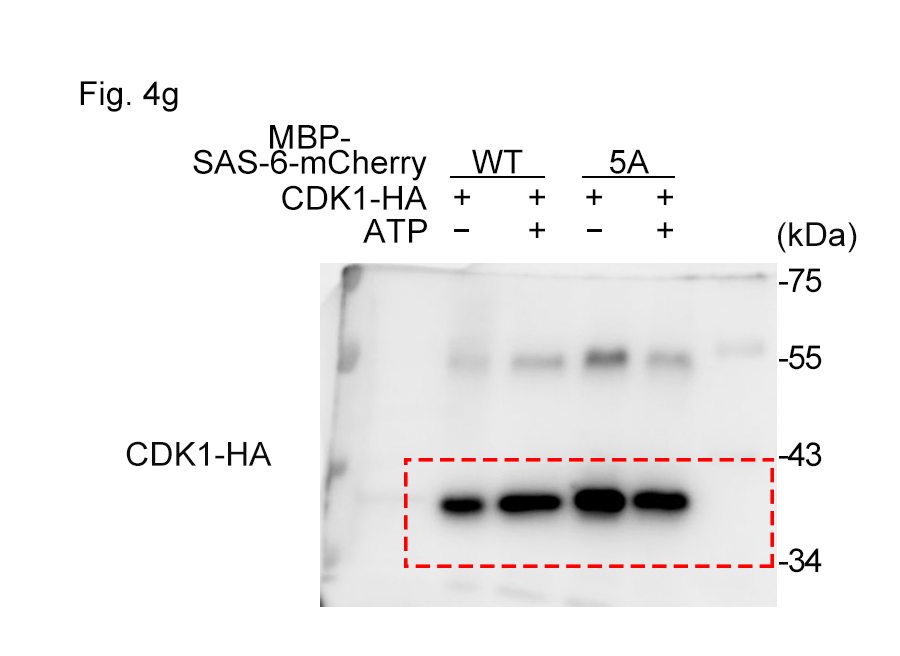

Supplement: Supplementary file 7 — Source data Fig. 4 [file 44319_2025_485_MOESM7_ESM.zip › Figure 4/4G/Fig. 4G_CDK1_HA.tif]

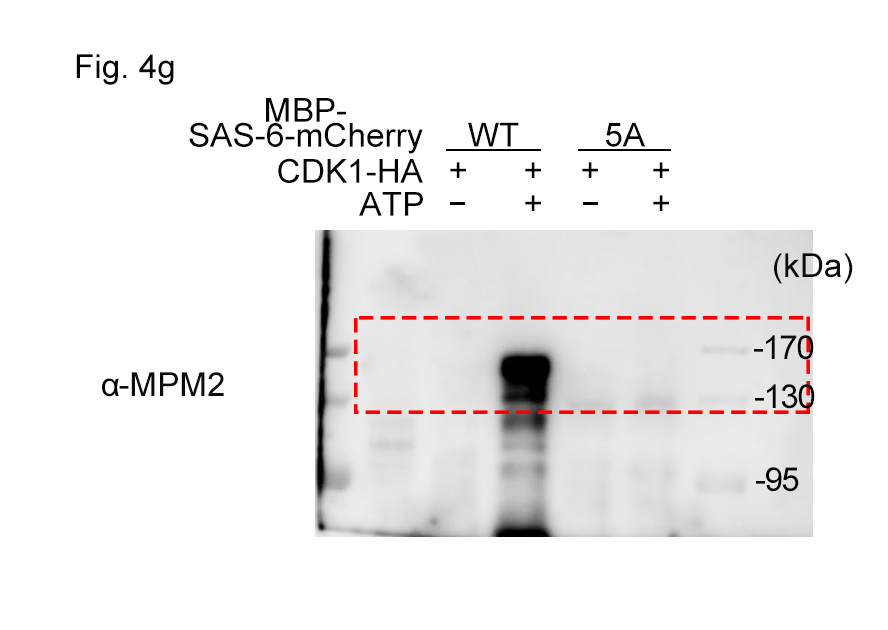

Supplement: Supplementary file 7 — Source data Fig. 4 [file 44319_2025_485_MOESM7_ESM.zip › Figure 4/4G/Fig. 4G_MPM2.tif]

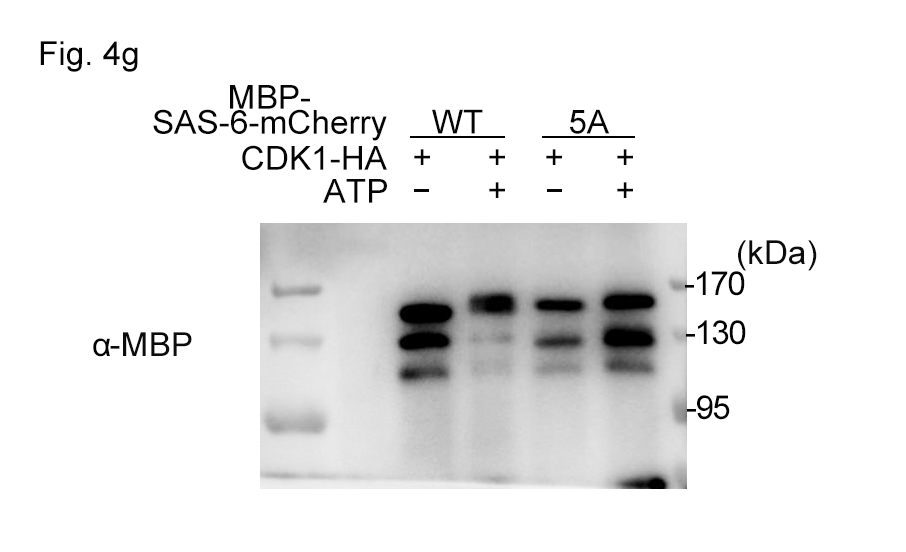

Supplement: Supplementary file 7 — Source data Fig. 4 [file 44319_2025_485_MOESM7_ESM.zip › Figure 4/4G/Fig. 4G_MBP.tif]

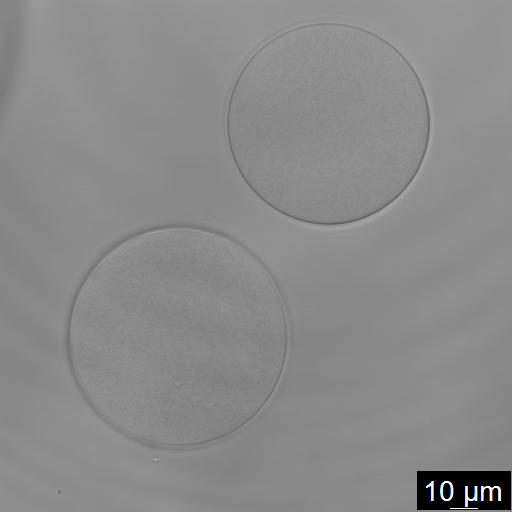

Supplement: Supplementary file 8 — Source data Fig. 5 [file 44319_2025_485_MOESM8_ESM.zip › Figure 5/5I/Fig_5I_╬öC_BF.tif]

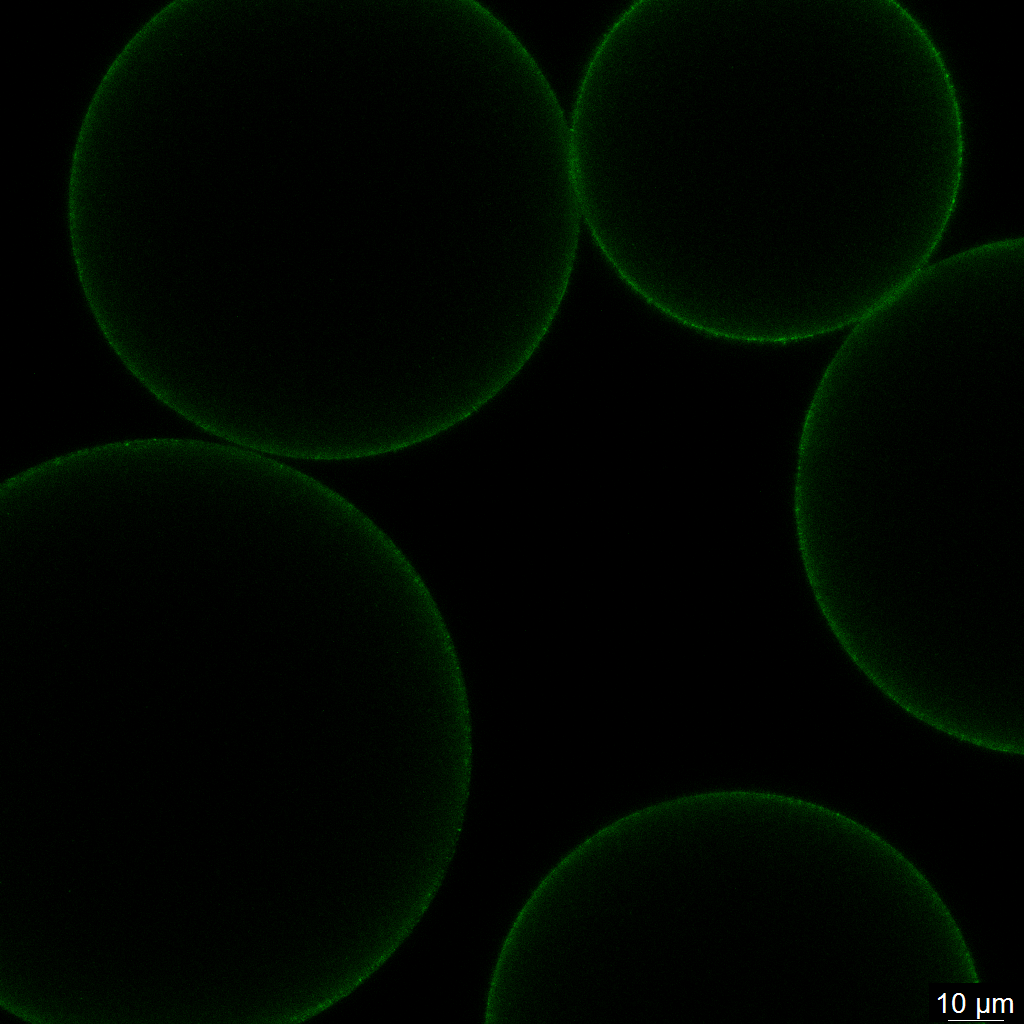

Supplement: Supplementary file 8 — Source data Fig. 5 [file 44319_2025_485_MOESM8_ESM.zip › Figure 5/5I/Fig_5I_╬öN_SAS_4_GFP.tif]

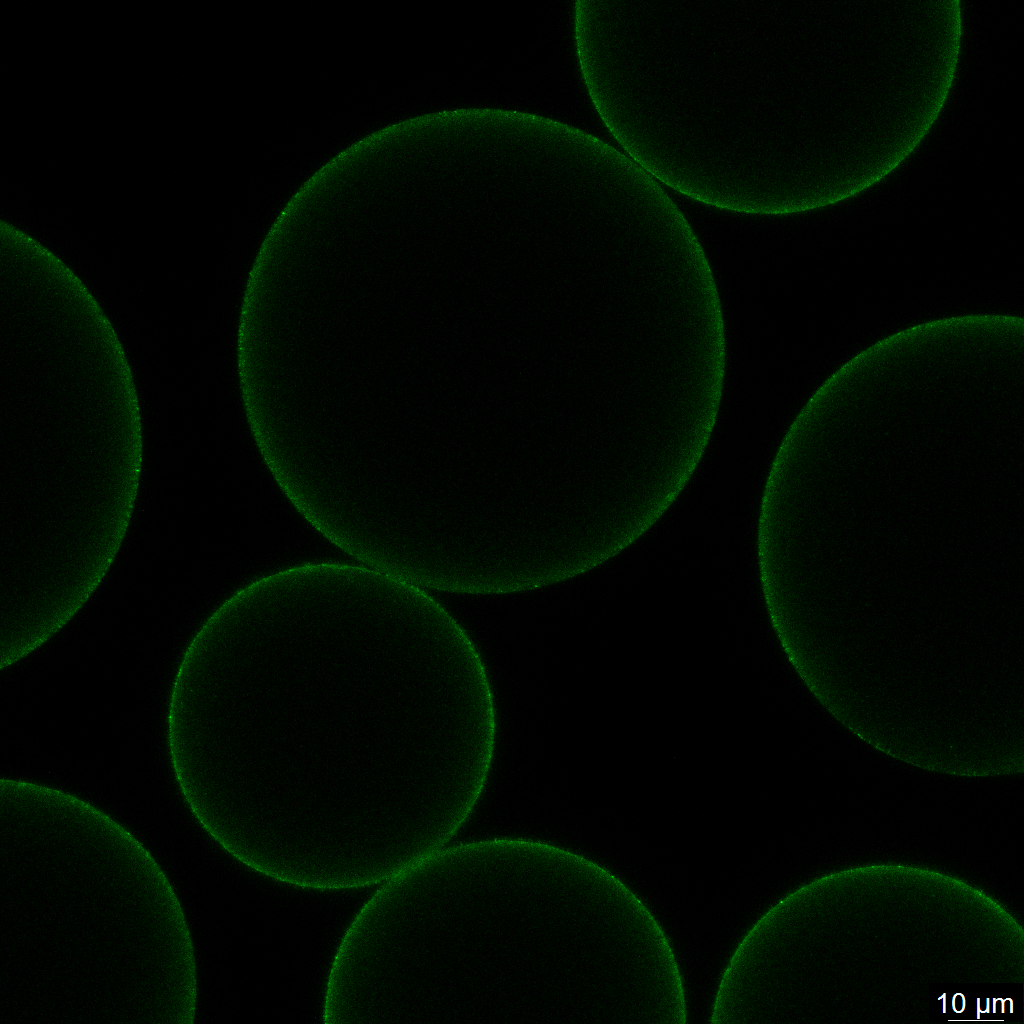

Supplement: Supplementary file 8 — Source data Fig. 5 [file 44319_2025_485_MOESM8_ESM.zip › Figure 5/5I/Fig_5I_FL_SAS_4_GFP.tif]

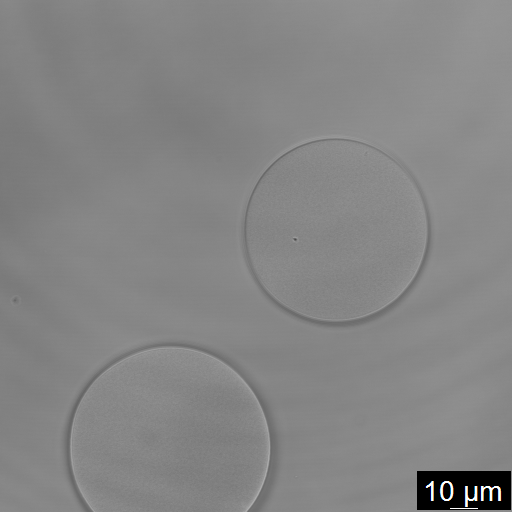

Supplement: Supplementary file 8 — Source data Fig. 5 [file 44319_2025_485_MOESM8_ESM.zip › Figure 5/5I/Fig_5I_M_SAS_4_GFP_preP.tif]

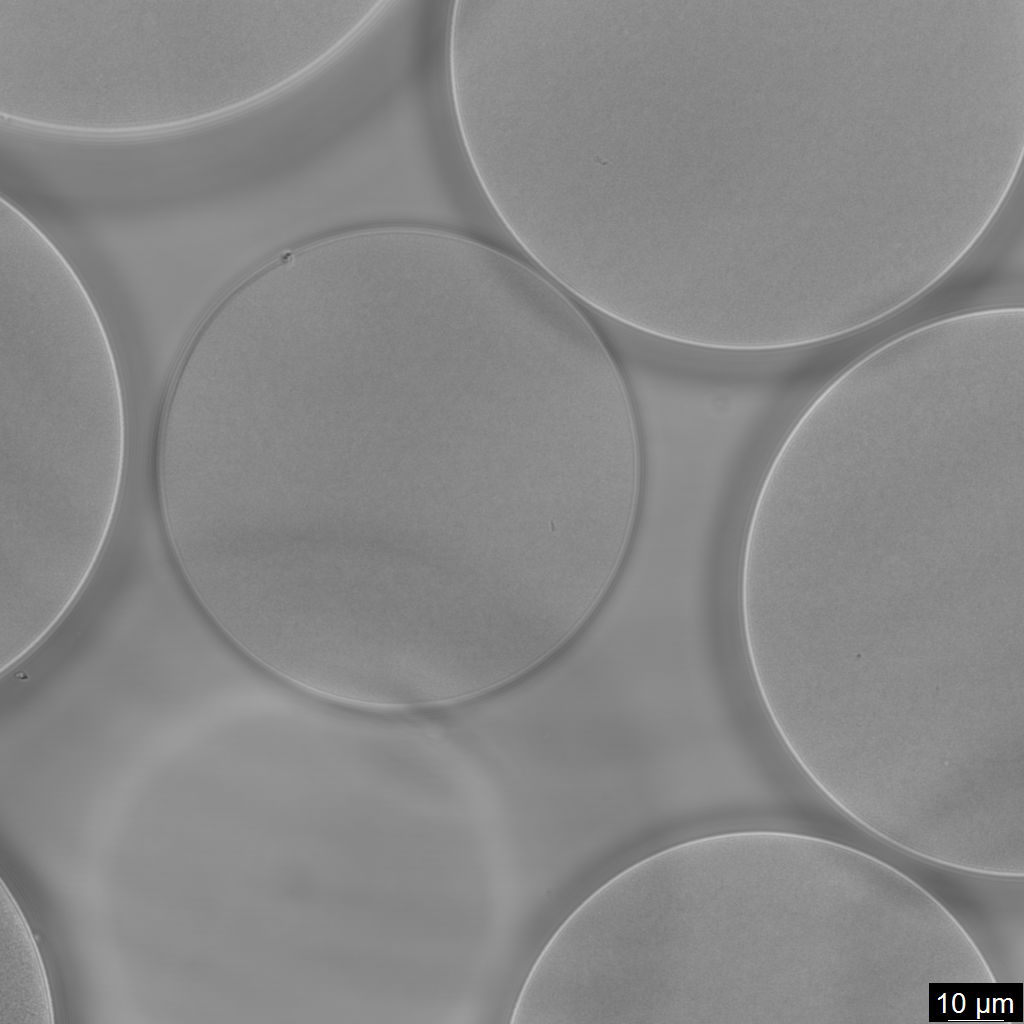

Supplement: Supplementary file 8 — Source data Fig. 5 [file 44319_2025_485_MOESM8_ESM.zip › Figure 5/5I/Fig_5I_╬öN_BF_preP.tif]

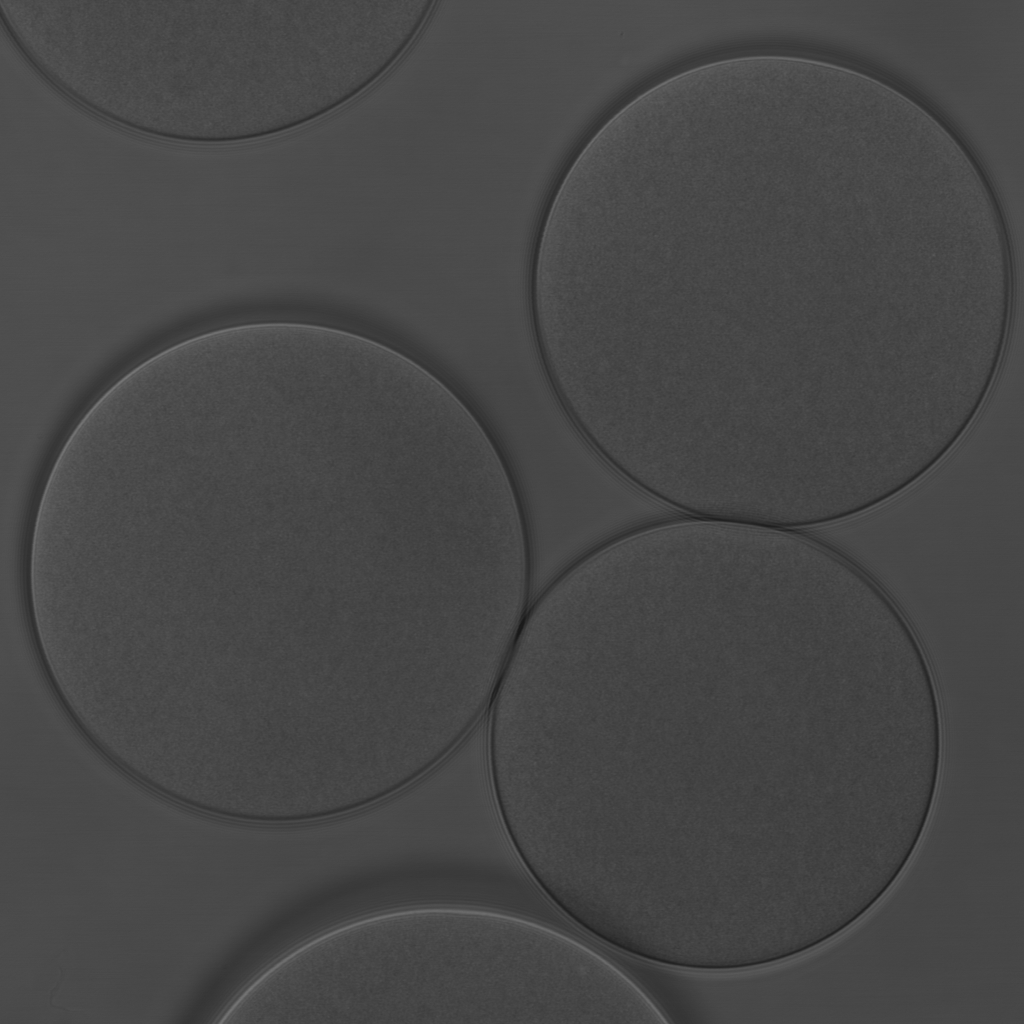

Supplement: Supplementary file 8 — Source data Fig. 5 [file 44319_2025_485_MOESM8_ESM.zip › Figure 5/5I/Fig_5I_GST_BF.tif]

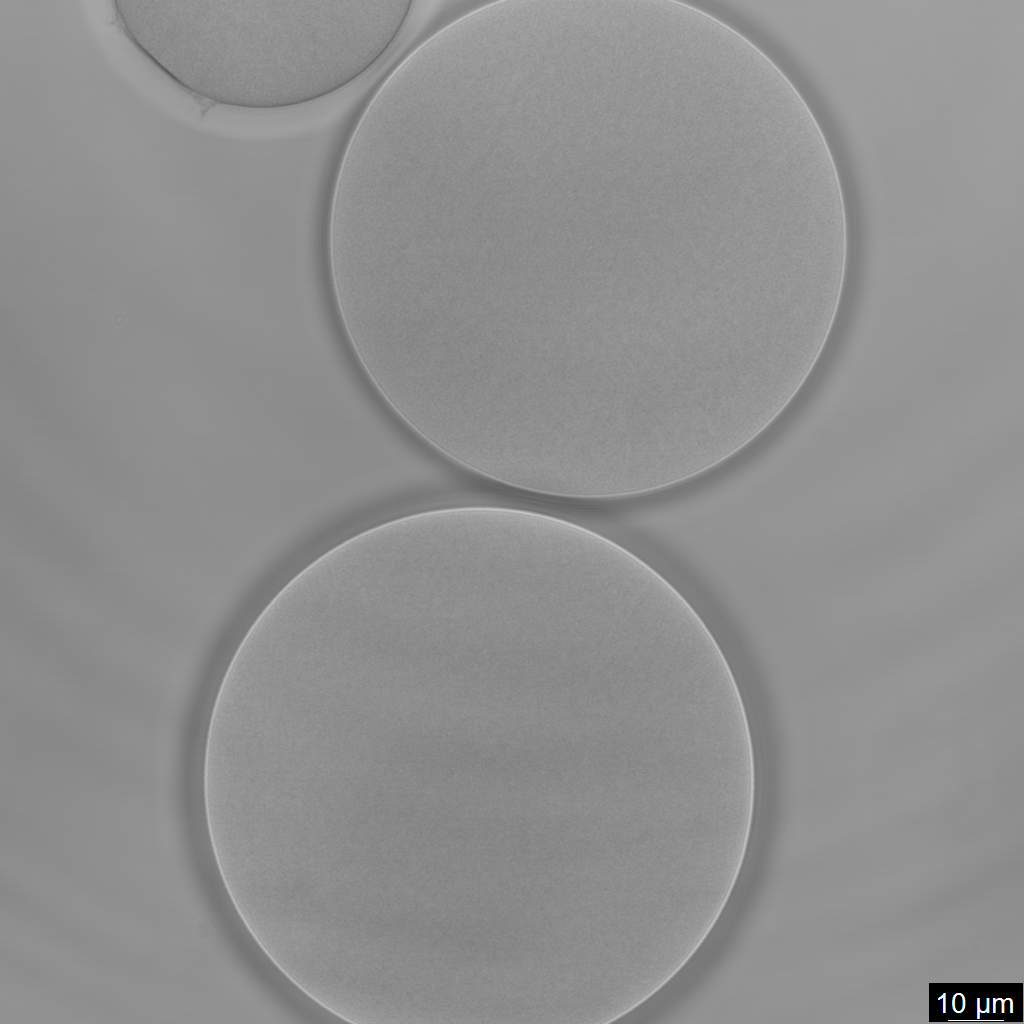

Supplement: Supplementary file 8 — Source data Fig. 5 [file 44319_2025_485_MOESM8_ESM.zip › Figure 5/5I/Fig_5I_╬öM_BF.tif]

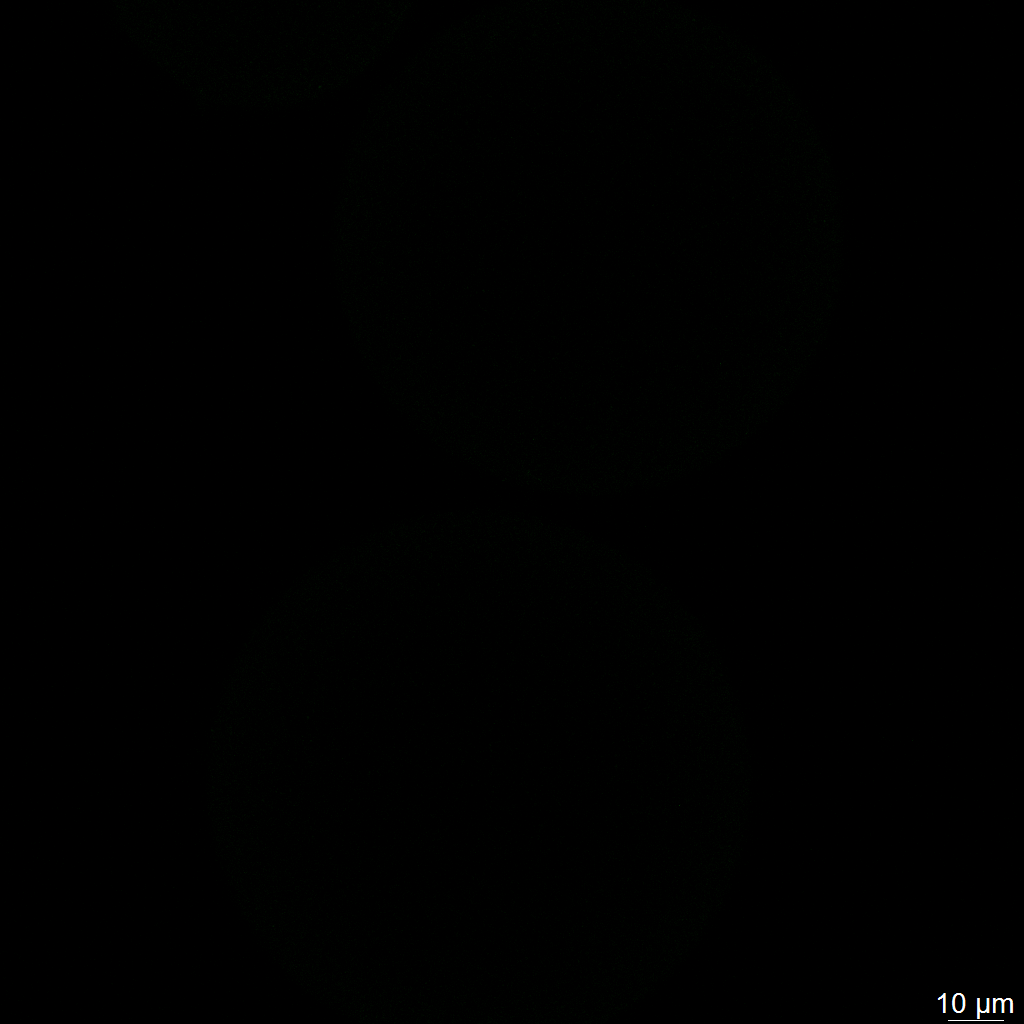

Supplement: Supplementary file 8 — Source data Fig. 5 [file 44319_2025_485_MOESM8_ESM.zip › Figure 5/5I/Fig_5I_╬öM_SAS_4_GFP.tif]

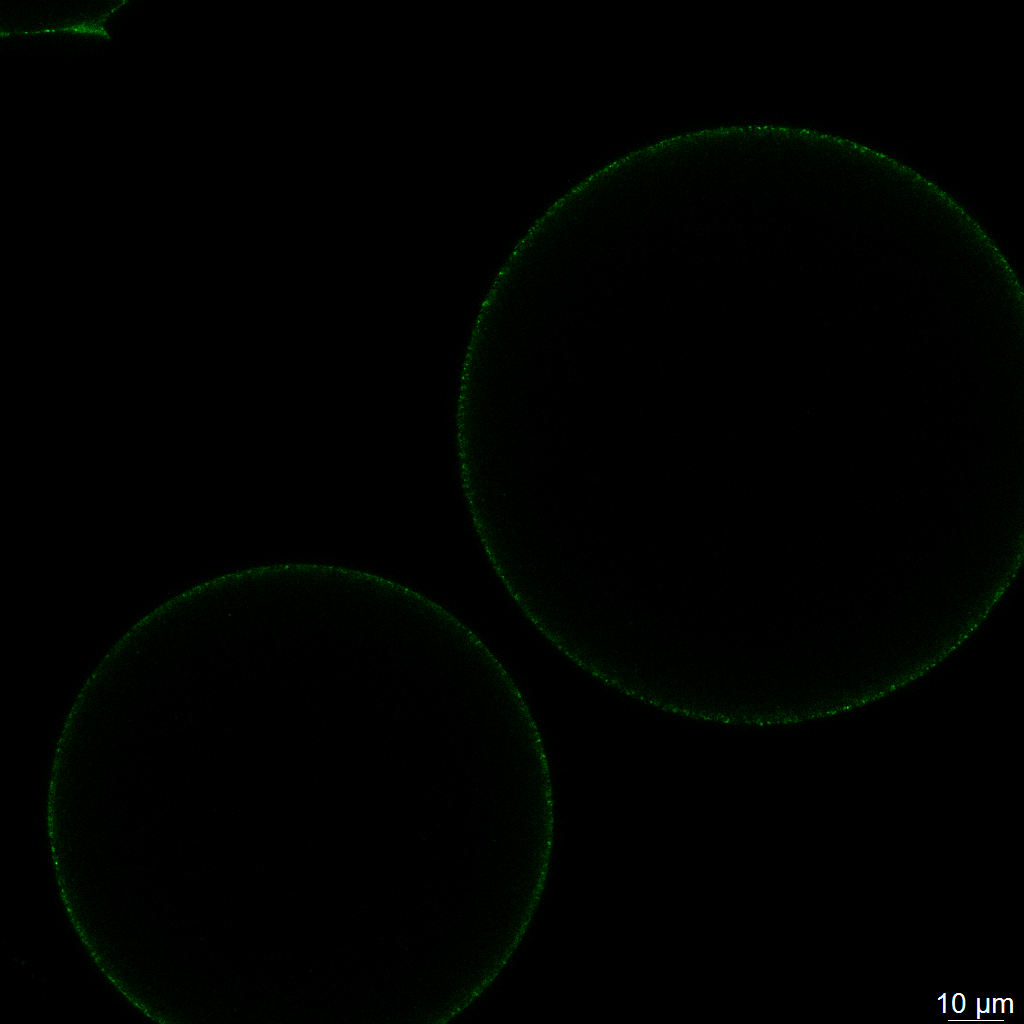

Supplement: Supplementary file 8 — Source data Fig. 5 [file 44319_2025_485_MOESM8_ESM.zip › Figure 5/5I/Fig_5I_fl_SAS_4_GFP_preP.tif]

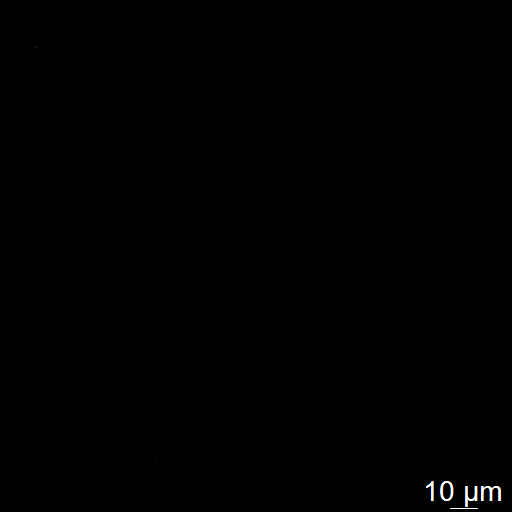

Supplement: Supplementary file 8 — Source data Fig. 5 [file 44319_2025_485_MOESM8_ESM.zip › Figure 5/5I/Fig_5I_╬öC_SAS_4_GFP.tif]

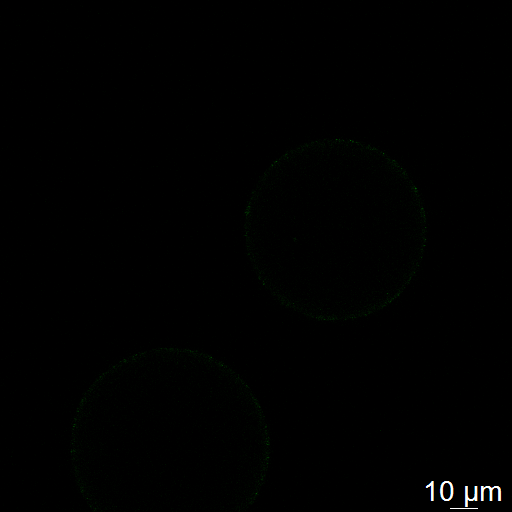

Supplement: Supplementary file 8 — Source data Fig. 5 [file 44319_2025_485_MOESM8_ESM.zip › Figure 5/5I/Fig_5I_M_BF_preP.tif]

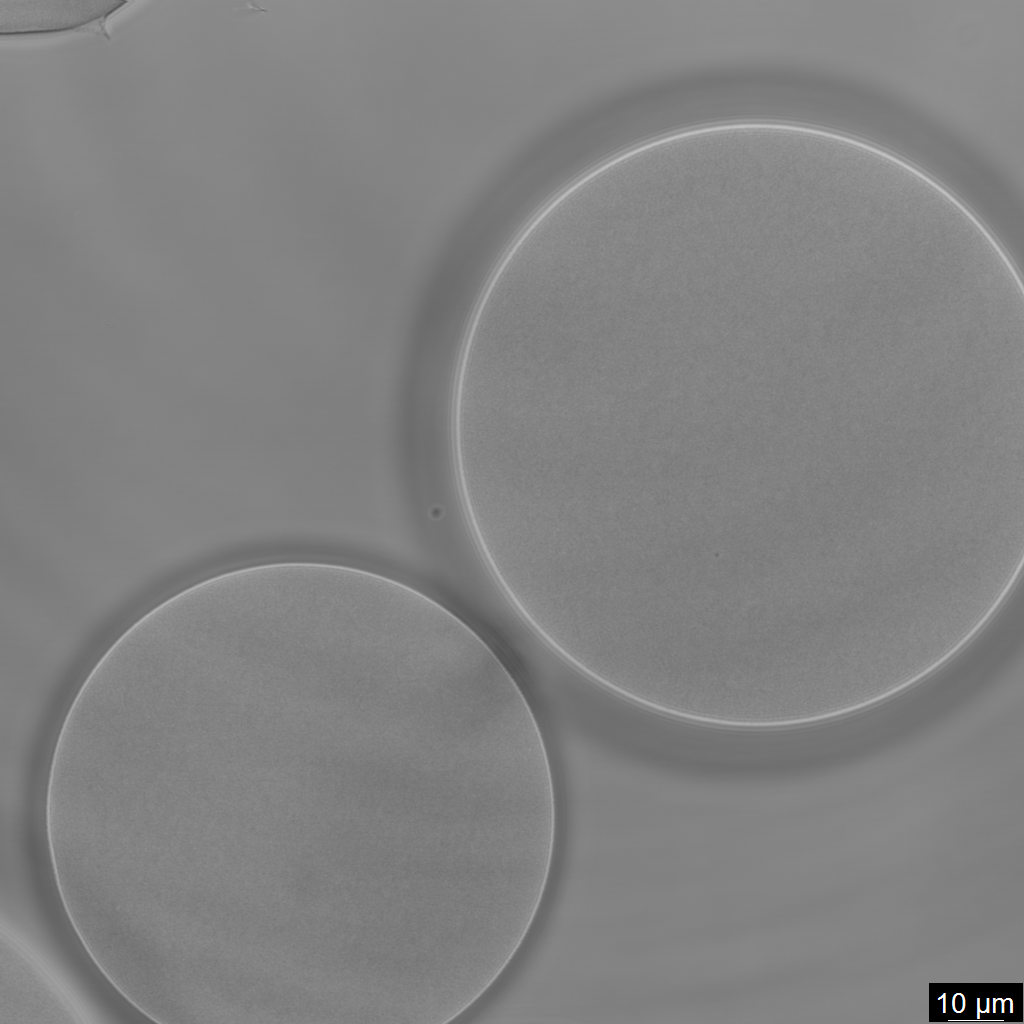

Supplement: Supplementary file 8 — Source data Fig. 5 [file 44319_2025_485_MOESM8_ESM.zip › Figure 5/5I/Fig_5I_FL_BF_preP.tif]

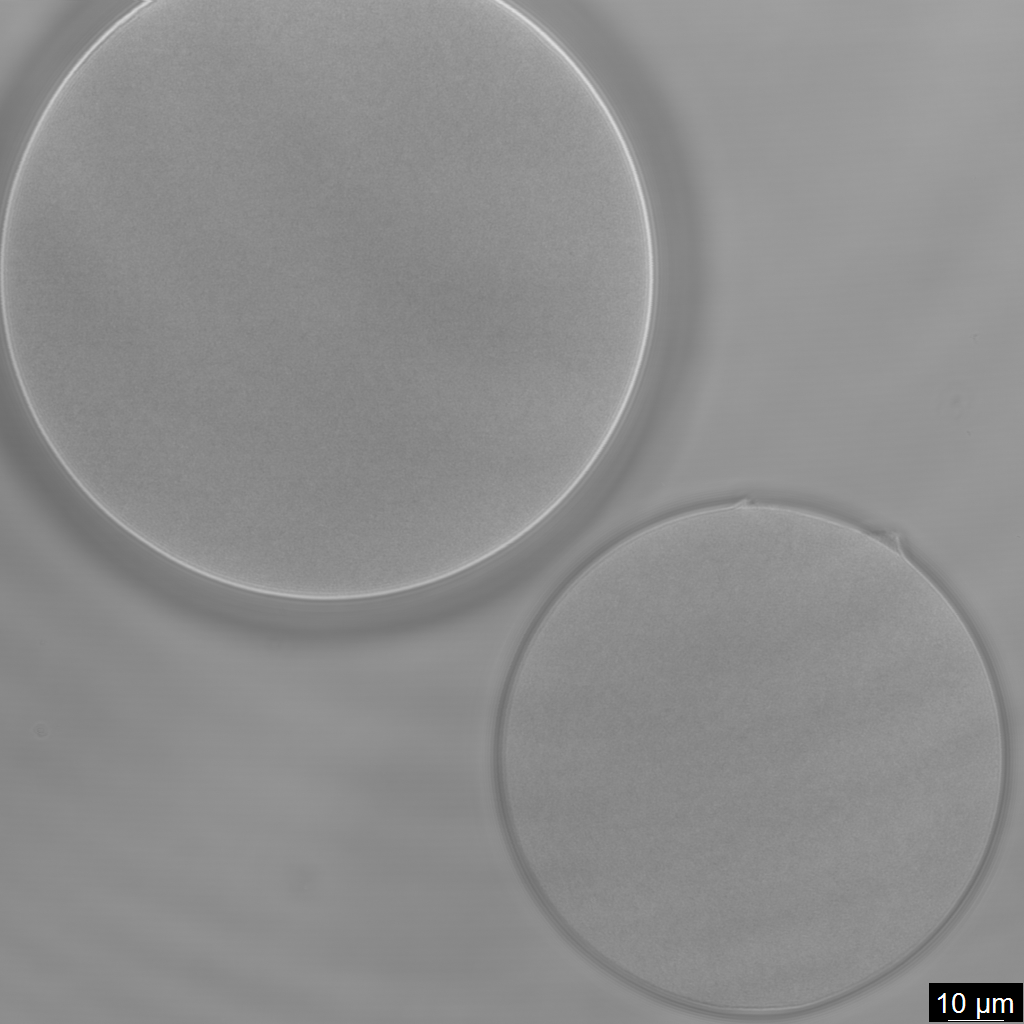

Supplement: Supplementary file 8 — Source data Fig. 5 [file 44319_2025_485_MOESM8_ESM.zip › Figure 5/5I/Fig_5I_N_BF.tif]

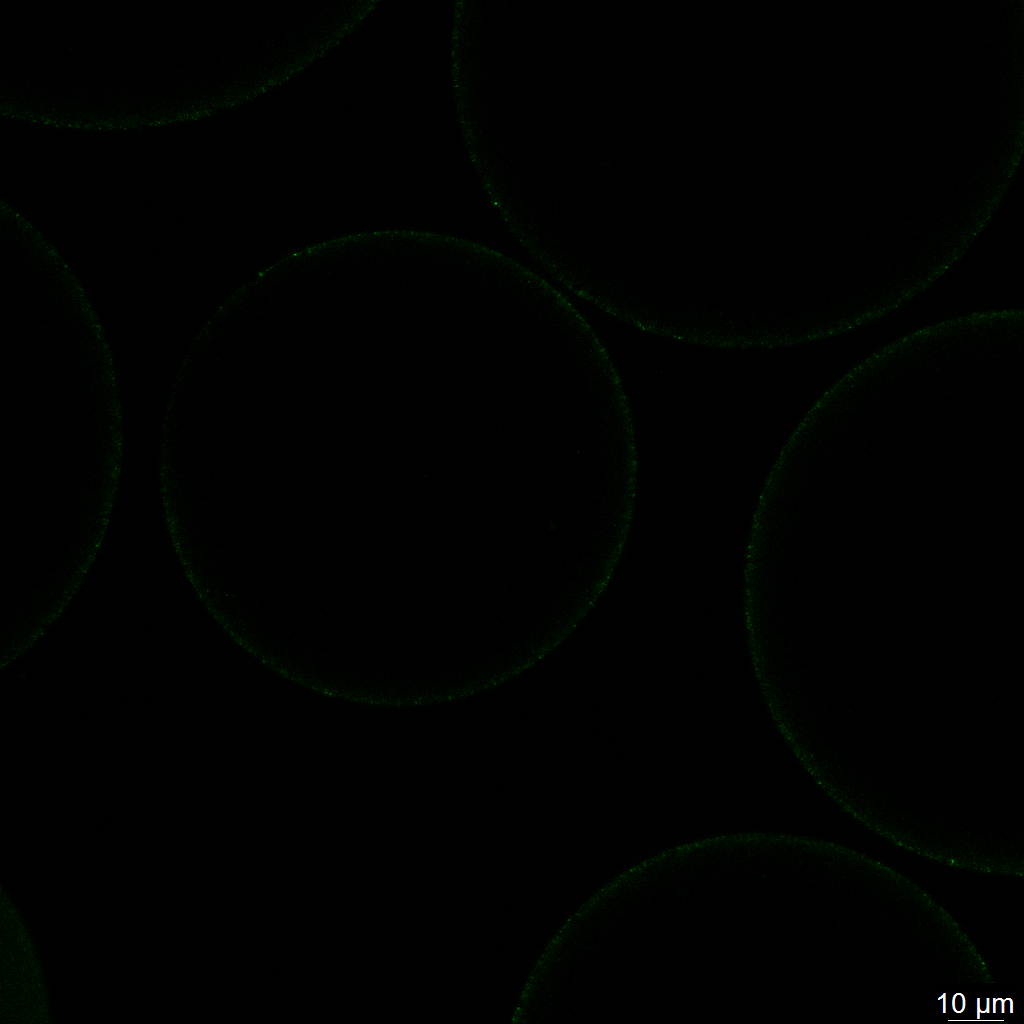

Supplement: Supplementary file 8 — Source data Fig. 5 [file 44319_2025_485_MOESM8_ESM.zip › Figure 5/5I/Fig_5I_╬öN_SAS_4_GFP_preP.tif]

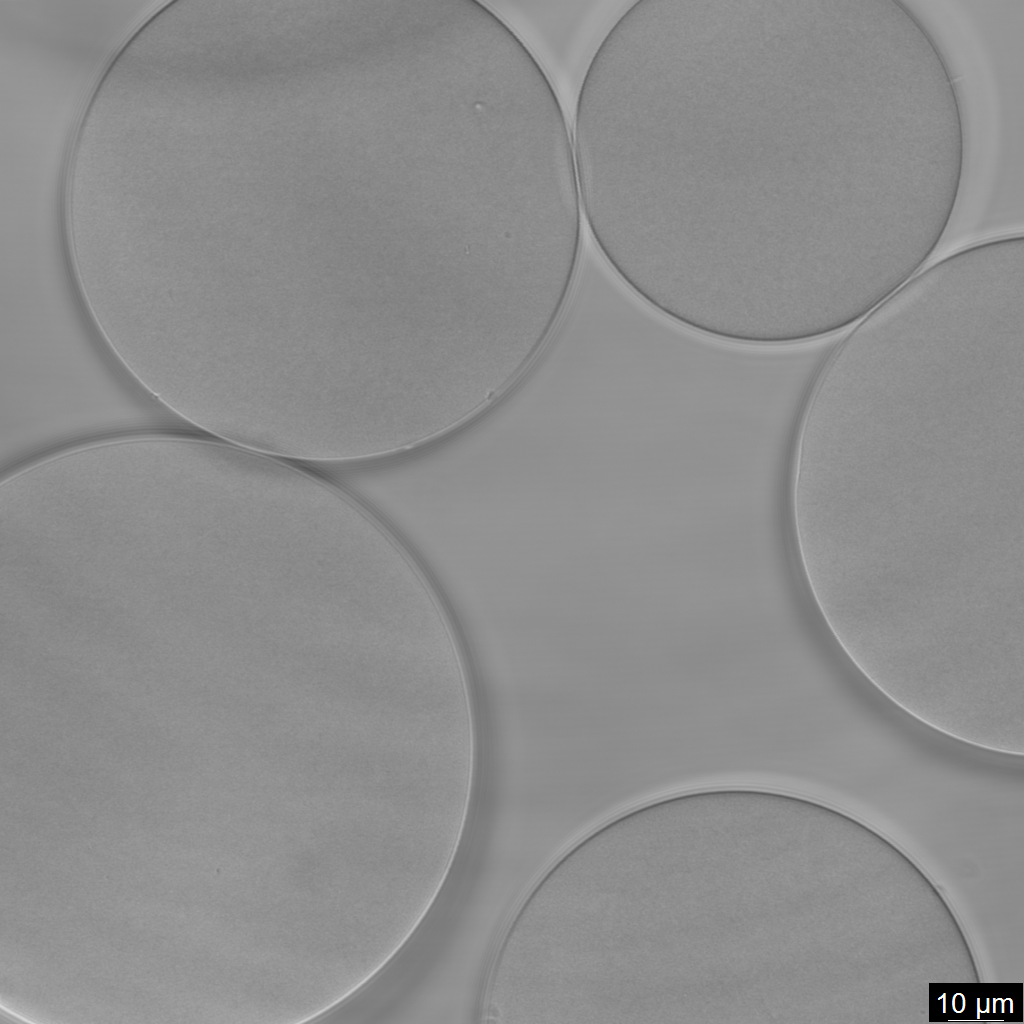

Supplement: Supplementary file 8 — Source data Fig. 5 [file 44319_2025_485_MOESM8_ESM.zip › Figure 5/5I/Fig_5I_╬öN_BF.tif]

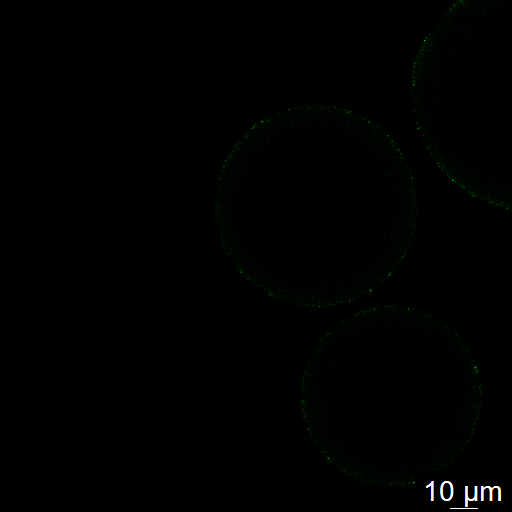

Supplement: Supplementary file 8 — Source data Fig. 5 [file 44319_2025_485_MOESM8_ESM.zip › Figure 5/5I/Fig_5I_M_SAS_4_GFP.tif]

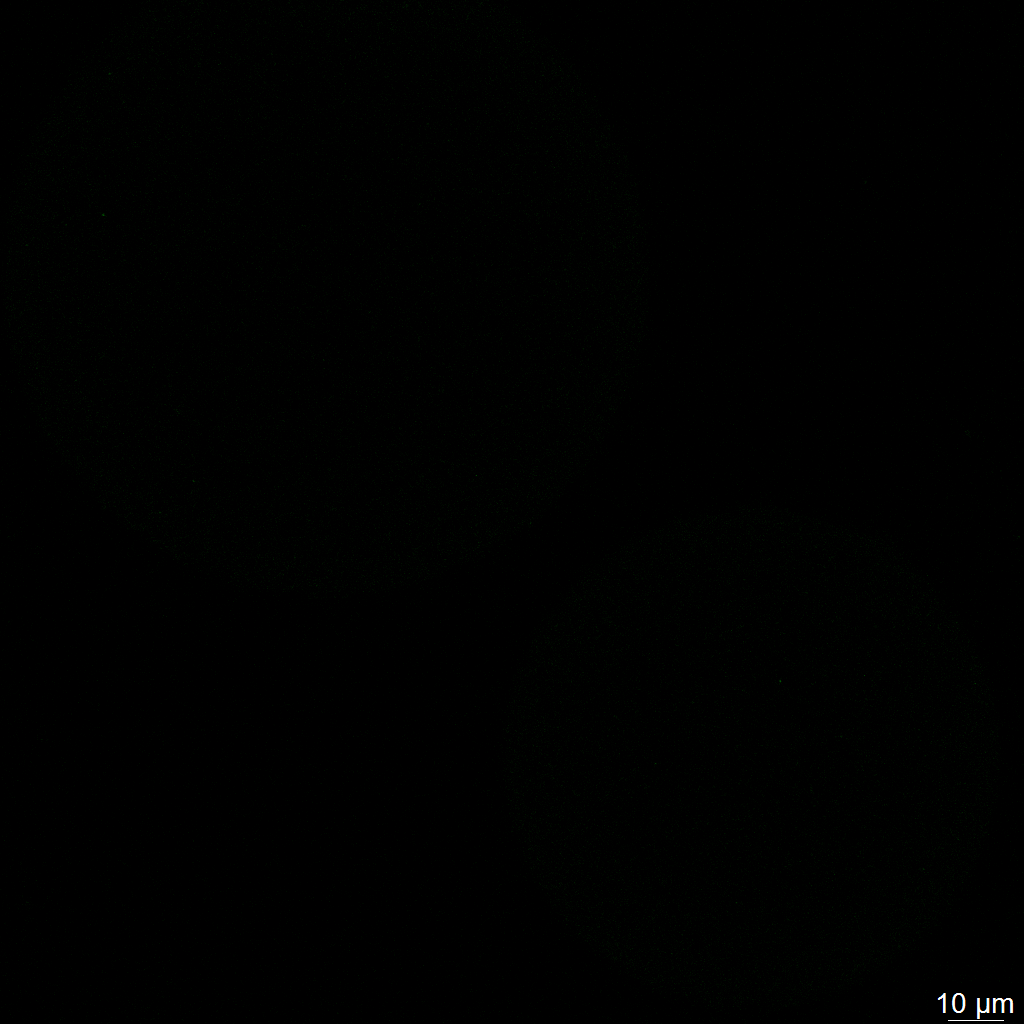

Supplement: Supplementary file 8 — Source data Fig. 5 [file 44319_2025_485_MOESM8_ESM.zip › Figure 5/5I/Fig_5I_N_SAS_4_GFP.tif]

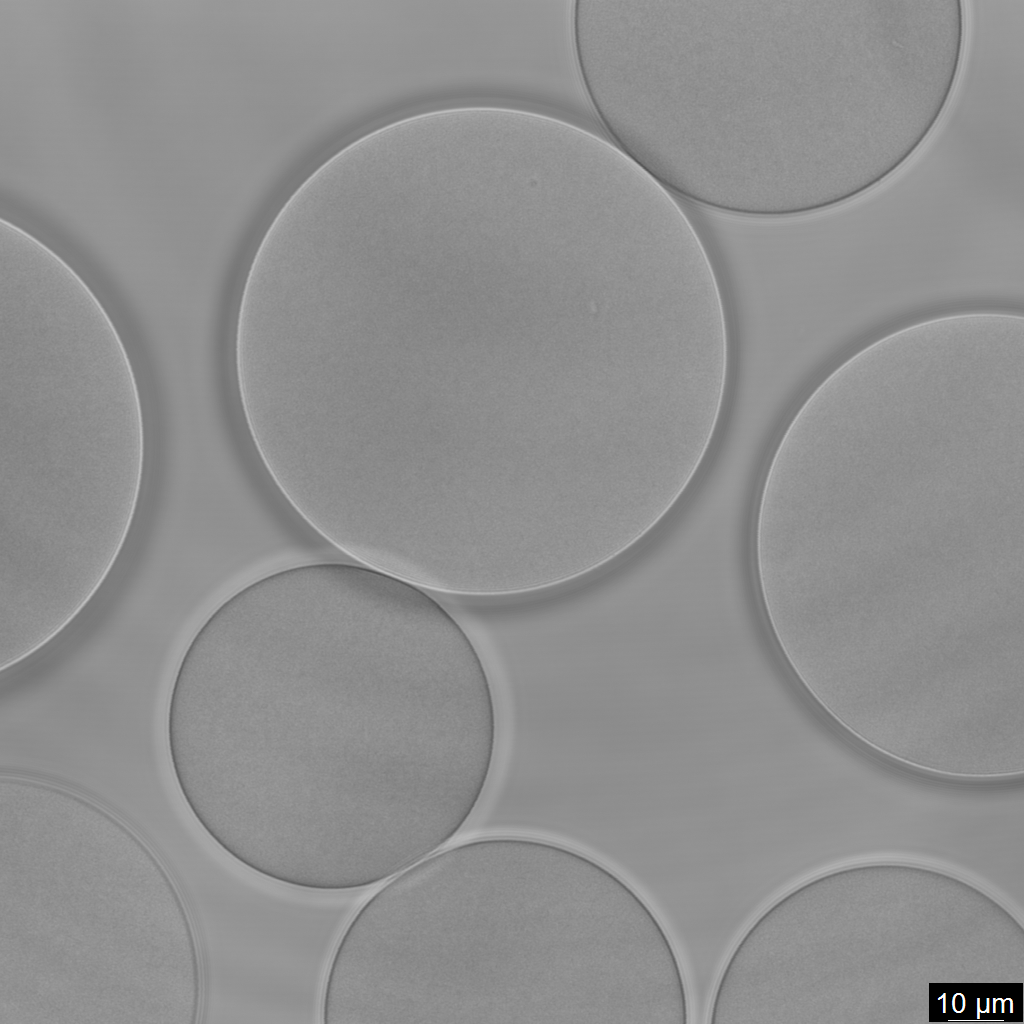

Supplement: Supplementary file 8 — Source data Fig. 5 [file 44319_2025_485_MOESM8_ESM.zip › Figure 5/5I/Fig_5I_FL_BF.tif]

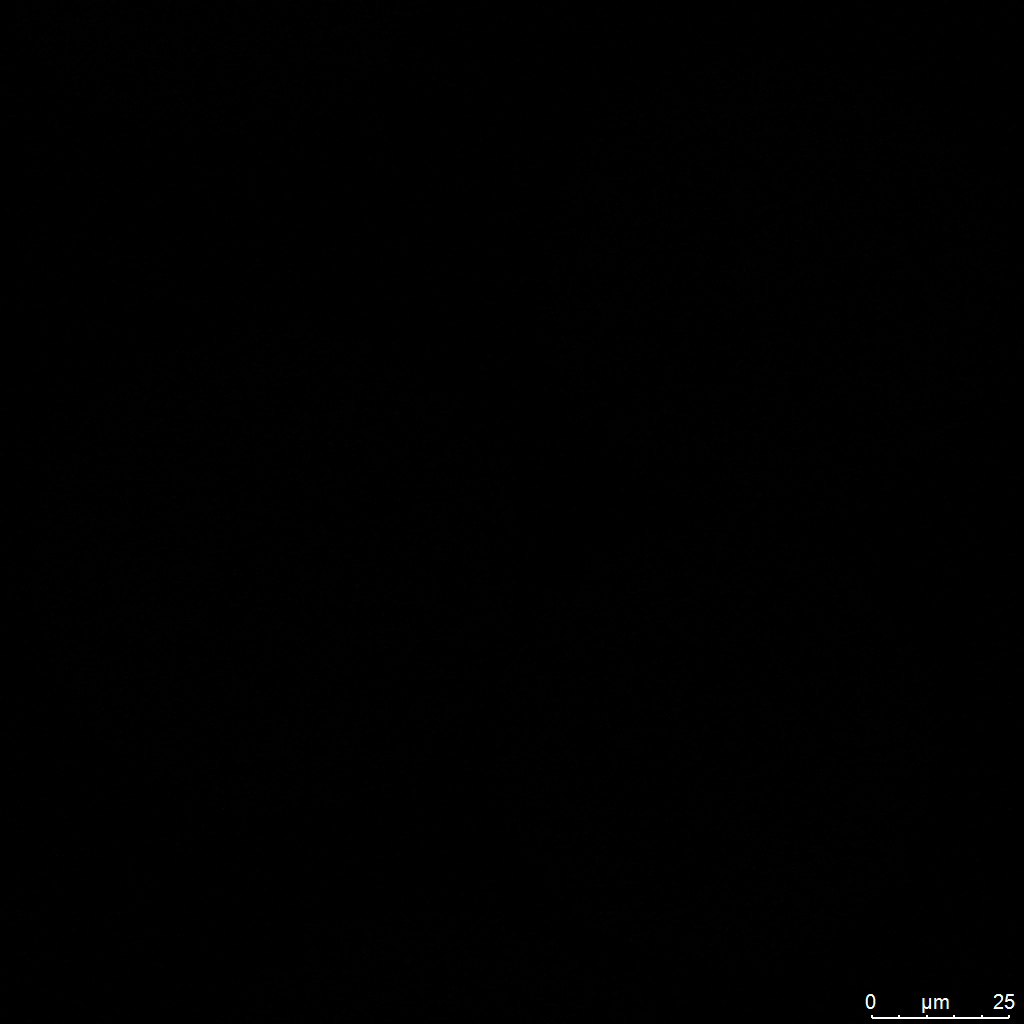

Supplement: Supplementary file 8 — Source data Fig. 5 [file 44319_2025_485_MOESM8_ESM.zip › Figure 5/5I/Fig_5I_GST_SAS_4_GFP.tif]

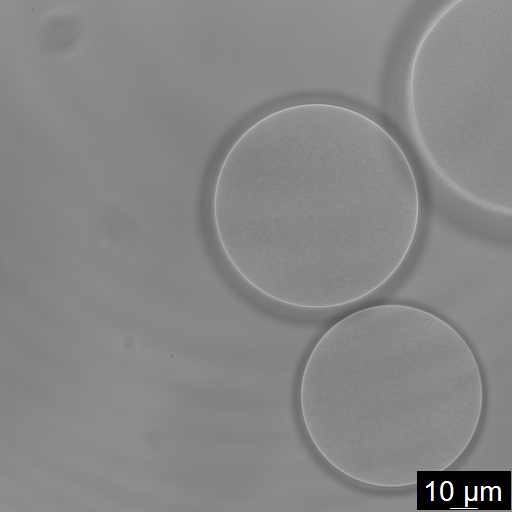

Supplement: Supplementary file 8 — Source data Fig. 5 [file 44319_2025_485_MOESM8_ESM.zip › Figure 5/5I/Fig_5I_M_BF.tif]

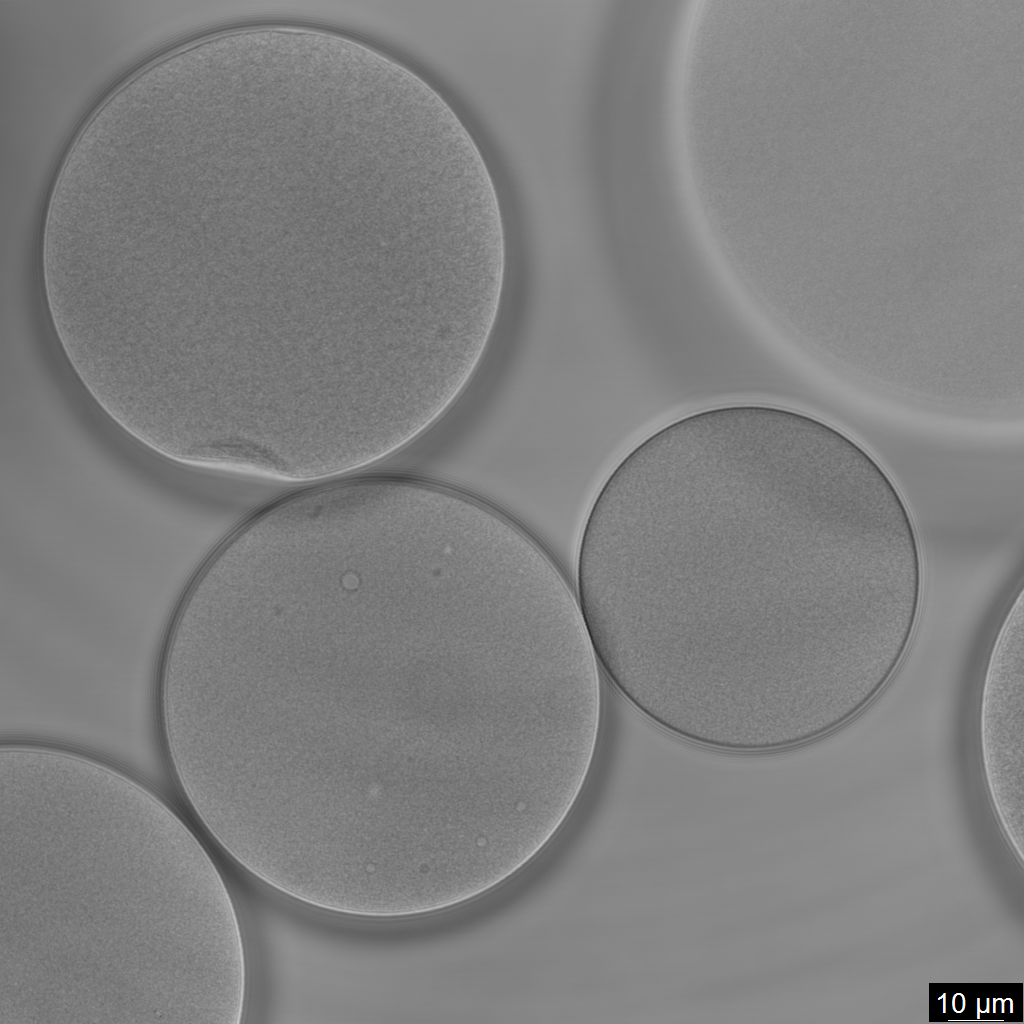

Supplement: Supplementary file 8 — Source data Fig. 5 [file 44319_2025_485_MOESM8_ESM.zip › Figure 5/5I/Fig_5I_C_BF.tif]

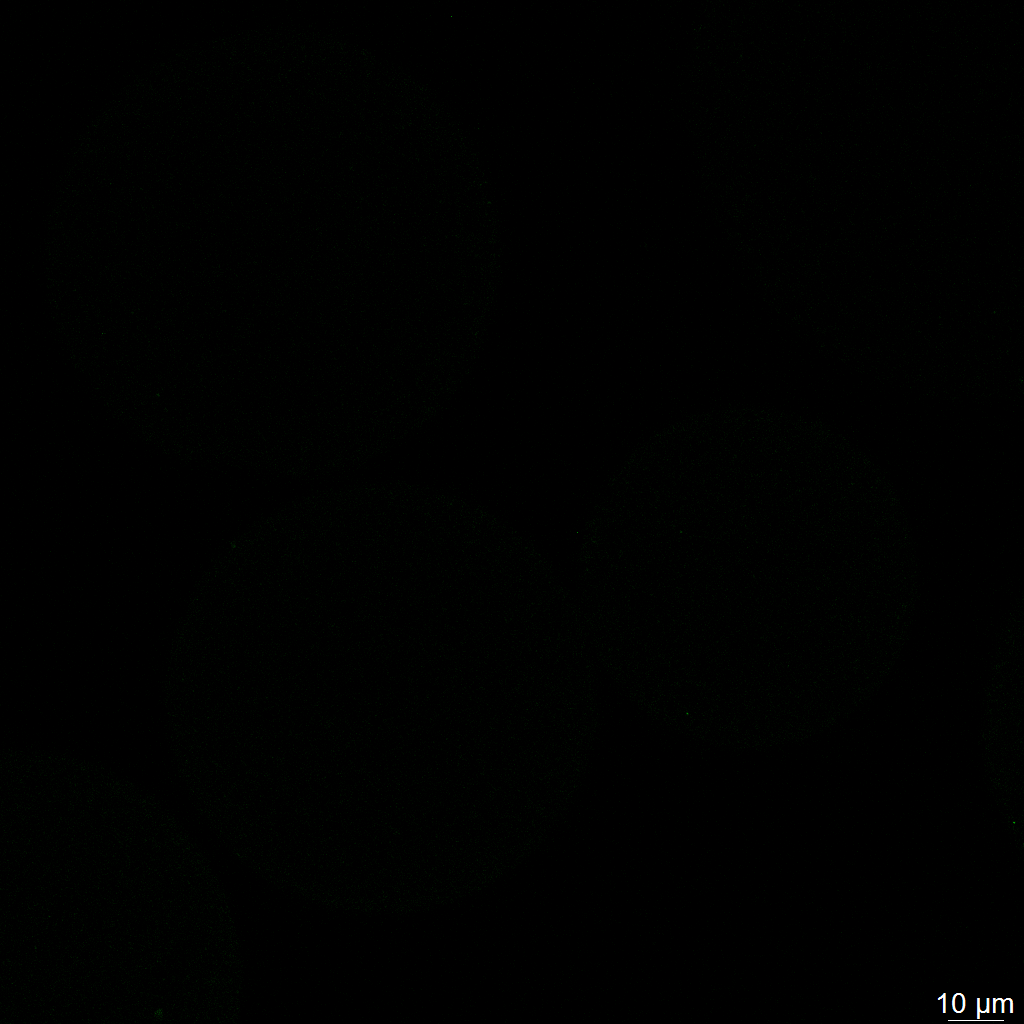

Supplement: Supplementary file 8 — Source data Fig. 5 [file 44319_2025_485_MOESM8_ESM.zip › Figure 5/5I/Fig_5I_C_SAS_4_GFP.tif]
