# Supplementary figures and images for: Dynamic SAS-6 phosphorylation aids centrosome duplication and elimination in C. elegans oogenesis (part 3 of 3)
Source: EMBO Rep. 2025 May 23;26(13):3411–44. doi: 10.1038/s44319-025-00485-7 (PMC12238530; doi:10.1038/s44319-025-00485-7)

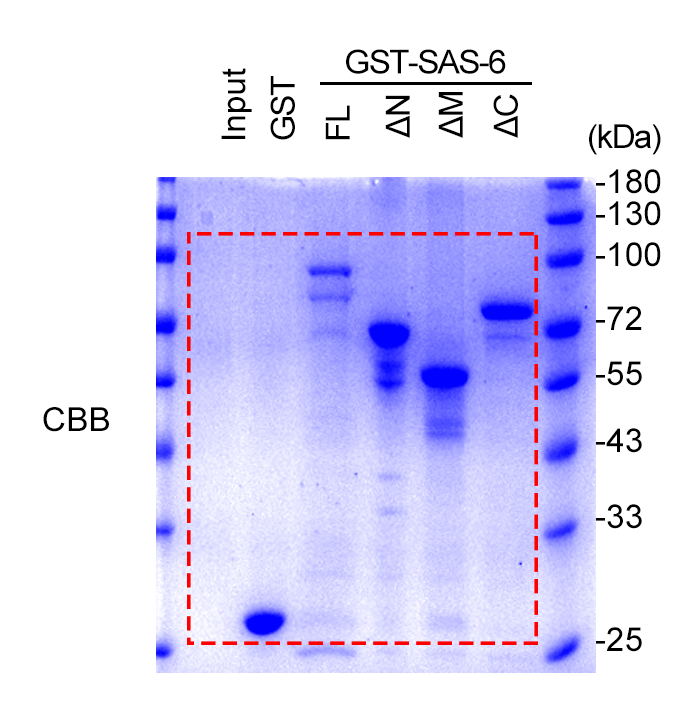

Supplement: Supplementary file 8 — Source data Fig. 5 [file 44319_2025_485_MOESM8_ESM.zip › Figure 5/5G/Fig_5G_CBB.tif]

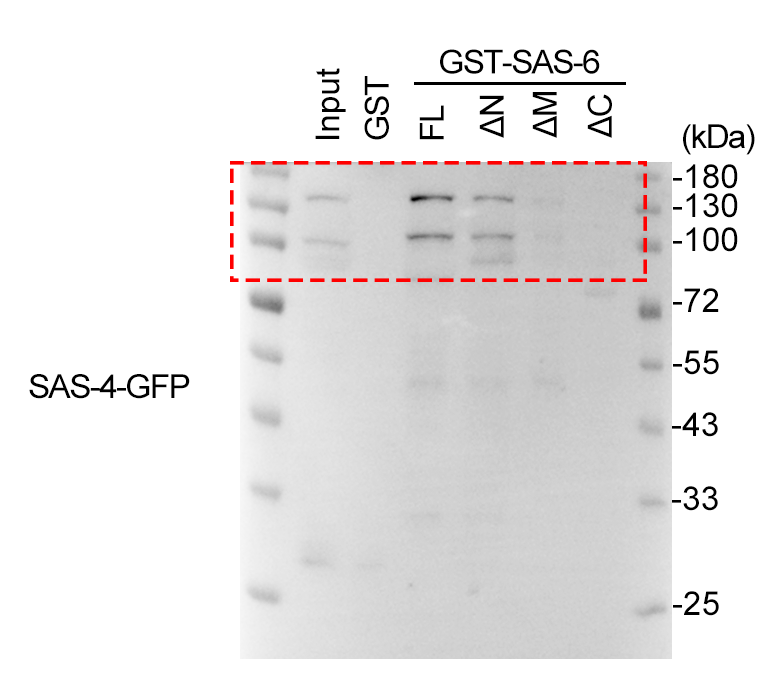

Supplement: Supplementary file 8 — Source data Fig. 5 [file 44319_2025_485_MOESM8_ESM.zip › Figure 5/5G/Fig_5G_SAS4_GFP.tif]

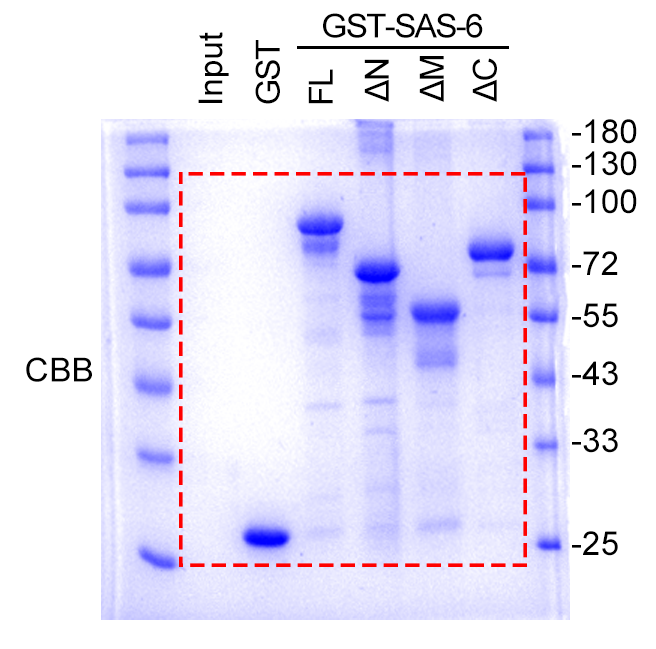

Supplement: Supplementary file 8 — Source data Fig. 5 [file 44319_2025_485_MOESM8_ESM.zip › Figure 5/5A/Fig_5A_CBB.tif]

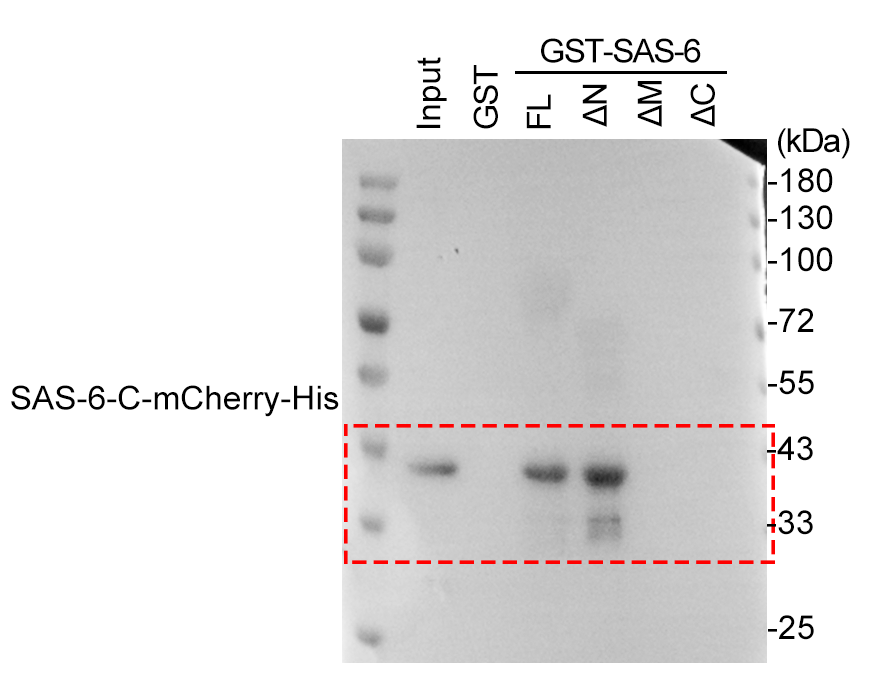

Supplement: Supplementary file 8 — Source data Fig. 5 [file 44319_2025_485_MOESM8_ESM.zip › Figure 5/5A/Fig_5A_SAS6_C_mCherry.tif]

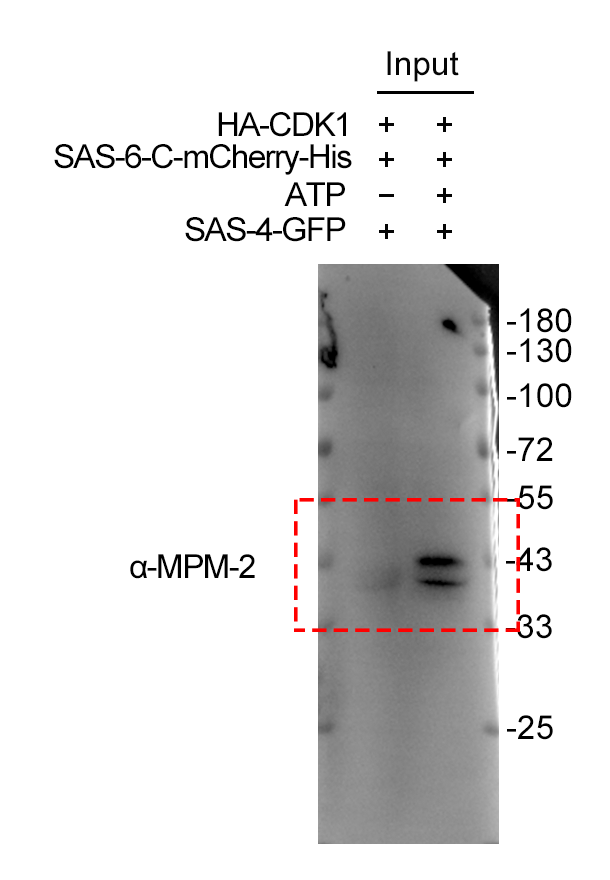

Supplement: Supplementary file 8 — Source data Fig. 5 [file 44319_2025_485_MOESM8_ESM.zip › Figure 5/5H/Fig_5H_MPM2.tif]

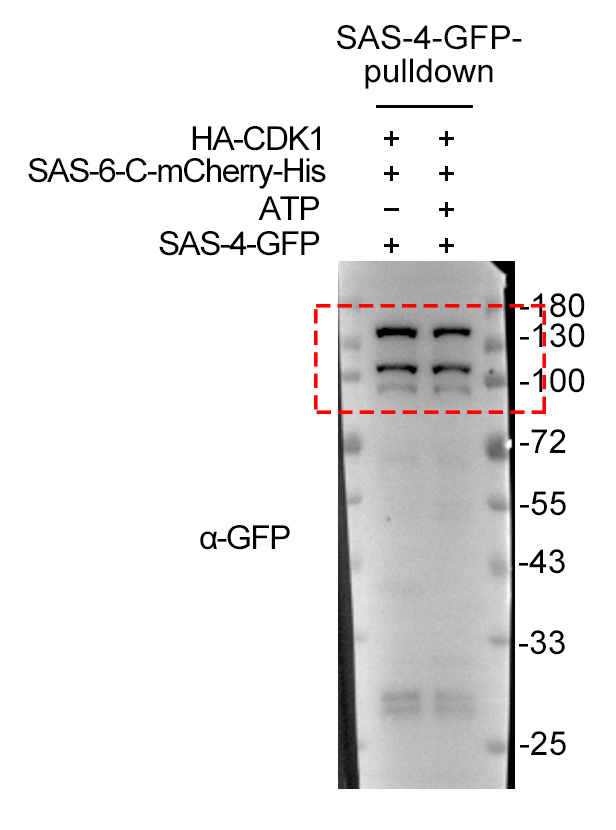

Supplement: Supplementary file 8 — Source data Fig. 5 [file 44319_2025_485_MOESM8_ESM.zip › Figure 5/5H/Fig_5H_GFP.tif]

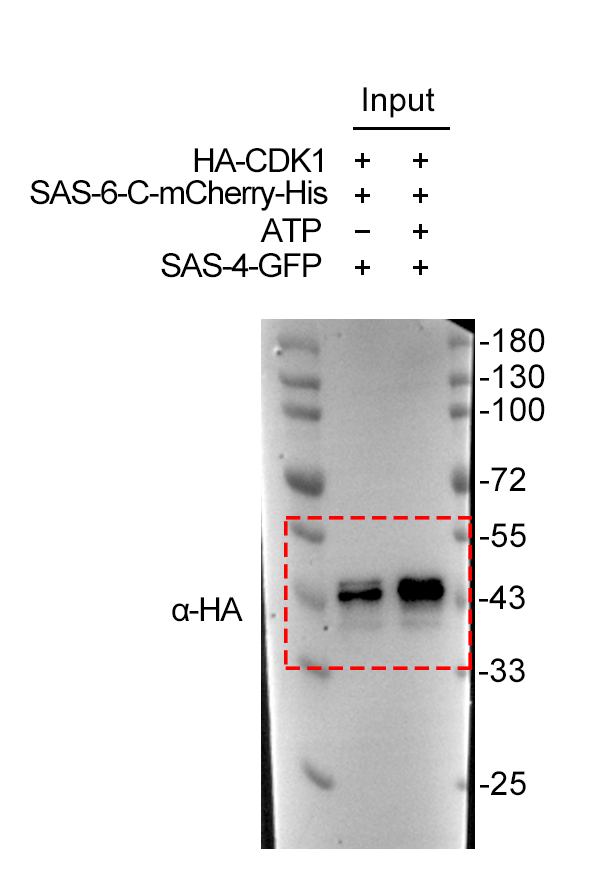

Supplement: Supplementary file 8 — Source data Fig. 5 [file 44319_2025_485_MOESM8_ESM.zip › Figure 5/5H/Fig_5H_HA.tif]

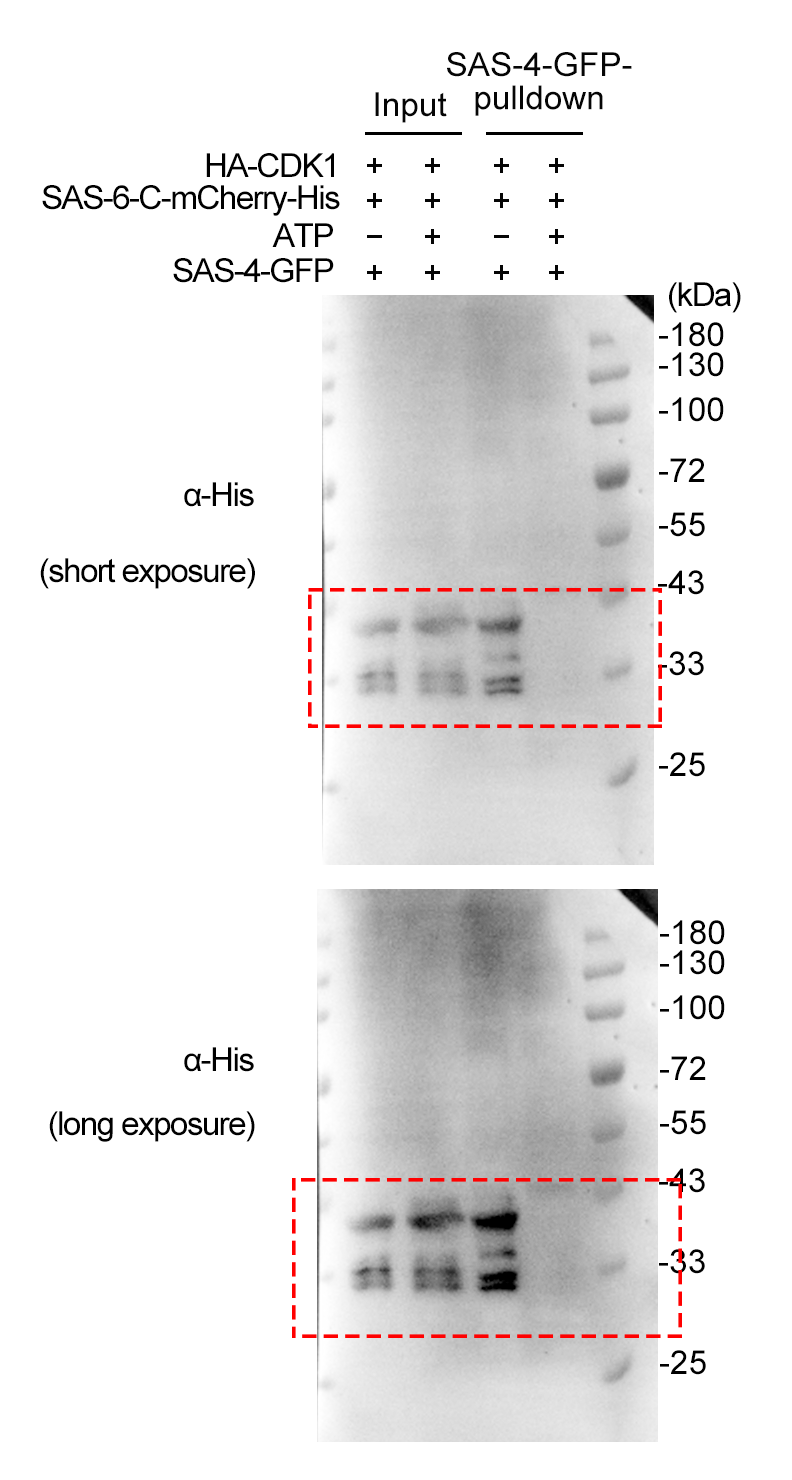

Supplement: Supplementary file 8 — Source data Fig. 5 [file 44319_2025_485_MOESM8_ESM.zip › Figure 5/5H/Fig_5H_His.tif]

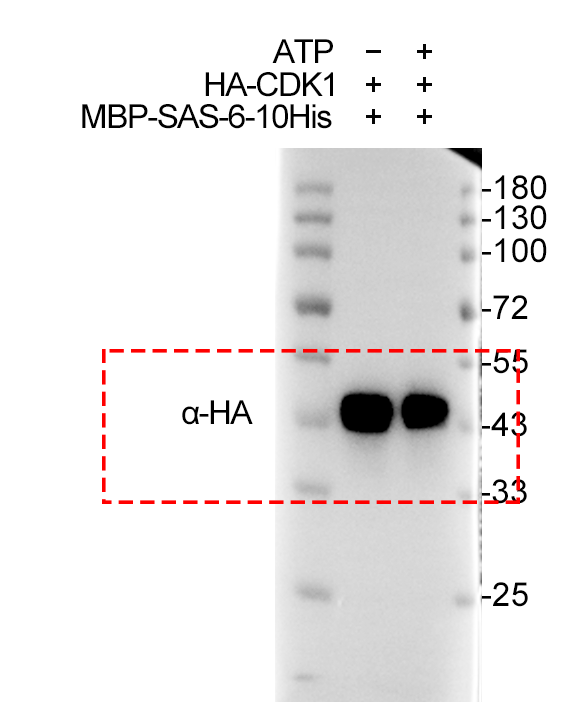

Supplement: Supplementary file 8 — Source data Fig. 5 [file 44319_2025_485_MOESM8_ESM.zip › Figure 5/5C/Fig_5C_HA.tif]

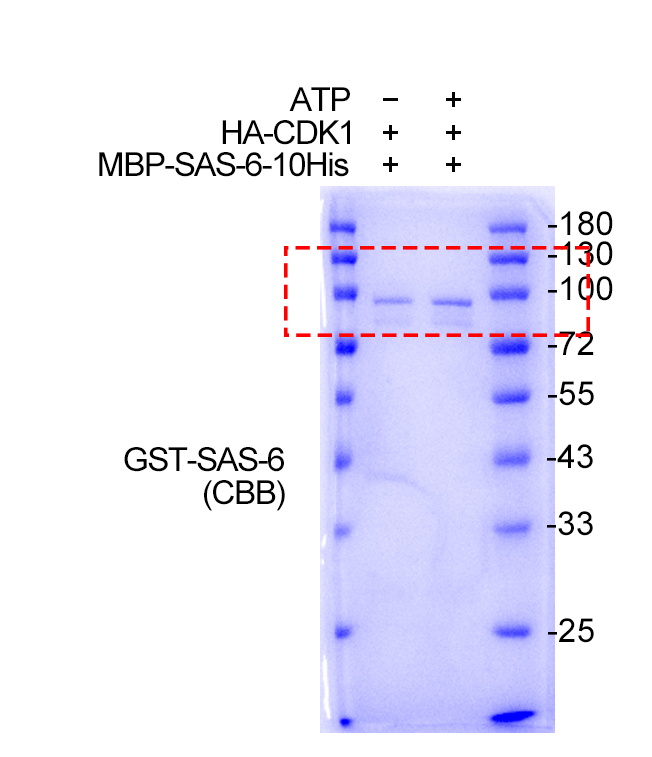

Supplement: Supplementary file 8 — Source data Fig. 5 [file 44319_2025_485_MOESM8_ESM.zip › Figure 5/5C/Fig_5C_CBB.tif]

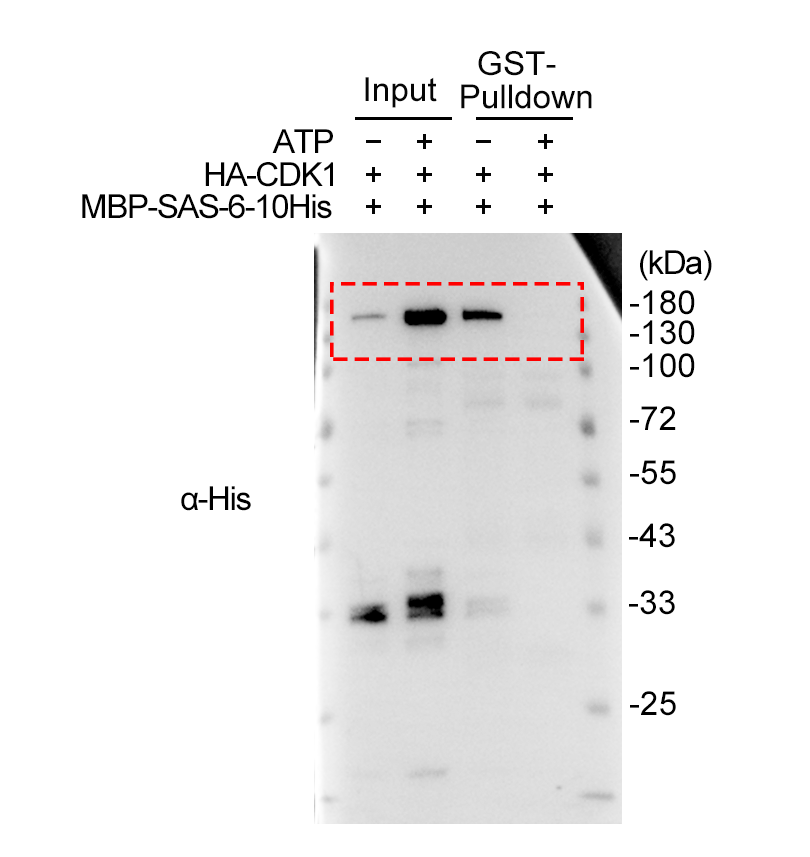

Supplement: Supplementary file 8 — Source data Fig. 5 [file 44319_2025_485_MOESM8_ESM.zip › Figure 5/5C/Fig_5C_His.tif]

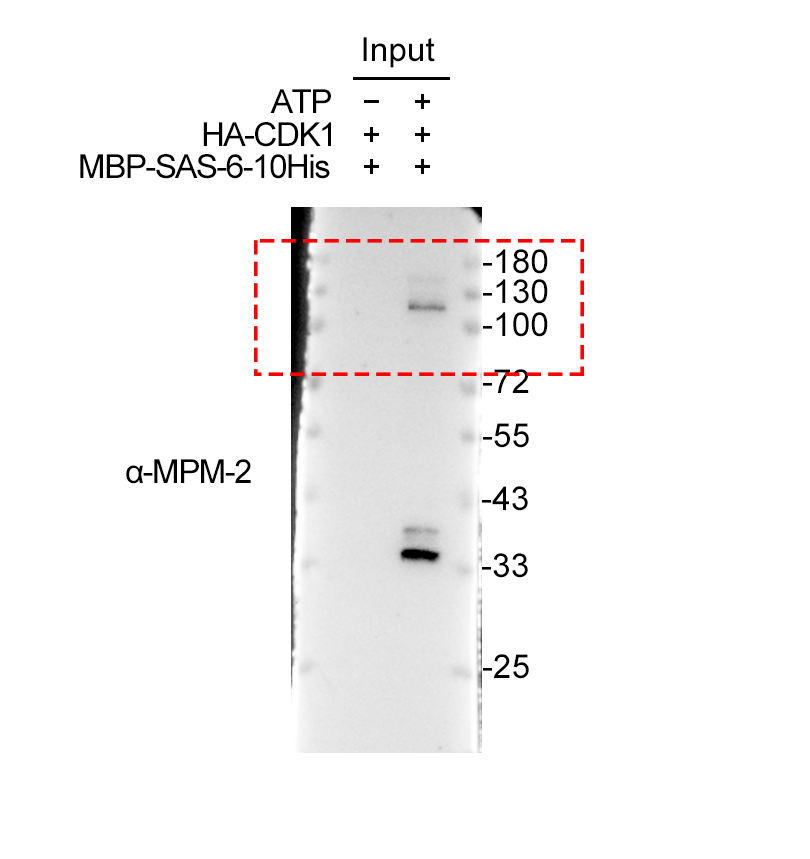

Supplement: Supplementary file 8 — Source data Fig. 5 [file 44319_2025_485_MOESM8_ESM.zip › Figure 5/5C/Fig_5C_MPM2.tif]

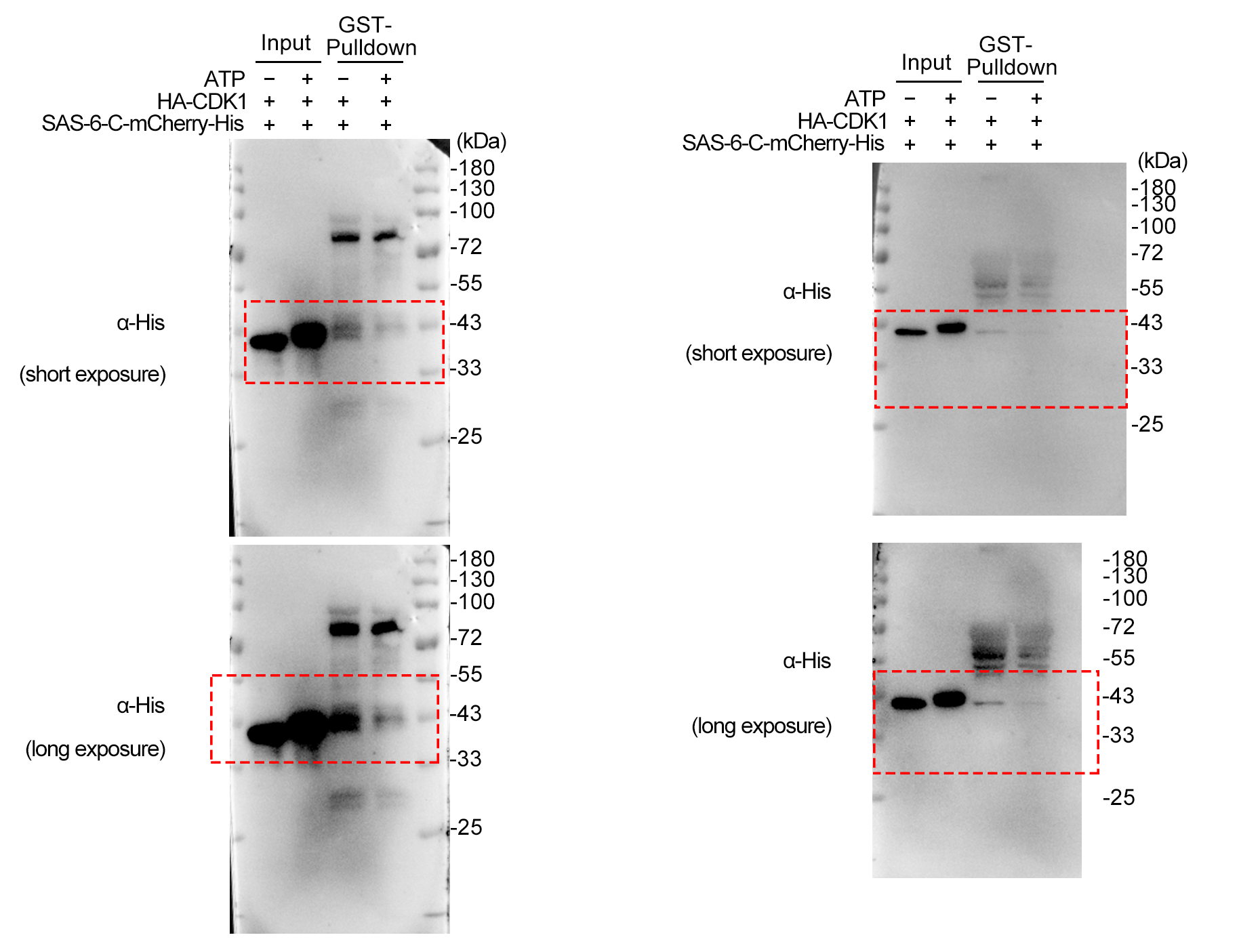

Supplement: Supplementary file 8 — Source data Fig. 5 [file 44319_2025_485_MOESM8_ESM.zip › Figure 5/5D/Fig_5D_His.tif]

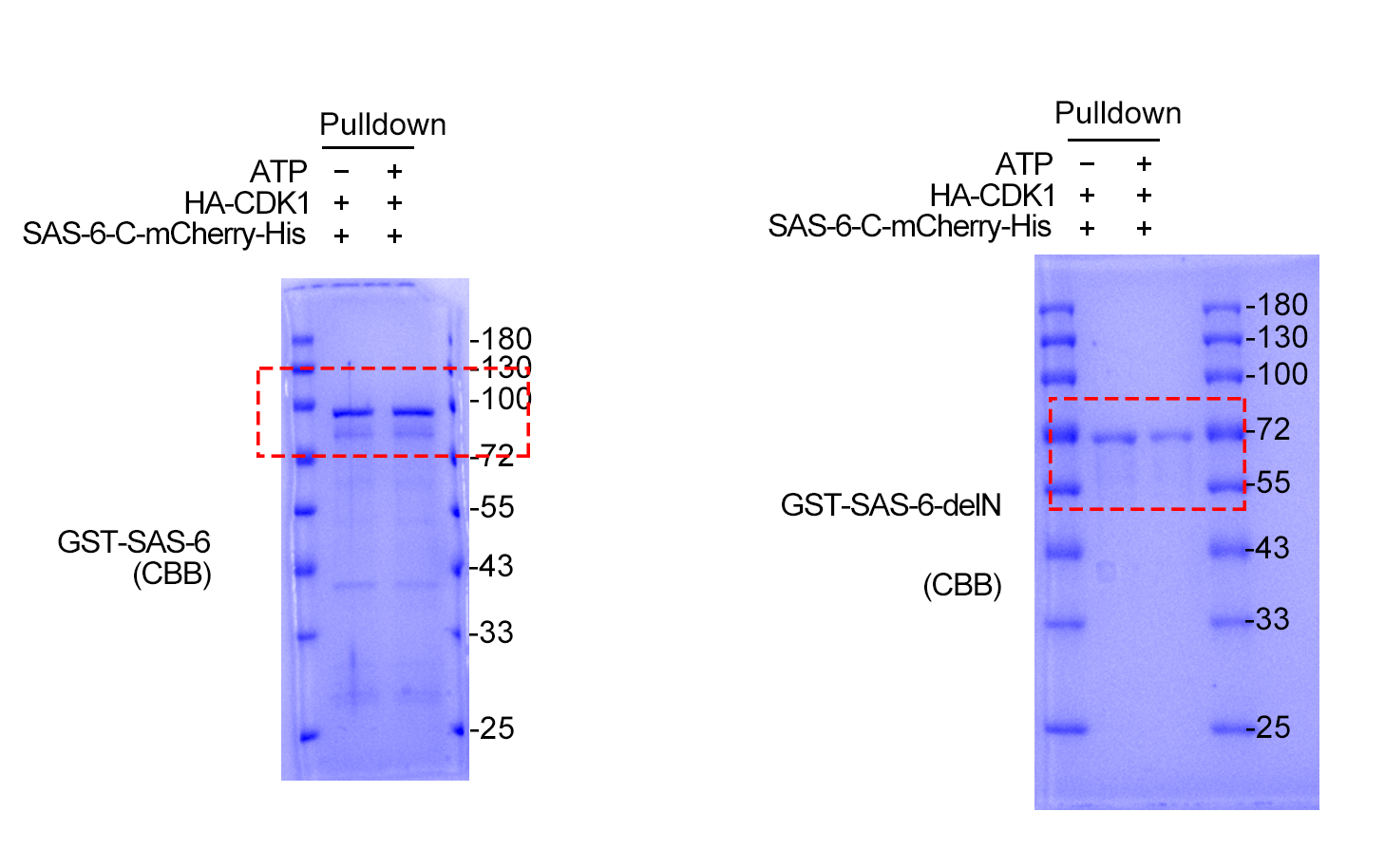

Supplement: Supplementary file 8 — Source data Fig. 5 [file 44319_2025_485_MOESM8_ESM.zip › Figure 5/5D/Fig_5D_CBB.tif]

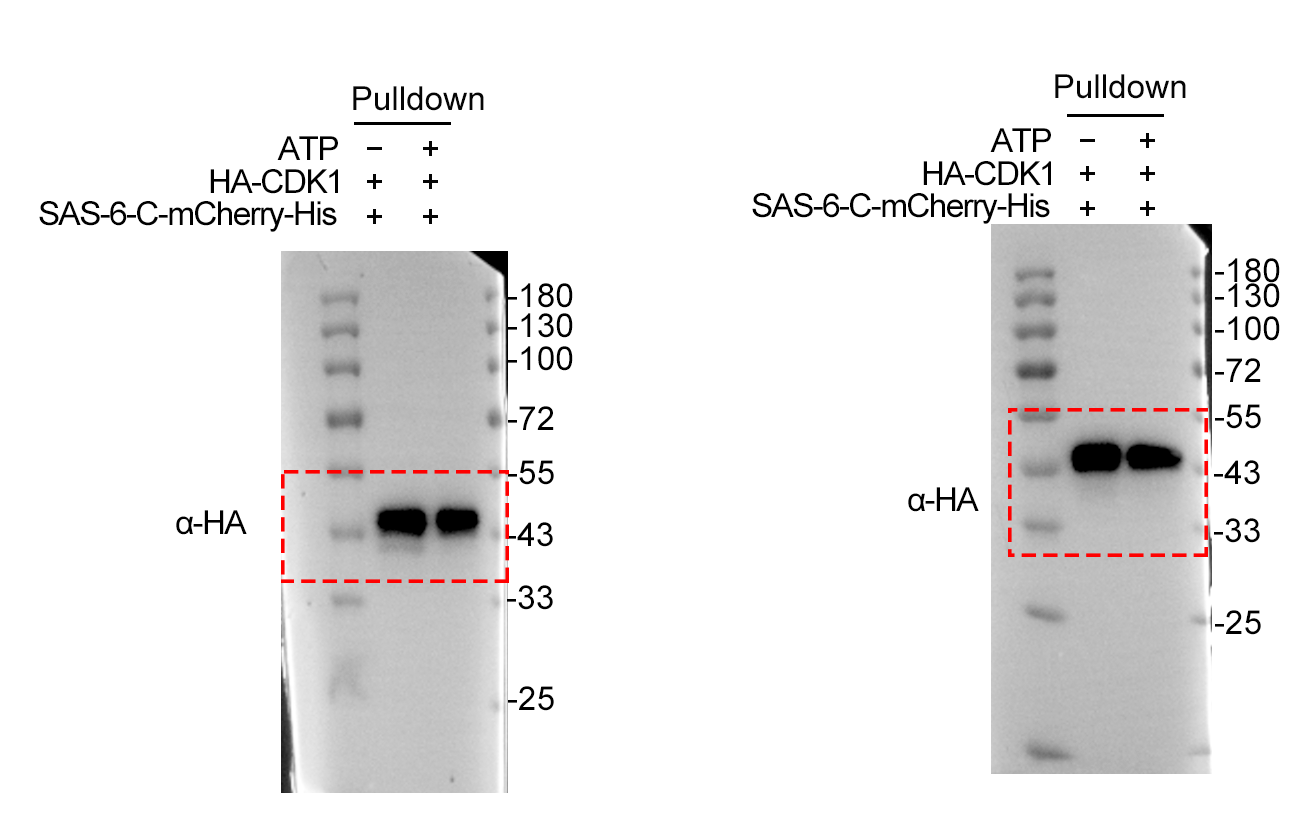

Supplement: Supplementary file 8 — Source data Fig. 5 [file 44319_2025_485_MOESM8_ESM.zip › Figure 5/5D/Fig_5D_HA.tif]

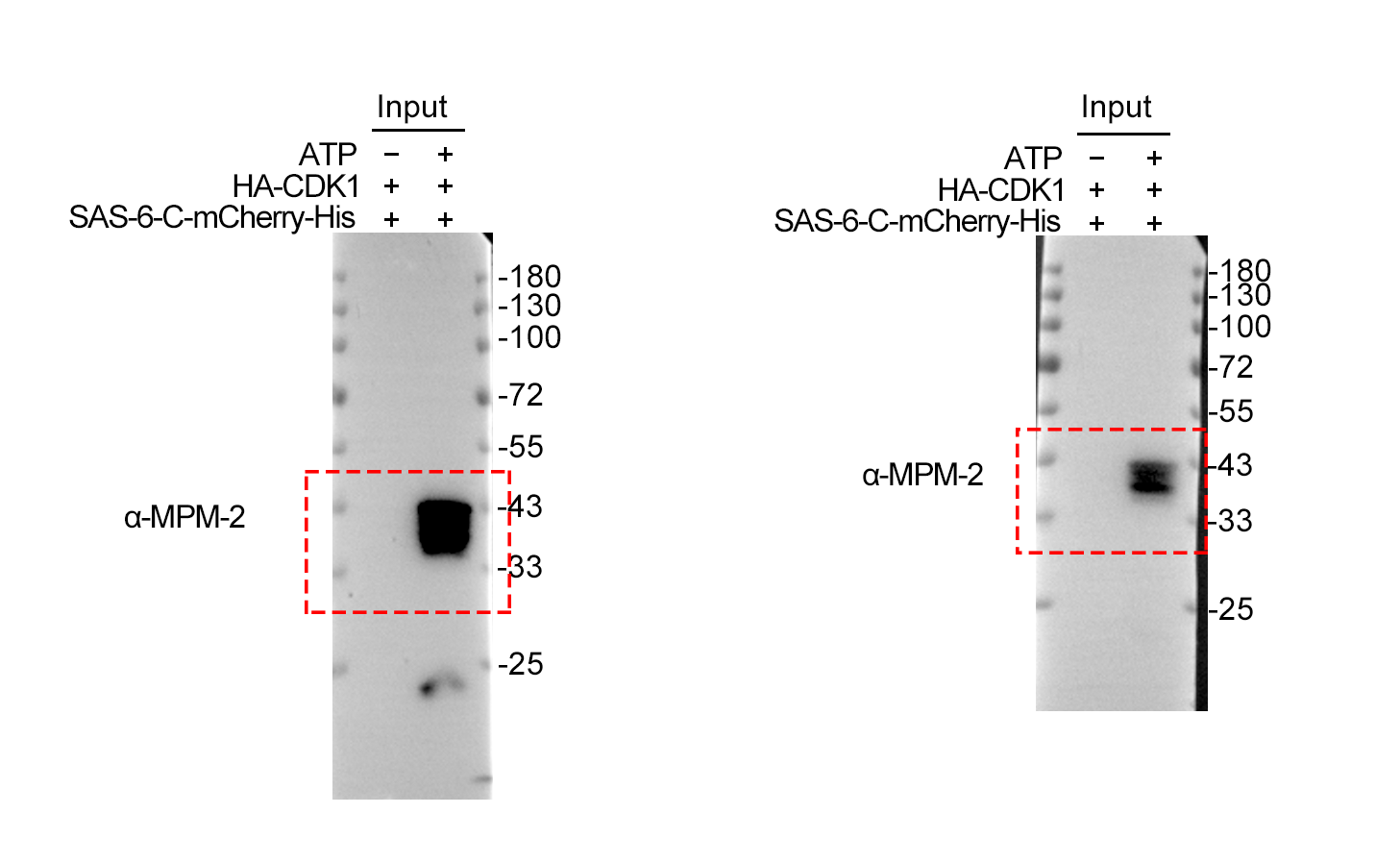

Supplement: Supplementary file 8 — Source data Fig. 5 [file 44319_2025_485_MOESM8_ESM.zip › Figure 5/5D/Fig_5D_MPM2.tif]

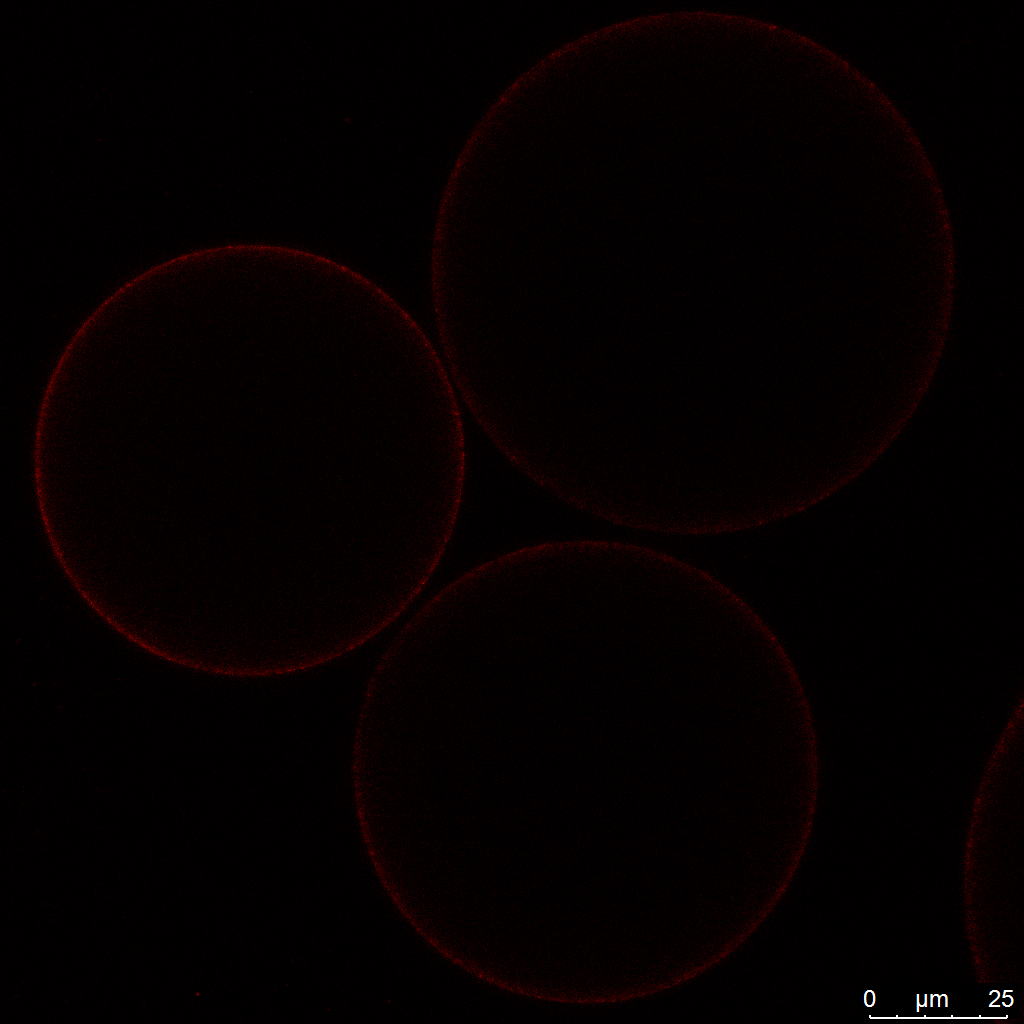

Supplement: Supplementary file 8 — Source data Fig. 5 [file 44319_2025_485_MOESM8_ESM.zip › Figure 5/5E/Fig_5E_GST_SAS_6_FL_mCherry_preP.tif]

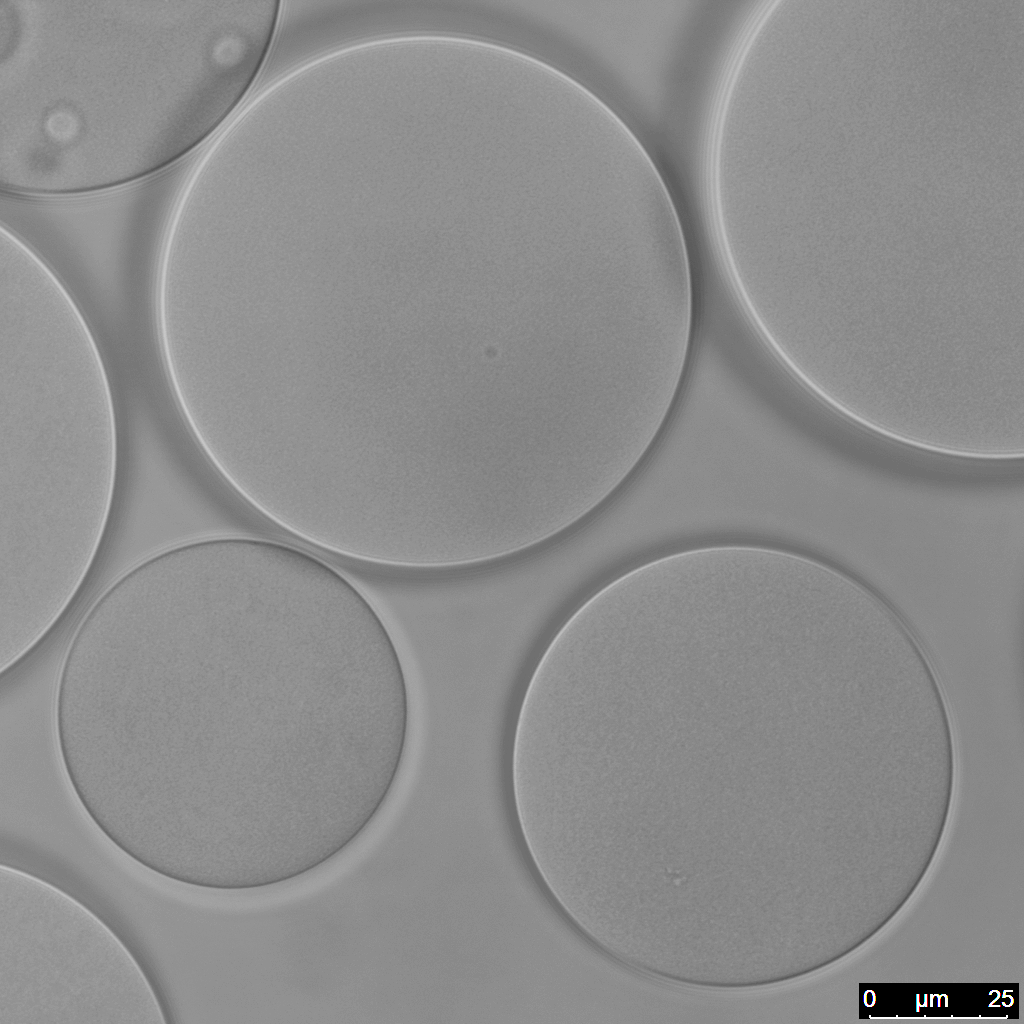

Supplement: Supplementary file 8 — Source data Fig. 5 [file 44319_2025_485_MOESM8_ESM.zip › Figure 5/5E/Fig_5E_GST_SAS_6_FL_BF.tif]

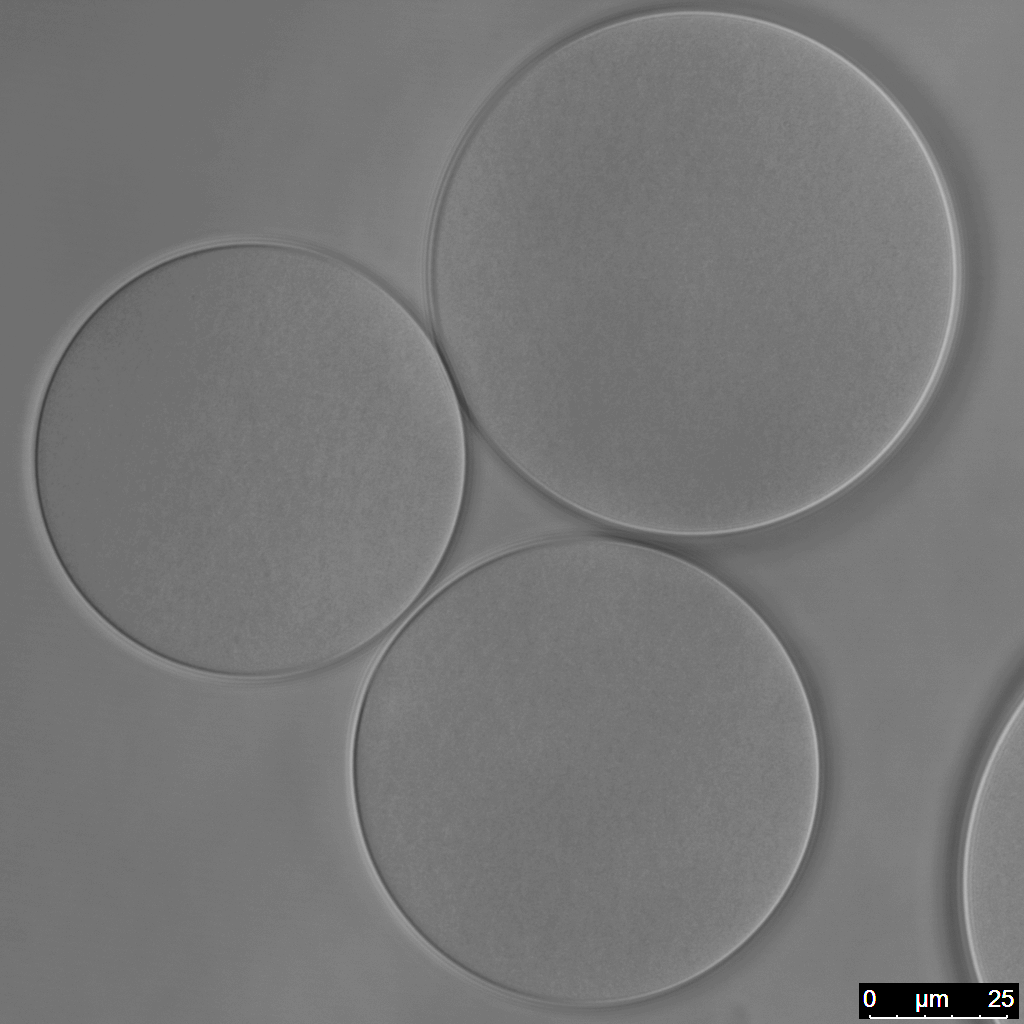

Supplement: Supplementary file 8 — Source data Fig. 5 [file 44319_2025_485_MOESM8_ESM.zip › Figure 5/5E/Fig_5E_GST_SAS_6_FL_BF_preP.tif]

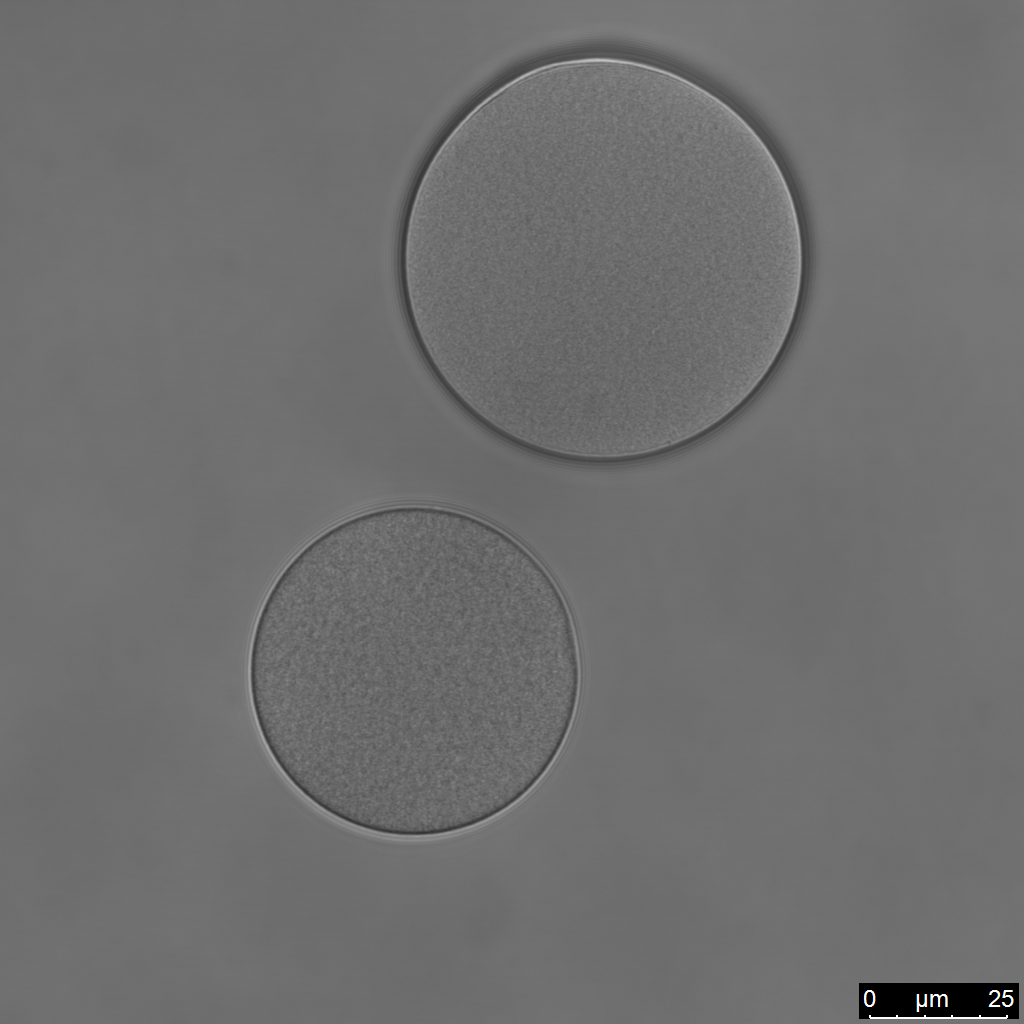

Supplement: Supplementary file 8 — Source data Fig. 5 [file 44319_2025_485_MOESM8_ESM.zip › Figure 5/5E/Fig_5E_GST_SAS_6deltaN_BF_preP.tif]

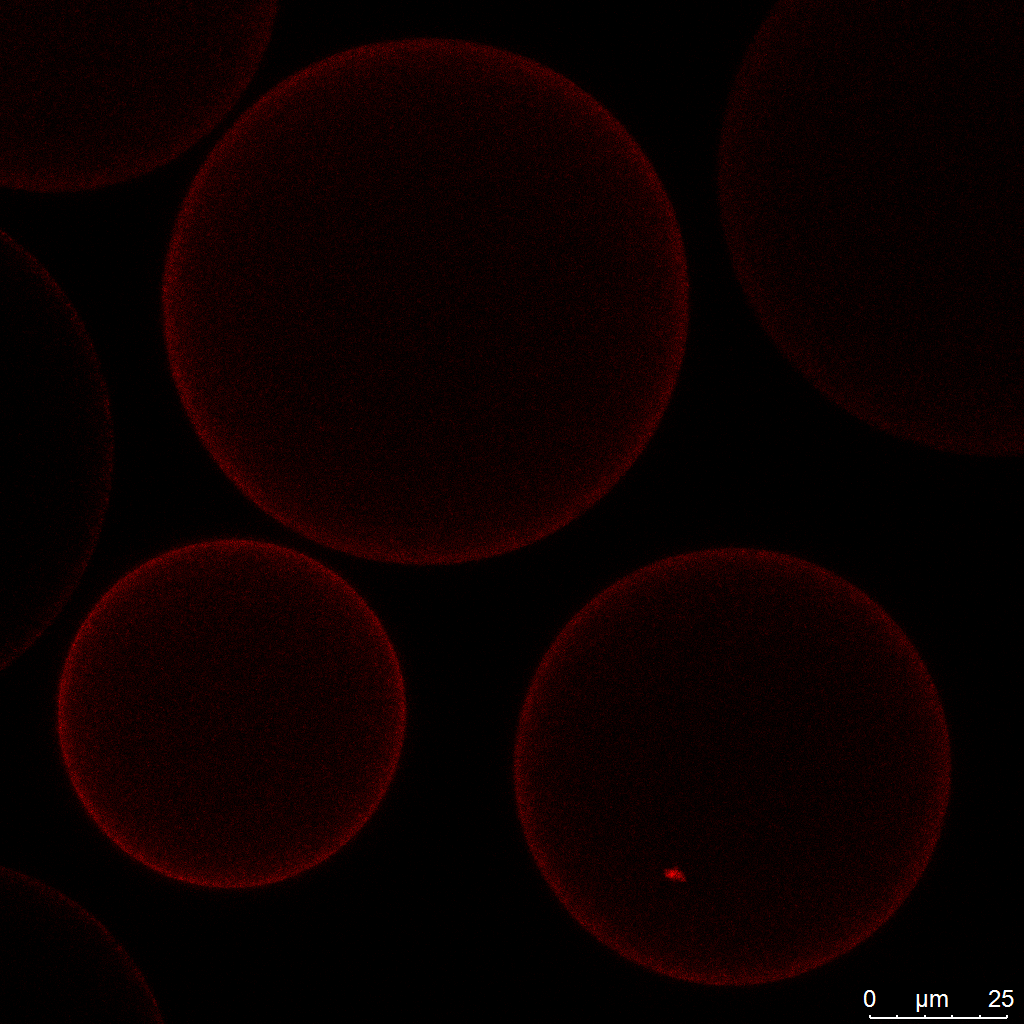

Supplement: Supplementary file 8 — Source data Fig. 5 [file 44319_2025_485_MOESM8_ESM.zip › Figure 5/5E/Fig_5E_GST_SAS_6_FL_mCherry.tif]

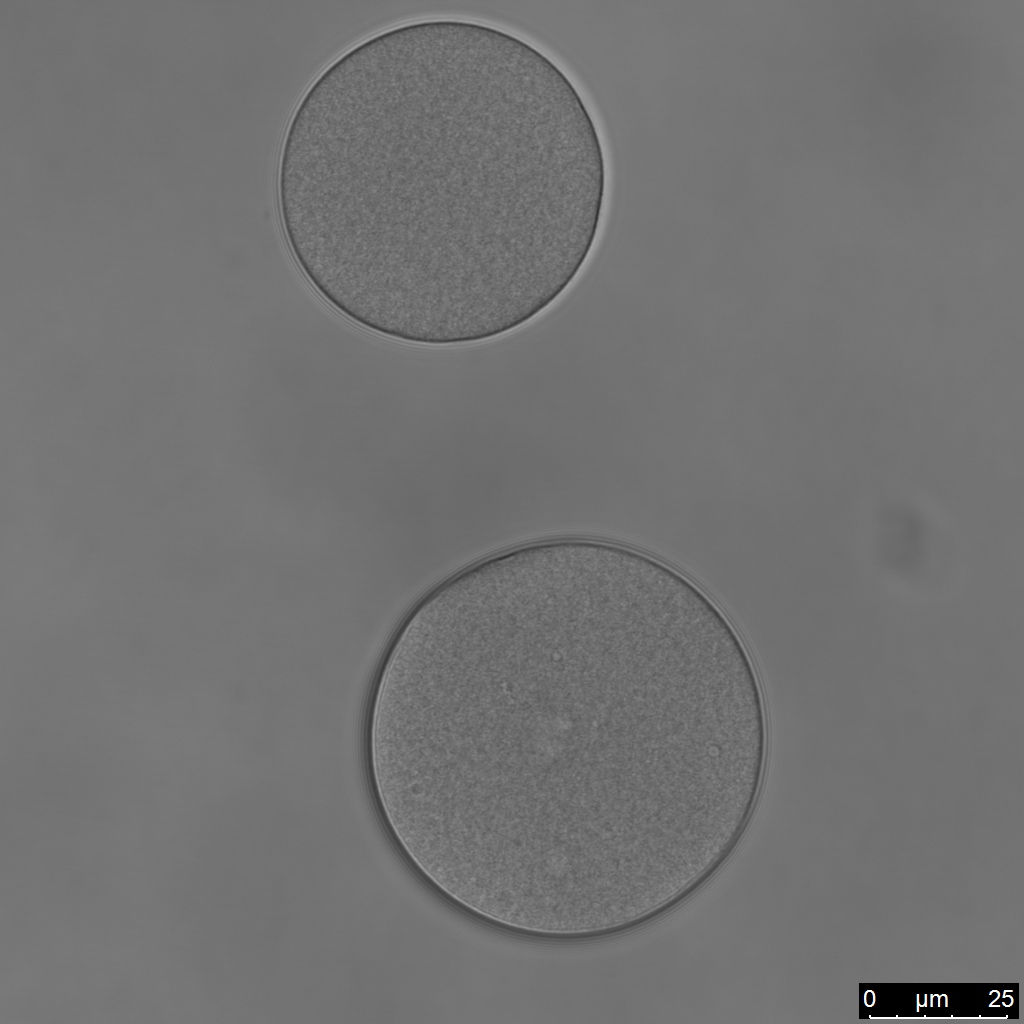

Supplement: Supplementary file 8 — Source data Fig. 5 [file 44319_2025_485_MOESM8_ESM.zip › Figure 5/5E/Fig_5E_GST_SAS_6deltaN_BF.tif]

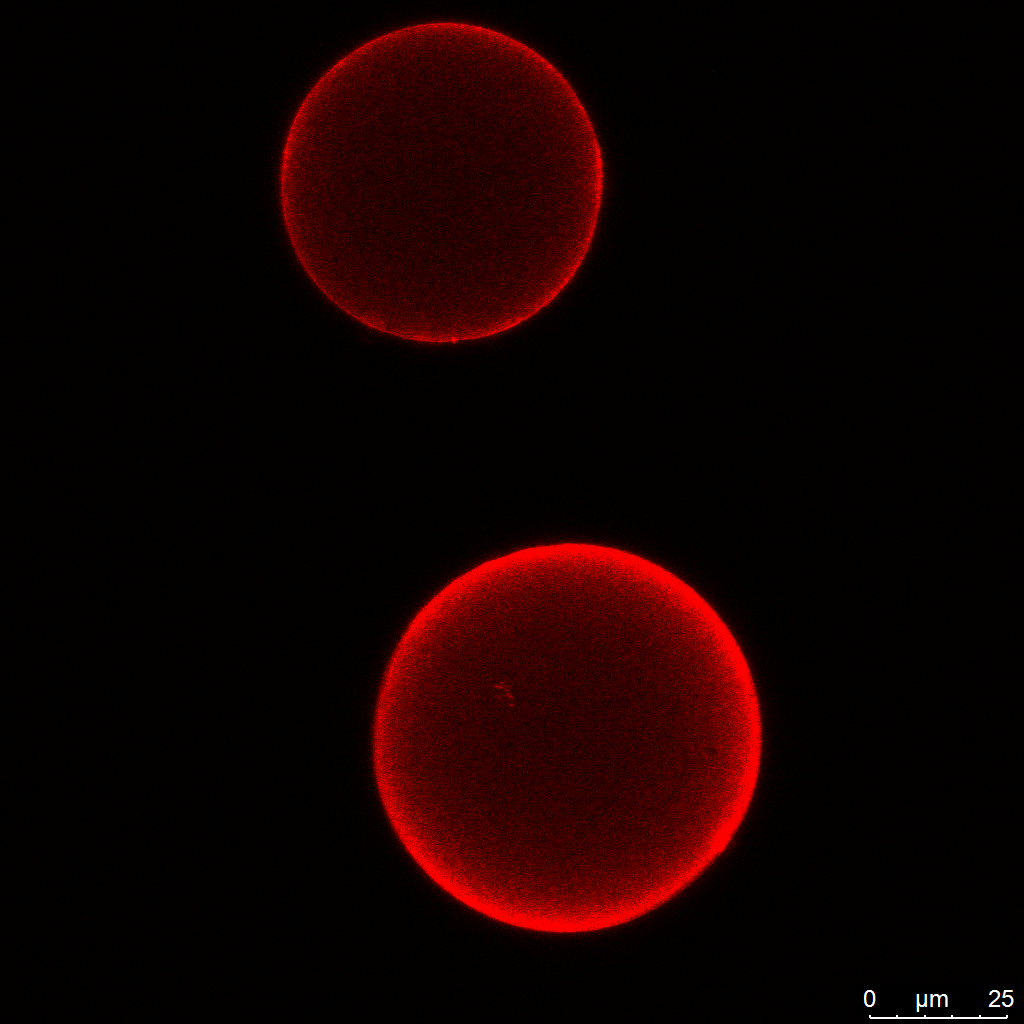

Supplement: Supplementary file 8 — Source data Fig. 5 [file 44319_2025_485_MOESM8_ESM.zip › Figure 5/5E/Fig_5E_GST_SAS_6deltaN_mCherry.tif]

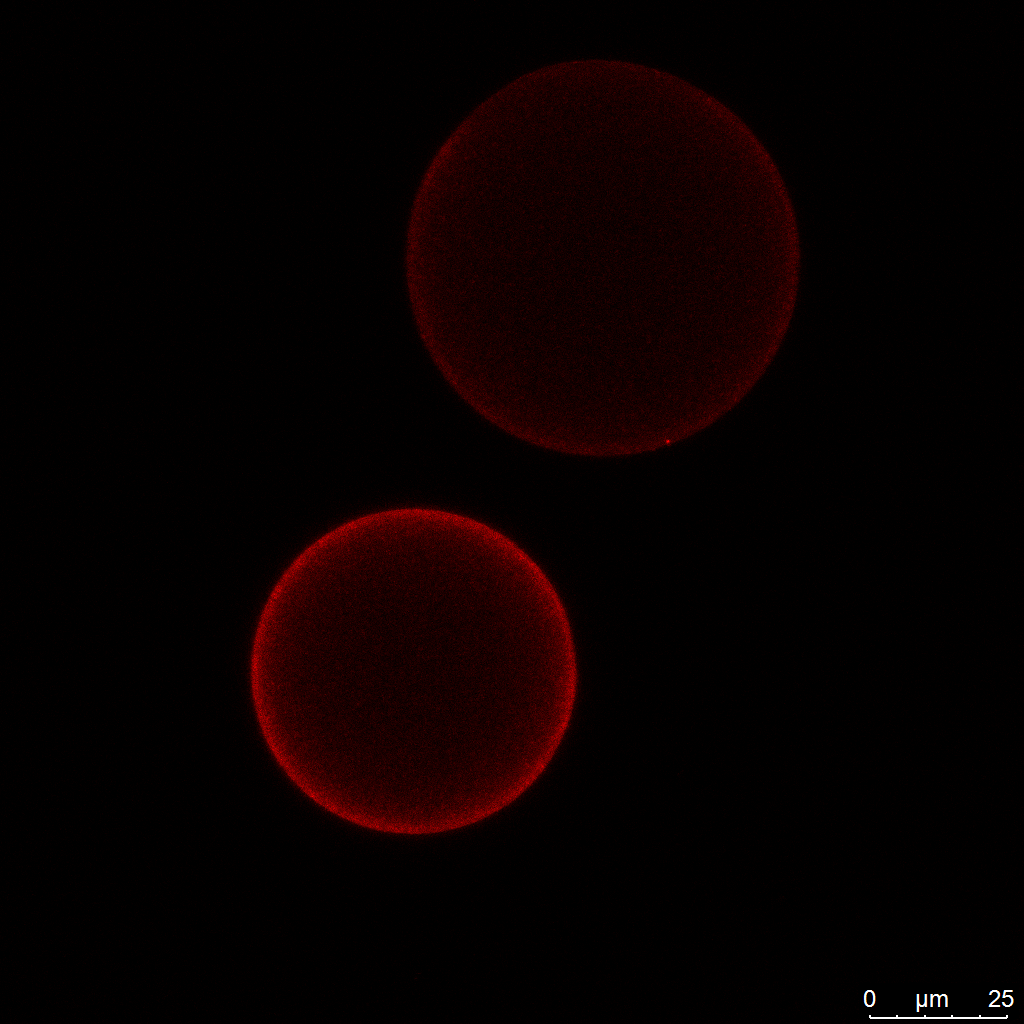

Supplement: Supplementary file 8 — Source data Fig. 5 [file 44319_2025_485_MOESM8_ESM.zip › Figure 5/5E/Fig_5E_GST_SAS_6deltaN_mCherry_preP.tif]

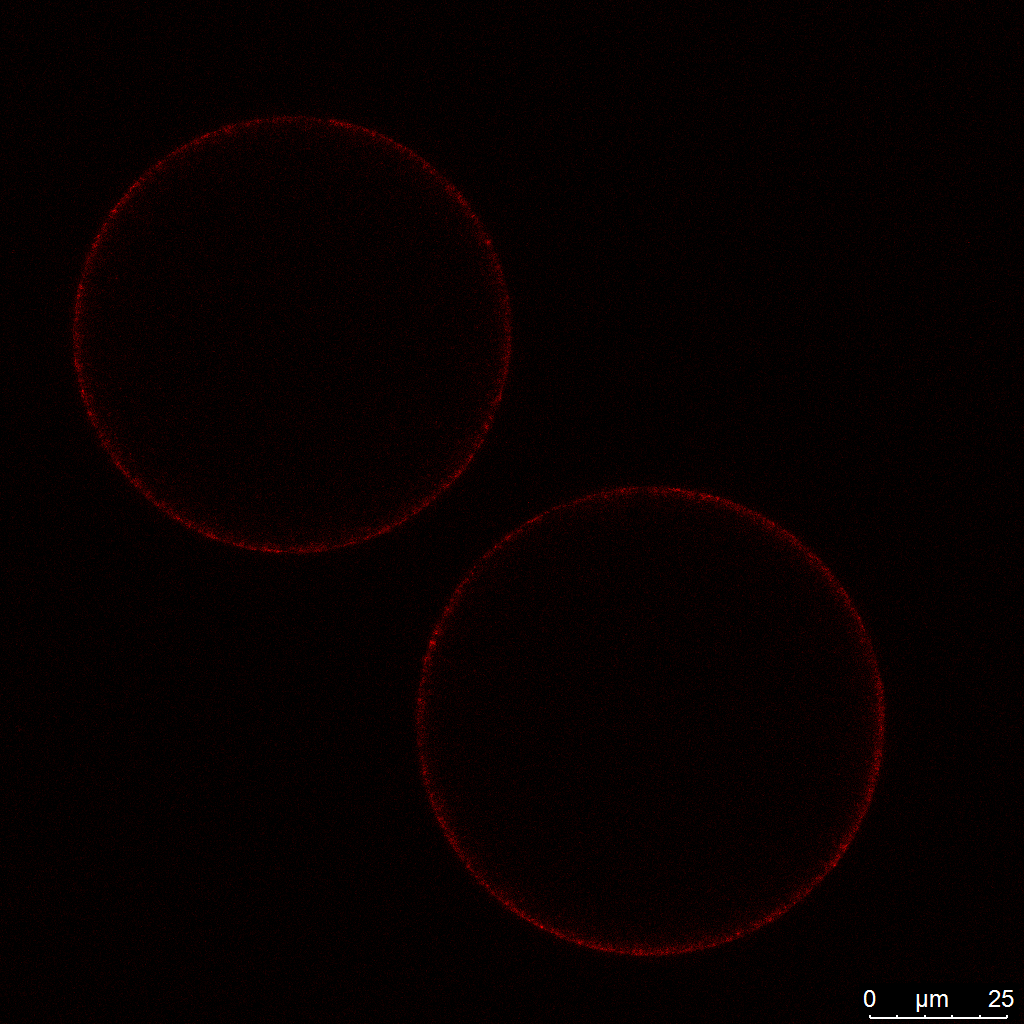

Supplement: Supplementary file 8 — Source data Fig. 5 [file 44319_2025_485_MOESM8_ESM.zip › Figure 5/5B/Fig. 5B_delN_mCherry.tif]

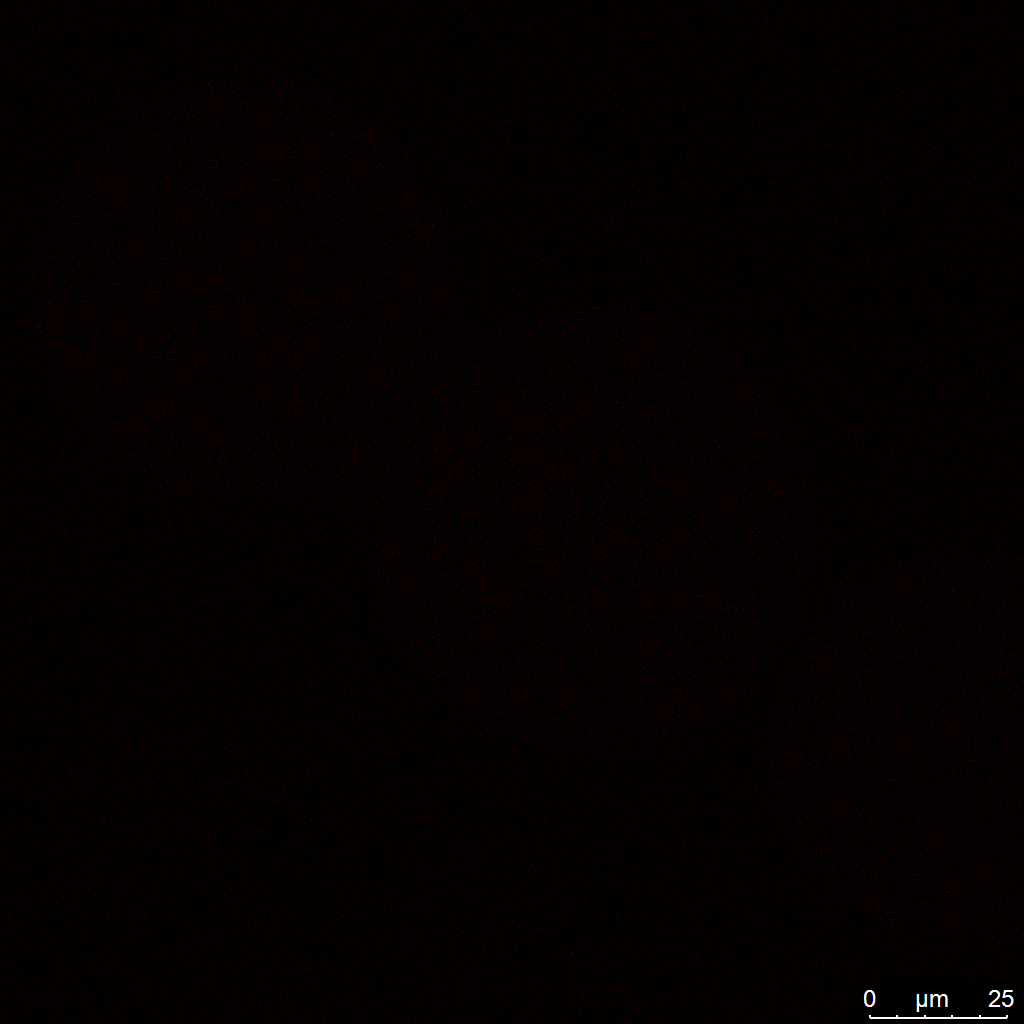

Supplement: Supplementary file 8 — Source data Fig. 5 [file 44319_2025_485_MOESM8_ESM.zip › Figure 5/5B/Fig. 5B_GST_mCherry.tif]

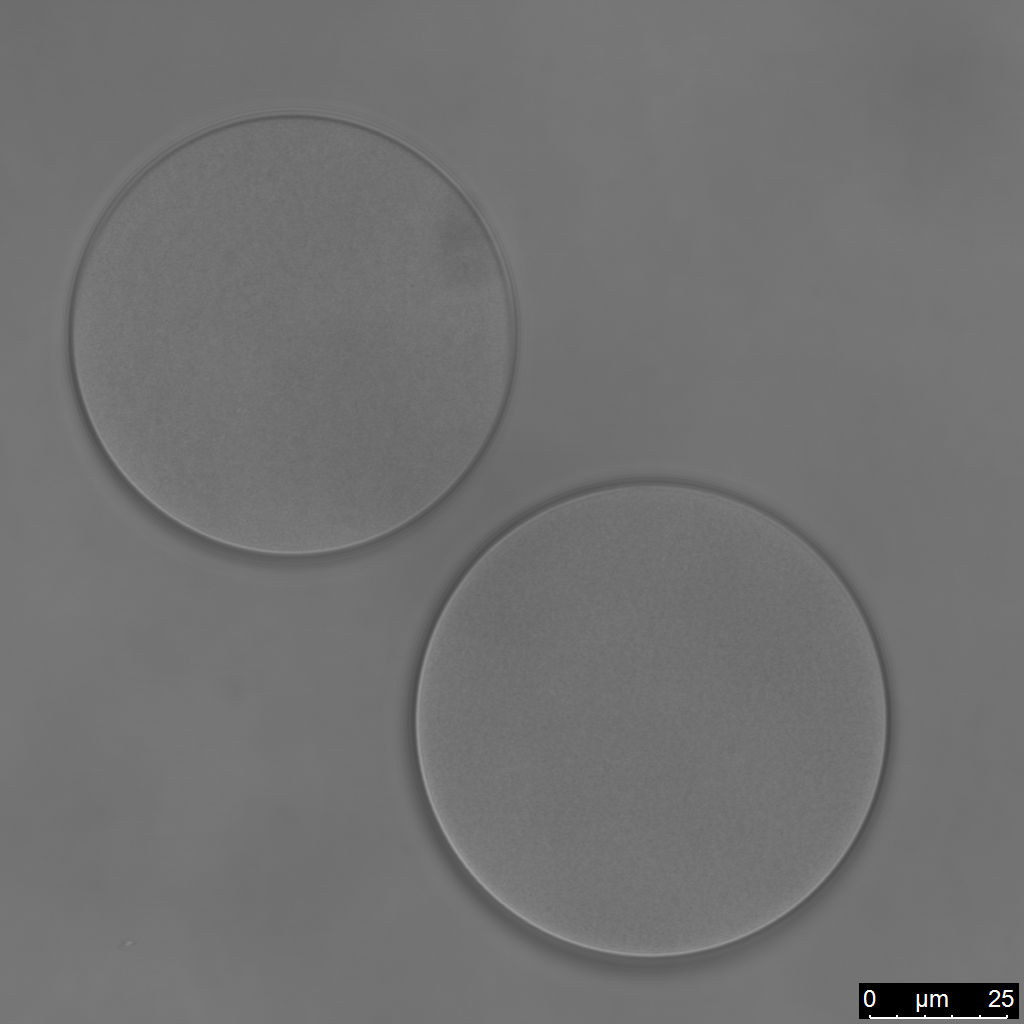

Supplement: Supplementary file 8 — Source data Fig. 5 [file 44319_2025_485_MOESM8_ESM.zip › Figure 5/5B/Fig. 5B_delN_BF.tif]

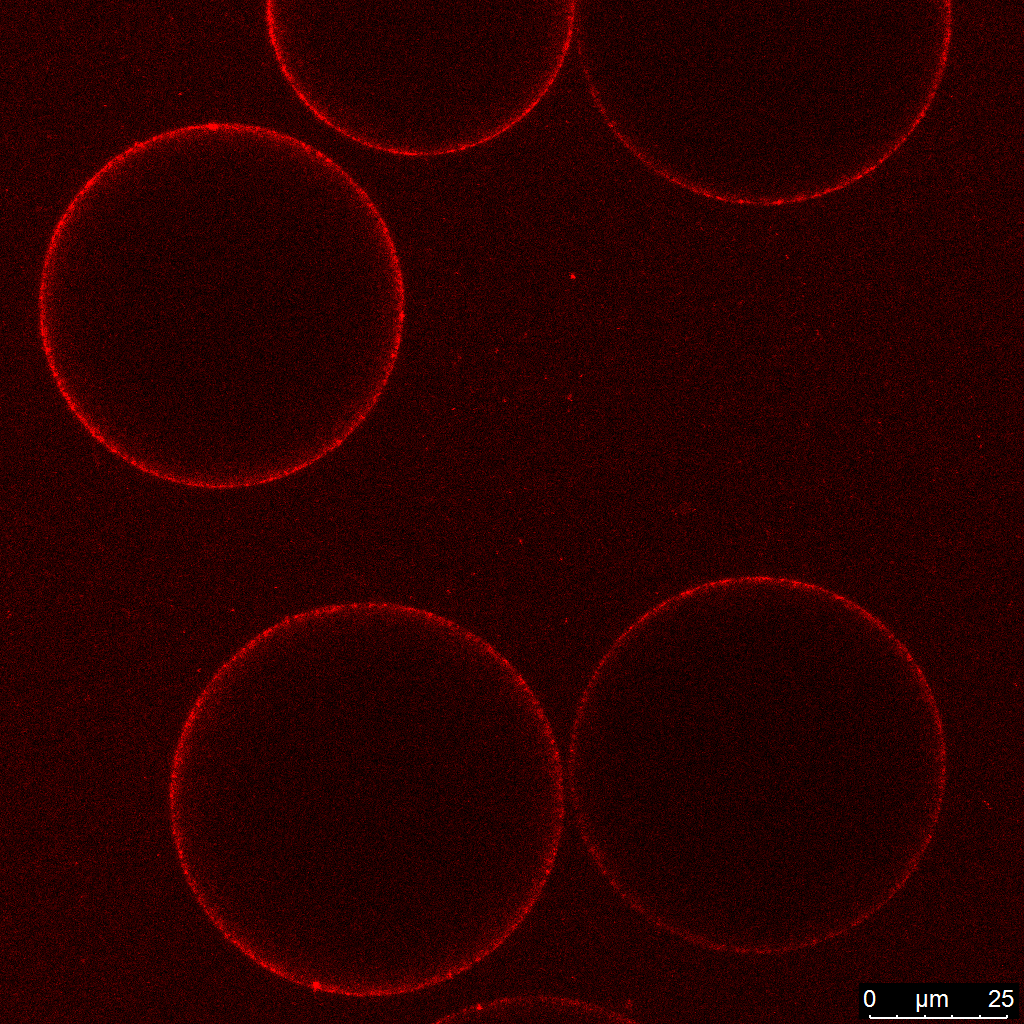

Supplement: Supplementary file 8 — Source data Fig. 5 [file 44319_2025_485_MOESM8_ESM.zip › Figure 5/5B/Fig. 5B_FL_mCherry.tif]

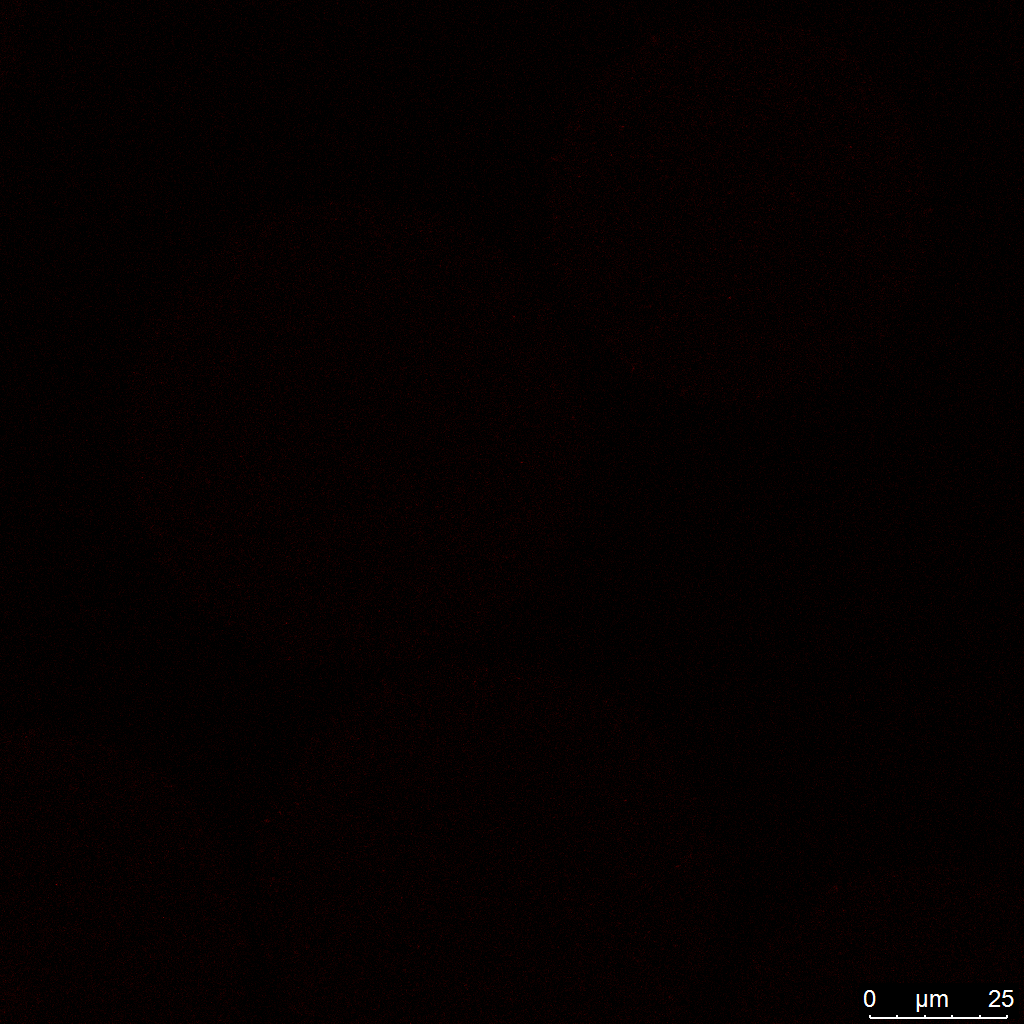

Supplement: Supplementary file 8 — Source data Fig. 5 [file 44319_2025_485_MOESM8_ESM.zip › Figure 5/5B/Fig. 5B_delM_mCherry.tif]

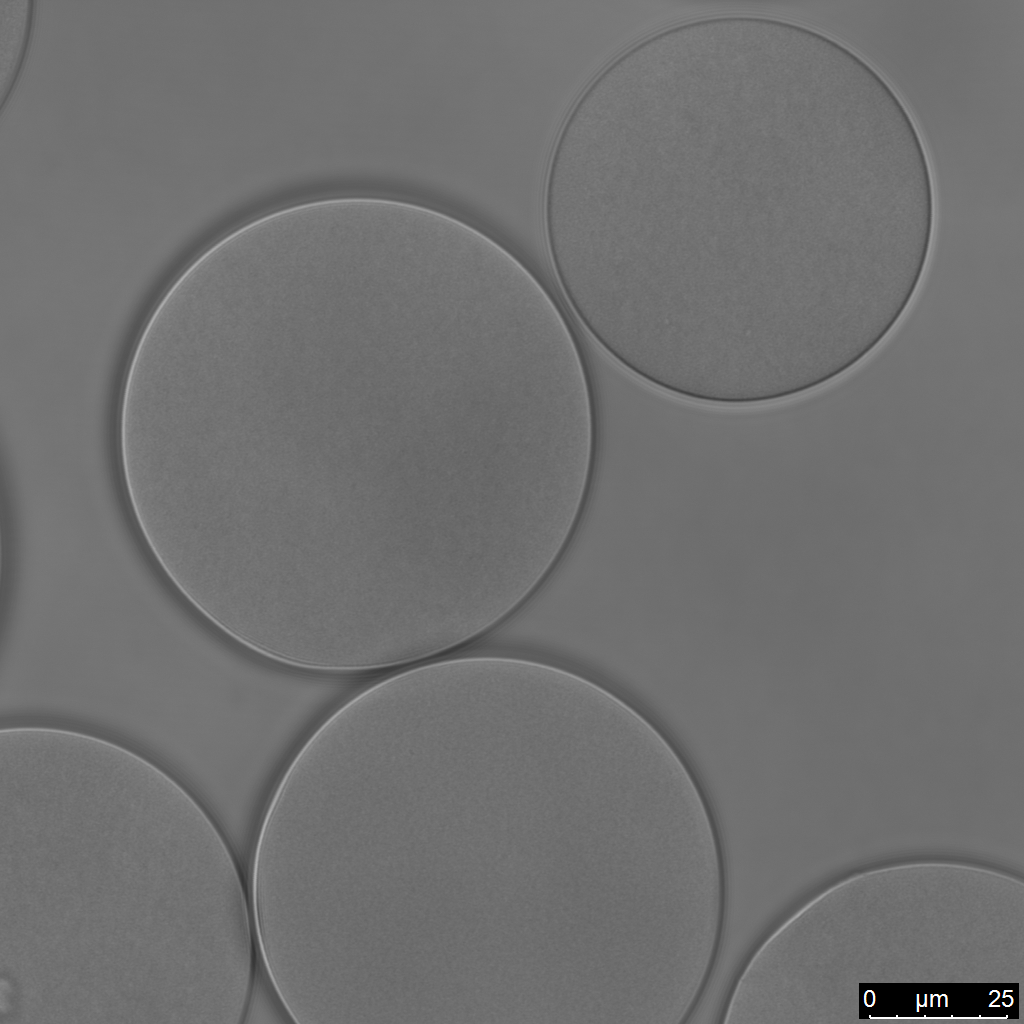

Supplement: Supplementary file 8 — Source data Fig. 5 [file 44319_2025_485_MOESM8_ESM.zip › Figure 5/5B/Fig. 5B_delM_BF.tif]

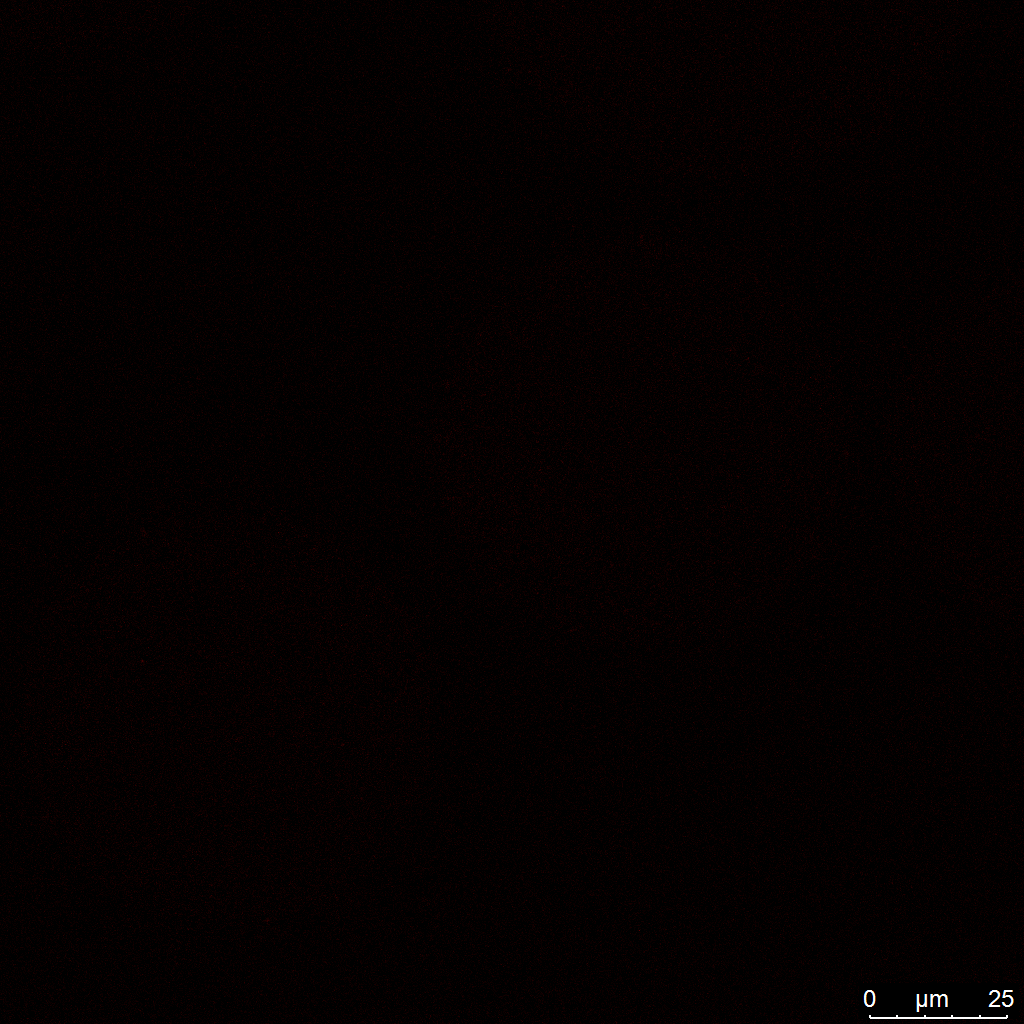

Supplement: Supplementary file 8 — Source data Fig. 5 [file 44319_2025_485_MOESM8_ESM.zip › Figure 5/5B/Fig. 5B_delC_mCherry.tif]

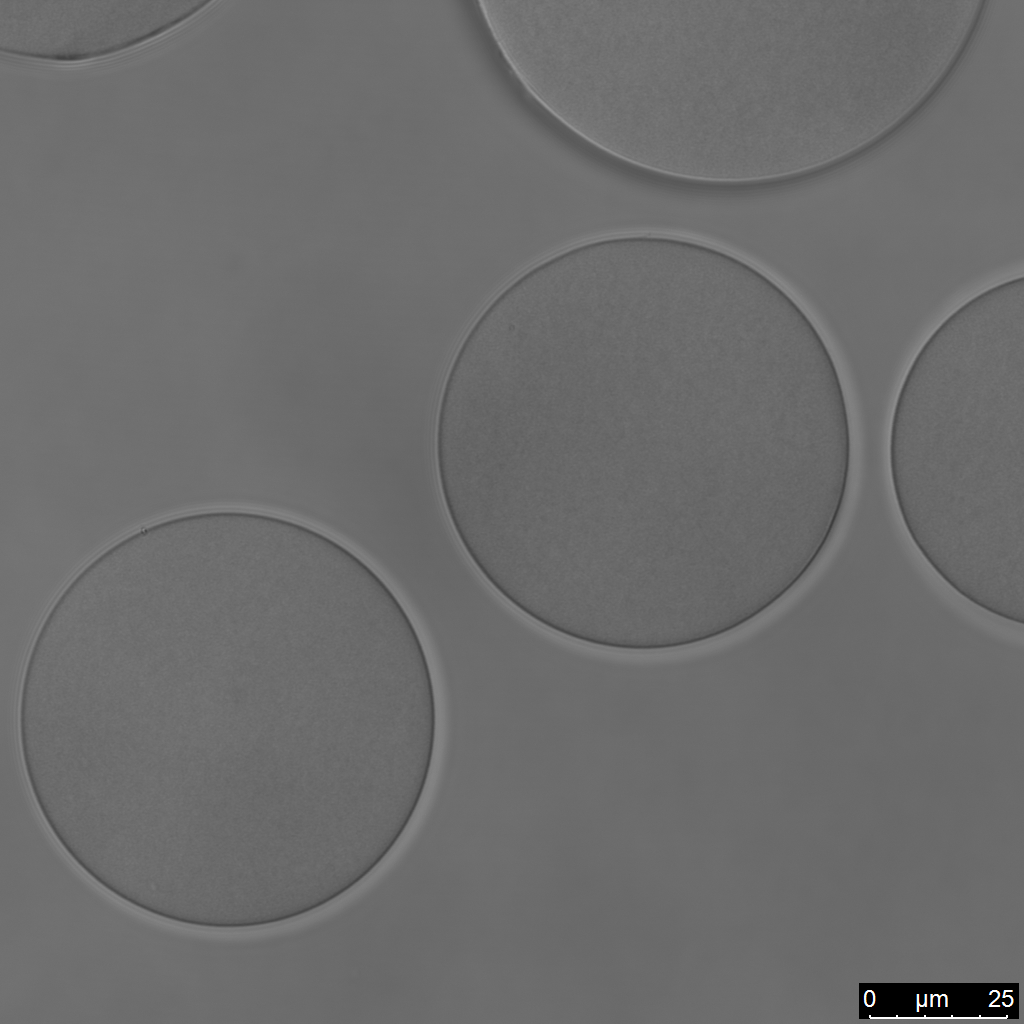

Supplement: Supplementary file 8 — Source data Fig. 5 [file 44319_2025_485_MOESM8_ESM.zip › Figure 5/5B/Fig. 5B_delC_BF.tif]

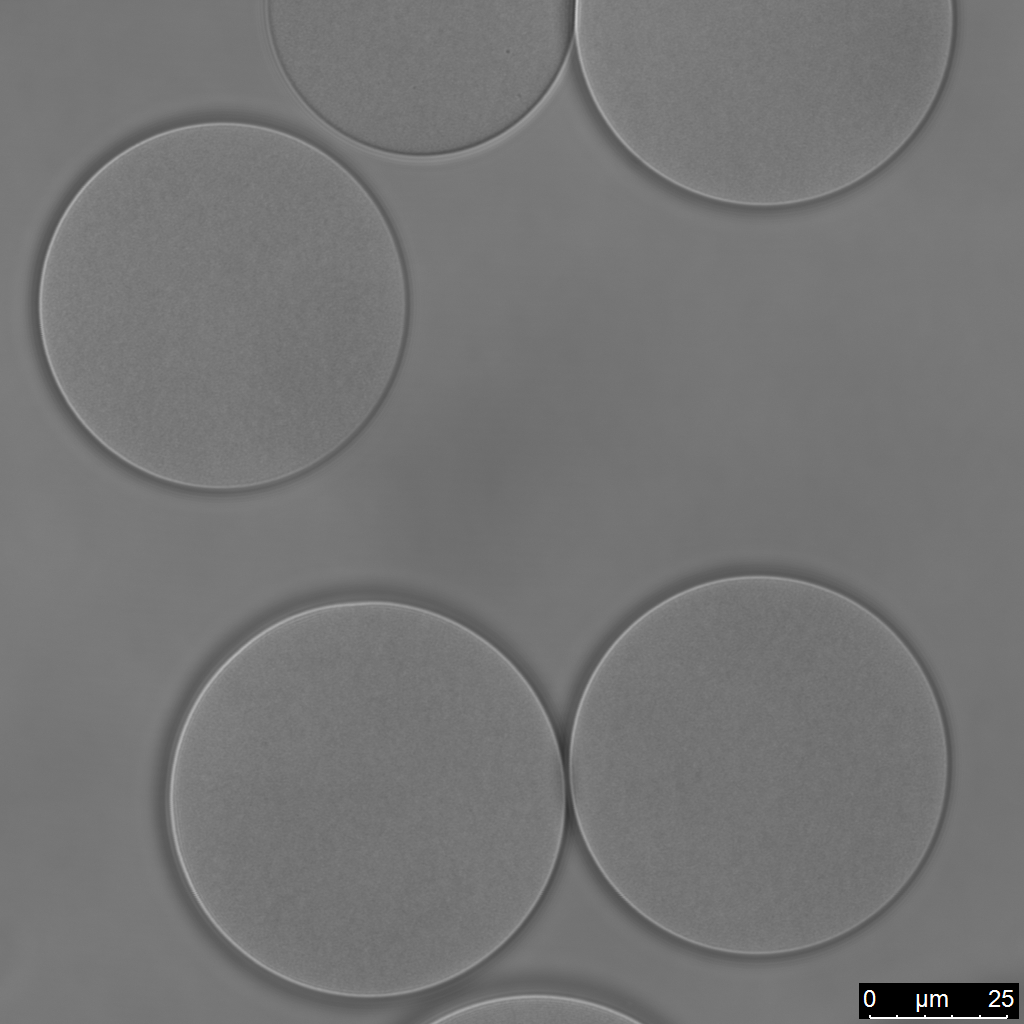

Supplement: Supplementary file 8 — Source data Fig. 5 [file 44319_2025_485_MOESM8_ESM.zip › Figure 5/5B/Fig. 5B_FL_BF.tif]

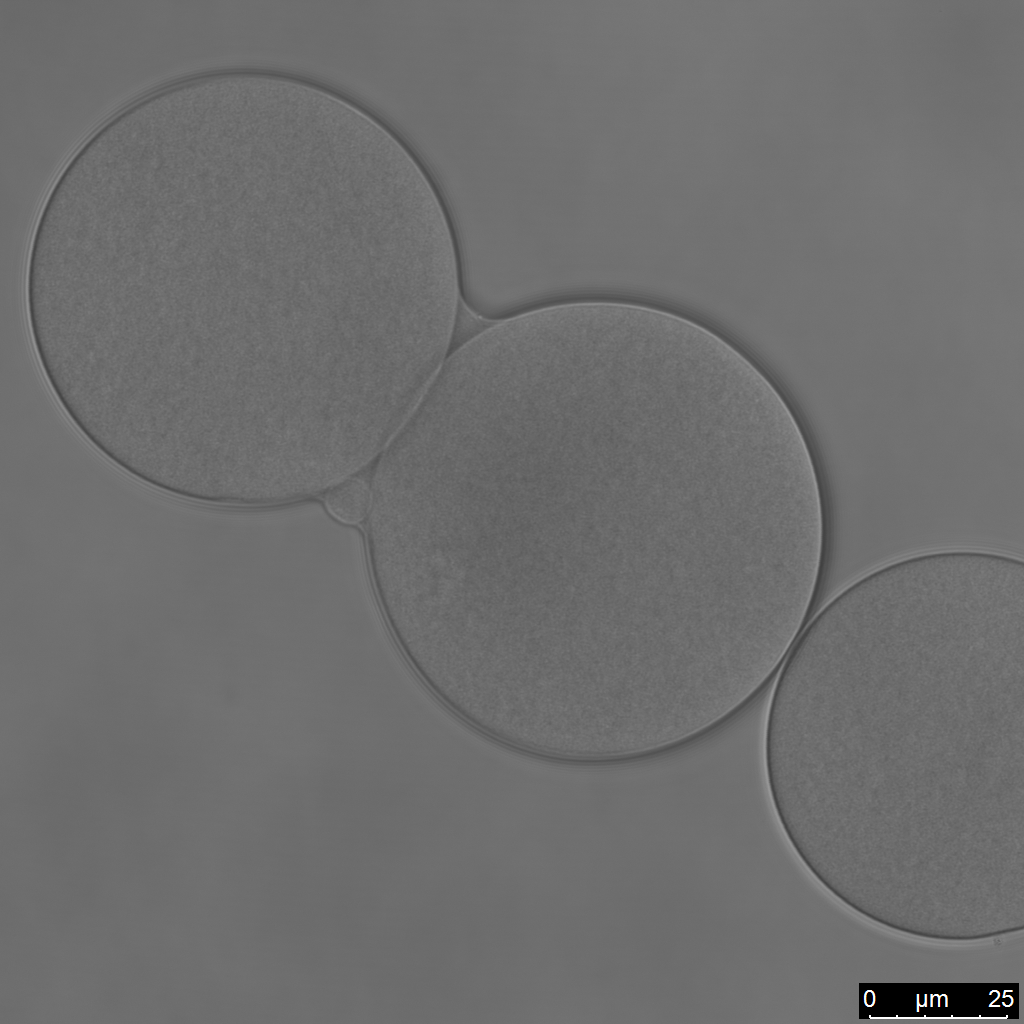

Supplement: Supplementary file 8 — Source data Fig. 5 [file 44319_2025_485_MOESM8_ESM.zip › Figure 5/5B/Fig. 5B_GST_BF.tif]

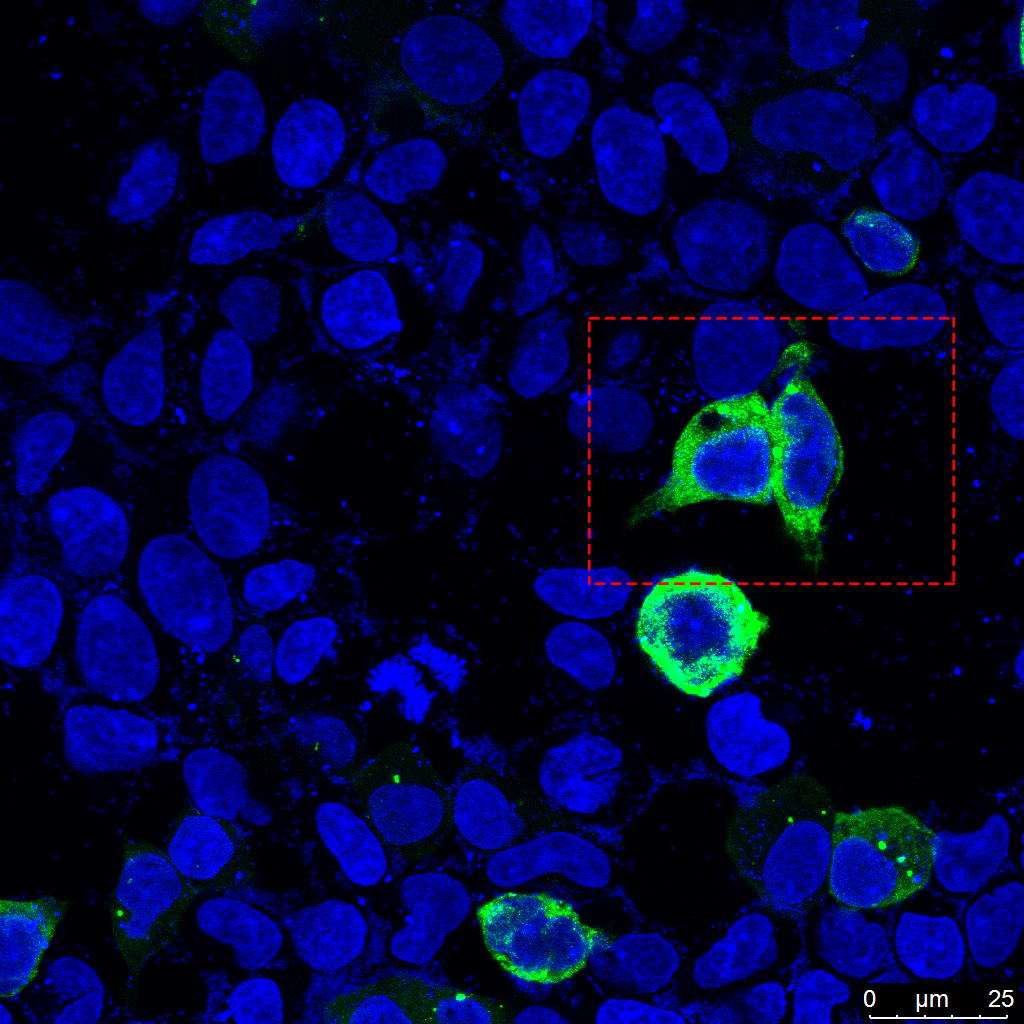

Supplement: Supplementary file 9 — Source data Fig. 6 [file 44319_2025_485_MOESM9_ESM.zip › Figure 6/6A/Fig.6A_SASS_6_deltaC.tif]

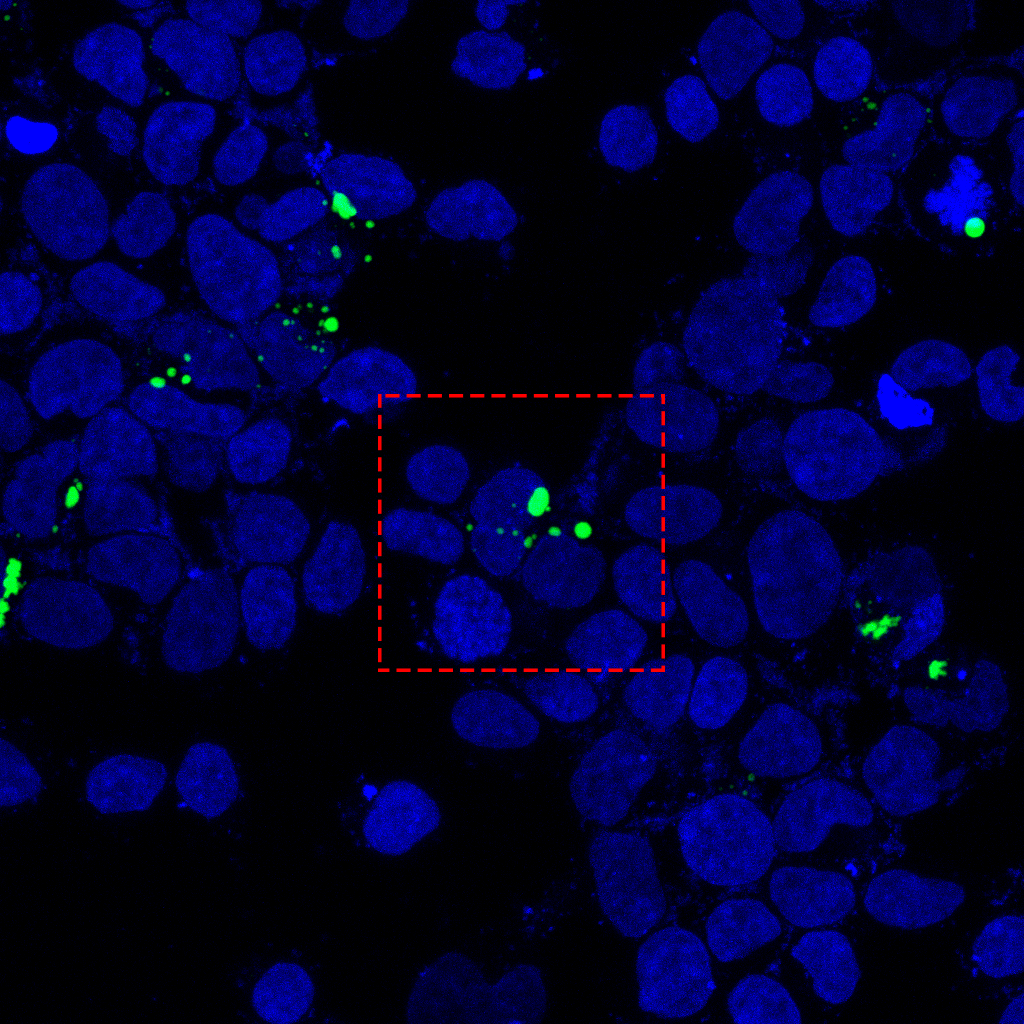

Supplement: Supplementary file 9 — Source data Fig. 6 [file 44319_2025_485_MOESM9_ESM.zip › Figure 6/6A/Fig.6A_SASS_6_FL.tif]

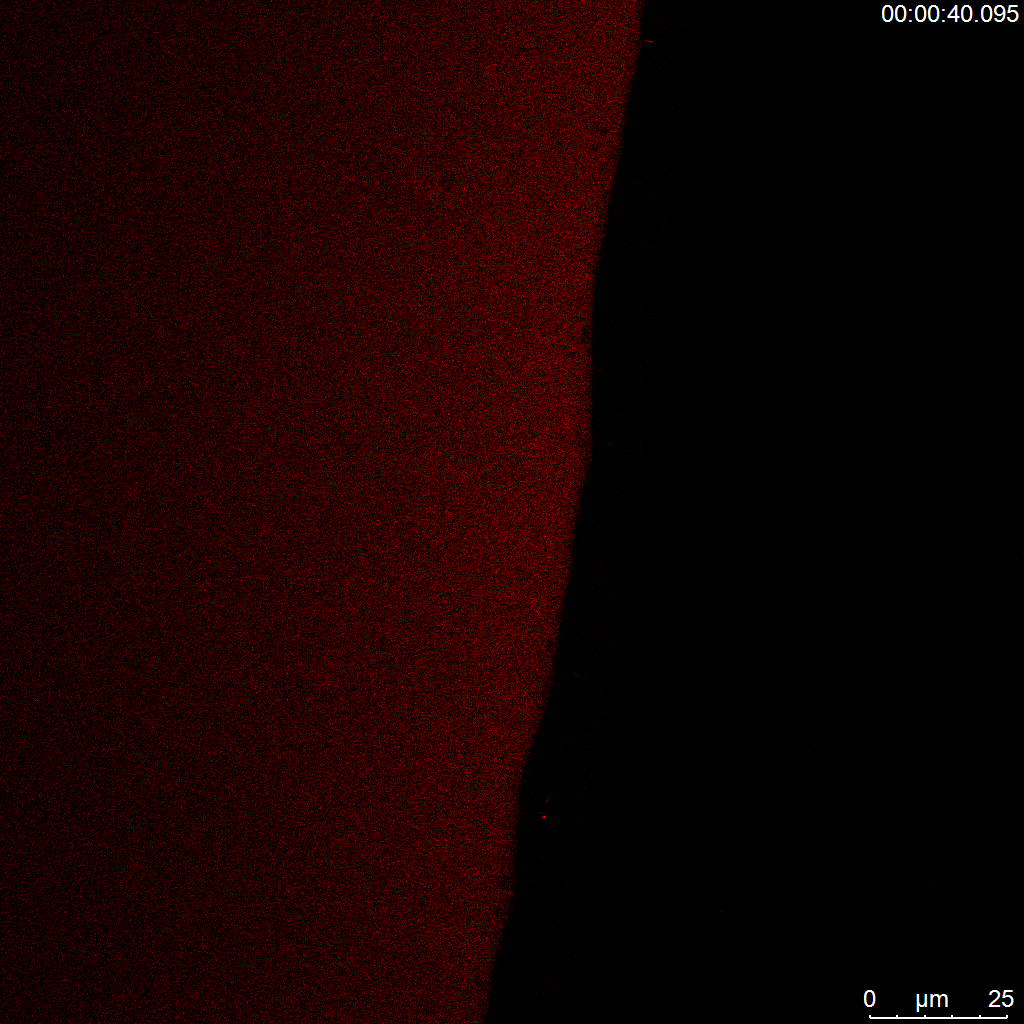

Supplement: Supplementary file 9 — Source data Fig. 6 [file 44319_2025_485_MOESM9_ESM.zip › Figure 6/6H/Fig. 6H_40S_preP.tif]

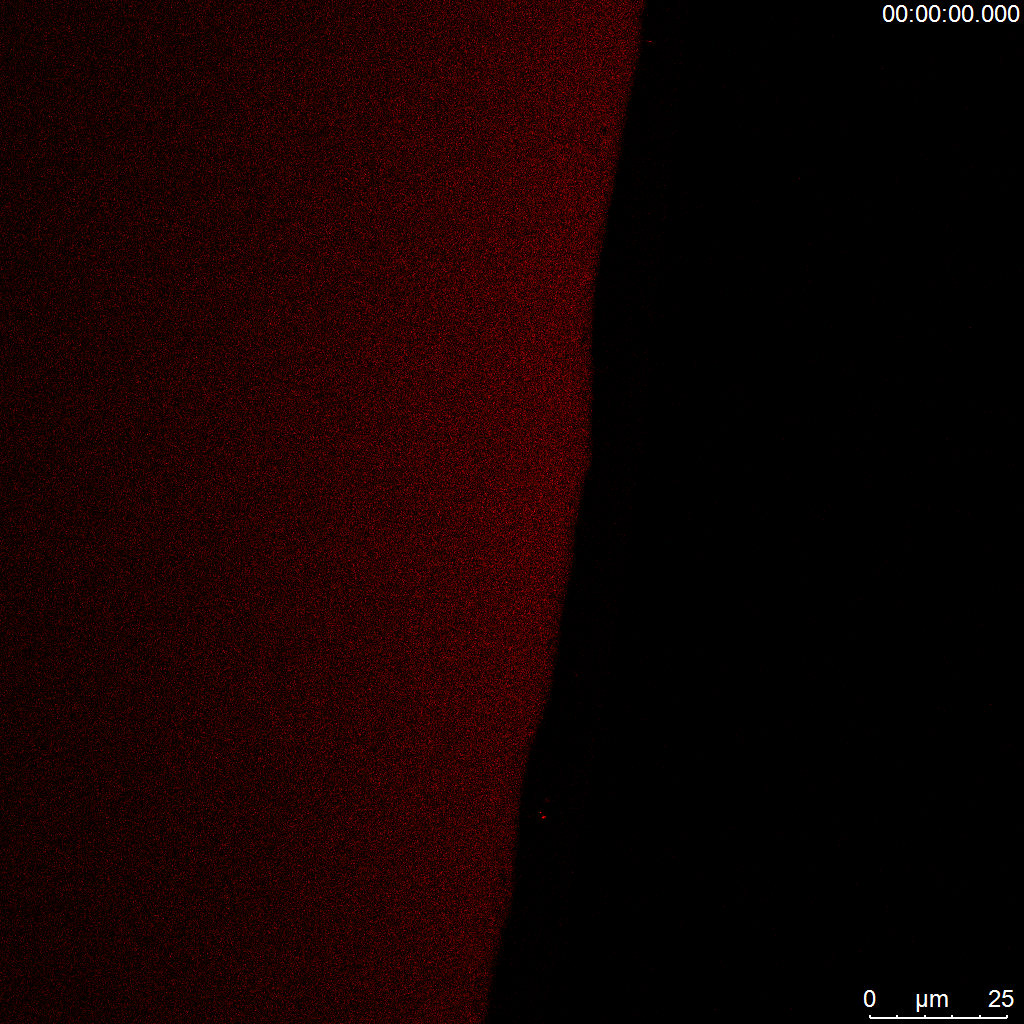

Supplement: Supplementary file 9 — Source data Fig. 6 [file 44319_2025_485_MOESM9_ESM.zip › Figure 6/6H/Fig. 6H_0S_preP.tif]

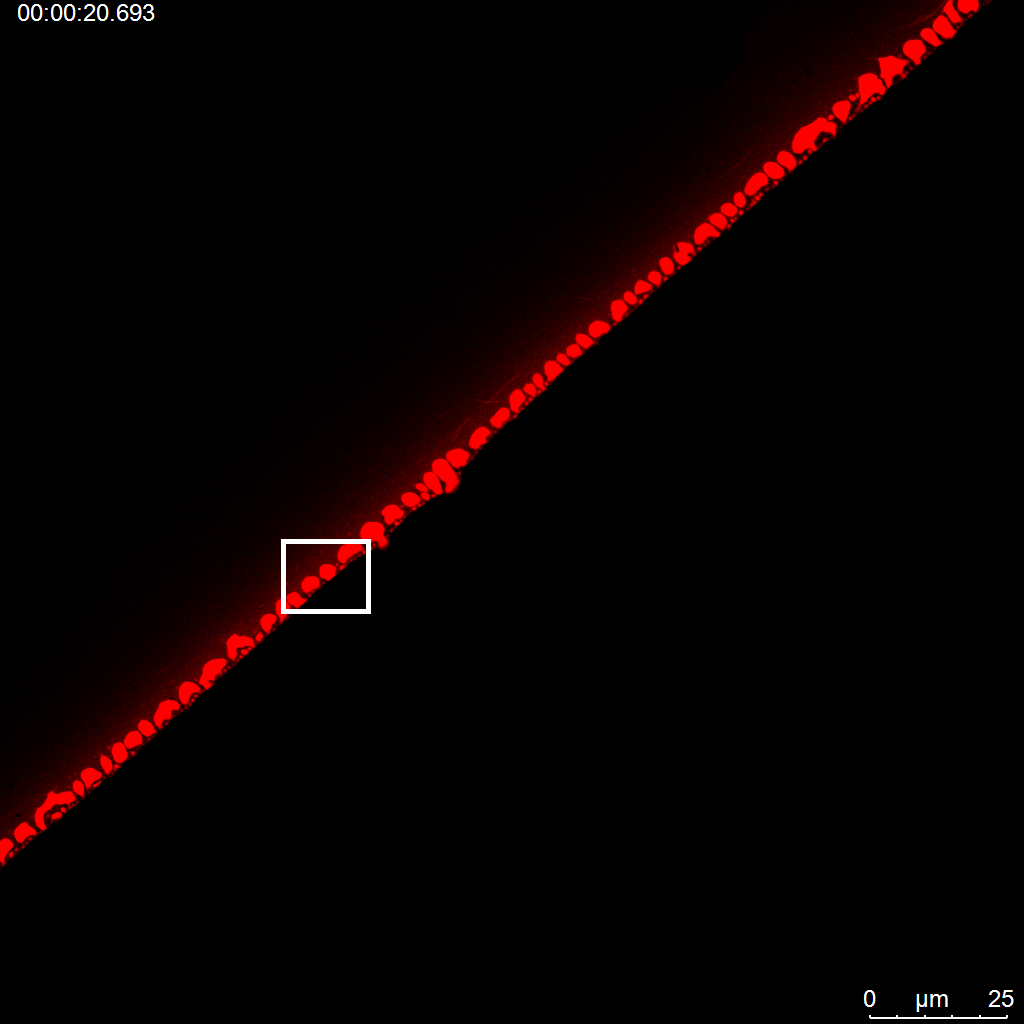

Supplement: Supplementary file 9 — Source data Fig. 6 [file 44319_2025_485_MOESM9_ESM.zip › Figure 6/6H/Fig. 6H_20S.tif]

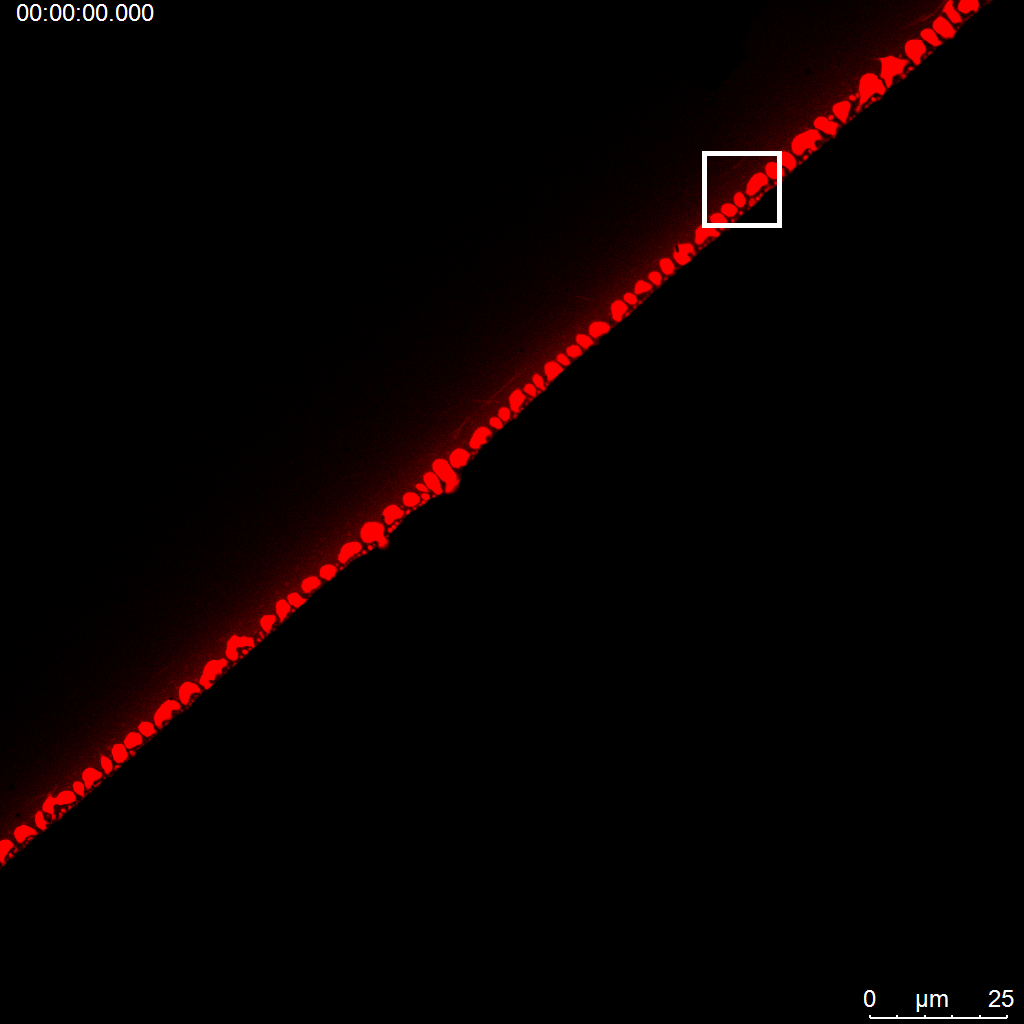

Supplement: Supplementary file 9 — Source data Fig. 6 [file 44319_2025_485_MOESM9_ESM.zip › Figure 6/6H/Fig. 6H_0S.tif]

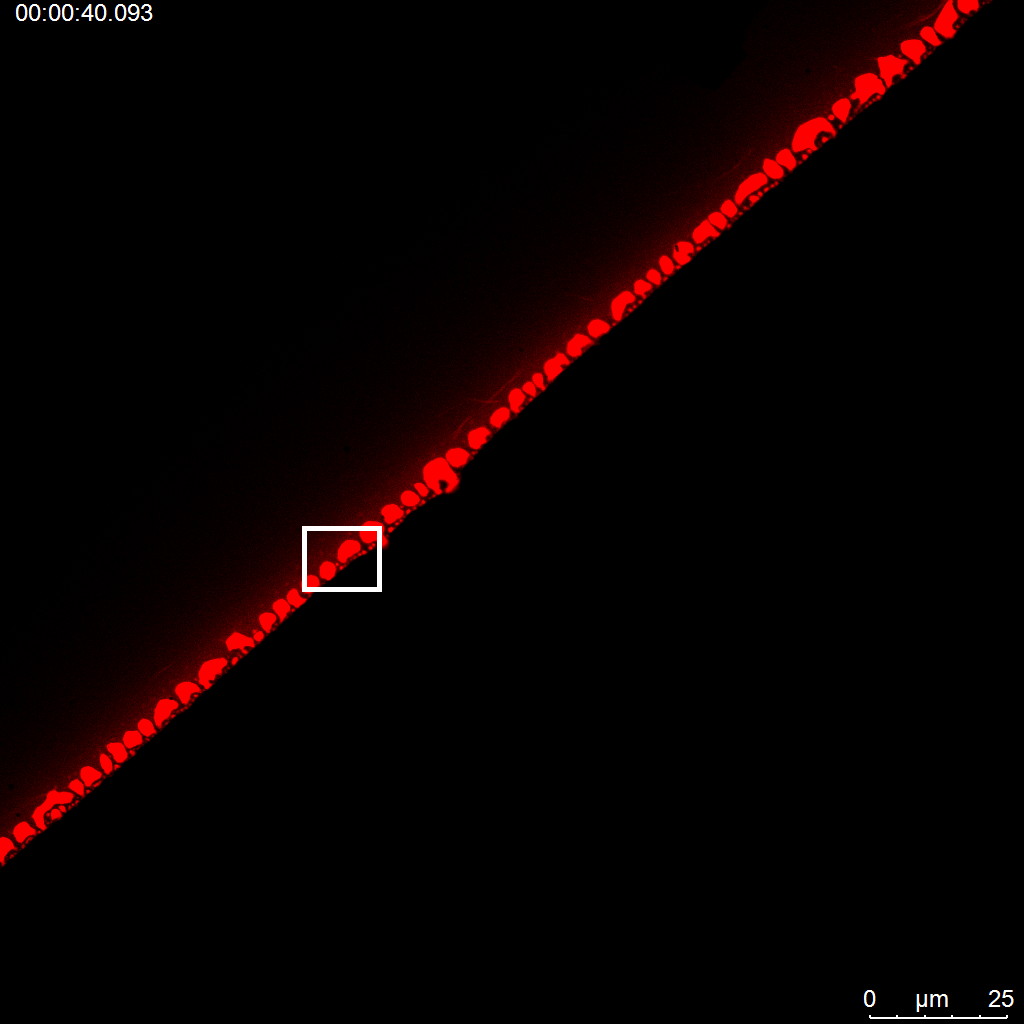

Supplement: Supplementary file 9 — Source data Fig. 6 [file 44319_2025_485_MOESM9_ESM.zip › Figure 6/6H/Fig. 6H_40S.tif]

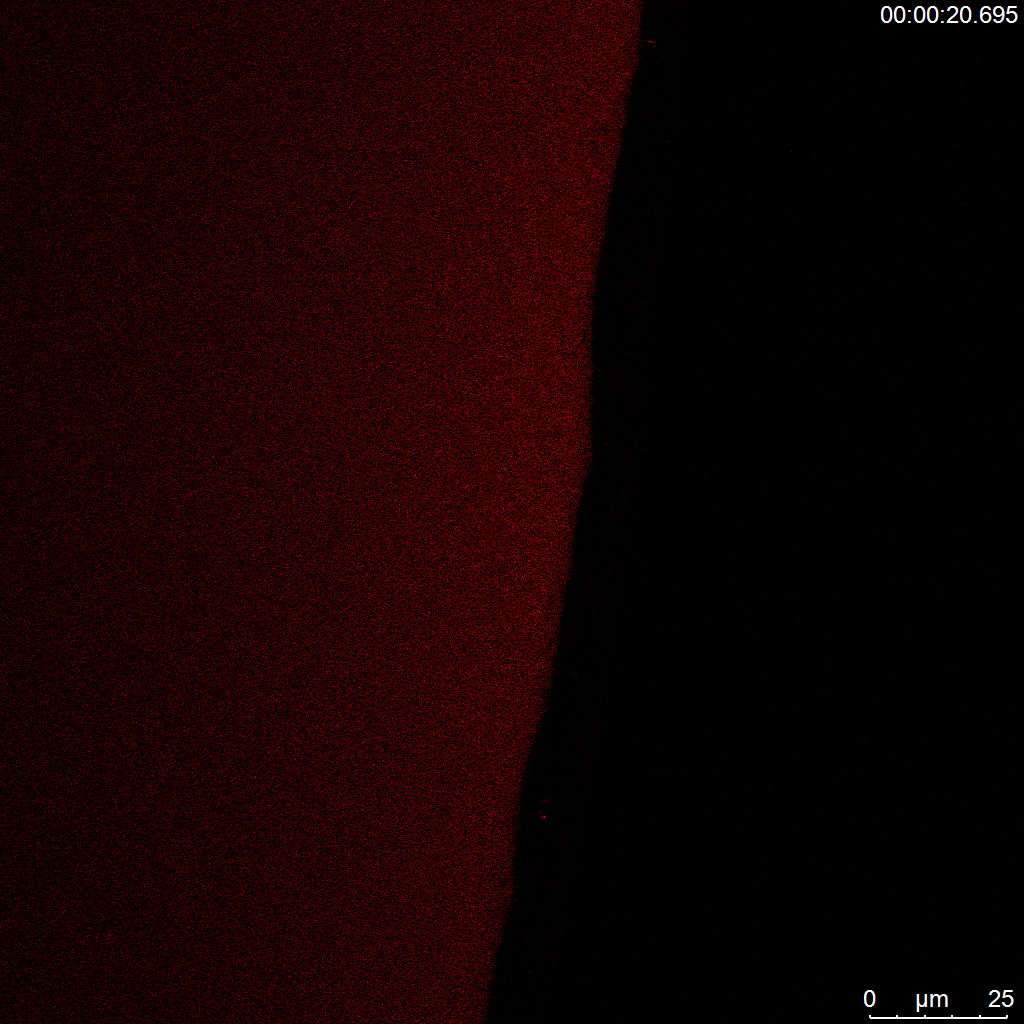

Supplement: Supplementary file 9 — Source data Fig. 6 [file 44319_2025_485_MOESM9_ESM.zip › Figure 6/6H/Fig. 6H_20S_preP.tif]

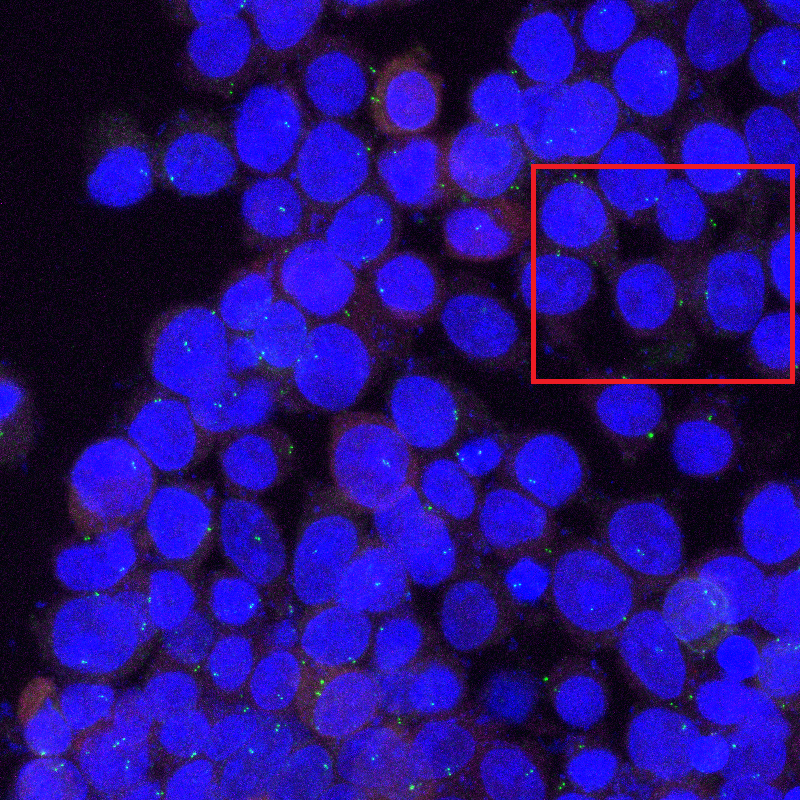

Supplement: Supplementary file 9 — Source data Fig. 6 [file 44319_2025_485_MOESM9_ESM.zip › Figure 6/6I/Fig.6I_1,6_Hex_Centrin.tif]

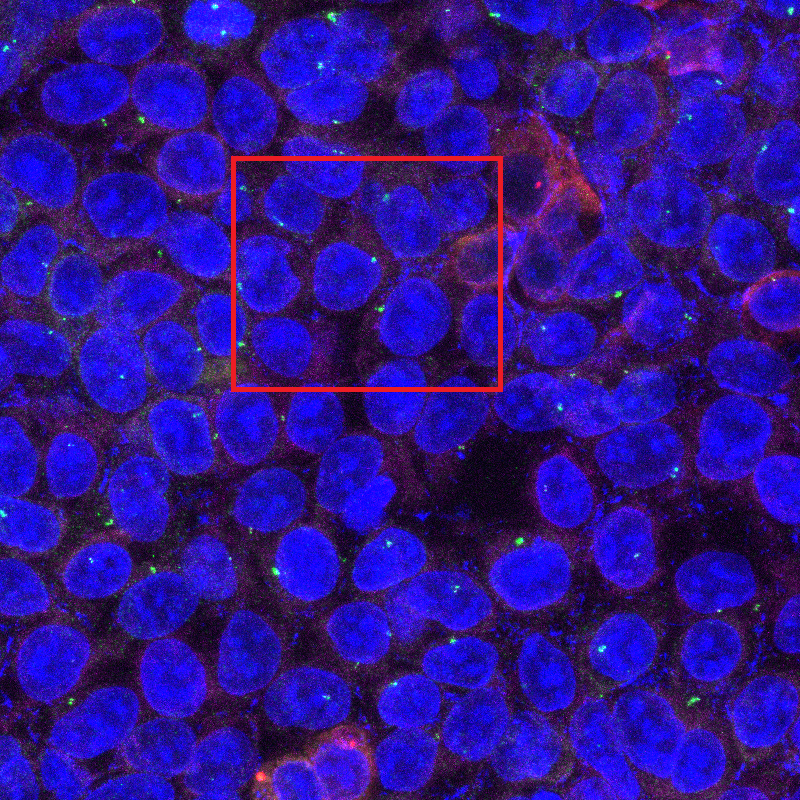

Supplement: Supplementary file 9 — Source data Fig. 6 [file 44319_2025_485_MOESM9_ESM.zip › Figure 6/6I/Fig.6I_control_Centrin.tif]

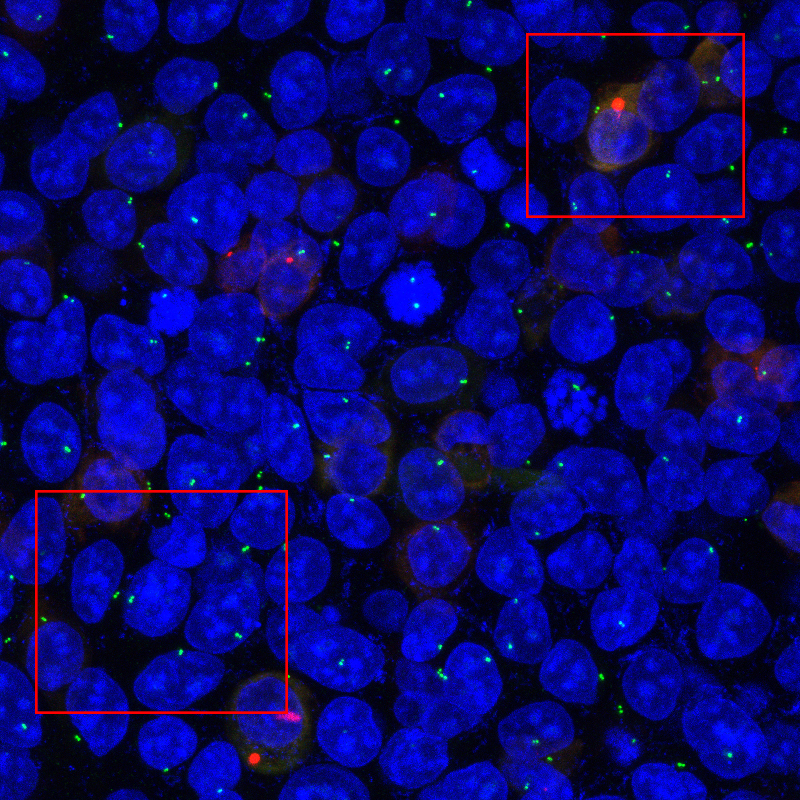

Supplement: Supplementary file 9 — Source data Fig. 6 [file 44319_2025_485_MOESM9_ESM.zip › Figure 6/6I/Fig.6I_control_SAS_6_mCherry_CEP192.tif]

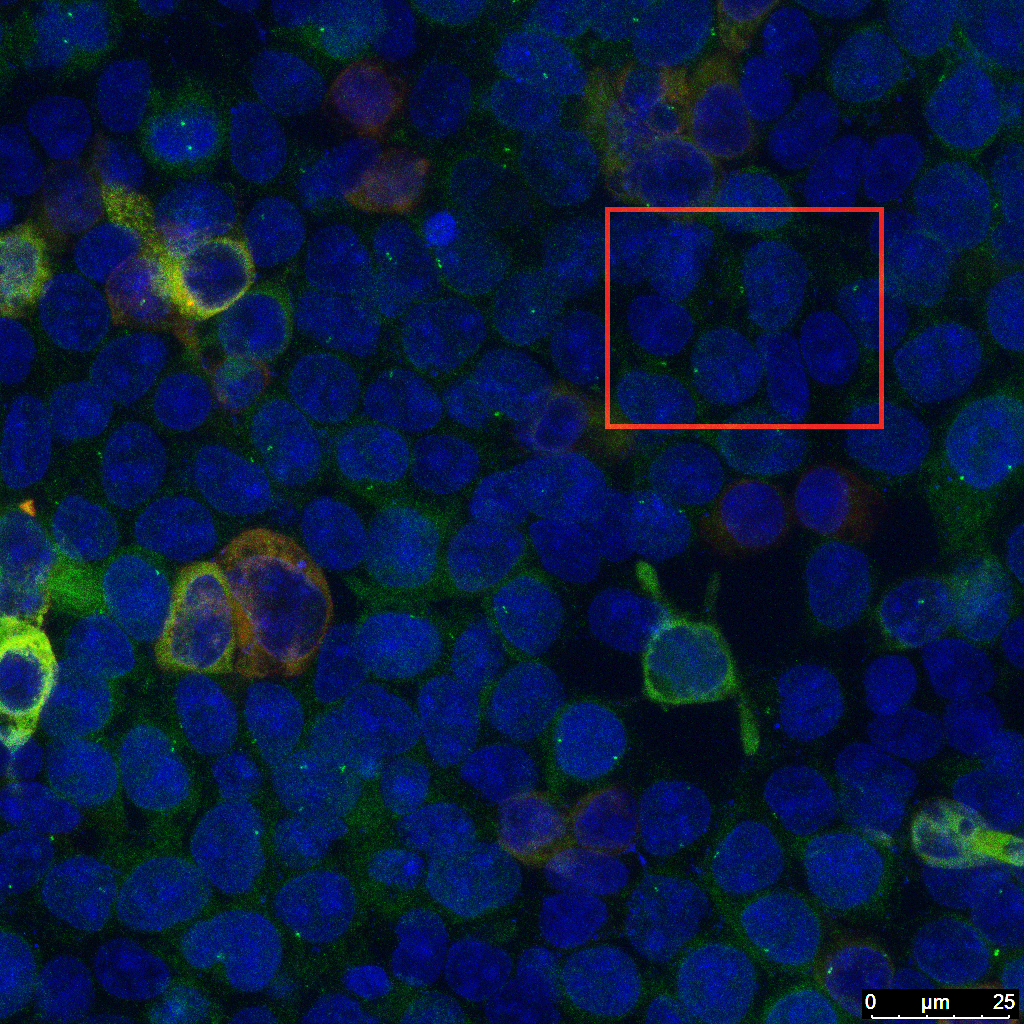

Supplement: Supplementary file 9 — Source data Fig. 6 [file 44319_2025_485_MOESM9_ESM.zip › Figure 6/6I/Fig.6I_control_SASS_6.tif]

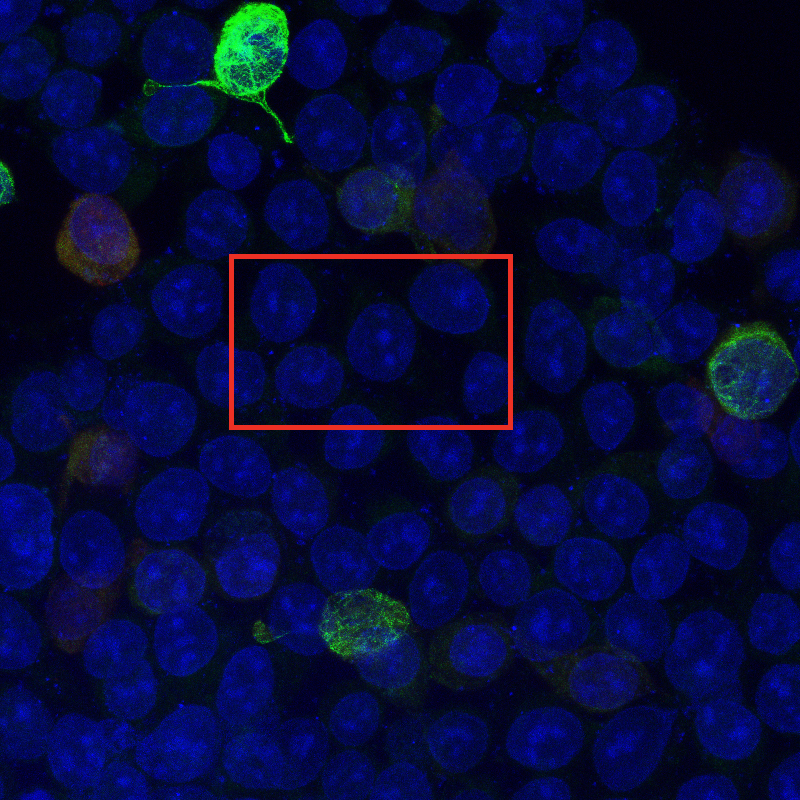

Supplement: Supplementary file 9 — Source data Fig. 6 [file 44319_2025_485_MOESM9_ESM.zip › Figure 6/6I/Fig.6I_1,6_Hex_r_tubulin.tif]

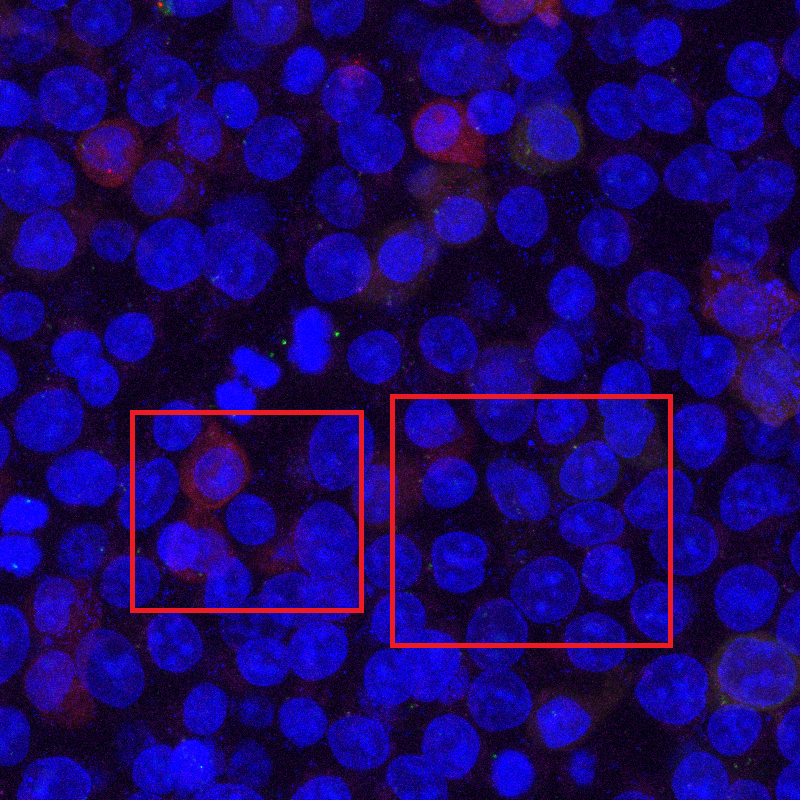

Supplement: Supplementary file 9 — Source data Fig. 6 [file 44319_2025_485_MOESM9_ESM.zip › Figure 6/6I/Fig.6I_1,6_Hex_SAS_6_mCherry_CEP192.tif]

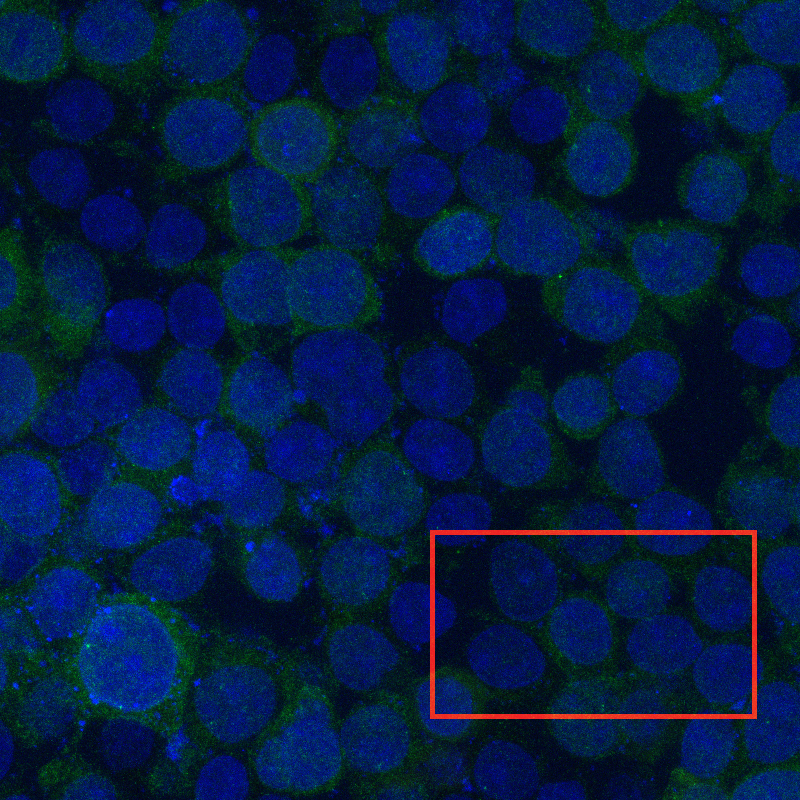

Supplement: Supplementary file 9 — Source data Fig. 6 [file 44319_2025_485_MOESM9_ESM.zip › Figure 6/6I/Fig.6I_1,6_Hex_SASS_6.tif]

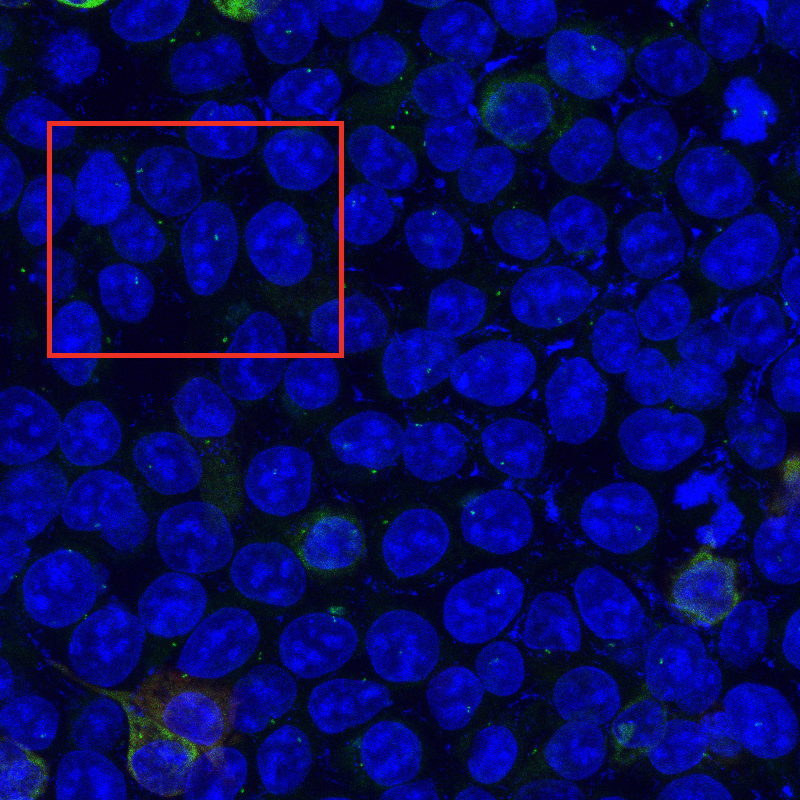

Supplement: Supplementary file 9 — Source data Fig. 6 [file 44319_2025_485_MOESM9_ESM.zip › Figure 6/6I/Fig.6I_control_r_tubulin.tif]

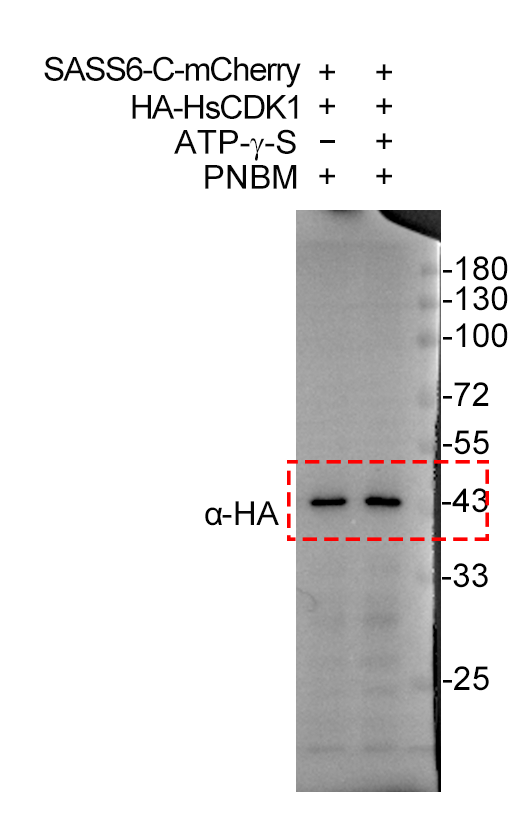

Supplement: Supplementary file 9 — Source data Fig. 6 [file 44319_2025_485_MOESM9_ESM.zip › Figure 6/6G/Fig. 6G_HA.tif]

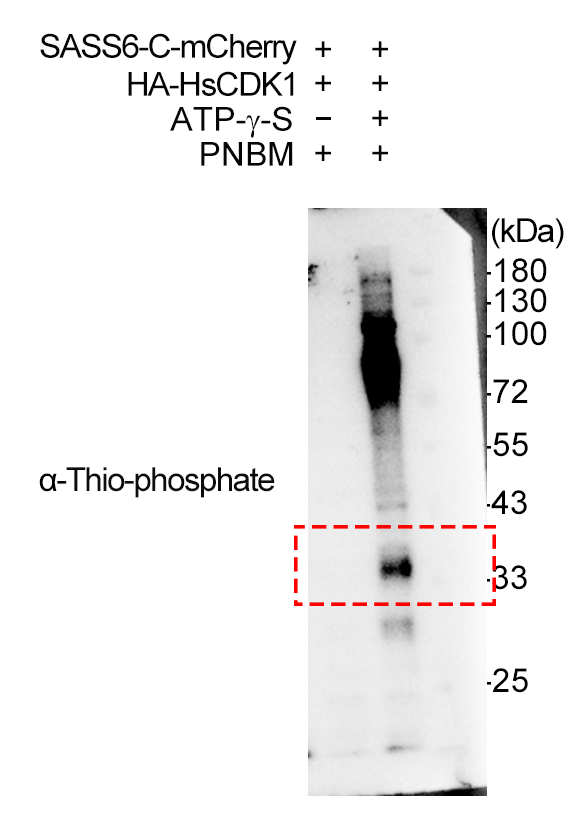

Supplement: Supplementary file 9 — Source data Fig. 6 [file 44319_2025_485_MOESM9_ESM.zip › Figure 6/6G/Fig. 6G_Thio.tif]

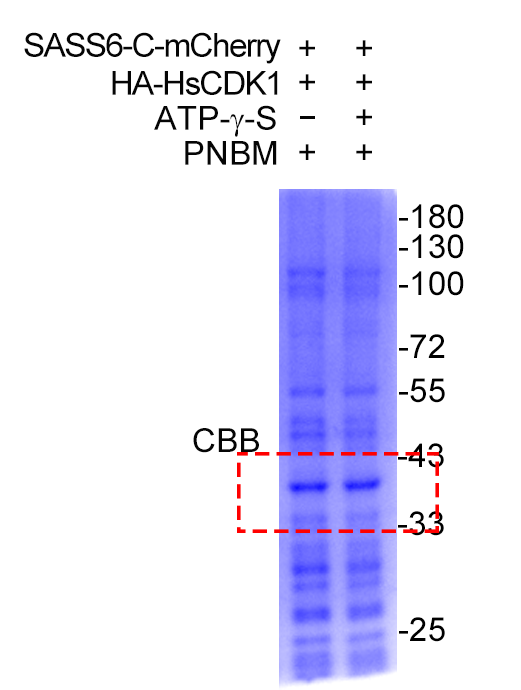

Supplement: Supplementary file 9 — Source data Fig. 6 [file 44319_2025_485_MOESM9_ESM.zip › Figure 6/6G/Fig. 6G_CBB.tif]

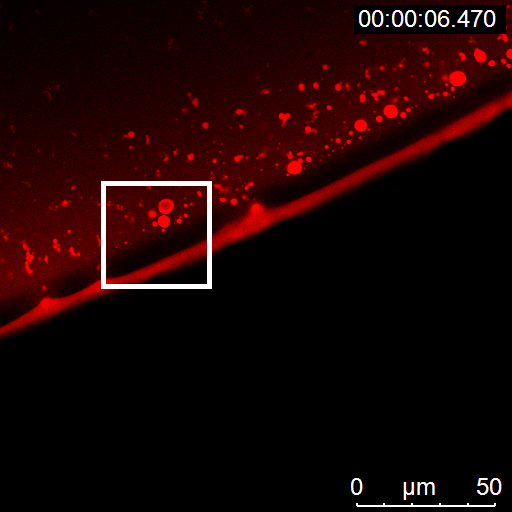

Supplement: Supplementary file 9 — Source data Fig. 6 [file 44319_2025_485_MOESM9_ESM.zip › Figure 6/6E/Fig. 6E-6.tif]

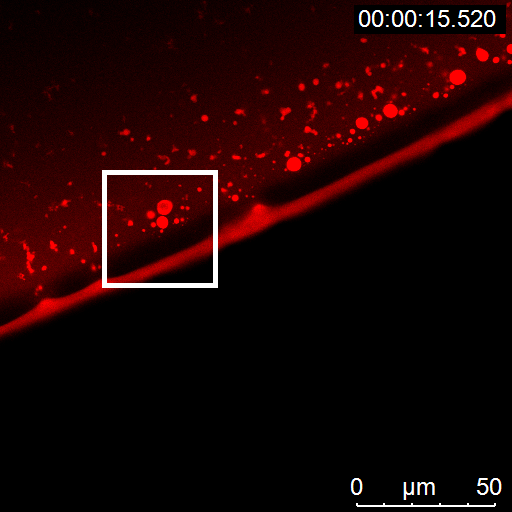

Supplement: Supplementary file 9 — Source data Fig. 6 [file 44319_2025_485_MOESM9_ESM.zip › Figure 6/6E/Fig. 6E-15.tif]

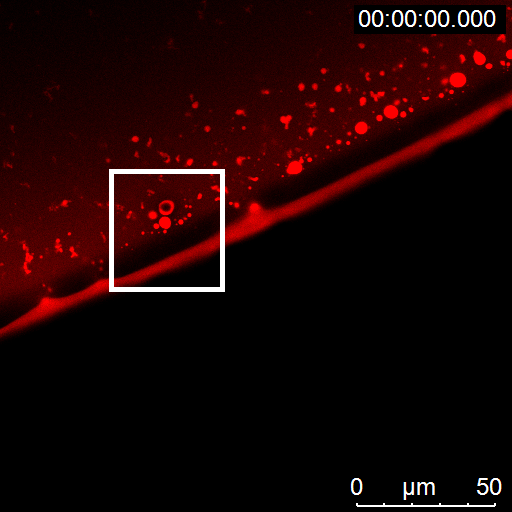

Supplement: Supplementary file 9 — Source data Fig. 6 [file 44319_2025_485_MOESM9_ESM.zip › Figure 6/6E/Fig. 6E-0.tif]

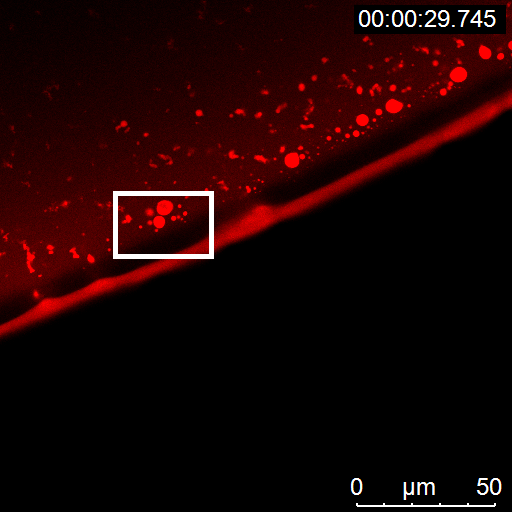

Supplement: Supplementary file 9 — Source data Fig. 6 [file 44319_2025_485_MOESM9_ESM.zip › Figure 6/6E/Fig. 6E-30.tif]

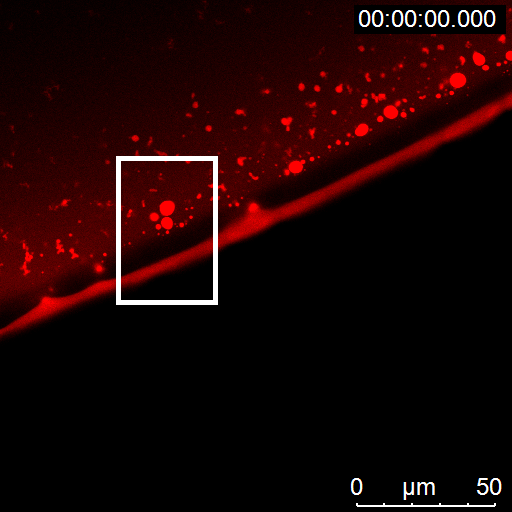

Supplement: Supplementary file 9 — Source data Fig. 6 [file 44319_2025_485_MOESM9_ESM.zip › Figure 6/6E/Fig. 6E--2.tif]

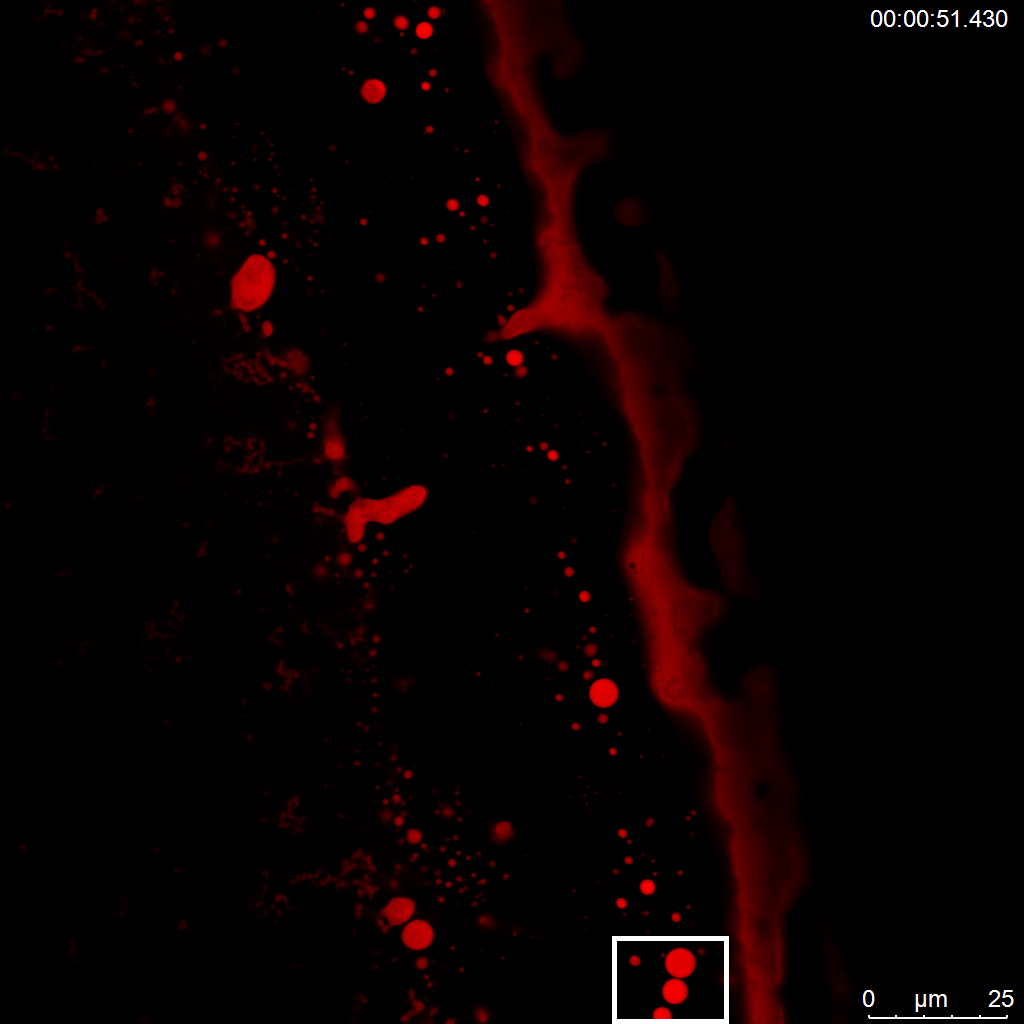

Supplement: Supplementary file 9 — Source data Fig. 6 [file 44319_2025_485_MOESM9_ESM.zip › Figure 6/6D/Fig. 6D_10_mCherry.tif]

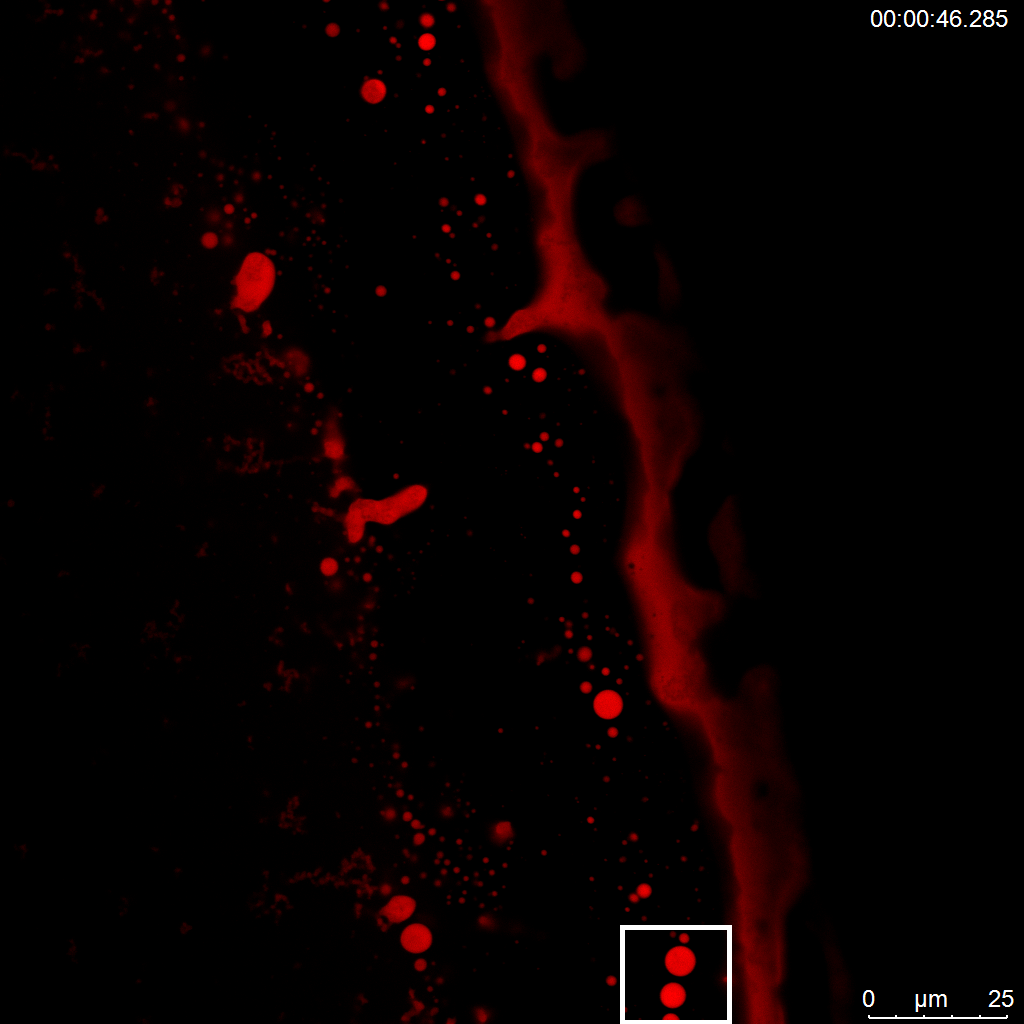

Supplement: Supplementary file 9 — Source data Fig. 6 [file 44319_2025_485_MOESM9_ESM.zip › Figure 6/6D/Fig. 6D_5_mCherry.tif]

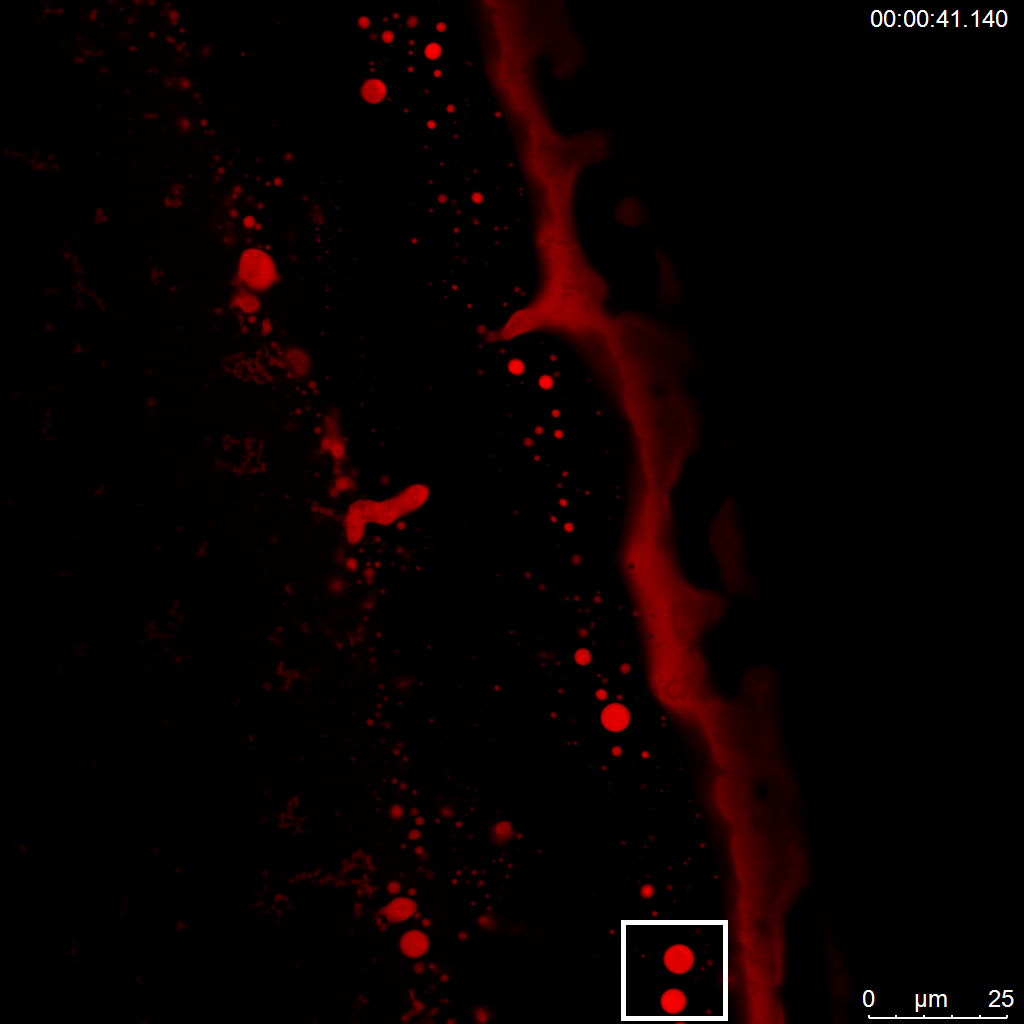

Supplement: Supplementary file 9 — Source data Fig. 6 [file 44319_2025_485_MOESM9_ESM.zip › Figure 6/6D/Fig. 6D_0_mCherry.tif]

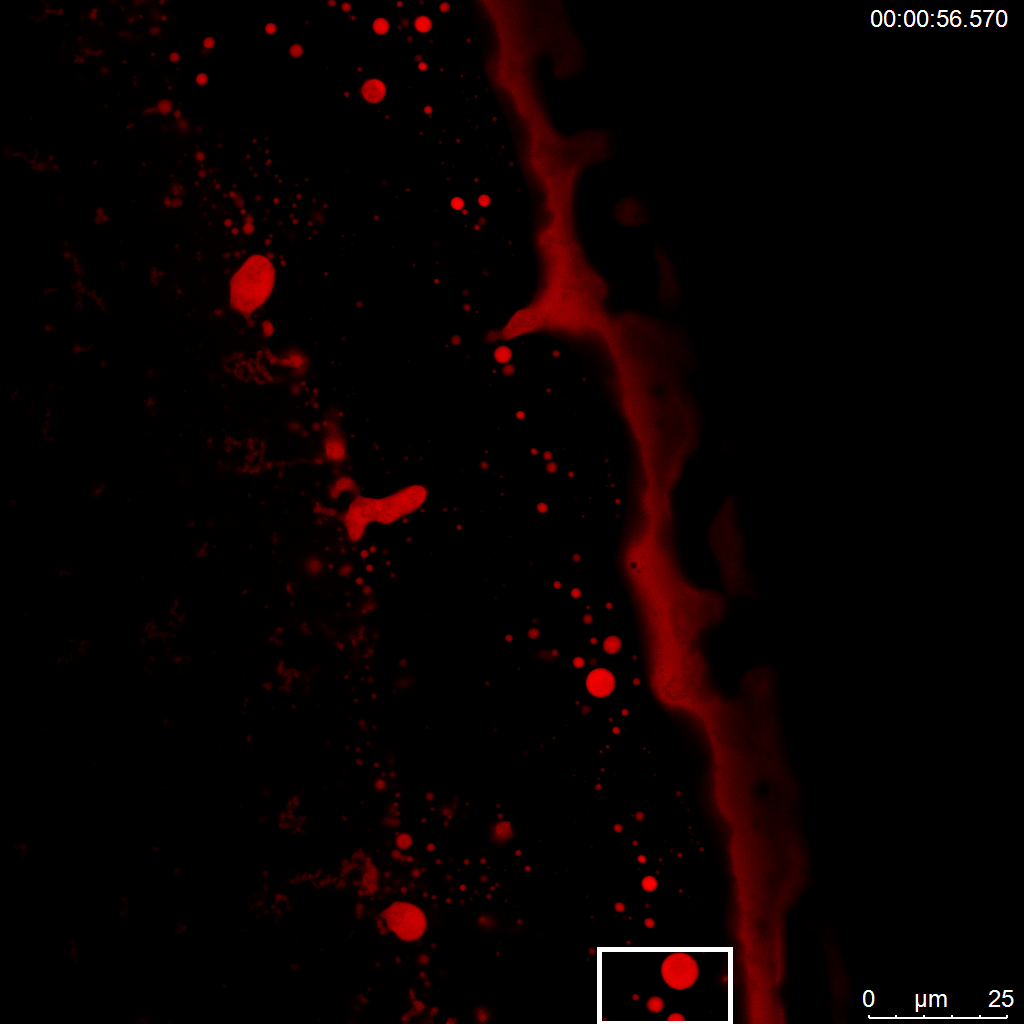

Supplement: Supplementary file 9 — Source data Fig. 6 [file 44319_2025_485_MOESM9_ESM.zip › Figure 6/6D/Fig. 6D_15_mCherry.tif]

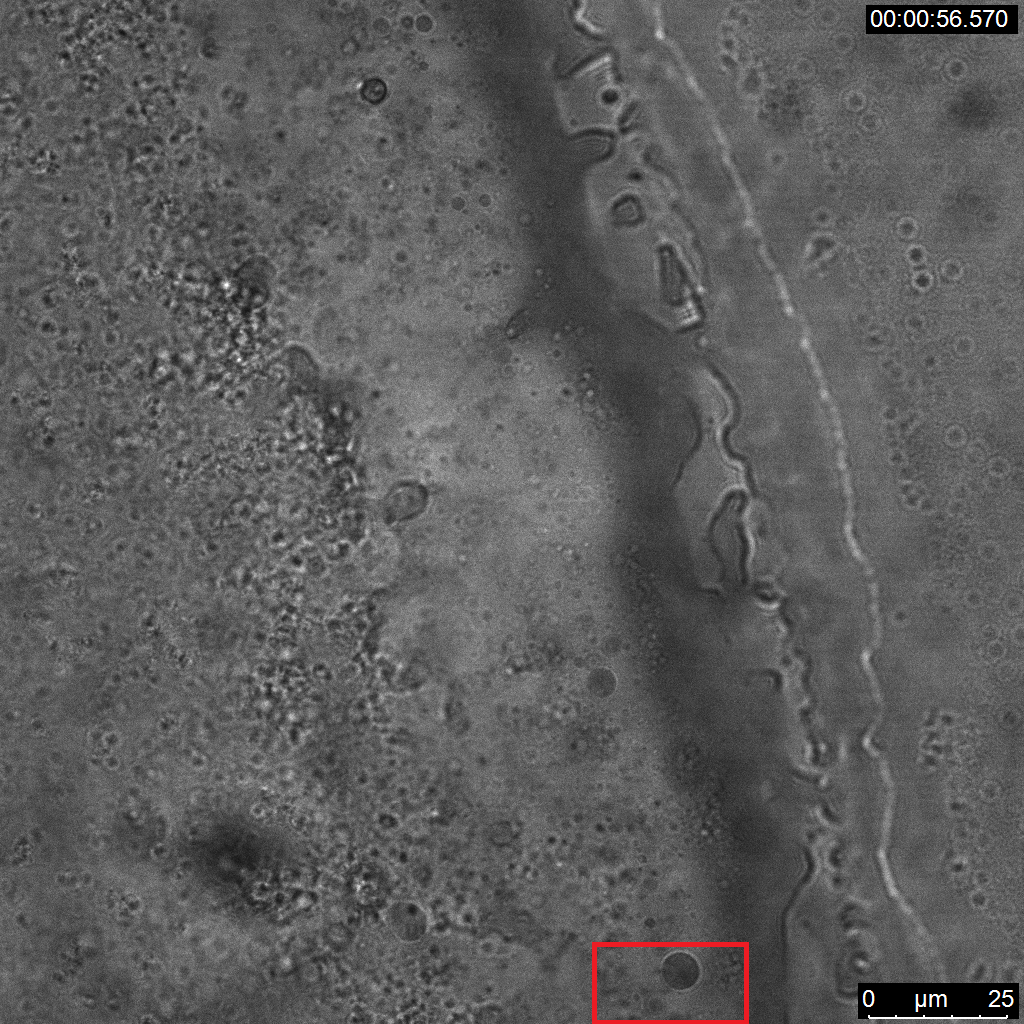

Supplement: Supplementary file 9 — Source data Fig. 6 [file 44319_2025_485_MOESM9_ESM.zip › Figure 6/6D/Fig. 6D_15_BF.tif]

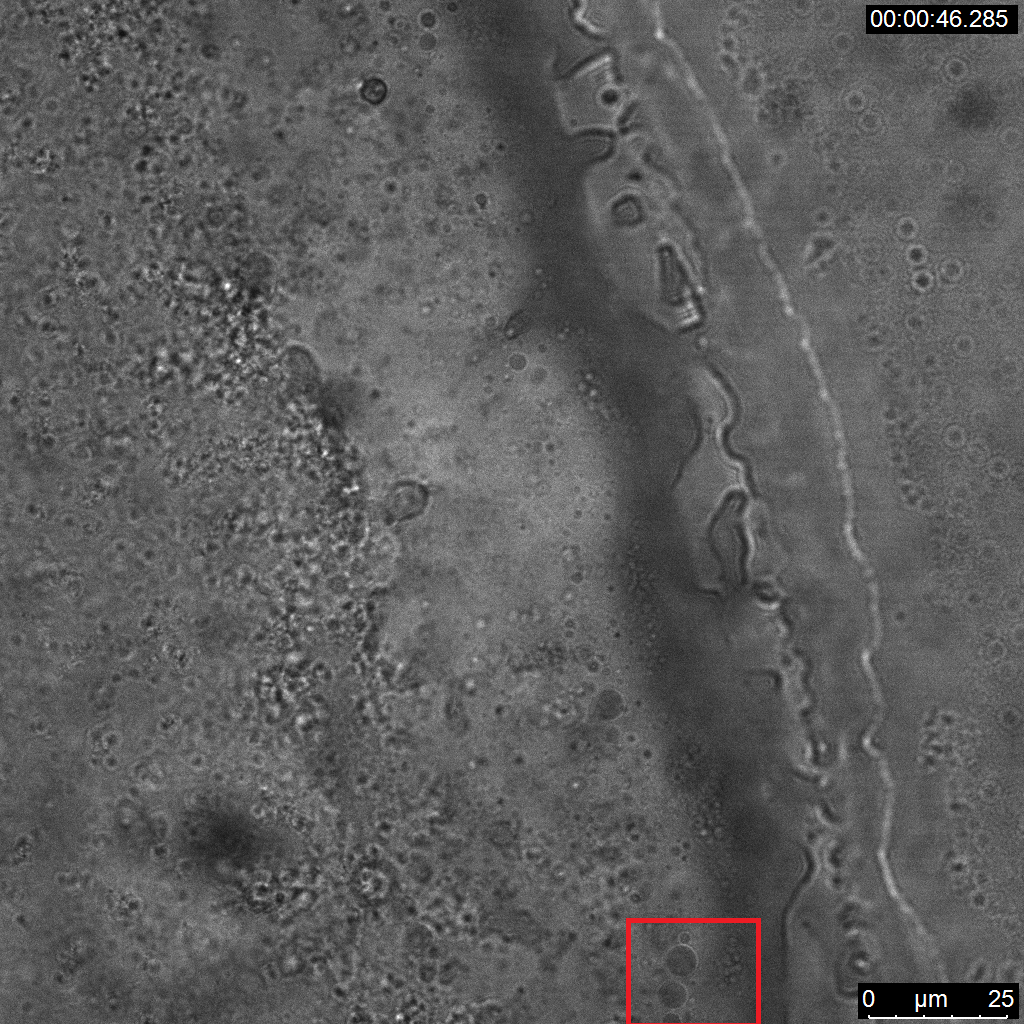

Supplement: Supplementary file 9 — Source data Fig. 6 [file 44319_2025_485_MOESM9_ESM.zip › Figure 6/6D/Fig. 6D_5_BF.tif]

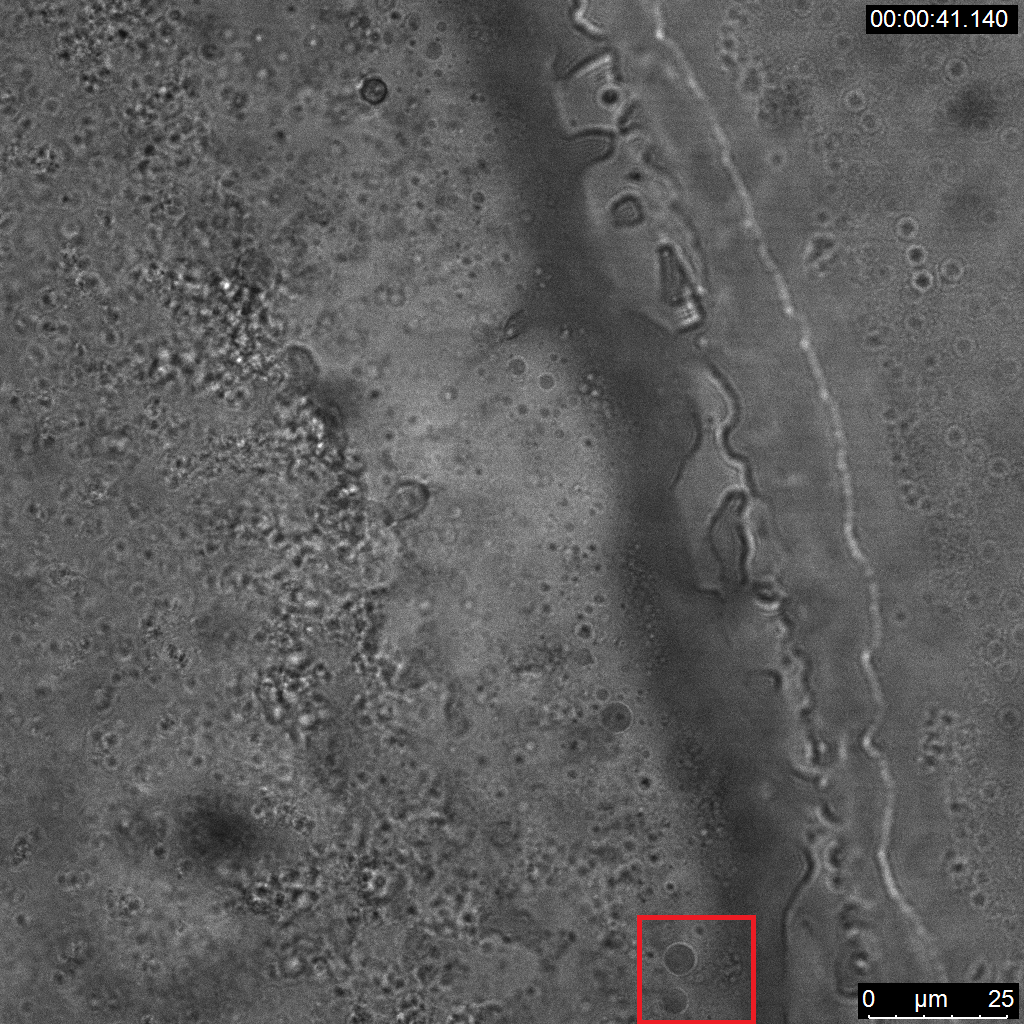

Supplement: Supplementary file 9 — Source data Fig. 6 [file 44319_2025_485_MOESM9_ESM.zip › Figure 6/6D/Fig. 6D_0_BF.tif]

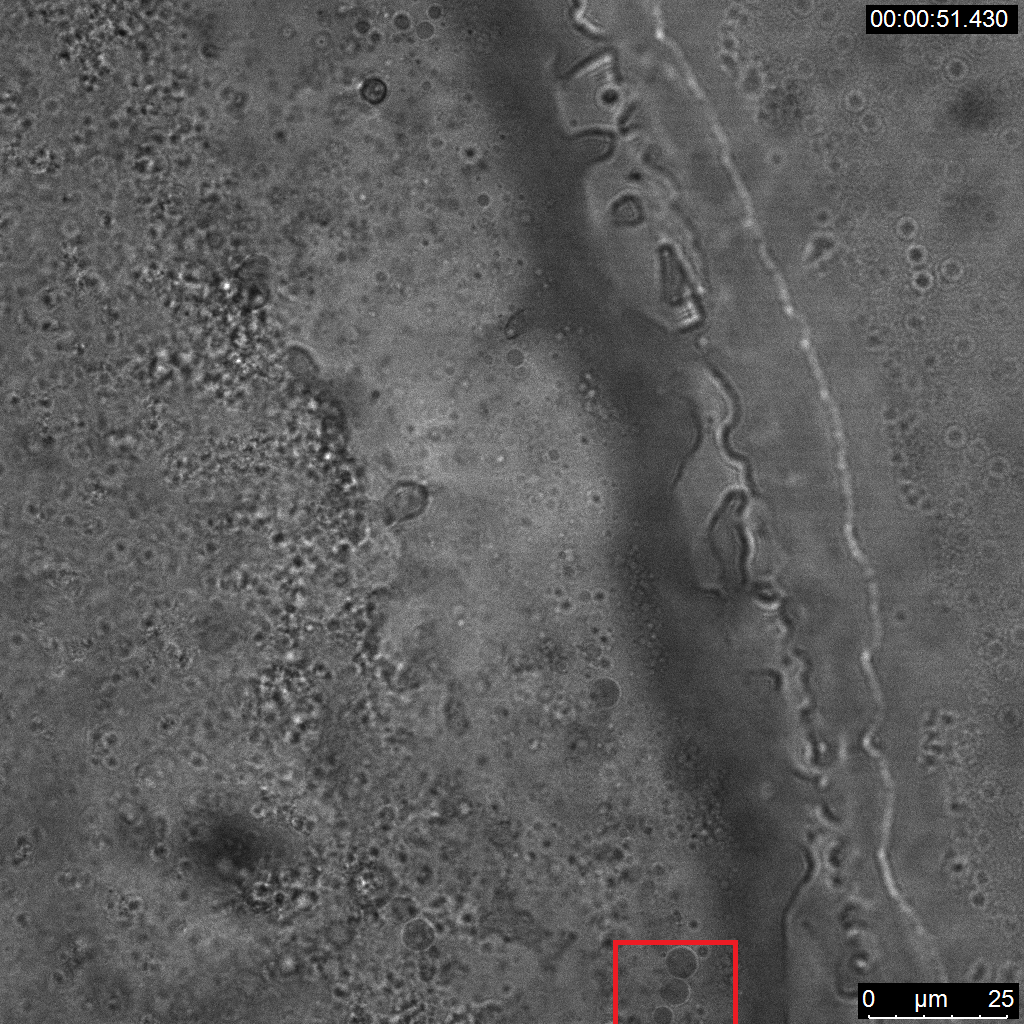

Supplement: Supplementary file 9 — Source data Fig. 6 [file 44319_2025_485_MOESM9_ESM.zip › Figure 6/6D/Fig. 6D_10_BF.tif]

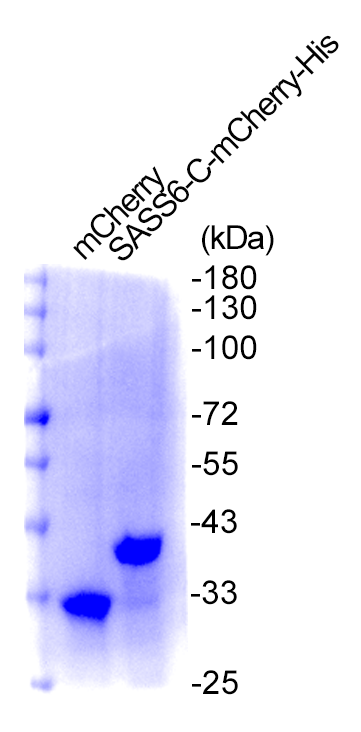

Supplement: Supplementary file 9 — Source data Fig. 6 [file 44319_2025_485_MOESM9_ESM.zip › Figure 6/6C/Fig. 6C.tif]

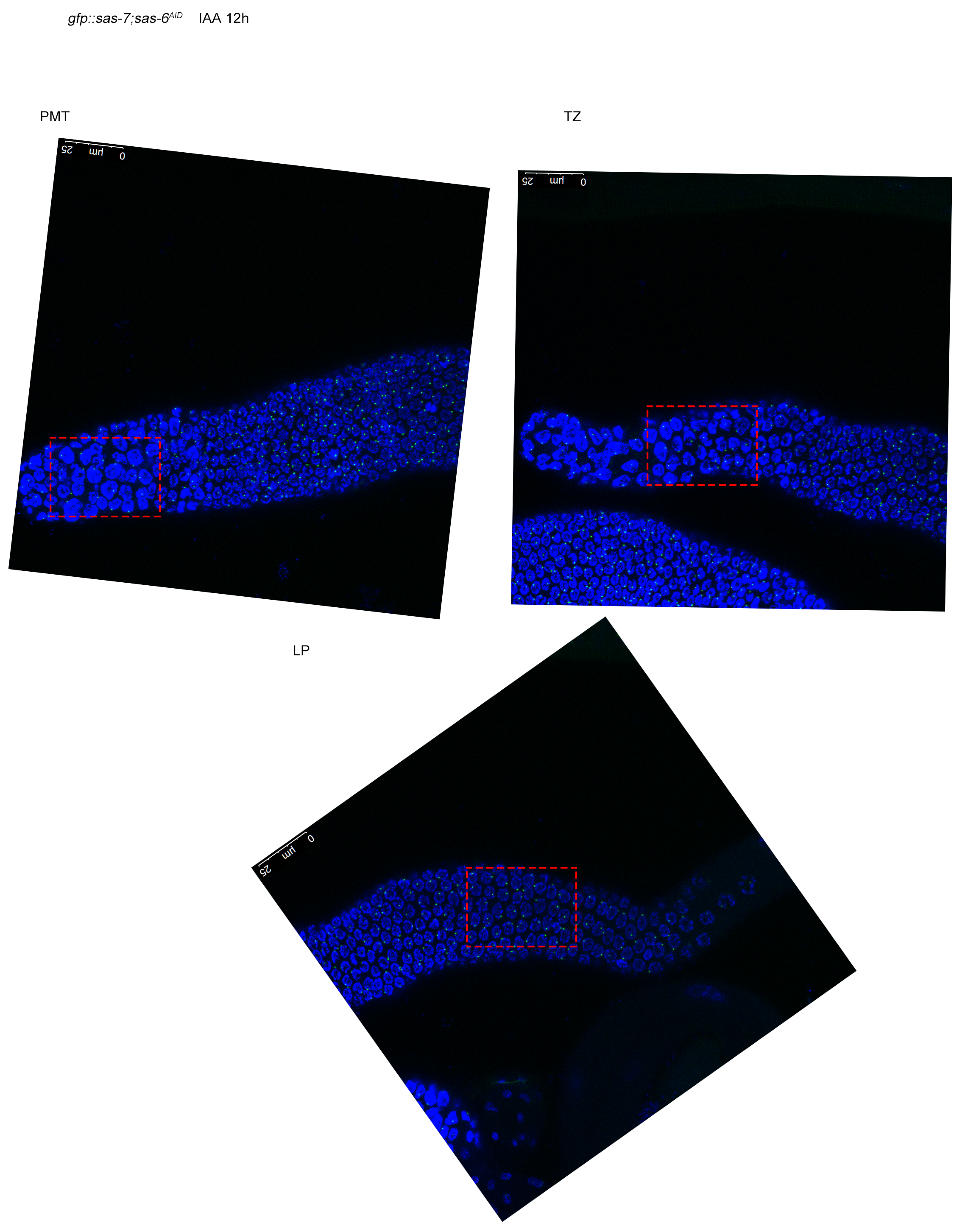

Supplement: Supplementary file 10 — Source data Fig. 7 [file 44319_2025_485_MOESM10_ESM.zip › Figure 7/7C/7C_gfp_sas_7;sas-6AID_PMT_LP_IAA_12h.tif]

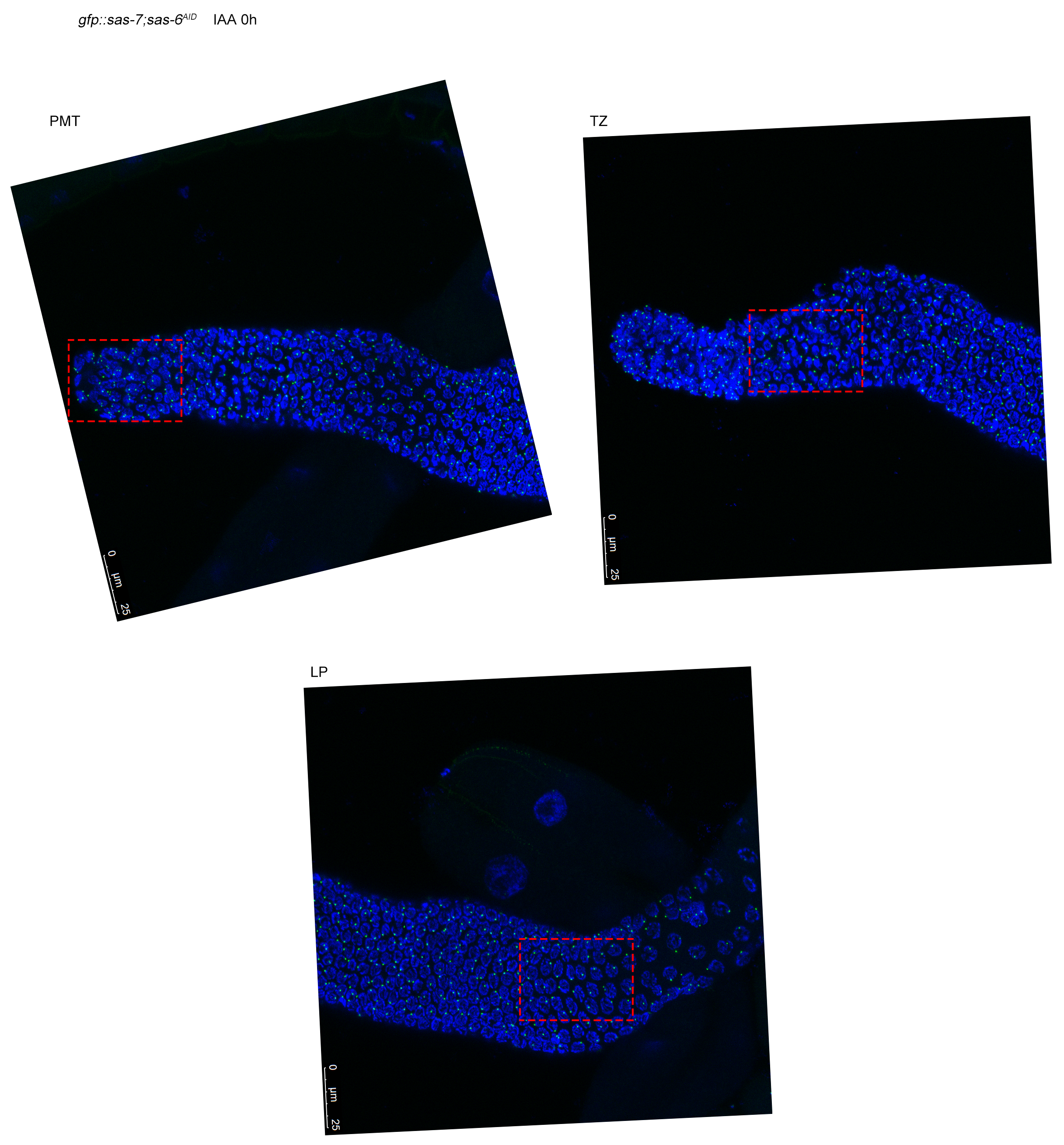

Supplement: Supplementary file 10 — Source data Fig. 7 [file 44319_2025_485_MOESM10_ESM.zip › Figure 7/7C/7C_gfp_sas_7;sas-6AID_PMT_LP_Control.tif]

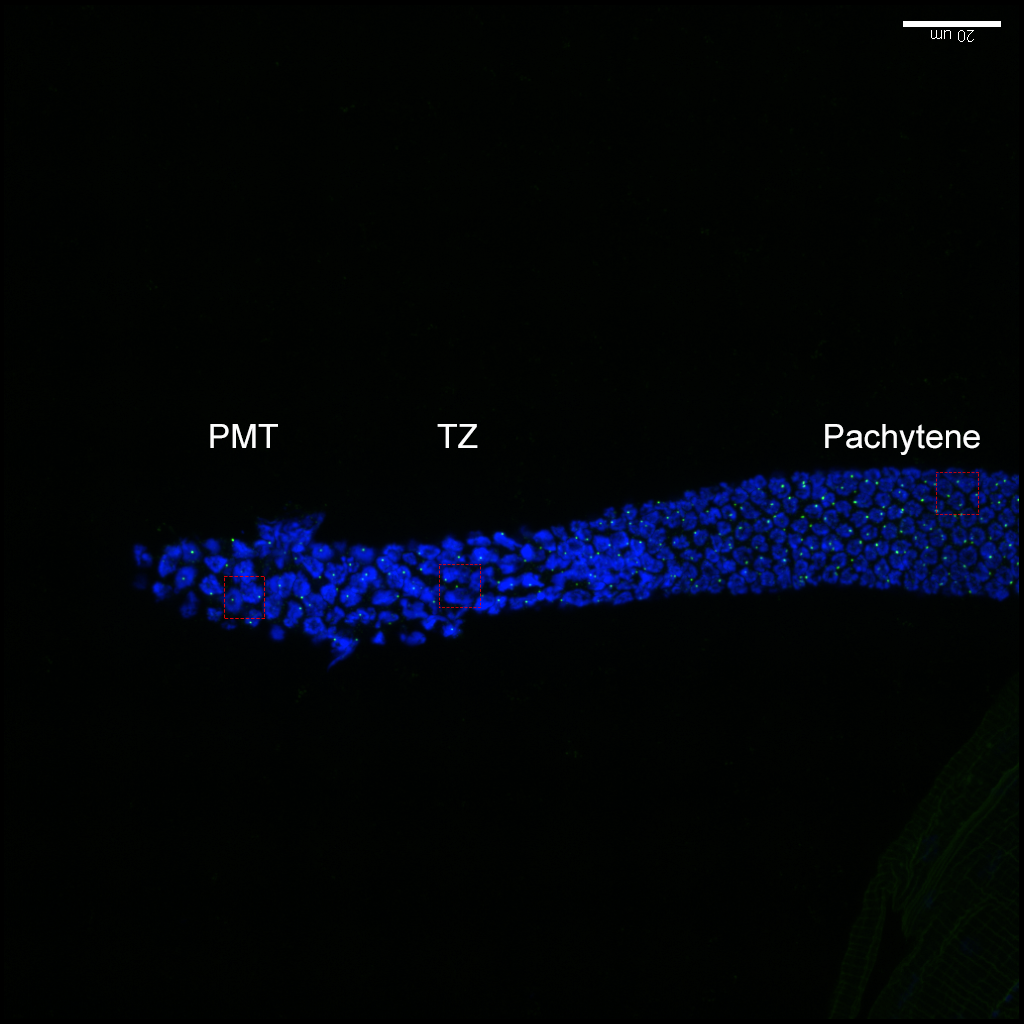

Supplement: Supplementary file 10 — Source data Fig. 7 [file 44319_2025_485_MOESM10_ESM.zip › Figure 7/7B/7B_sas_6AID_IAA 12h.tif]

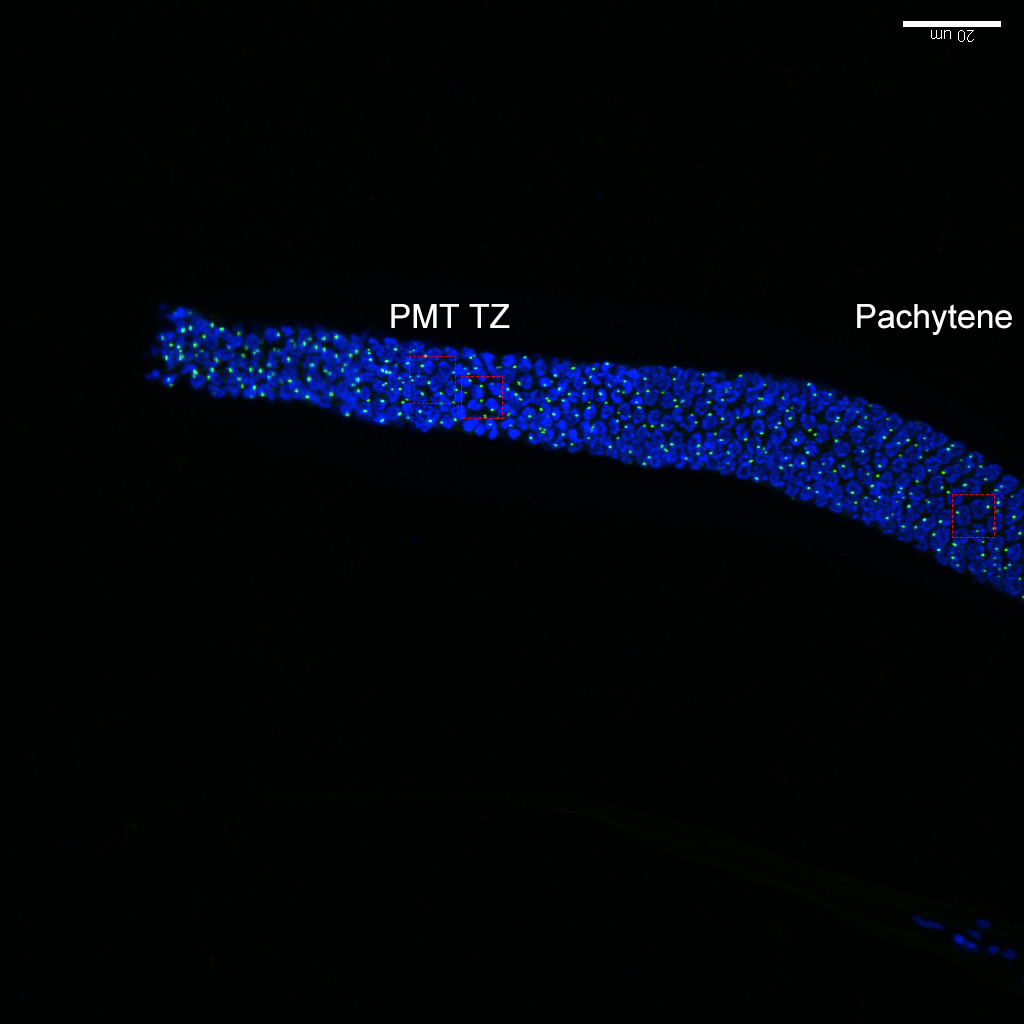

Supplement: Supplementary file 10 — Source data Fig. 7 [file 44319_2025_485_MOESM10_ESM.zip › Figure 7/7B/7B_sas_6AID_IAA 0h.tif]

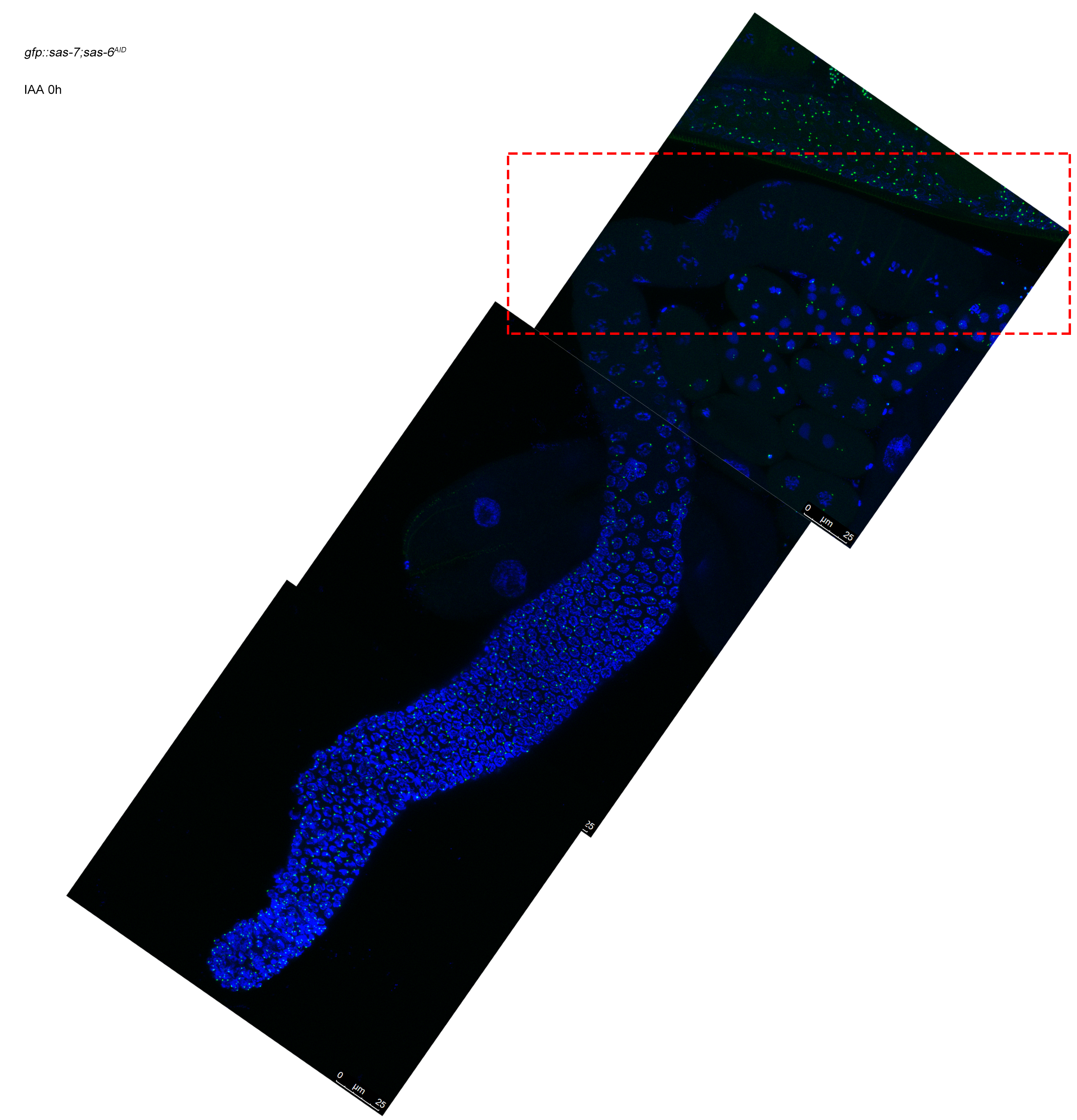

Supplement: Supplementary file 10 — Source data Fig. 7 [file 44319_2025_485_MOESM10_ESM.zip › Figure 7/7E/7E_gfp_sas_7;sas-6AID_Control.tif]

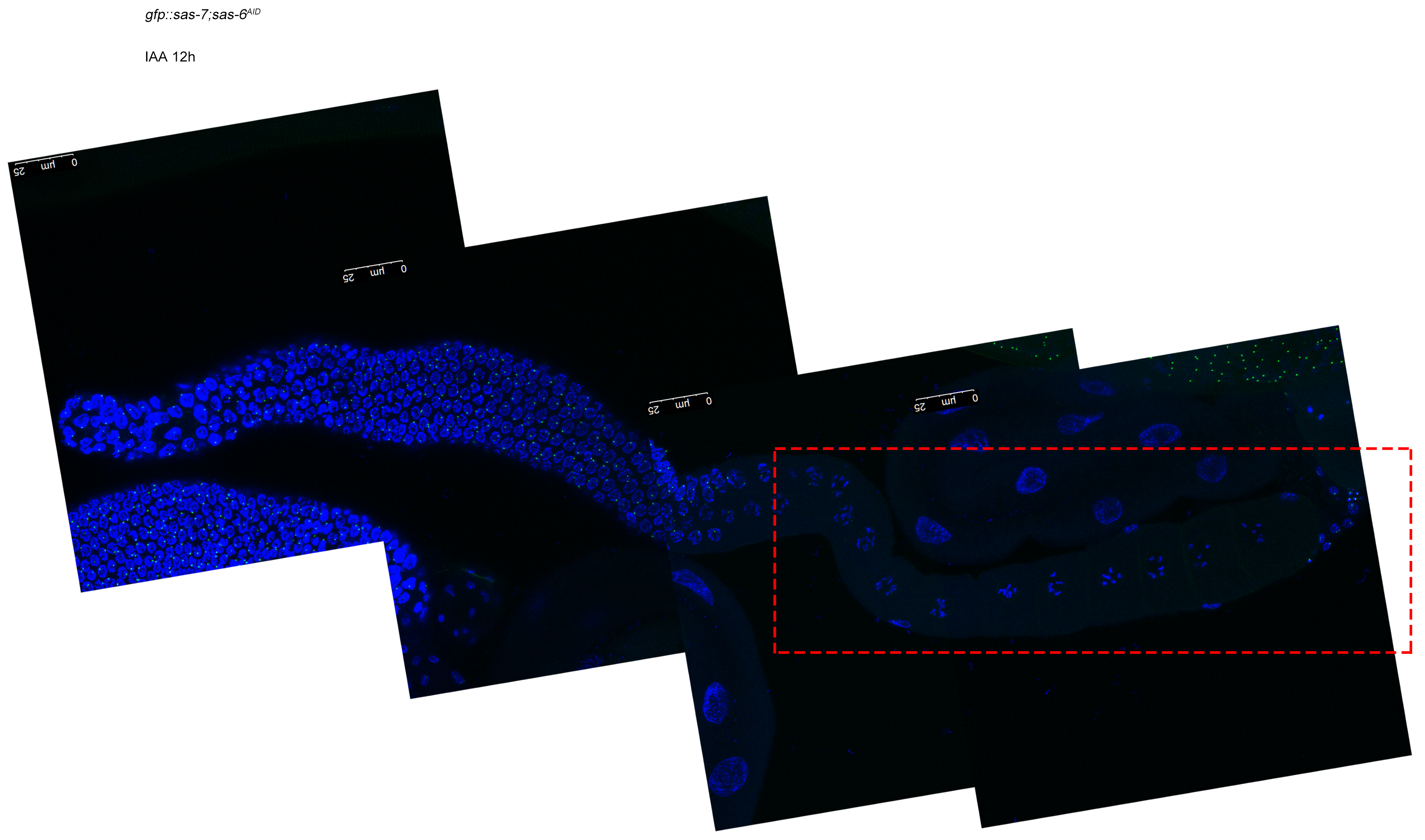

Supplement: Supplementary file 10 — Source data Fig. 7 [file 44319_2025_485_MOESM10_ESM.zip › Figure 7/7E/7E_gfp_sas_7;sas-6AID_IAA_12h.tif]

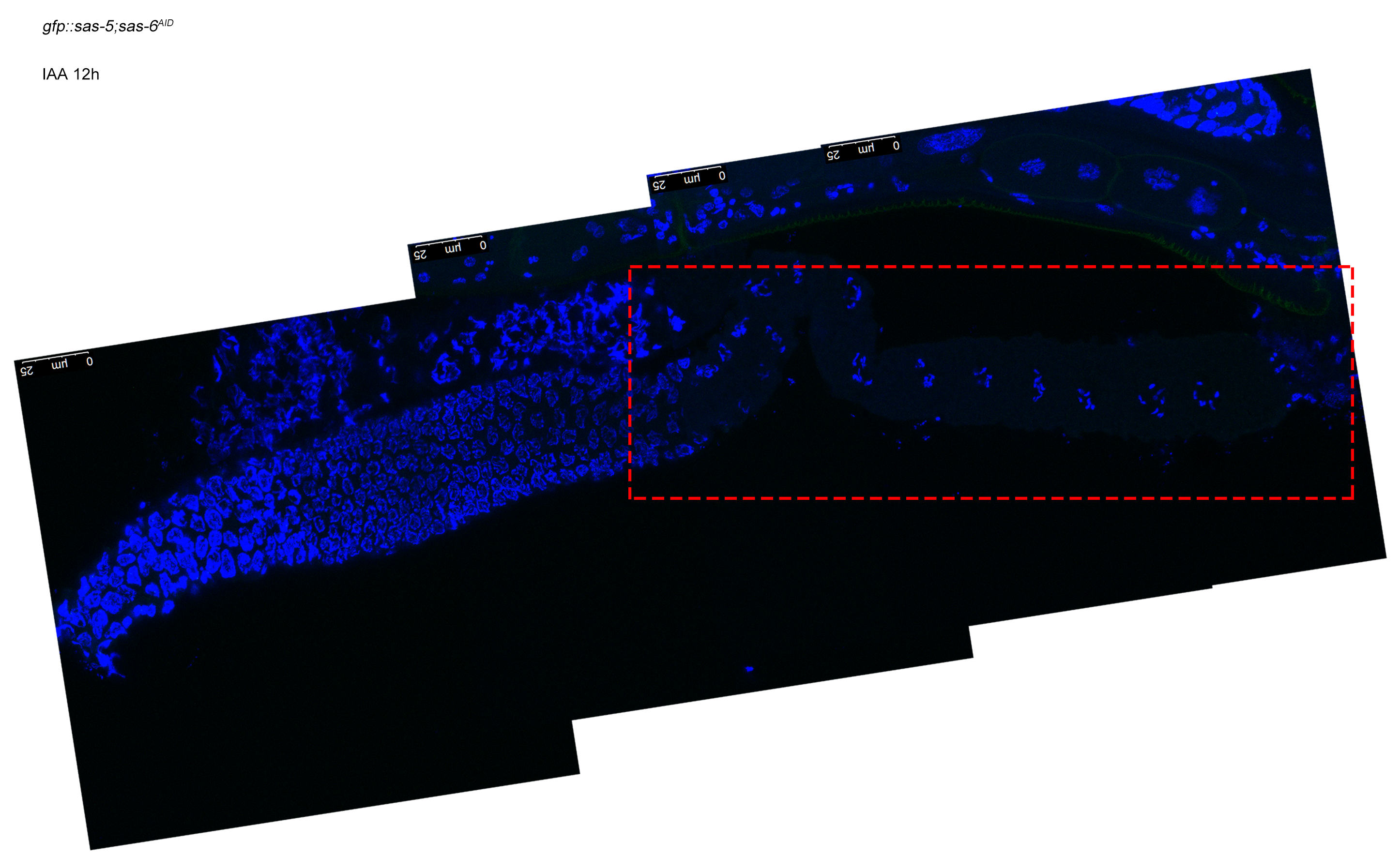

Supplement: Supplementary file 10 — Source data Fig. 7 [file 44319_2025_485_MOESM10_ESM.zip › Figure 7/7I/7I_gfp_sas_5;sas-6AID_IAA_12h.tif]

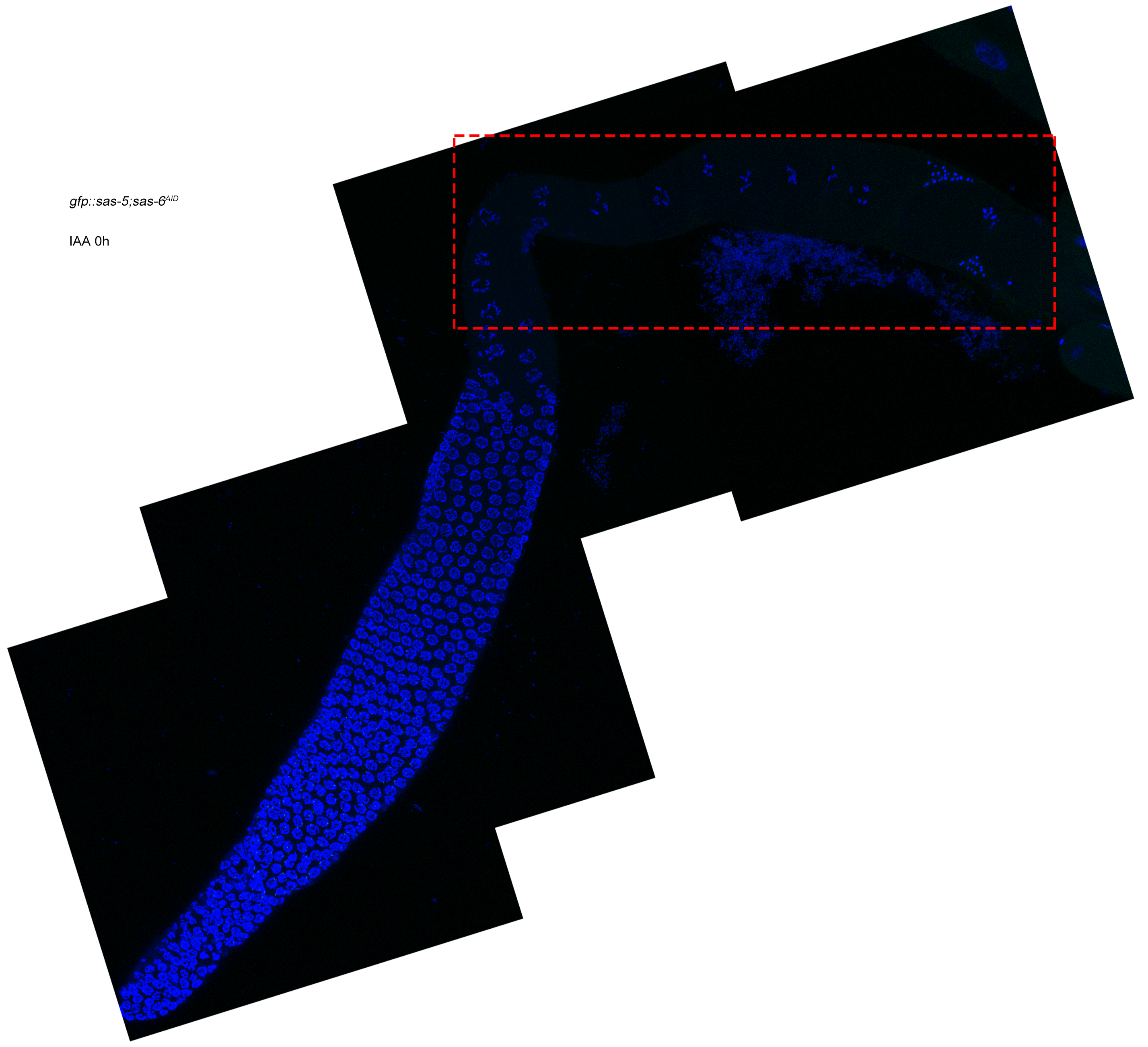

Supplement: Supplementary file 10 — Source data Fig. 7 [file 44319_2025_485_MOESM10_ESM.zip › Figure 7/7I/7I_gfp_sas_5;sas-6AID_Control.tif]

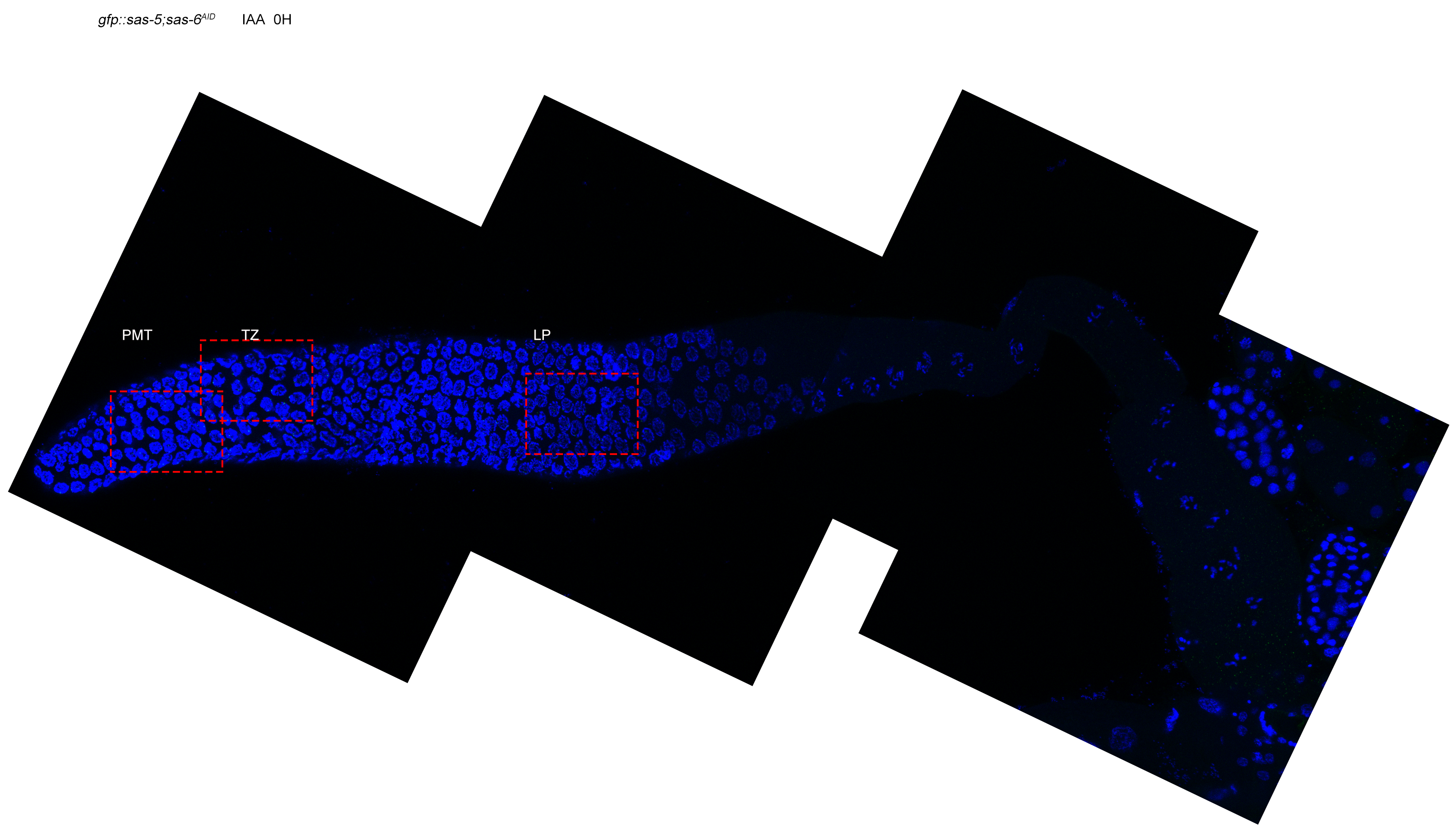

Supplement: Supplementary file 10 — Source data Fig. 7 [file 44319_2025_485_MOESM10_ESM.zip › Figure 7/7G/7G_gfp_sas_5;sas-6AID_PMT_LP_Control.tif]

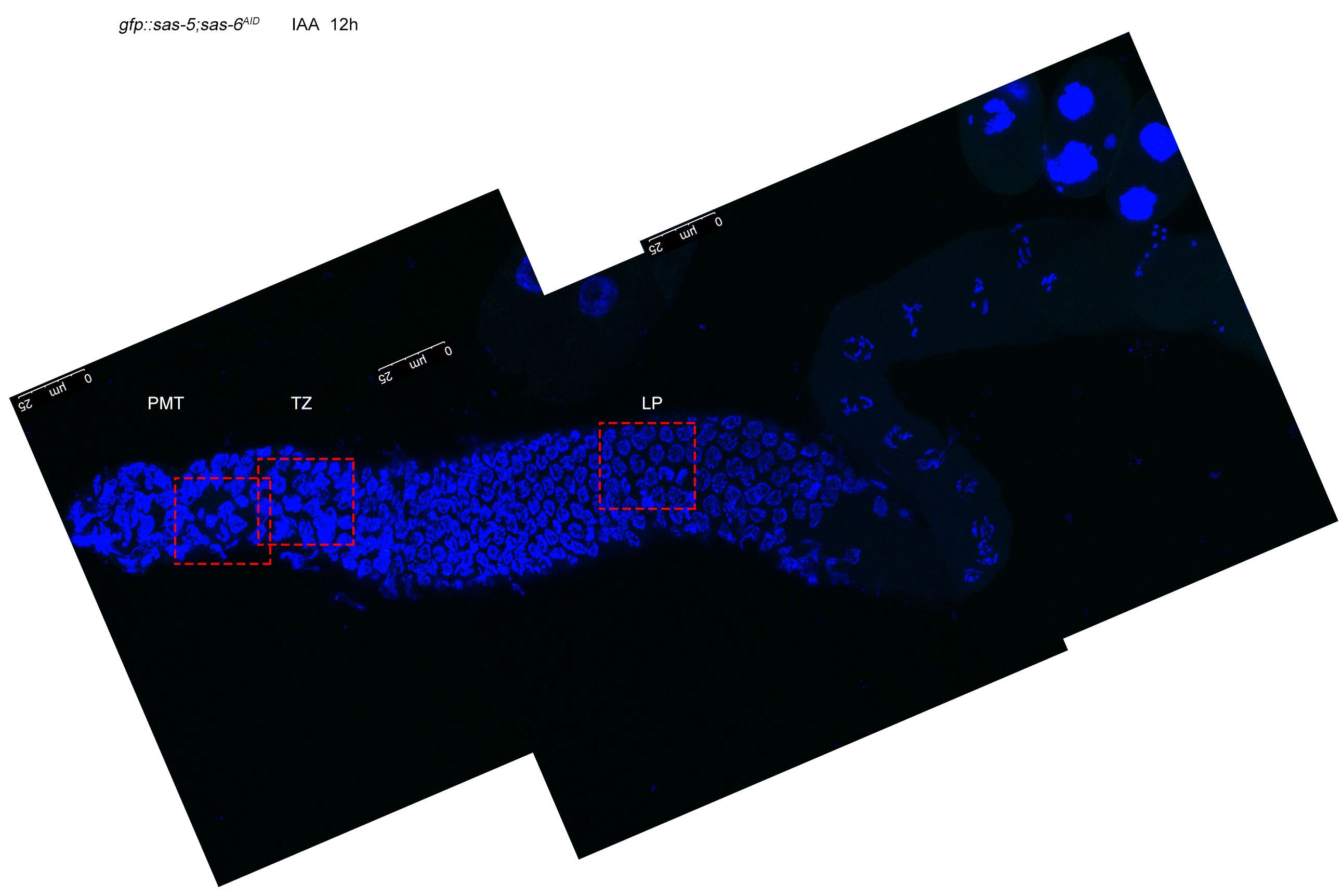

Supplement: Supplementary file 10 — Source data Fig. 7 [file 44319_2025_485_MOESM10_ESM.zip › Figure 7/7G/7G_gfp_sas_5;sas-6AID_PMT_LP_IAA_12h.tif]

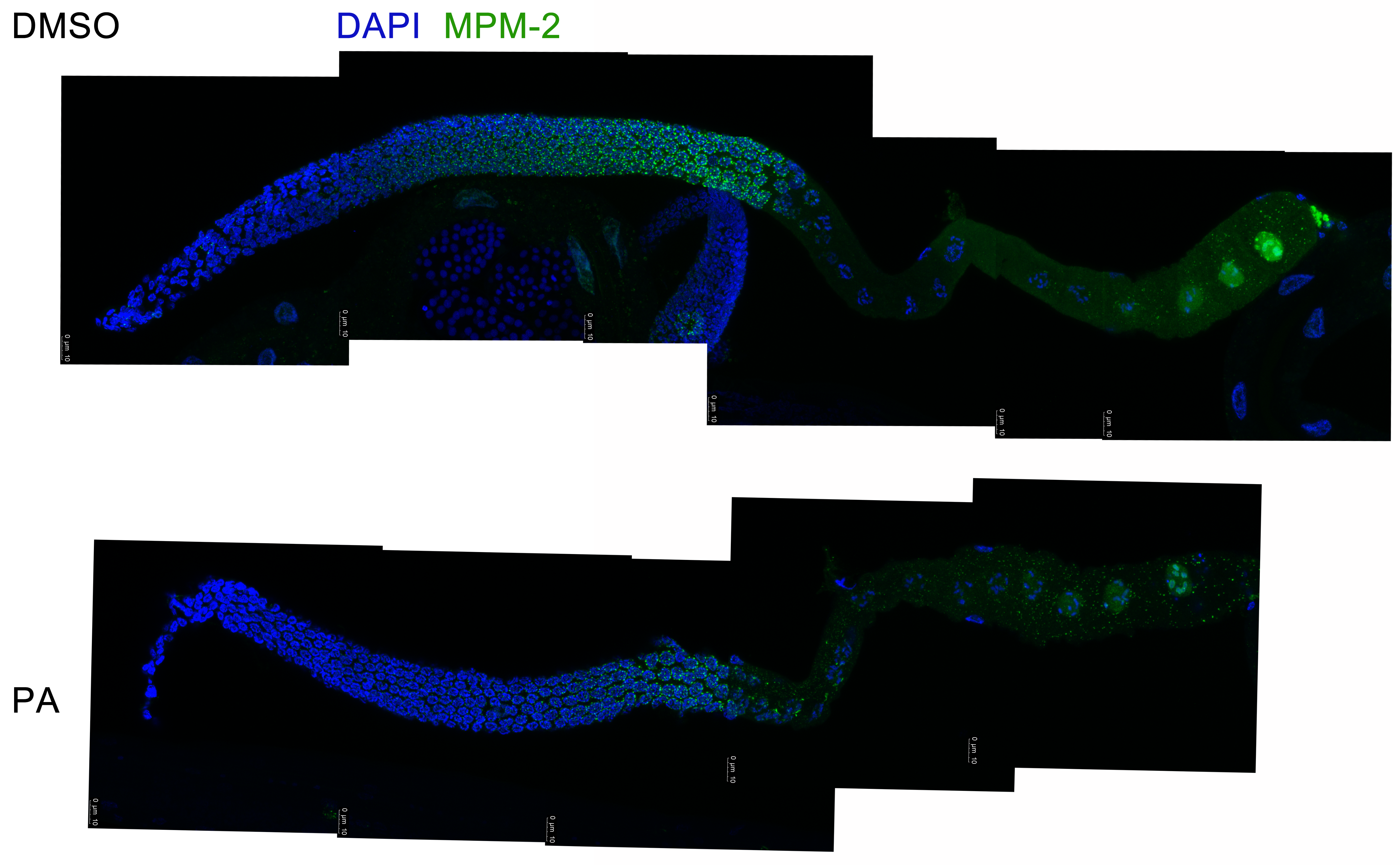

Supplement: Supplementary file 11 — Source data Fig. 8 [file 44319_2025_485_MOESM11_ESM.zip › Figure 8/8A/Fig.8A_DMSO_PA_MPM_2.tif]

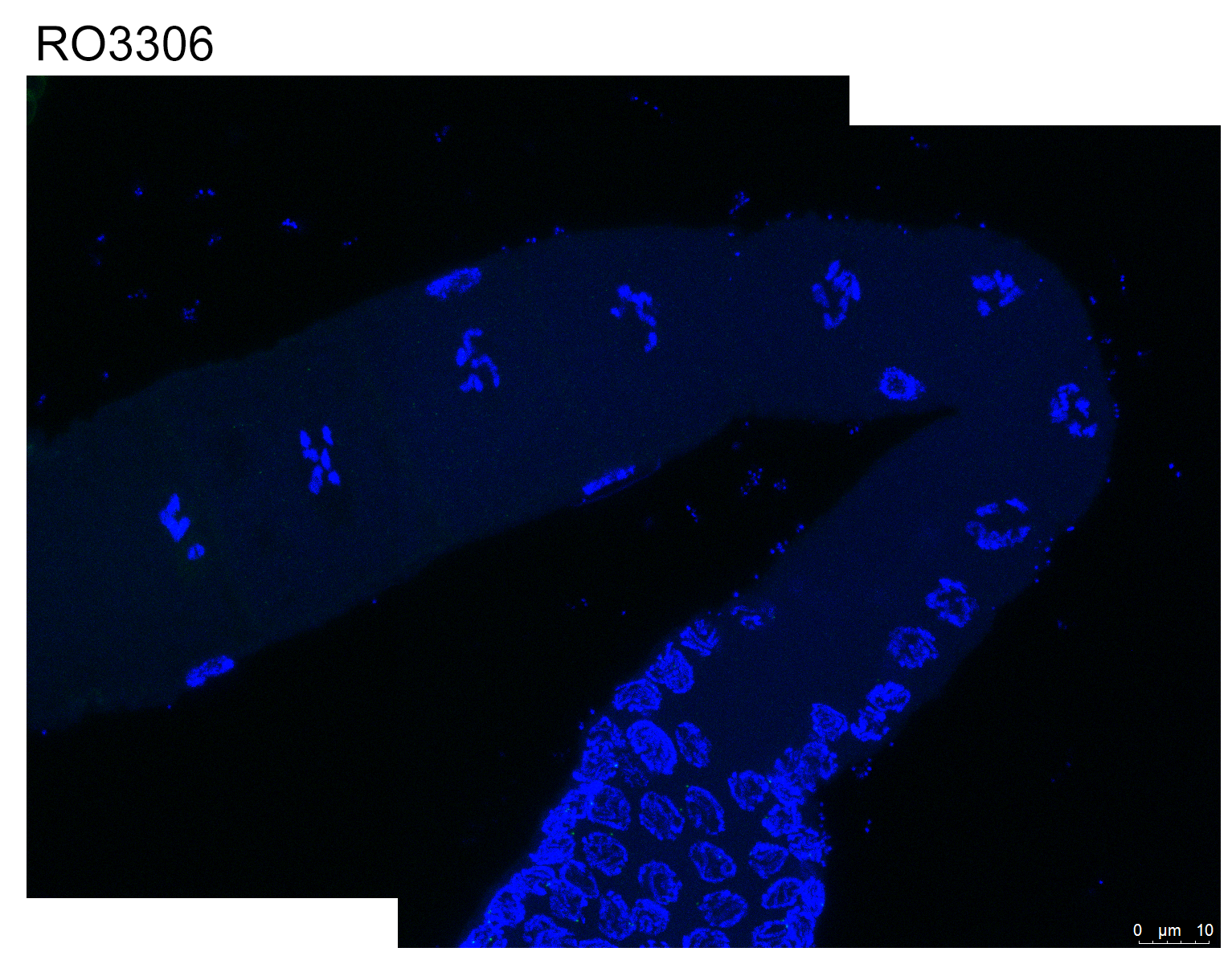

Supplement: Supplementary file 11 — Source data Fig. 8 [file 44319_2025_485_MOESM11_ESM.zip › Figure 8/8C/Fig. 8C_RO3306.tif]

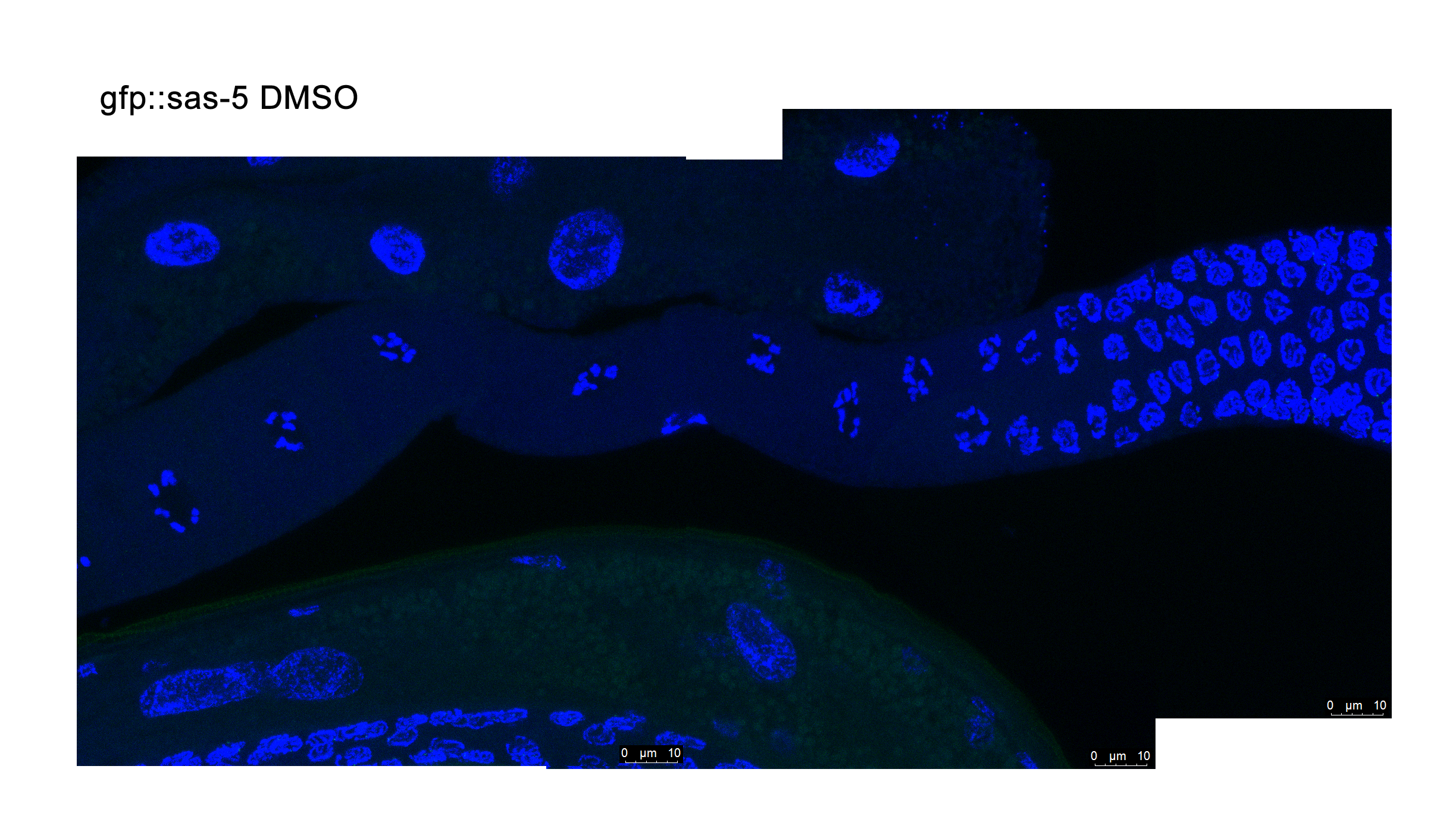

Supplement: Supplementary file 11 — Source data Fig. 8 [file 44319_2025_485_MOESM11_ESM.zip › Figure 8/8C/Fig. 8C_DMSO.tif]

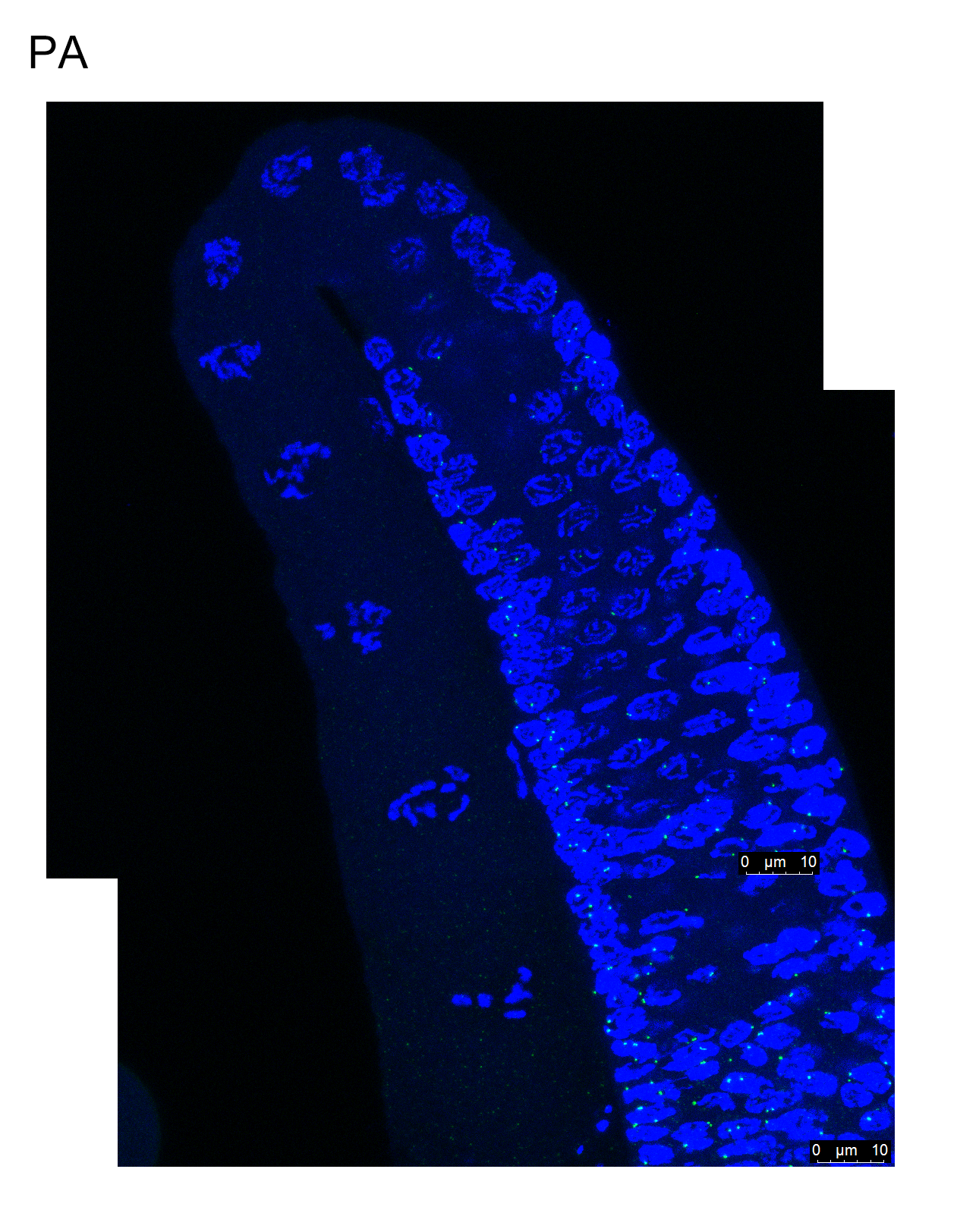

Supplement: Supplementary file 11 — Source data Fig. 8 [file 44319_2025_485_MOESM11_ESM.zip › Figure 8/8C/Fig. 8C_PA.tif]

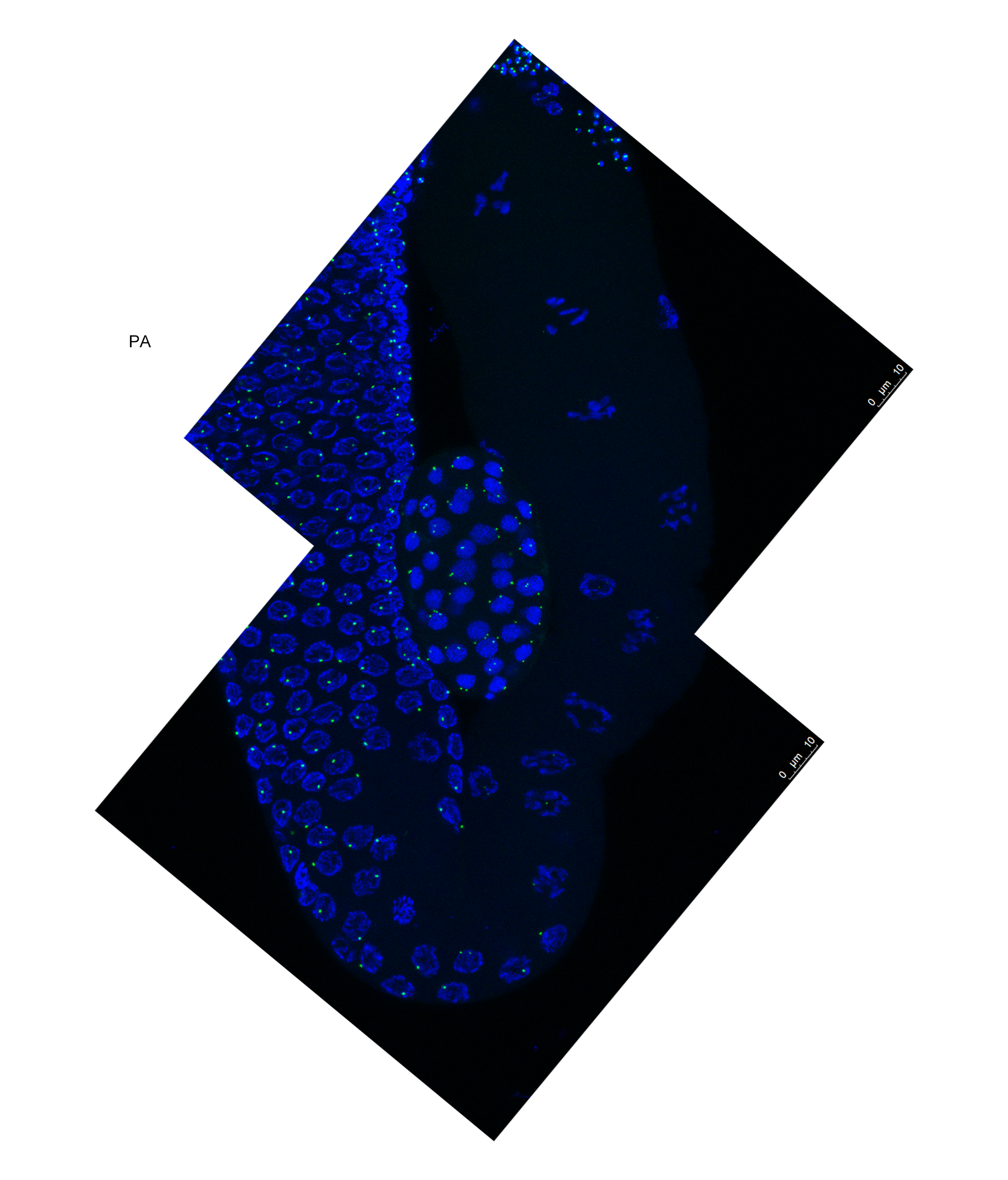

Supplement: Supplementary file 11 — Source data Fig. 8 [file 44319_2025_485_MOESM11_ESM.zip › Figure 8/8E/Fig. 8E_PA.tif]

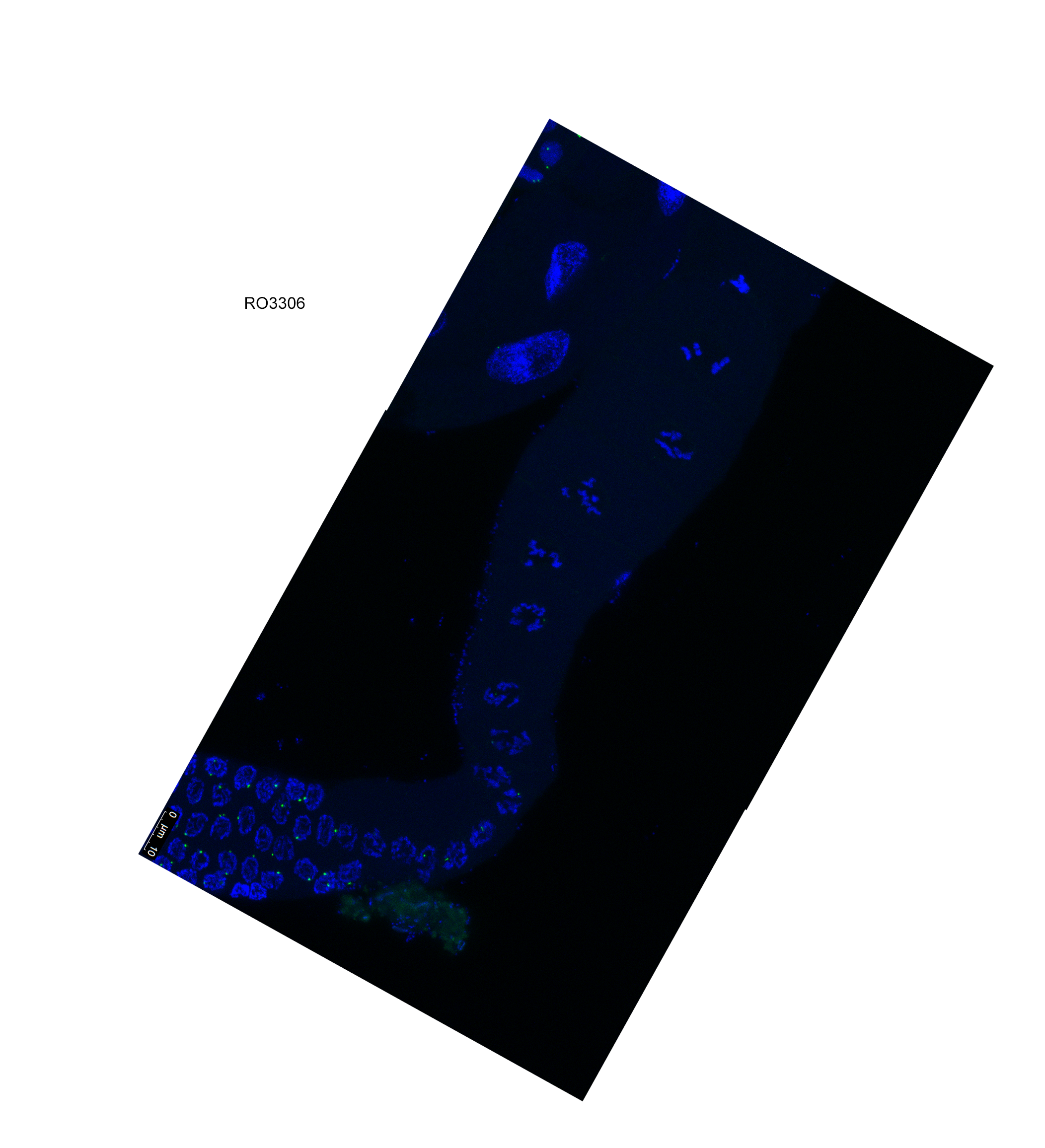

Supplement: Supplementary file 11 — Source data Fig. 8 [file 44319_2025_485_MOESM11_ESM.zip › Figure 8/8E/Fig. 8E_RO3306.tif]

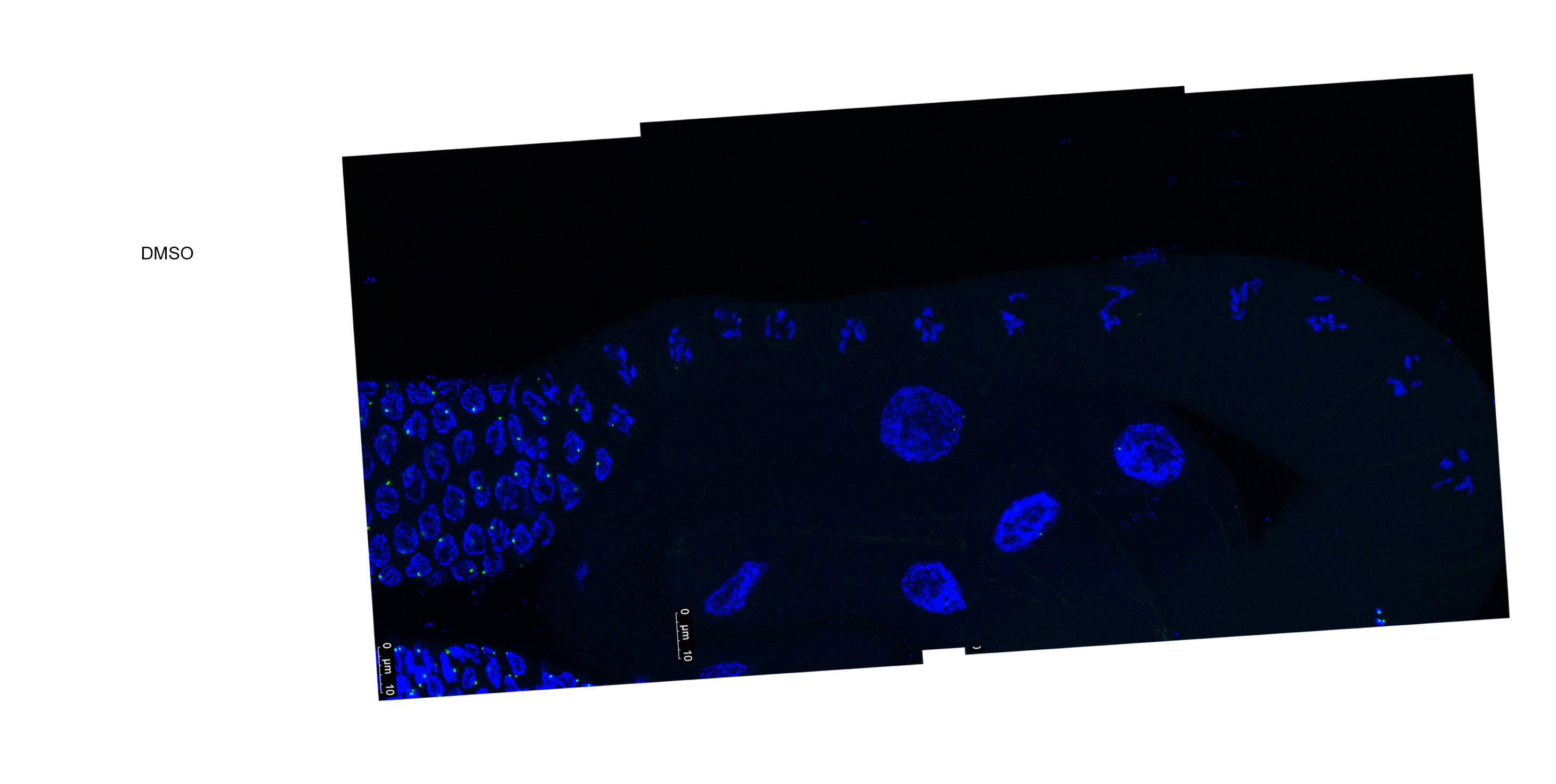

Supplement: Supplementary file 11 — Source data Fig. 8 [file 44319_2025_485_MOESM11_ESM.zip › Figure 8/8E/Fig. 8E_DMSO.tif]

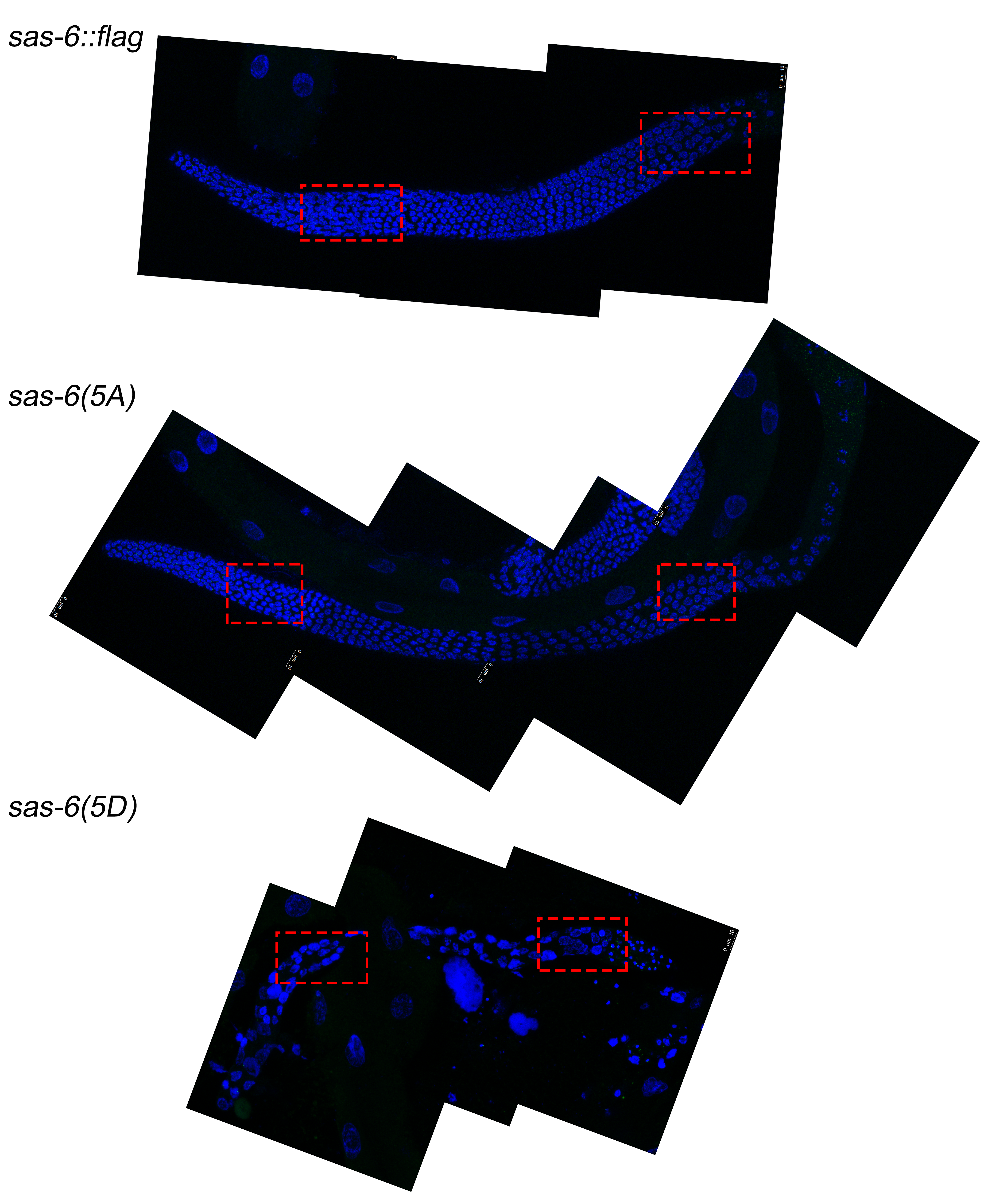

Supplement: Supplementary file 12 — Source data Fig. 9 [file 44319_2025_485_MOESM12_ESM.zip › Figure 9/9B/Fig.9B_wt_sas_6(5A)_sas_6(5D)_Flag.tif]

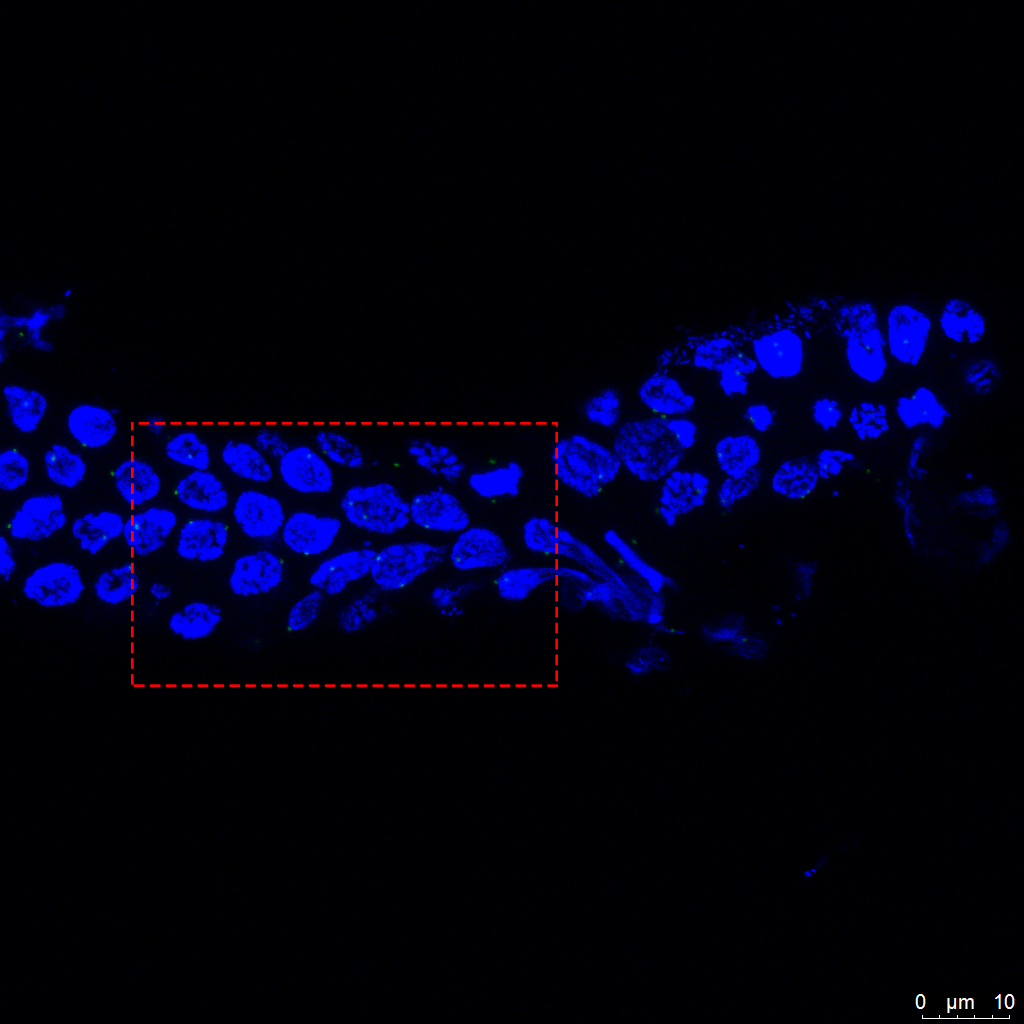

Supplement: Supplementary file 12 — Source data Fig. 9 [file 44319_2025_485_MOESM12_ESM.zip › Figure 9/9K/Fig.9K_sas_6(5D)_TZ.tif]

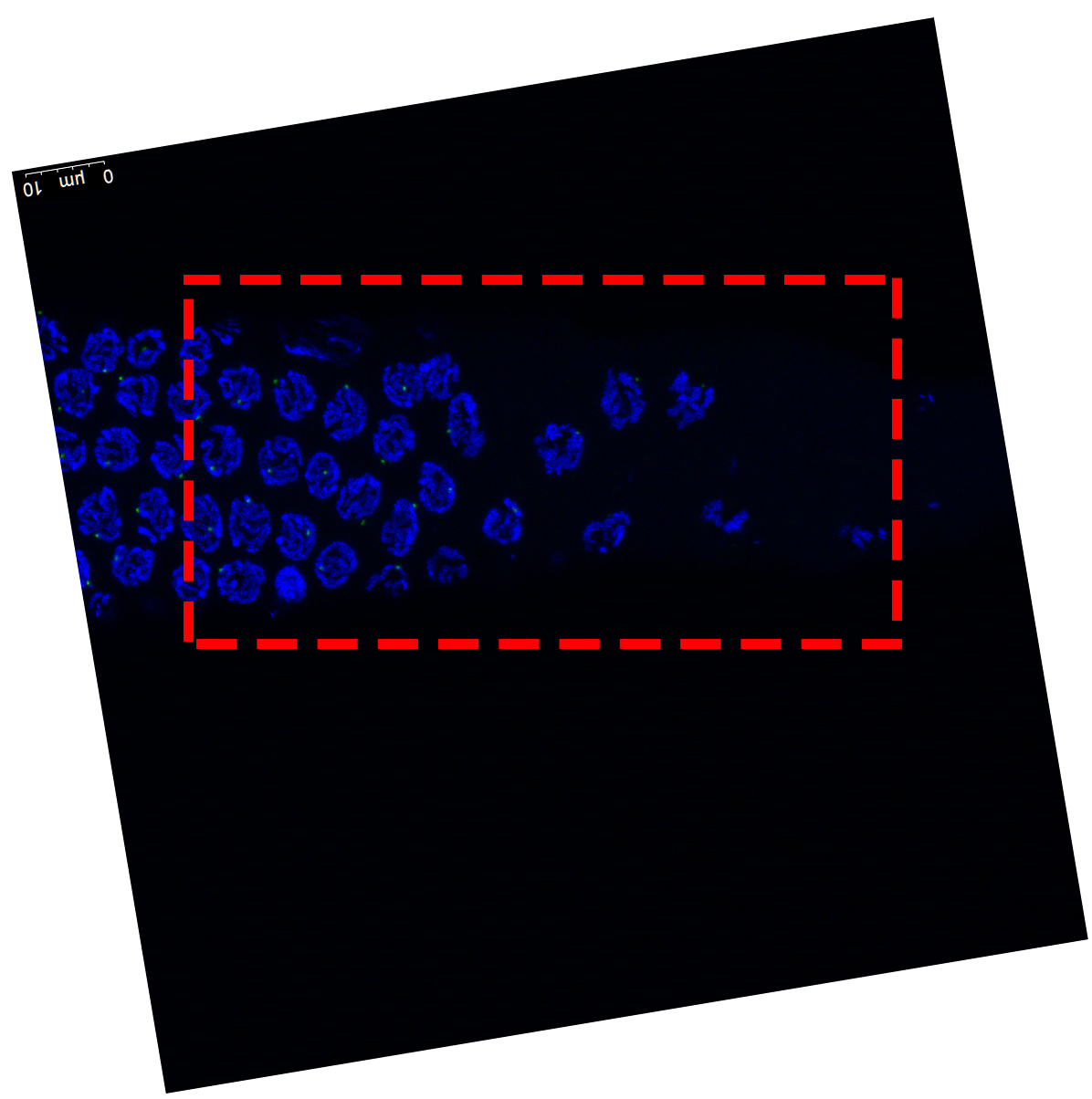

Supplement: Supplementary file 12 — Source data Fig. 9 [file 44319_2025_485_MOESM12_ESM.zip › Figure 9/9K/Fig.9K_wt_LP.tif]

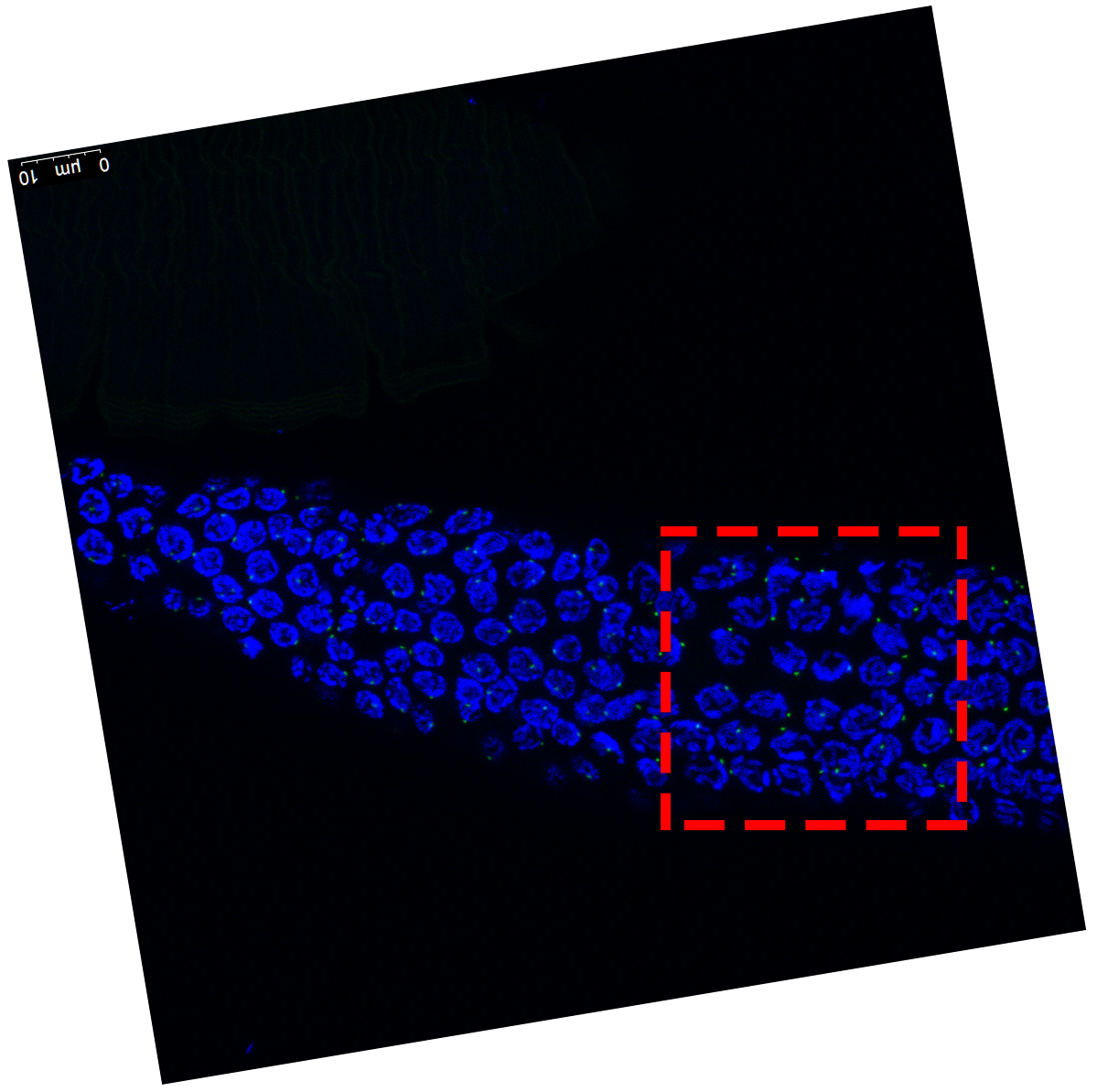

Supplement: Supplementary file 12 — Source data Fig. 9 [file 44319_2025_485_MOESM12_ESM.zip › Figure 9/9K/Fig.9K_wt_TZ.tif]

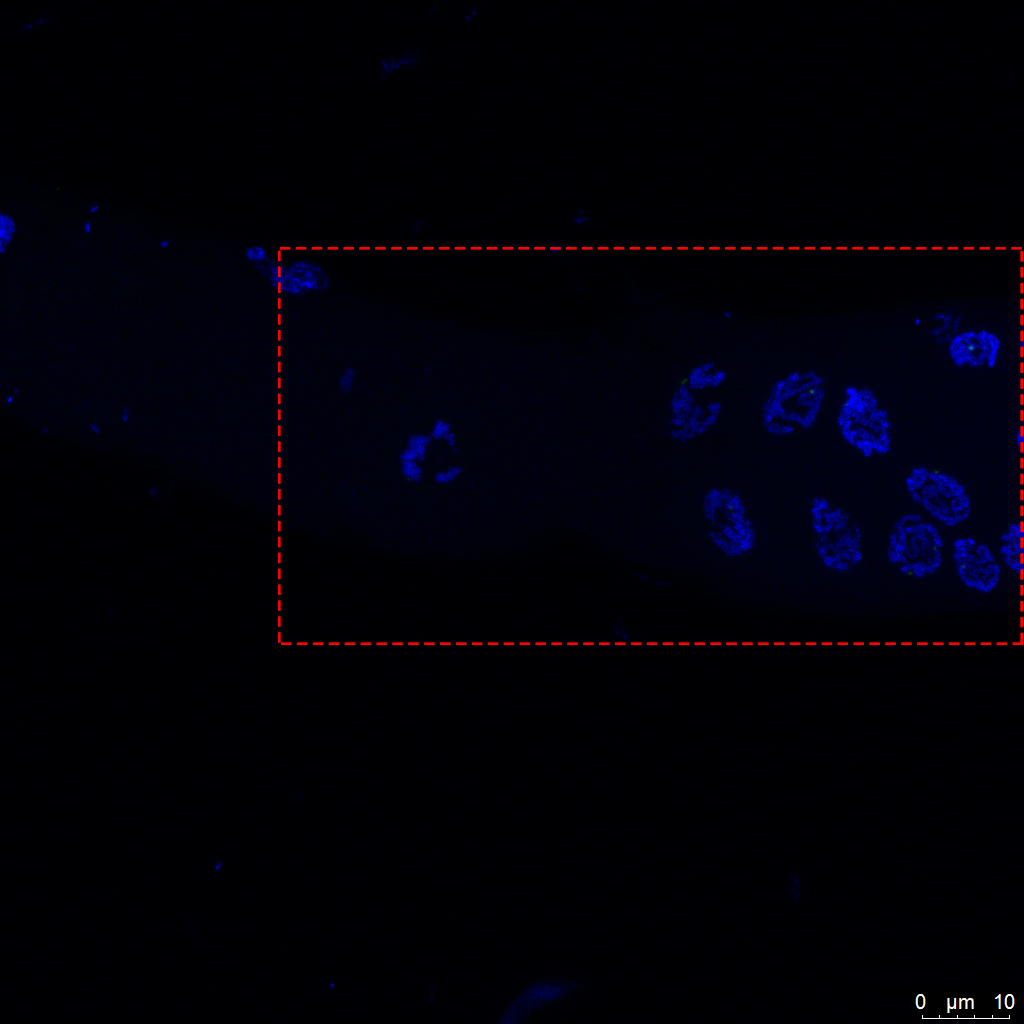

Supplement: Supplementary file 12 — Source data Fig. 9 [file 44319_2025_485_MOESM12_ESM.zip › Figure 9/9K/Fig.9K_sas_6(5D)_LP.tif]

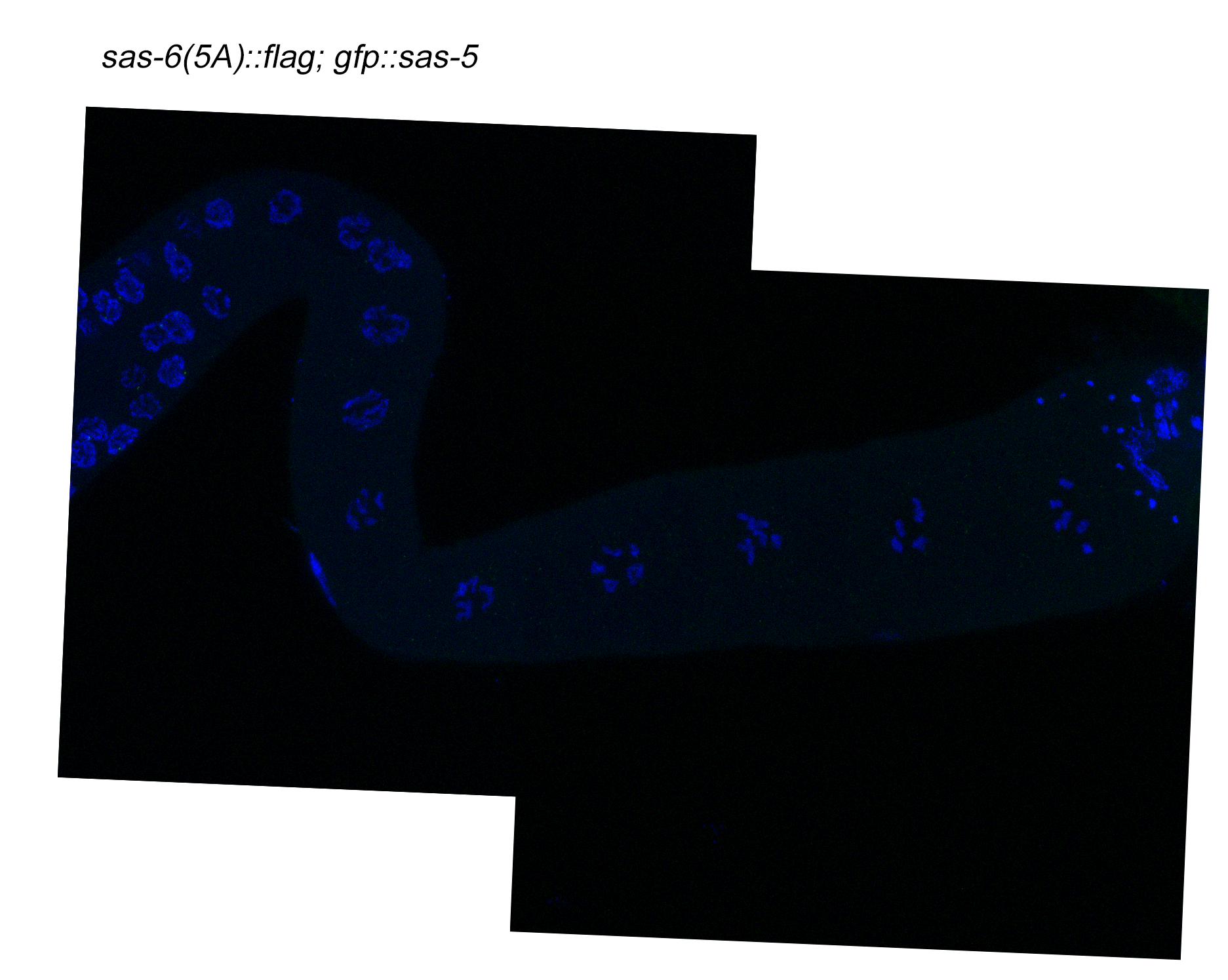

Supplement: Supplementary file 12 — Source data Fig. 9 [file 44319_2025_485_MOESM12_ESM.zip › Figure 9/9D/Fig. 9D_sas_6_gfp_sas_5.tif]

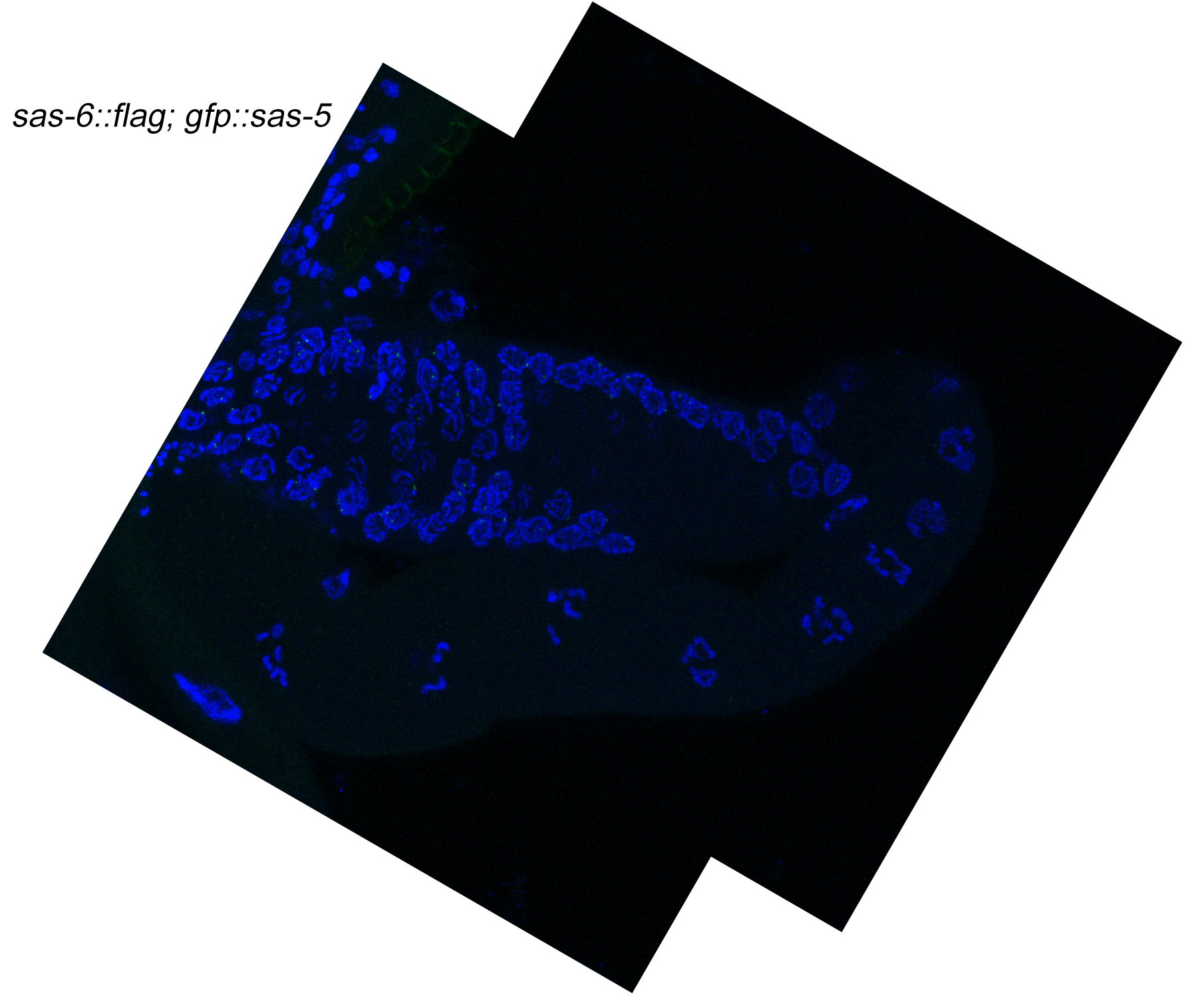

Supplement: Supplementary file 12 — Source data Fig. 9 [file 44319_2025_485_MOESM12_ESM.zip › Figure 9/9D/Fig. 9D_sas_6(5A)_gfp_sas_5.tif]

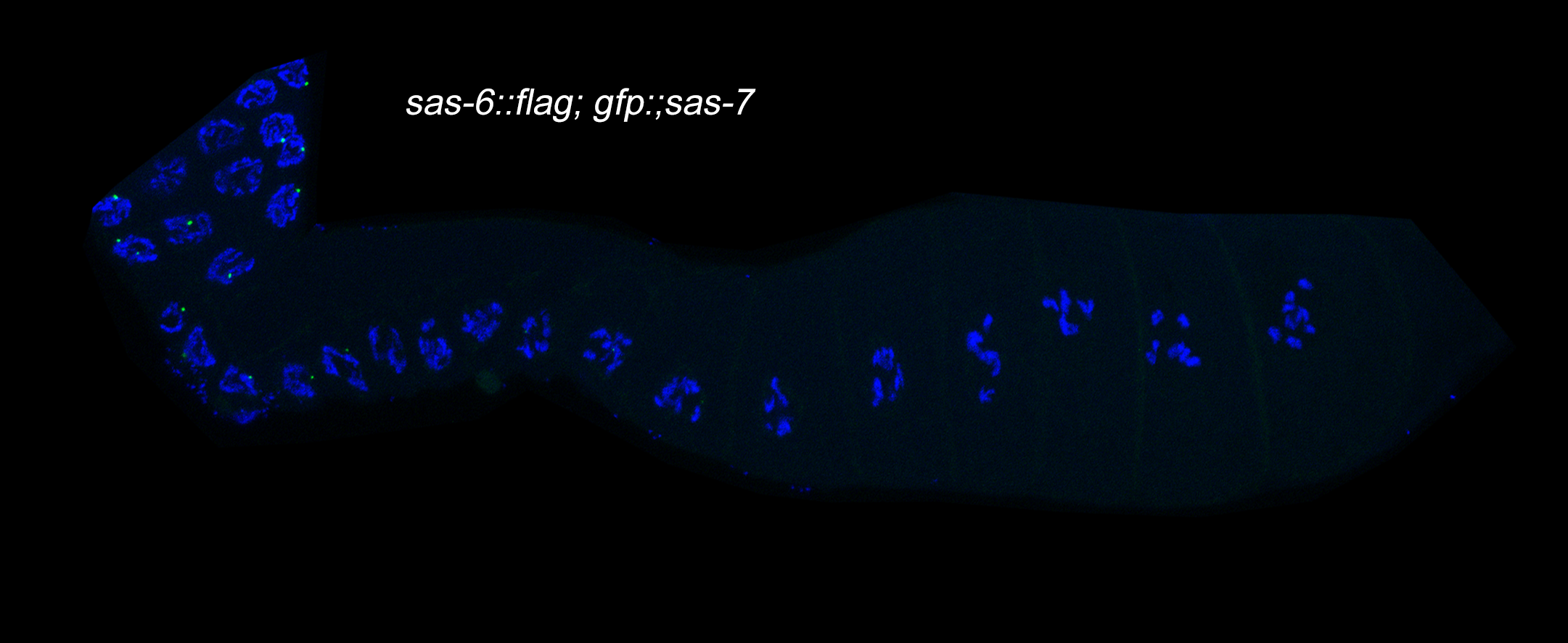

Supplement: Supplementary file 12 — Source data Fig. 9 [file 44319_2025_485_MOESM12_ESM.zip › Figure 9/9F/Fig. 9F_sas_6_gfp_sas_7.tif]

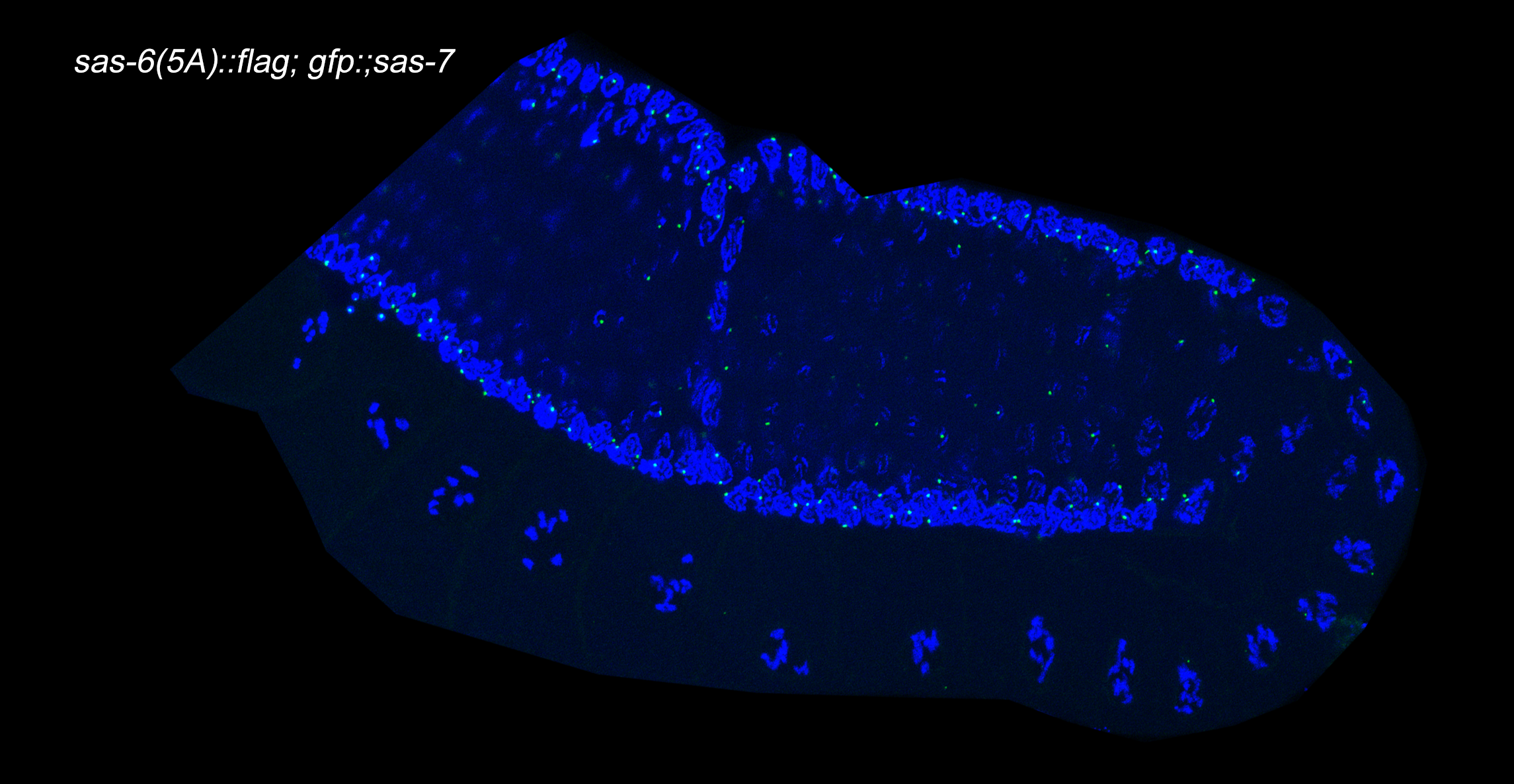

Supplement: Supplementary file 12 — Source data Fig. 9 [file 44319_2025_485_MOESM12_ESM.zip › Figure 9/9F/Fig. 9F_sas_6(5A)_gfp_sas_7.tif]

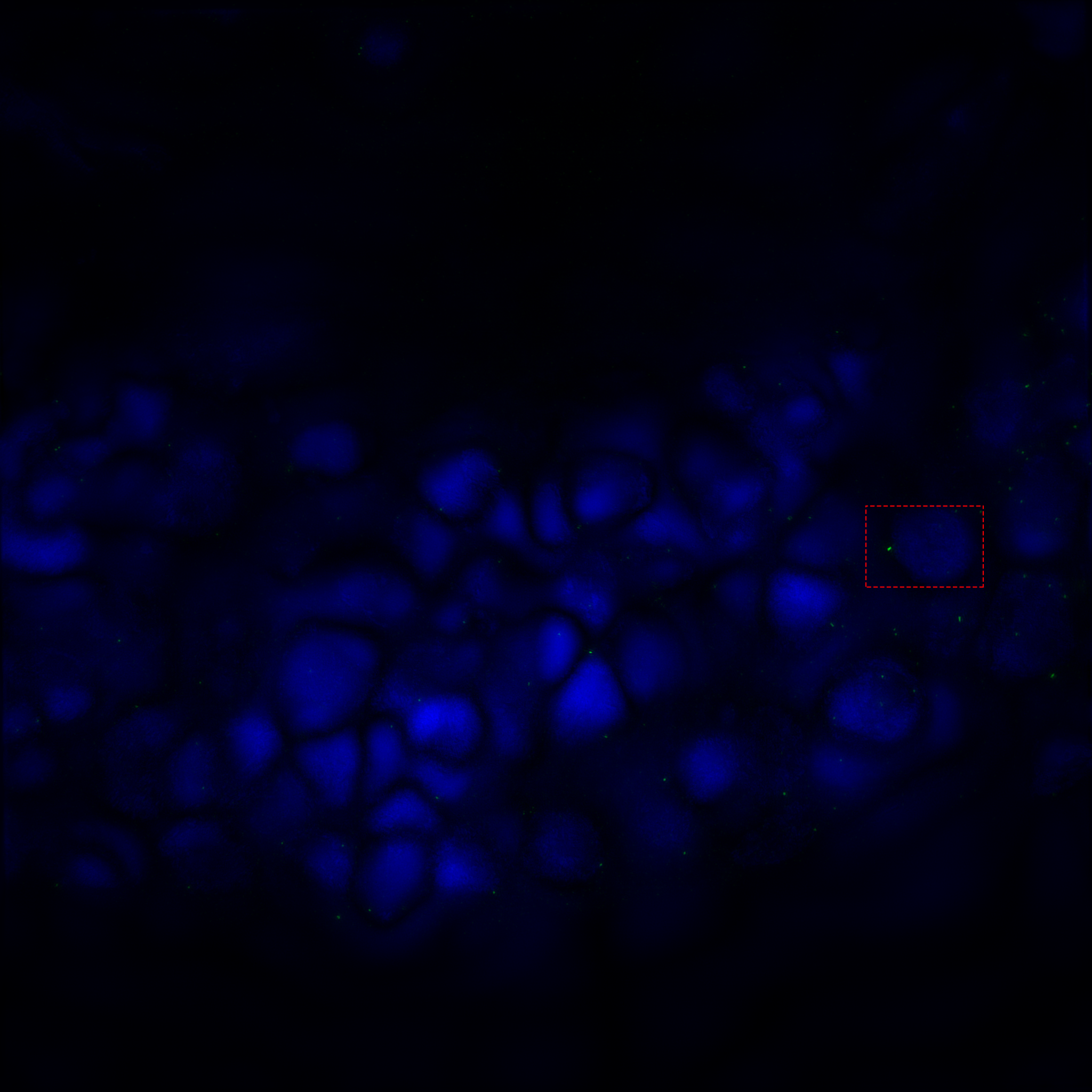

Supplement: Supplementary file 12 — Source data Fig. 9 [file 44319_2025_485_MOESM12_ESM.zip › Figure 9/9H/Fig.9H_sas_6(5D).tif]

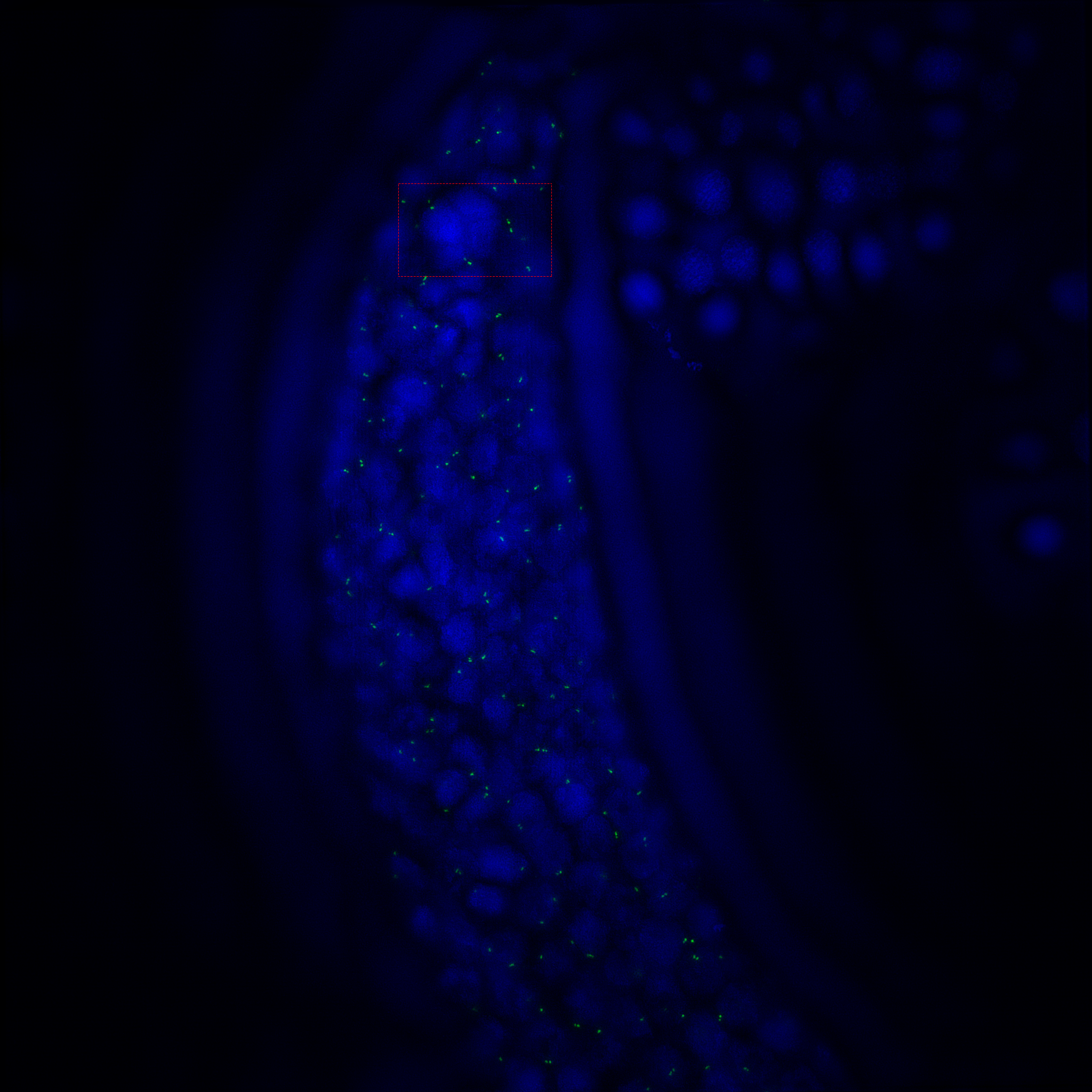

Supplement: Supplementary file 12 — Source data Fig. 9 [file 44319_2025_485_MOESM12_ESM.zip › Figure 9/9H/Fig.9H_wt.tif]

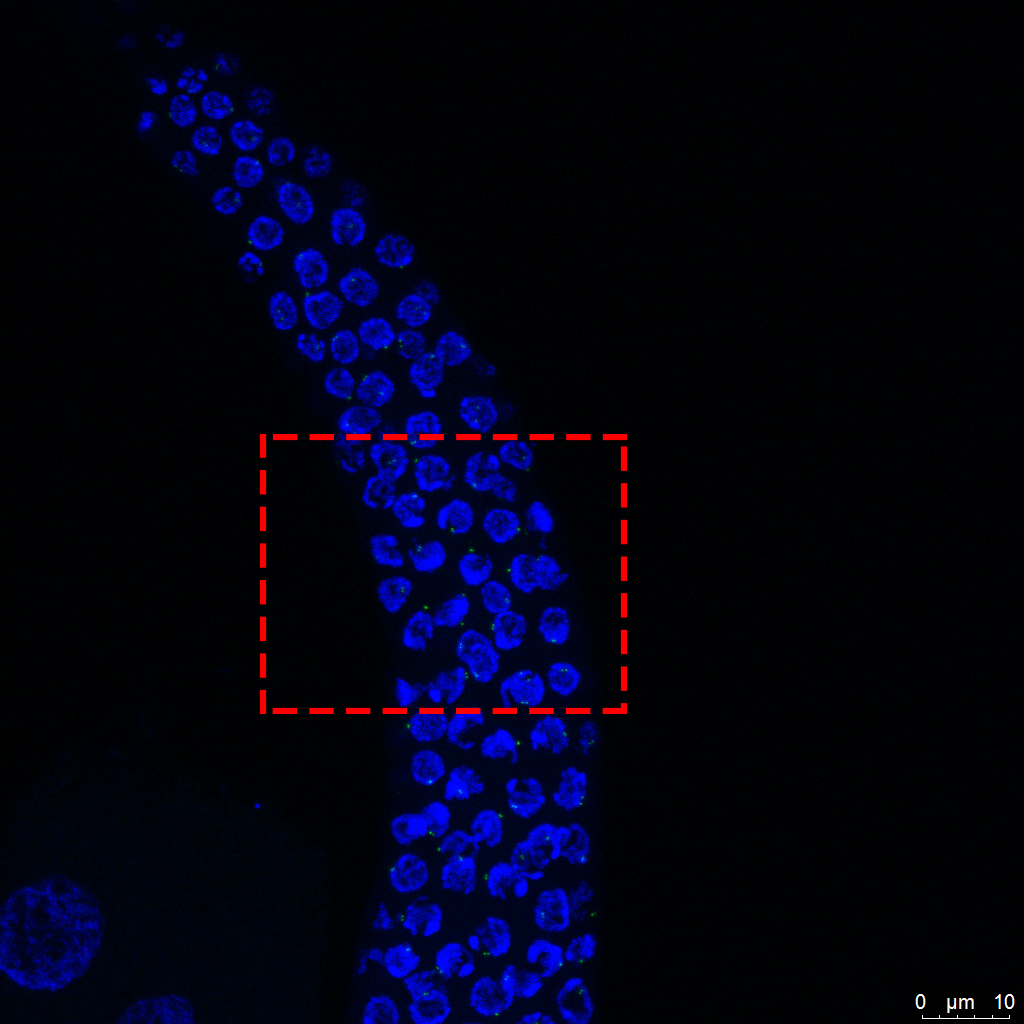

Supplement: Supplementary file 12 — Source data Fig. 9 [file 44319_2025_485_MOESM12_ESM.zip › Figure 9/9I/Fig.9I_wt_TZ.tif]

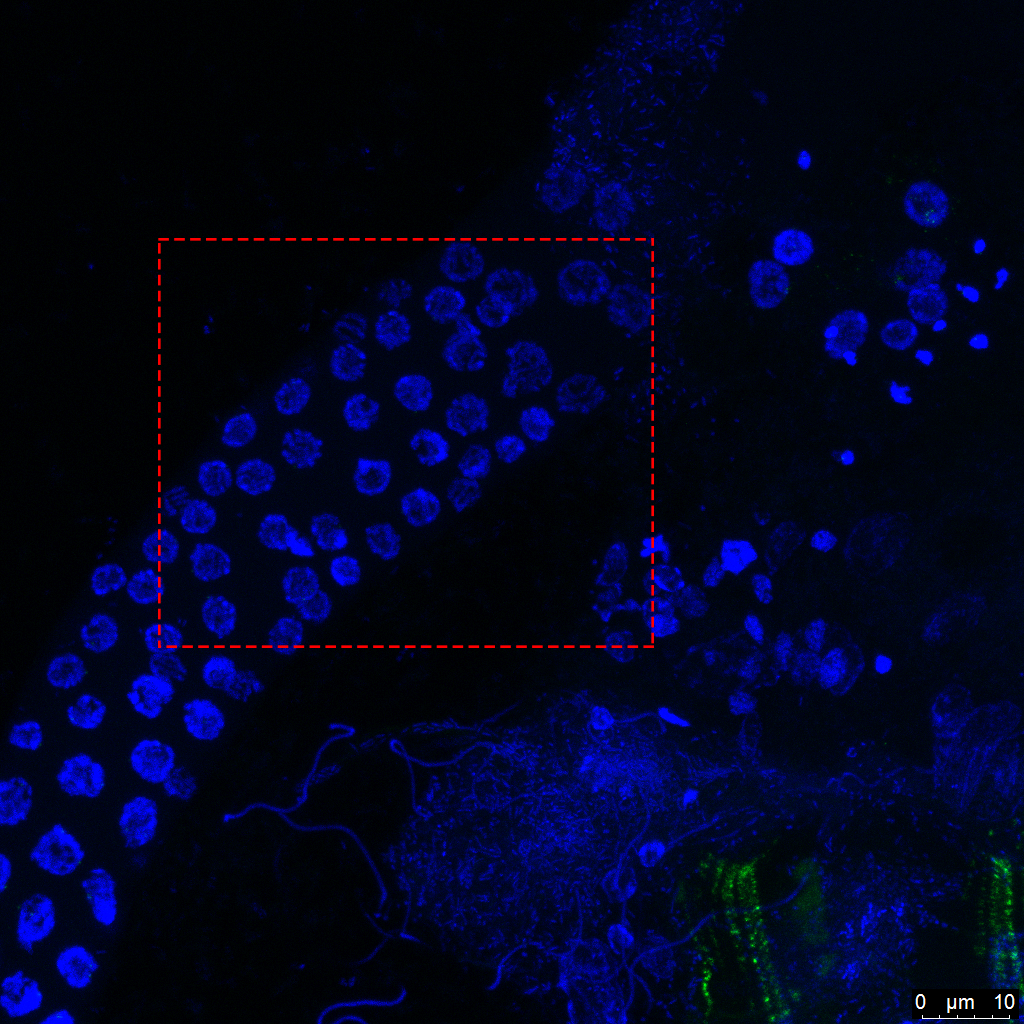

Supplement: Supplementary file 12 — Source data Fig. 9 [file 44319_2025_485_MOESM12_ESM.zip › Figure 9/9I/Fig.9I_sas_6(5D)_LP.tif]

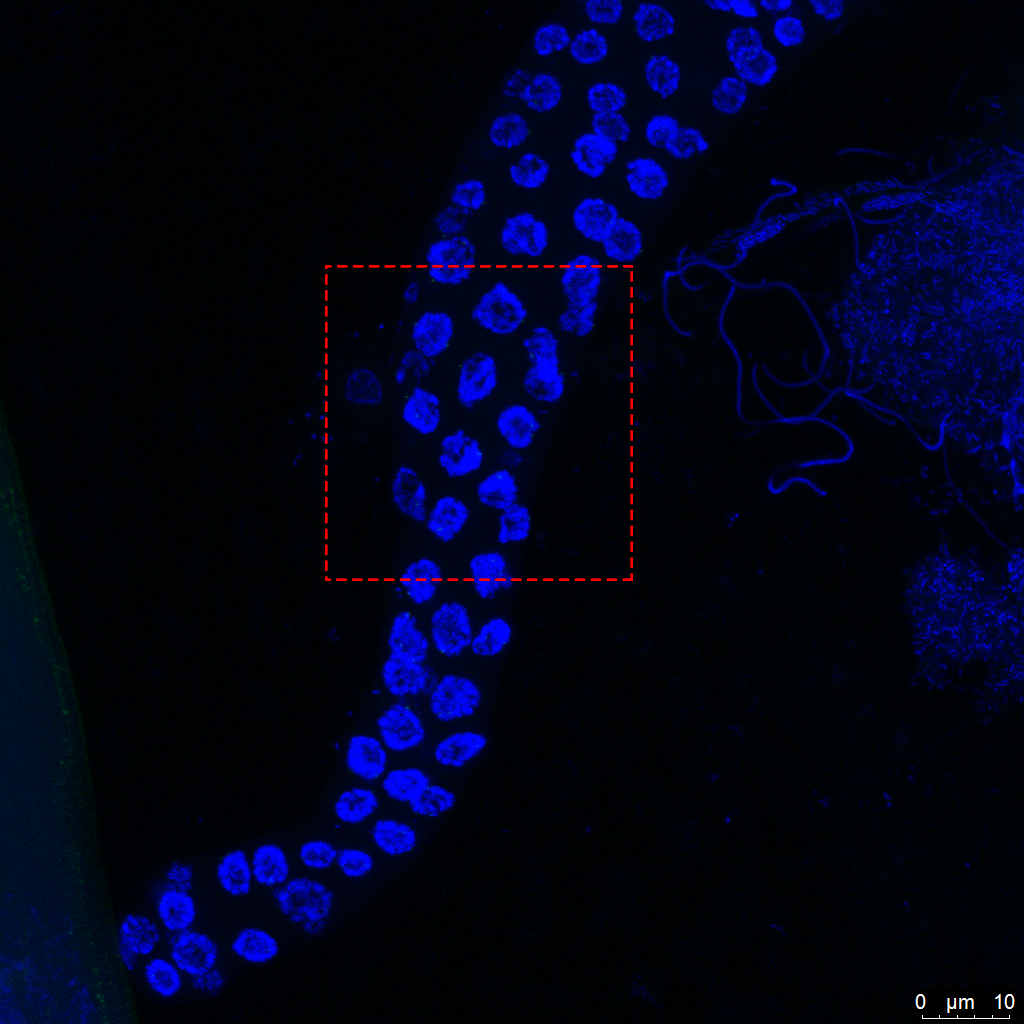

Supplement: Supplementary file 12 — Source data Fig. 9 [file 44319_2025_485_MOESM12_ESM.zip › Figure 9/9I/Fig.9I_sas_6(5D)_TZ.tif]

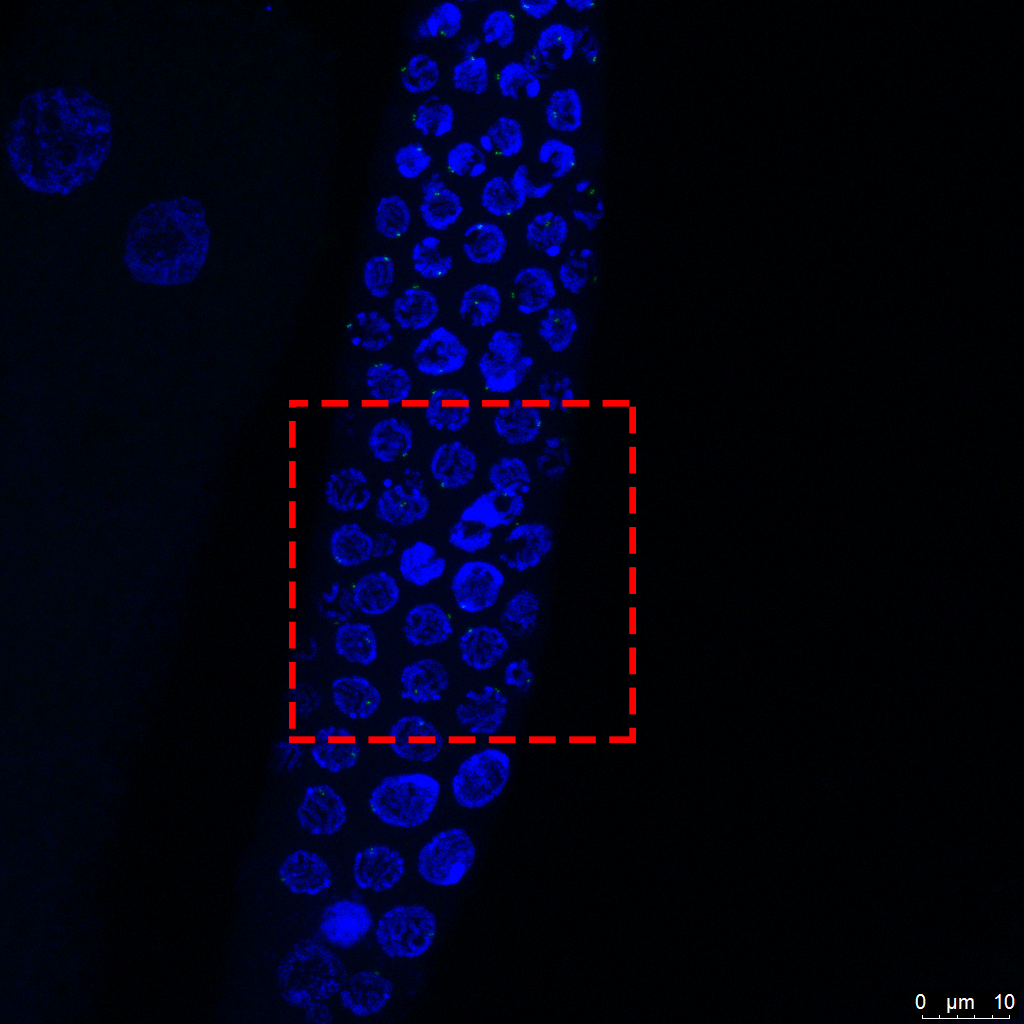

Supplement: Supplementary file 12 — Source data Fig. 9 [file 44319_2025_485_MOESM12_ESM.zip › Figure 9/9I/Fig.9I_wt_LP.tif]
