# Supplementary material for: Dynamic SAS-6 phosphorylation aids centrosome duplication and elimination in C. elegans oogenesis
Source: EMBO Rep. 2025 May 23;26(13):3411–44. doi: 10.1038/s44319-025-00485-7 (PMC12238530; doi:10.1038/s44319-025-00485-7)
Supplement: Supplementary file 13 — Expanded View Figures [file 44319_2025_485_MOESM13_ESM.pdf]

Expanded View Figures

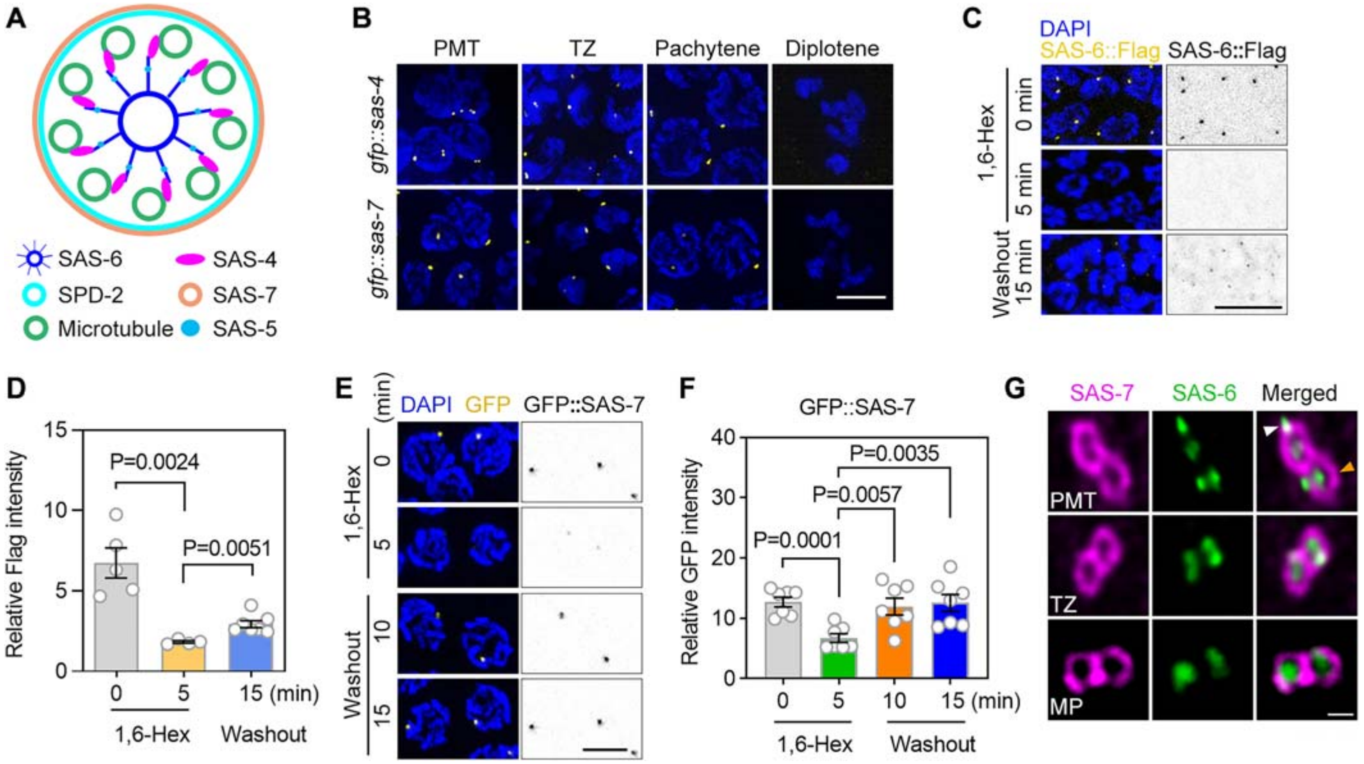

Figure EV1. Centrosome elimination and dynamic properties of centriole proteins during meiotic prophase in *C. elegans*.

(**A**) Schematic representation of the top view of the centriole, with centriole proteins depicted in various colors. (**B**) Immunofluorescence images of germ cells expressing GFP::SAS-4 or GFP::SAS-7 at the indicated meiotic stages. Chromatin was stained with DAPI (blue). In all four gonads imaged per genotype, GFP signals began to decline at diplotene, consistent with representative images shown. Scale bar, 5 µm. (**C**, **D**) Representative images (**C**) and quantification (**D**) of SAS-6::Flag fluorescence intensity before or after treatment of *sas-6::flag* worms with 10% 1,6-hexanediol for 5 min, followed by washout for 15 min. Quantification data are shown as mean ± SEM. *P* values were determined using two-tailed unpaired t-tests. At least four gonads were measured. DNA was stained with DAPI (blue). Scale bar, 10 µm. (**E**, **F**) Representative images (**E**) and quantification (**F**) of GFP::SAS-7 fluorescence intensity before and after treatment of *gfp::sas-7* worms with 10% 1,6-hexanediol for 5 min, followed by washout for 10 min or 15 min. Quantification data are shown as mean ± SEM. *P* values were determined using two-tailed unpaired t-tests. Each dot represents data from a single gonad ($n = 7$ per group). DNA was stained with DAPI (blue). Scale bar, 5 µm. (**G**) Expansion microscopy analysis of GFP::SAS-7 and SAS-6::FLAG during premeiotic tip (PMT) and early meiotic prophase in germ cells of *gfp::sas-7;sas-6::flag* worms. White arrowheads point to procentrioles, and orange arrowheads to centrioles. SAS-6 and SAS-7 localization patterns were consistent across all gonads examined ($n = 3$). TZ, transition zone; MP, mid pachytene. Scale bar, 500 nm.

 © The Author(s)

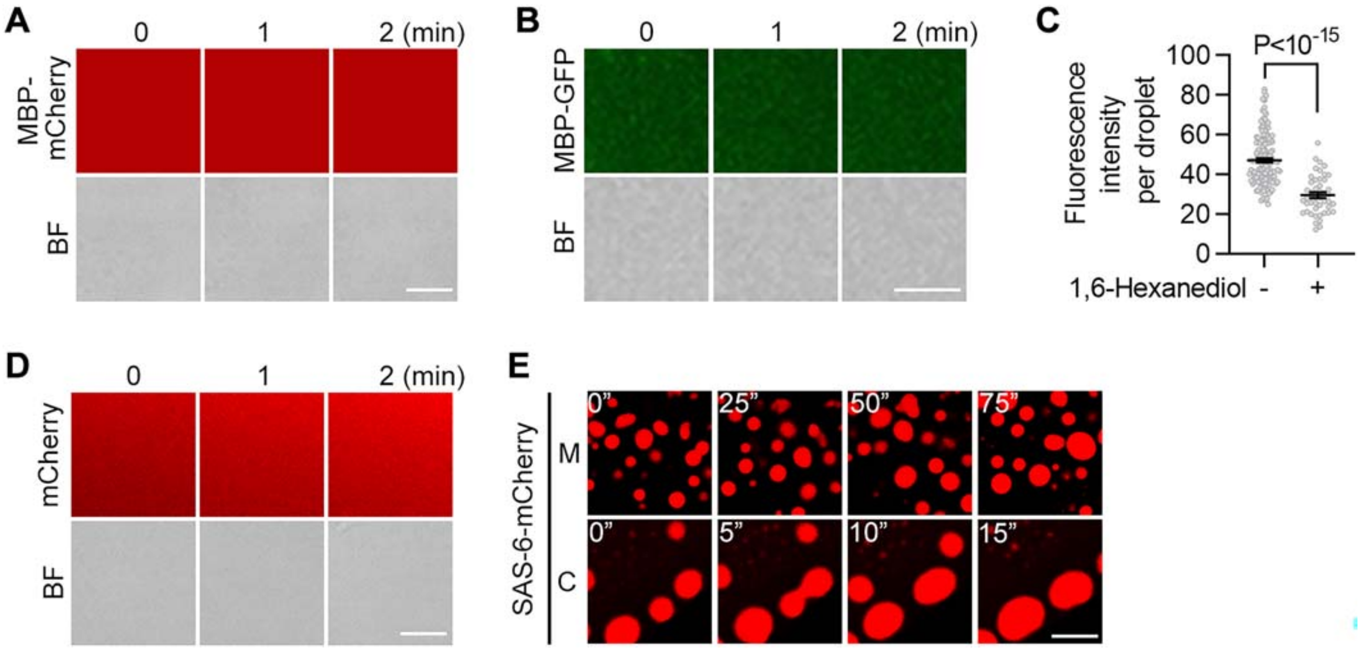

Figure EV2. Purified SAS-6 protein undergoes phase separation in vitro.

(**A**) Fluorescence and bright field (BF) examination of in vitro phase separation of purified MBP-mCherry. Results were consistent across two independent replicates. Scale bar, 5 µm. (**B**) Fluorescence and BF examination of in vitro phase separation of purified MBP-GFP. Results were consistent across two independent replicates. Scale bar, 5 µm. (**C**) Quantification of the fluorescence intensity of SAS6-mCherry droplets with or without 10% 1,6-hexanediol treatment as performed in Fig. 2G. Data are shown as mean ± SEM. *P* values were determined using two-tailed unpaired t-tests. Droplets analyzed: −, $n = 150$; +, $n = 50$. (**D**) Fluorescence and BF examination of in vitro phase separation of purified mCherry. Results were consistent across four independent replicates. Scale bar, 5 µm. (**E**) Fluorescence images showing that SAS-6-M-mCherry and SAS-6-C-mCherry droplets grow over time. Phase separation capacity of these proteins was consistent across three independent replicates. Scale bar, 5 µm.

© The Author(s)

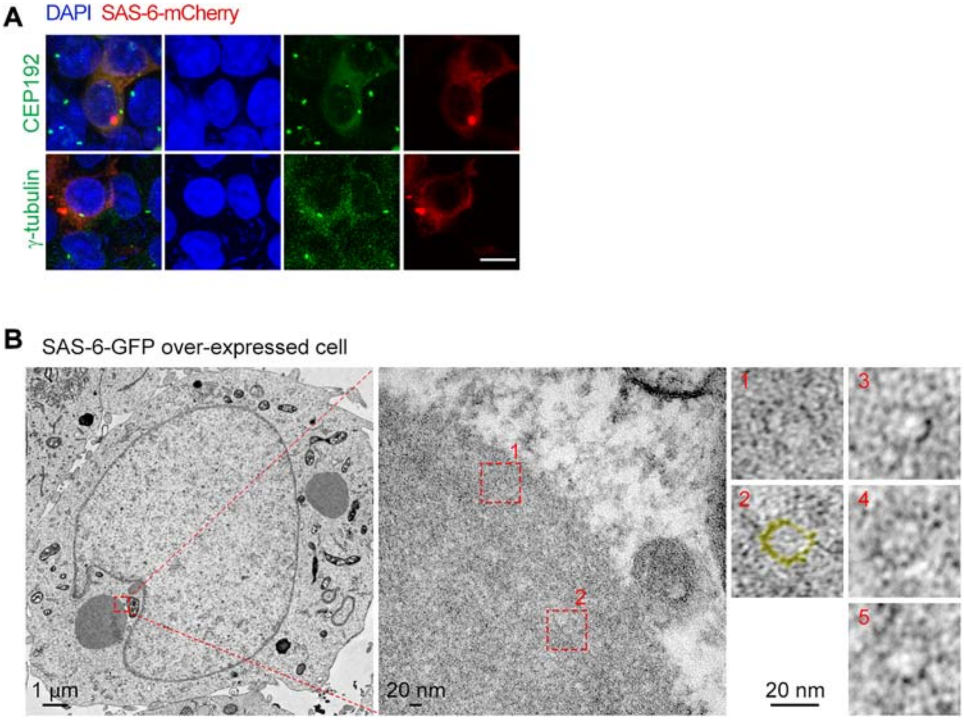

Figure EV3. SAS-6 droplet formation in HEK293T cells.

(**A**) Representative images showing immunostaining of endogenous centriolar proteins (green) and exogenously overexpressed SAS-6-mCherry (red) in HEK293T cells. The relative localization of SAS-6-mCherry droplets and centriolar proteins was consistent across three biological replicates (≥ 10 transfected cells analyzed). Scale bar, 10 µm. (**B**) Representative negative-stain electron micrograph of SAS-6-GFP condensates formed in HEK293T cells. Examples of cartwheel ring structures are shown on the right. The first two examples (1 and 2) are from the field on the left, and the last three (3, 4, and 5) are from other fields. A total of ten spherical droplets, each 0.8-2.0 µm in diameter, were imaged, with an average of approximately 8 cartwheel-like structures observed per droplet. Scale bars are labeled in the figure.

 © The Author(s)

A ***syb1706: sas-6::degron::flag::ha::flag::ha***

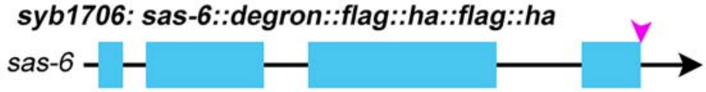

Inserted sequence (flanking sequence):

GCTCAACGACCTAAAGATCCAGCCAAACCTCCGGCCAAGGCACAAGTTGTGGGATGGCCACCGGTGAGATCATACCGGAA
GAACGTGATGGTTTCCTGCCAAAAATCAAGCGGTGGCCCGGAGGCGGCGGCGTTCGTGAAGGATTACAAGGACGATGACA
AGTACCCATACGATGTTCCAGATTACGCTGATTACAAGGACGATGACAAGTACCCATACGATGTTCCAGATTACGCTTAA
aatctt

syb3988: sas-6(5A)::degron::flag::ha::flag::ha

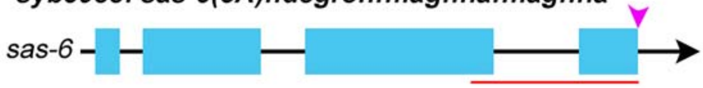

Repair template sequence:

GCGCCAGCTCCTAGTGGGCTTCCCGGGCTTCAGACCGGACTAACAAATAGGCTTGCACCATCATTCAAGCCTGTTCTTGG
ACCGCATGCTCCATATGGAGCTAATTTGAACTCGgttagtagaagttctacgaaatgagctacattatcgatttttccgg
caatttccaattttcacacaatttttgagcatttttccgaactgtttcaactcaaaaaaatattttaatttgttcaaatt
tcagCGAACTCCATTCCGTGACAATACAACTCTTAATTTCCAAAATTCGACAATTGCAGCTCCTCATGCTTTTCGTTTCA
ACAGTCAACTAATCGCCGACGAAACTACTGGTTCAAGTGTGACGAACGCCCCACCCGCTCAACGACCTAAAGATCCAGCC
AAACCTCCGGCCAAGGCACAAGTTGTGGGATGGCCACCGGTGAGATCATACCGGAAGAACGTGATGGTTTCCTGCCAAAA
ATCAAGCGGTGGCCCGGAGGCGGCGGCGTTCGTGAAGGATTACAAGGACGATGACAAGTACCCATACGATGTTCCAGATT
ACGCTGATTACAAGGACGATGACAAGTACCCATACGATGTTCCAGATTACGCT

syb5687: sas-6(5D)::degron::4xflag

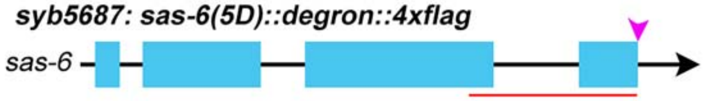

Repair template sequence:

GATCCAGCTCCTAGTGGGCTTCCCGGGCTTCAGACCGGACTAACAAATAGGCTGGATCCATCATTCAAGCCTGTTCTTGG
ACCCCACGATCCATATGGAGCTAATTTGAACTCGgttagtagaagttctacgaaatgagctacattatcgatttttccgg
caatttccaattttcacacaatttttgagcatttttccgaactgtttcaactcaaaaaaatattttaatttgttcaaatt
tcagCGCACGCCATTCCGTGACAATACAACTCTTAATTTCCAAAATTCGACAATTGCAGATCCTCATGCTTTTCGTTTCA
ACAGTCAACTAATCGCCGACGAAACTACTGGTTCAAGTGTGACGAACGACCCACCCGCTCAACGACCTAAAGATCCAGCC
AAACCTCCGGCCAAGGCACAAGTTGTGGGATGGCCACCGGTGAGATCATACCGGAAGAACGTGATGGTTTCCTGCCAAAA
ATCAAGCGGTGGCCCGGAGGCGGCGGCGTTCGTGAAGGATTACAAGGACGATGACAAGTACCCATACGATGTTCCAGATT
ACGCTGATTACAAGGACGATGACAAGTACCCATACGATGTTCCAGATTACGCT

B

404 AQRFSPAPSGLPGLQTGLTNR 424

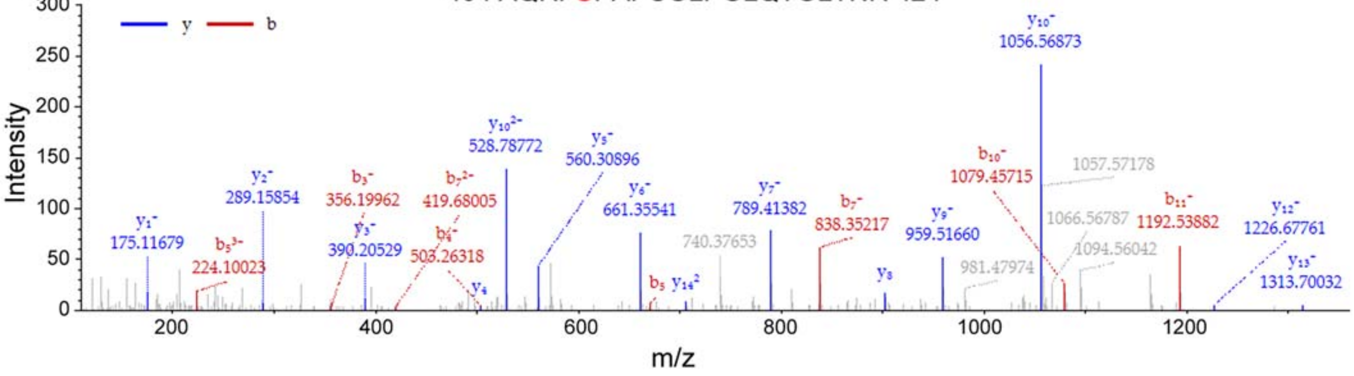

◀ **Figure EV4. Creation of *sas-6::degron* and mutant strains by the CRISPR/Cas9 method and detection of SAS-6 phosphorylation in *C. elegans.***

(**A**) Top: schematic diagram of genome editing to create the endogenously tagged *sas-6::degron::flag* strain. The sequence encoding the degron and FLAG tag is shown in pink. Depending on the context, the genotypes of this strain are labeled as *sas-6::degron::flag*, *sas-6::degron*, or *sas-6::flag* in this study. Middle and Bottom: schematic diagrams of genome editing to create the *sas-6(5A)* and *sas-6(5D)* mutants. Mutation sites are indicated in red, and inserted tagging sequences are shown in pink. The genotypes of these strains are labeled as *sas-6(5 A)* or *sas-6(5 A)::flag*, and *sas-6(5D)* or *sas-6(5D)::flag*, respectively, in this study. (**B**) Mass spectrometry identification of the SAS-6 peptide with Ser408 phosphorylation. Mass spectrometry analysis was performed with anti-FLAG immunoprecipitation of worm lysates from synchronized *sas-6::flag* young adults.

 © The Author(s)

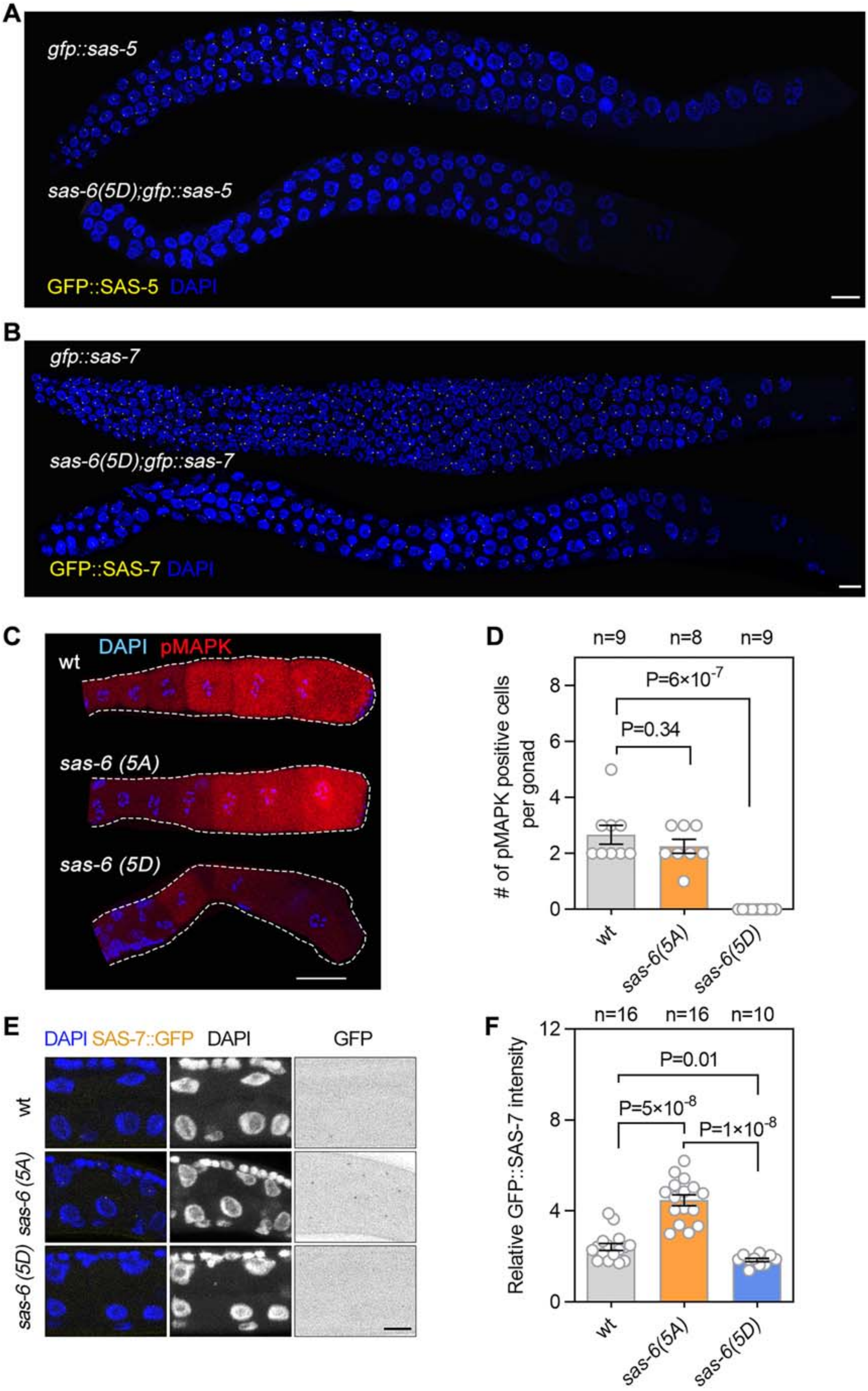
A
gfp::sas-5
sas-6(5D);gfp::sas-5
GFP::SAS-5 DAPI
B
gfp::sas-7
sas-6(5D);gfp::sas-7
GFP::SAS-7 DAPI
C
DAPI pMAPK
wt
sas-6 (5A)
sas-6 (5D)
D
n=9
n=8
n=9
P=6×10-7
P=0.34
# of pMAPK positive cells per gonad
0
2
4
6
8
wt
sas-6(5A)
sas-6(5D)
E
DAPI SAS-7::GFP
DAPI
GFP
wt
sas-6 (5A)
sas-6 (5D)
F
n=16
n=16
n=10
P=0.01
P=5×10-8
P=1×10-8
Relative GFP::SAS-7 intensity
0
4
8
12
wt
sas-6(5A)
sas-6(5D)

Figure EV5. Centrosome duplication and elimination in germline and intestine of *sas-6* mutant worms.

(**A**) Projection images showing GFP::SAS-5 (yellow) focus formation in the germlines of the indicated genotypes. DNA was stained with DAPI (blue). Scale bar, 10 μm. (**B**) Projection images showing GFP::SAS-7 (yellow) focus formation in the germlines of the indicated genotypes. DNA was stained with DAPI (blue). Scale bar, 10 μm. (**C**, **D**) Immunofluorescence images (**C**) and quantification (**D**) of phosphorylated MAPK (pMAPK) positive oocytes per gonad arm. Quantification data are shown as mean ± SEM. *P* values were determined using two-tailed unpaired t-tests. The numbers of worms measured are indicated. (**E**) Maintenance of GFP::SAS-7 foci in intestinal cells of L2 worms with the indicated genotypes. Intestinal cells were distinguished from the other cells based on their location and nuclear morphology. (**F**) Quantification of the SAS-7 focus intensity in the intestine of the indicated genotype. Data are shown as mean ± SEM. Each dot represents the average fluorescence intensity of GFP::SAS-7 across all intestinal cells of a worm. *P* values were determined using two-tailed unpaired t-tests. The numbers of worms measured are indicated.

 © The Author(s)
